# The head-regeneration transcriptome of the planarian *Schmidtea mediterranea*

**DOI:** 10.1186/gb-2011-12-8-r76

**Published:** 2011-08-16

**Authors:** Thomas Sandmann, Matthias C Vogg, Suthira Owlarn, Michael Boutros, Kerstin Bartscherer

**Affiliations:** 1 Division Signaling and Functional Genomics, German Cancer Research Center (DKFZ), Im Neuenheimer Feld 580, D-69120 Heidelberg, Germany; 2 CellNetworks Cluster of Excellence, Heidelberg University, Im Neuenheimer Feld 267, 69120 Heidelberg, Germany; 3 Max Planck Research Group Stem Cells and Regeneration, Max Planck Institute for Molecular Biomedicine, Von-Esmarch-Str. 54, 48149 Münster, Germany; 4 Department of Cell and Molecular Biology, Faculty of Medicine Mannheim, Heidelberg University, Ludolf-Krehl-Straße 13-17, 68167 Mannheim, Germany

## Abstract

**Background:**

Planarian flatworms can regenerate their head, including a functional brain, within less than a week. Despite the enormous potential of these animals for medical research and regenerative medicine, the mechanisms of regeneration and the molecules involved remain largely unknown.

**Results:**

To identify genes that are differentially expressed during early stages of planarian head regeneration, we generated a *de novo *transcriptome assembly from more than 300 million paired-end reads from planarian fragments regenerating the head at 16 different time points. The assembly yielded 26,018 putative transcripts, including very long transcripts spanning multiple genomic supercontigs, and thousands of isoforms. Using short-read data from two platforms, we analyzed dynamic gene regulation during the first three days of head regeneration. We identified at least five different temporal synexpression classes, including genes specifically induced within a few hours after injury. Furthermore, we characterized the role of a conserved Runx transcription factor, *smed-runt-like1*. RNA interference (RNAi) knockdown and immunofluorescence analysis of the regenerating visual system indicated that *smed-runt-like1 *encodes a transcriptional regulator of eye morphology and photoreceptor patterning.

**Conclusions:**

Transcriptome sequencing of short reads allowed for the simultaneous *de novo *assembly and differential expression analysis of transcripts, demonstrating highly dynamic regulation during head regeneration in planarians.

## Background

The limited regenerative capabilities of humans call for therapies that can replace or heal wounded tissues. The treatment of neurodegenerative diseases has been a major focus of regenerative medicine, as these diseases can cause irreversible damage to the central nervous system (CNS). It is crucial, therefore, to understand the molecular mechanisms of regeneration and the intrinsic and extrinsic signals that induce and promote this process.

Planarian flatworms are one of the few animals that can regenerate their CNS. Planarians are free-living Platyhelminthes with a relatively simple CNS consisting of a bilaterally symmetrical brain made from two cephalic ganglia, and two longitudinal ventral nerve cords, which extend along the body axis and send out axonal projections into nearly any micrometer of the body (reviewed in [1]). Despite its relatively simple morphology, the planarian brain is highly complex at the cellular level, and consists of a large number of different neuronal cell types [2-4]. Many genes expressed in the planarian CNS are highly conserved in humans [5].

Planarians are characterized by their large pool of pluripotent adult stem cells that facilitate the regeneration of whole animals from only small pieces of their body (reviewed in [6]). Strikingly, planarians can develop a new head within a week. This process can be classified into several distinct events. First, wounding induces a generic, body-wide proliferation response of stem cells within the first 6 hours. Attracted by as yet unidentified guidance signals possibly released from cells at the site of tissue loss, stem cells accumulate at the wound within 18 hours. This response is regeneration-specific and is not detected in wounded animals that have not experienced any tissue loss [7]. A second, regeneration-specific, localized proliferation response that reaches its peak after 2 days of regeneration generates stem cell progeny that contribute to the growth of the blastema. This progeny starts to differentiate into different cell types between 1 and 2 days after the cut [7]. In decapitated animals, brain rudiment is detected within 24 hours, which continuously grows and develops into a properly patterned bi-lobed brain. The first clusters of photoreceptor neurons appear between 2 and 3 days, dorsally to the brain. With photoreceptors-to-brain, and brain-to-ventral nerve cord connections, structural and functional recovery is completed between 4 to 7 days of regeneration [8,9].

Their unique regenerative properties in combination with the efficiency of gene knockdown by RNA interference (RNAi) have made planarians an attractive model organism for investigating the molecular processes that underlie regeneration and stem cell biology *in vivo *(reviewed in [10,11]). One of the most frequently used species in planarian research is *Schmidtea mediterranea*. These planarians were collected in the Mediterranean area and have been maintained in laboratories worldwide for many years, often as clonal lines originating from a single wild animal. They reproduce sexually or asexually by fission, are 0.1 to 2 cm in size, and have a diploid genome of approximately 850 Mb, arranged into four chromosomes [12].

Based on the *S. mediterranea *genome sequencing project [13], approximately 30,000 genes have been predicted using the MAKER genome annotation pipeline [12]. However, the repetitiveness and A/T richness of the genome, and the fragmentation of its assembly into approximately 43,000 supercontigs, make genome annotations difficult, resulting in many incomplete, redundant and error-laden predictions.

To overcome these limitations and to discover potential regulators of planarian head regeneration, we constructed an annotated head regeneration transcriptome library by *de novo *assembly of hundreds of millions of short raw reads generated by next generation sequencing without genomic sequence information. We used this library to map and count expressed sequence reads from different stages of regeneration and identified hundreds of genes showing differential expression at different time points during the first 3 days following decapitation. We show that an early growth response (EGR)-like gene is transcriptionally induced as an early response to injury. In addition, we further characterized the biological function of a putative Runx transcription factor, *smed-runt-like1*, which controls photoreceptor patterning during the regeneration of the visual system. Our study demonstrates that next generation sequencing is a powerful tool for gene function discovery even in organisms with no or only poorly annotated genomes.

## Results

### A time course of planarian head regeneration

**Figure 1:**
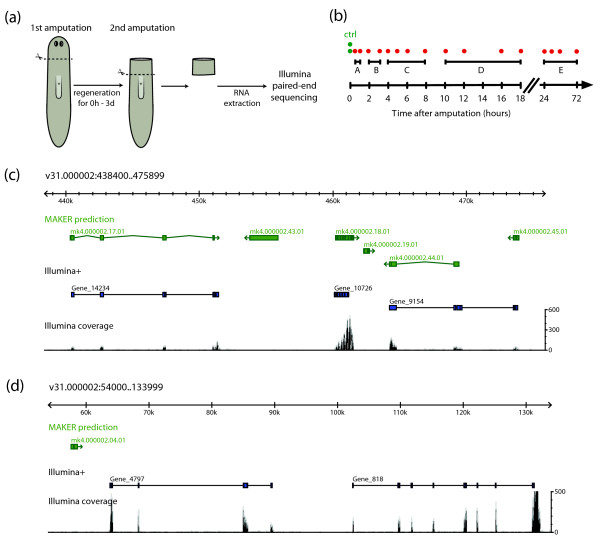
**Next generation sequencing reveals the planarian head regeneration transcriptome**. **(a) **Schematic overview of the two-step amputation and sample collection approach. **(b) **Schematic overview of the regeneration time course. Individual samples are indicated as red dots, which were analyzed in five pooled sequencing libraries (black lines, A to E). Two independent control samples were taken immediately after amputation (green dots). **(c, d) **Examples of Illumina transcriptome sequencing (RNAseq) reads mapped with Tophat to regions on genomic supercontig v31.000002. The short read coverage, calculated from reads from all sequenced samples, is shown in black. Green gene models represent MAKER predictions; blue models exemplify the results of the *de novo *assembly (Illumina+).

To study the dynamic changes in gene expression during head regeneration, we collected samples at 16 different time points between 30 minutes and 3 days after head amputation, as well as two control samples frozen immediately after decapitation. To facilitate the detection of genes expressed in or proximal to the blastema, we extracted mRNA specifically from anterior pre-pharyngeal tissue rather than from whole animals (Figure 1a). We prepared seven fragmented cDNA libraries for 2 × 36-bp paired-end sequencing, each including material from two to four pooled samples (Figure 1b). Sequencing on an Illumina Genome Analyzer II yielded more than 336 million raw reads (168 million read pairs), of which 274 million (81.5%) could be mapped to supercontigs of the preliminary *S. mediterranea *genome assembly using Tophat [14]. While good correspondence between MAKER gene predictions and Illumina read coverage was observed in some cases (Figure 1c), we frequently detected transcription from genomic regions lacking annotation (Figure 1d). To identify differentially expressed loci independent of prior gene annotation, we therefore used our short-read transcriptome sequencing (RNAseq) data to assemble the expressed transcriptome *de novo*.

### *De novo *assembly with Velvet and Oases

**Figure 2:**
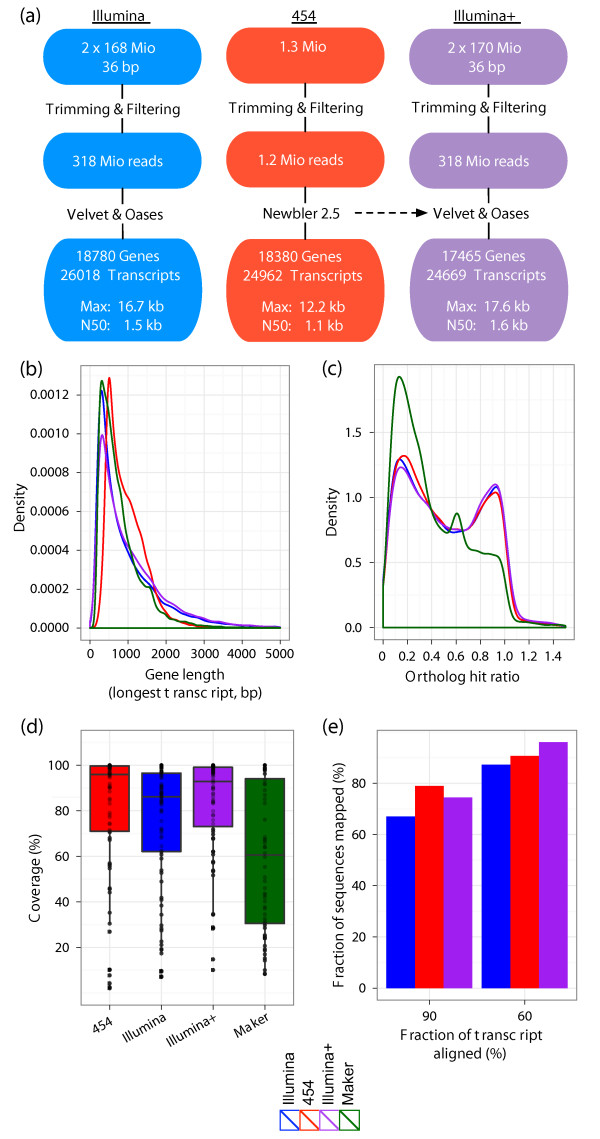
***De novo *assembly of the planarian head regeneration transcriptome**. **(a) **Schematic overview of the assembly strategies, using only 2 × 36-bp paired-end Illumina reads (blue), only 454 reads (red), or an assisted assembly of Illumina reads using transcripts previously assembled from 454 data as scaffolds (purple). Quality metrics shown include longest sequences in each assembly and the length N50, for which 50% of all bases are contained in transcripts at least as long as N50. **(b) **Kernel densities of the length distributions for sequences assembled only from Illumina data (blue), 454 data (red), Illumina data and 454 isotig scaffolds (purple), or for computationally predicted transcripts by MAKER (green). For multi-isoform loci, only the longest isoform was considered. **(c) **Kernel densities of ortholog hit ratios obtained by comparing sequences from the different assemblies or computational prediction to the *Schistosoma mansoni *proteome using blastx. For multi-isoform loci, only the longest isoform was considered. Colors as in (b). **(d) **Coverage of the 125 complete cDNA sequences from *S. mediterranea *available from GenBank by the best reciprocal blat hit from each dataset. For multi-isoform loci, only the longest isoform was considered. The boxplot indicates the 75th, 50th (median) and 25th percentile of cDNA coverage. In addition, individual points show the full coverage distribution for all reciprocal best hits (454, *n *= 77; Illumina, *n *= 86; Illumina+, *n *= 75; MAKER, *n *= 60). **(e) **Fraction of sequences from the different assemblies that could be aligned over 90% or 60% of their total length to a single genomic supercontig using blat. Colors as in (b).

The assembly of transcripts needs to account for alternative splicing events as well as post-transcriptional sequence modifications, for example, poly-adenylation of RNAs. After filtering the dataset for low base-calling quality, we employed a two-step strategy to assemble the remaining 318 million (94.6%) high quality reads: we first generated a preliminary assembly using Velvet [15], incorporating 187 million reads (58.8%), followed by the construction of transcripts by Oases [16]. We obtained 26,018 transcripts, corresponding to 18,780 non-overlapping sequences (Figure 2a; Illumina) with a minimum length of 200 bp.

To assess the quality of this assembly, we first compared it to results obtained with a complementary sequencing technique, Roche 454 sequencing. Recently, two independent studies generated 454 sequence datasets from different stages and tissues of *S. mediterranea *[17,18]. To generate reference sequences for comparison with the Illumina assembly, we assembled these datasets, separately or combined into a single set of 454 reads, using the isoform-aware assembler Newbler 2.5. As expected, combining reads from both 454 datasets significantly improved both individual assemblies, as judged, for example, by the improved average and maximum lengths of the assembled transcripts (N50 = 1.1 kb, longest sequences = 12.2 kb) (Figure S1a-c in Additional file 1) and an improved orthology hit ratio (Figure S1d in Additional file 1). To evaluate assembly quality, we compared our short-read *de novo *assembly with the assembly obtained with the combined 454 datasets ('454').

Transcriptome assemblies based on Illumina or 454 data yielded similar numbers of isogroups (non-overlapping sequences, from hereon referred to as genes) and isotigs (isogroups and their putative splice isoforms, from hereon referred to as transcripts) (Figure 2a), as well as comparable mean sequence lengths (454, 946 bp; Illumina, 1,005 bp). Yet, their length distributions differed, with the Illumina assembly being strongly skewed towards longer sequences, reflected in a high weighted median statistic (N50 = 1.5 kb) and greater maximum transcript length (16.7 kb), and the 454 assembly presenting a more symmetrical distribution with a median of 839 bp (Figure 2b), approximately twice the length of the reported raw reads (Figure S1e in Additional file 1). Both assemblies reached an average length greater than the computational set of predictions made by MAKER (mean, 796 bp; median, 624 bp).

To investigate whether the increase in average sequence length observed in our *de novo *assemblies was likely to reflect improved gene models rather than artifacts due to greedy assembly algorithms, we identified the closest homologs for all genes in the genome of *Schistosoma mansoni*, the evolutionarily closest species with a high-quality gene annotation, and determined the ortholog hit ratios for assembled or predicted sequences (Figure 2c) [19]. Both 454- and Illumina-based assemblies display similar bimodal ratio distributions: one group of genes achieved an ortholog hit ratio close to 1.0 and was therefore likely to contain near full-length sequences. A second peak, at a ratio of approximately 0.15, indicated that a roughly similarly sized group contained genes considerably shorter than their best blast homolog in *S. mansoni*. Most of the computational MAKER predictions fell into the latter group, highlighting the validity of the additional information available through transcriptome sequencing.

As an alternative way of assessing the quality of our assemblies, we compared the 125 full length *S. mediterranea *cDNA sequences available from NCBI's GenBank with their best reciprocal blat hits from each assembly. Most known genes were well represented in each assembly (Figure 2d). For example, the Illumina+ assembly contained near full-length sequences (median of 92.9% cDNA sequence recovered) for 75 (60%) of the 125 known genes.

Next, we mapped the assembled genes onto the approximately 43,000 genomic supercontigs using blat [20]. As no genomic information had been used to construct the transcriptome, an independent convergence of *de novo *assembled and genomic sequences would indicate a high quality of the assembly process. More than two thirds (67%) of all genes assembled from Illumina data could be matched to the genome with alignments including more than 90% of each transcript length. With the same settings, a considerably larger fraction, 79%, of the sequences assembled from 454 data could be matched (Figure 2e). By allowing alignments including only 60% of the gene length, nearly all of the sequences from the 454 assembly (96.1%) and a very large fraction of the Illumina dataset (87%) could be located on a single genomic supercontig.

**Figure 3:**
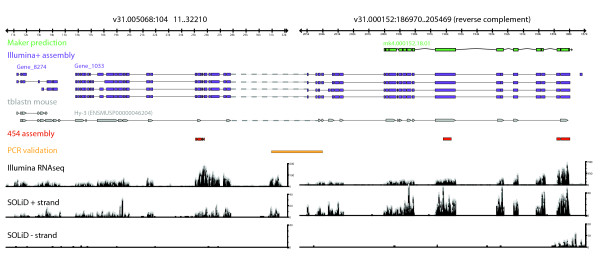
***De novo *assembled transcripts connect genomic supercontigs**. Schematic overview of end regions of two genomic supercontigs, v31.005068 (left) and v31.000152 (right; reverse complemented, showing previous MAKER predictions in green). Shown are *de novo *transcripts from the Illumina+ assembly (purple), a blastn alignment of the *Mus musculus *Hy-3 protein to both supercontigs (grey), transcripts assembled from 454 data alone (red), an experimentally validated PCR amplicon spanning both supercontigs (orange), and transcriptome sequencing coverage summed over all Illumina (non-stranded) or SOLiD samples (strand-specific, coverage of plus and minus strands are shown in separate panels).

The draft genome assembly is highly fragmented and 44% of all supercontigs are shorter than 10 kb (median length of 11.3 kb), putting them into the same size range as the longest sequences in our *de novo *assemblies (Additional file 2). We therefore inspected the partial alignments of long gene loci that could not be aligned to any single supercontig. We identified 1,449 sequences (length > 1,000 bp) with non-overlapping, high-scoring alignments to different supercontigs. Of these, 413 displayed significant homology to proteins in the NCBI non-redundant protein database overlapping with the putative supercontig junctions, lending independent support to the validity of our *de novo *assemblies (Additional file 3; see Materials and methods for details). For example, four transcripts from Gene_1033 up to 13.4 kb long were assembled from Illumina short-read data, while only short fragments of these transcripts could be assembled using the 454 datasets. The transcripts could be aligned to supercontig v31.000152 at their 5' ends (6.2 kb, 47% of the longest transcript), but matched supercontig v31.005068 at their 3' ends (6.1 kb, 45.5% of the longest transcript) with more than 99% identity between cDNA and genome sequence (Figure 3).

The closest mouse ortholog, the *Hy-3 *gene, is homologous to both the 5' and 3' end of the transcripts' sequences and aligns to the same genomic regions, pointing towards the physical continuity of these two supercontigs. We tested this hypothesis experimentally and confirmed it by PCR amplification and Sanger sequencing of a supercontig-spanning sequence (Figure 3; Additional file 4). This exemplifies the potential for *de novo *transcriptome assemblies to aid in refining the *S. mediterranea *genome, similar to a recent case study performed on the *Caenorhabditis elegans *genome [21]. Closer inspection of the alignment of mouse *Hy-3 *also revealed overlap with the adjacent, separate gene identified in our assembly (Figure 3, Gene_8274), which is likely continuous with Gene_1033.

To independently verify the expression of individual genes assembled from Illumina short-read data, we picked 14 sequences for experimental validation of expression and amplicon size by RT-PCR, all of which could be detected at the expected sequence lengths, further demonstrating the accuracy of our *de novo *assembly (Additional file 5).

Based on these combined results, we conclude that our paired-end Illumina transcriptome assembly contained high quality, often near full-length sequences. To improve the assembly further, while maintaining the ability to assemble multiple isoforms for each gene, we repeated the Velvet/Oases assembly of the Illumina reads, this time providing the result of the 454 assembly and EST sequences obtained from GenBank as scaffolds to the algorithms. This allowed us to connect isolated clusters of assembled Illumina reads via bridging with longer sequences, while still requiring a minimum short read coverage of the final sequence. This 'assisted assembly' yielded 24,669 isotigs/transcripts, grouped into 17,465 isogroups/genes and achieved a further increase in average and maximum transcript lengths (maximum length, 17.6 kb; N50, 1.6 kb; Figure 2a). We therefore chose this dataset, from hereon referred to as 'Illumina+', for further characterization and the identification of differentially regulated genes.

**Figure 4:**
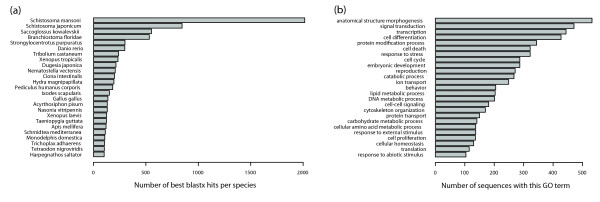
**Preliminary annotation of the *de novo *assembled transcriptome**. **(a) **Top 25 species yielding the top-scoring hit in a blastx comparison of the Illumina+ assembly with NCBI's non-redundant (nr) protein database. For multi-isoform loci, only the longest isoform was considered. **(b) **Top 25 Gene Ontology (GO) 'biological process' annotations of the Illumina+ transcriptome obtained with Blast2GO. For multi-isoform loci, only the longest isoform was considered.

To provide a preliminary annotation of the Illumina+ assembly, we performed a blastx search of the longest transcript from each gene against the NCBI non-redundant (nr) protein database and identified homologous protein sequences for 10,112 out of 17,465 genes (57.9%, e-value cutoff of 10^-3^). Focusing on the highest scoring match for each sequence revealed that the largest number of top hits originated from *S. mansoni *(2,015 hits, 19.9%) or *Schistosoma japonicum *(845 hits, 8.4%) (Figure 4; Additional file 6), trematode parasites from the Platyhelminthes phylum. Next, we used internally consistent Gene Ontology (GO) annotations from the top 20 blast hits to provide a preliminary functional annotation to the *de novo *assembly with the Blast2GO tool [22] and obtained predictions for 6,678 (66.0%) of the genes with significant blast hits. Among the most frequently assigned 'GO biological process' terms were, for example, signal transduction (GO:0007165), response to stress (GO:0006950) and cell differentiation (GO:0030154), indicating that our assembly captured at least part of the regulatory kit of planarian cells (Figure 4b; Additional file 7).

### Dynamic gene expression during head regeneration

To identify genes dynamically regulated in response to tissue loss and during early head regeneration, we mapped the reads from each of the seven Illumina libraries, composed of samples collected from two control samples (0 h) and between 0.5 and 1 h, 2 to 3 h, 4 to 8 h, 10 to 18 h or 24 to 72 h post-amputation, respectively, to the Illumina+ assembly using bowtie [23]. On average, 57.9% of all reads could be mapped uniquely to the assembly, corresponding to a total of between 11.7 and 16.3 million counts for each library (Additional file 8).

**Figure 5:**
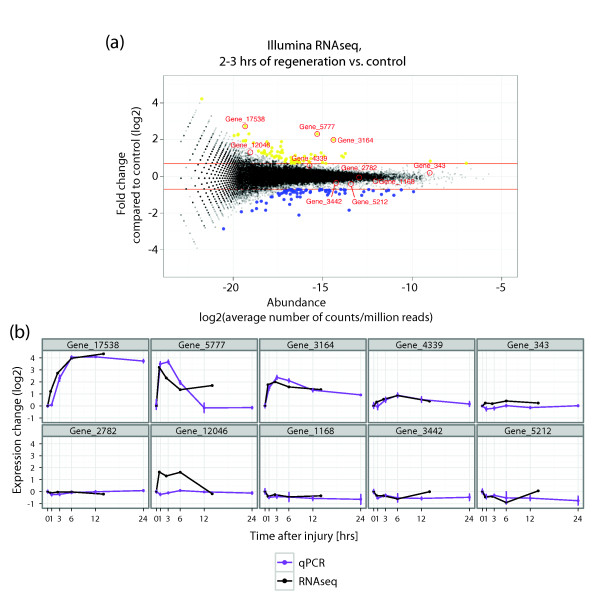
**Differential gene expression during planarian regeneration**. **(a) **For the longest isoform of each locus from the Illumina+ assembly, the expression fold change (log_2 _scale) of the 2 to 3 h sample relative to the control (0 h) is plotted against its log average abundance (MA plot). Statistically significant up- or down-regulation (adjusted *P*-value < 0.001 and log_2 _fold change > 0.7 or < -0.7 (red lines)) is indicated in yellow and blue, respectively. Genes chosen for quantitative RT-PCR (qRT-PCR) validation are labeled. **(b) **Comparison of results from the Illumina transcriptome sequencing (RNAseq) time course with an independent head and tail amputation experiment assayed by qRT-PCR. Expression levels were normalized to that of intact controls (time point 0). RNAseq results are shown at the mid-point of the respective window of time covered by samples in each library. Error bars for qRT-PCR data represent standard errors of the mean of three biological replicates.

**Figure 6:**
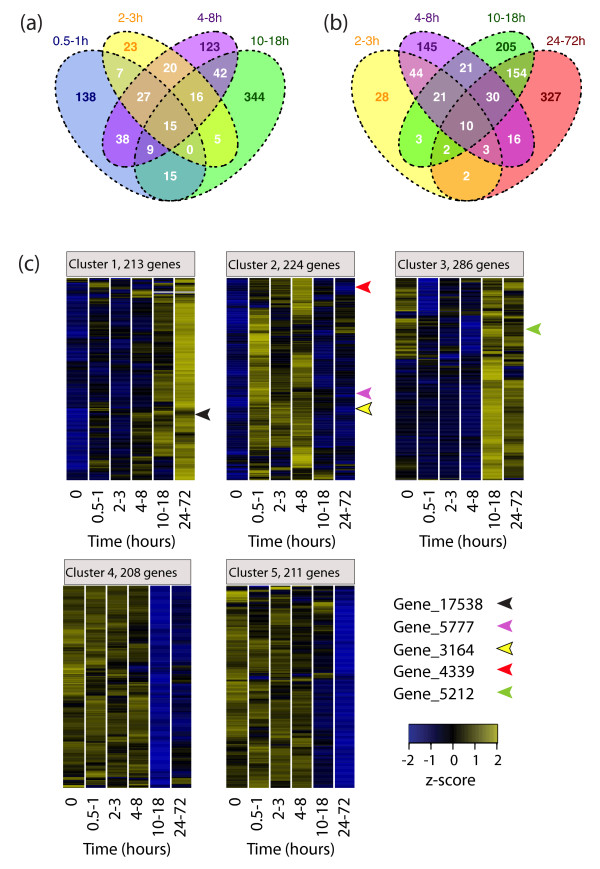
**Classes of dynamically regulated genes during planarian regeneration**. **(a, b) **Venn diagrams depicting the number of statistically significantly regulated genes detected during the Illumina transcriptome sequencing (RNAseq) time course (adjusted *P*-value < 0.001, and log_2 _fold change > 0.7 or < -0.7) at one or more time points. Colors indicate 0.5 to 1 h samples (blue; included only in (a)), 2 to 3 h samples (yellow), 4 to 8 h samples (purple), 10 to 18 h samples (green) and 24 to 72 h samples (red; included only in (b)). **(c) **Heatmap display of the consensus results from 100 k-means clustering runs with z-score transformed expression changes of significantly regulated genes (*P*-value and log_2 _fold change cutoff as in (a)). Rows correspond to genes, columns correspond to Illumina RNAseq time points. The color scale ranges from negative (blue) through neutral (black) to positive z-scores (yellow). Genes discussed in the main text are indicated by arrowheads.

To compare across different samples, we normalized the data to account for differences in the total number of reads per library and identified genes differentially expressed compared to the control samples (time point 0; see Materials and methods section for details; Figure 5a; Additional files 7 and 9) [24,25]. We identified 1,143 significantly regulated loci (adjusted *P*-value < 0.001 and a log_2 _fold change of ± 0.7 at one or more time points), with many genes displaying highly dynamic expression patterns during the recorded time course (Figures 5a and 6a, b; Additional file 9).

To verify the accuracy of our global transcription profiling results, we selected ten genes, both differentially expressed and negative controls, for in-depth validation by quantitative RT-PCR (qRT-PCR; Figure 5b). The analysis of biologically and technically independent time courses covering the first 24 h of regeneration revealed a very high concordance between RNAseq and qRT-PCR. For example, both approaches revealed strong upregulation of Gene_5777 and Gene_3164 as early as 1 h after amputation (Figure 5b; Additional files 7 and 9), with expression levels decreasing again by 6 h. In addition, the induction of Gene_17538 was detected between 3 and 6 h of regeneration, with both methods (Figure 5b; Additional files 7 and 9). These highly dynamic temporal expression profiles were also detected in an independent SOLiD RNAseq experiment recording changes after head and tail amputation (see below). This high reproducibility of expression profiles between independent technologies demonstrates the power of RNAseq for differential expression analysis of biological samples.

We next extended our analysis to the full Illumina time course to study global patterns of gene expression during regeneration. At each time point, unique as well as overlapping sets of differentially expressed genes were detected **(**Figure 6a, b). For example, out of 249 significantly regulated sequences detected at the 0.5 to 1 h time point, 111 (44.6%) were also detected as differentially regulated at one or more of the following time points, lending further support to each individual observation (adjusted *P*-value < 0.001 and a log2 fold change of ± 0.7; Figure 6a). To reveal the underlying regulatory dynamics, we transformed the expression changes of all significant genes to z-scores and applied an unsupervised learning technique, k-means clustering, and identified five distinct temporal classes (Figure 6c). Cluster 1 featured genes that showed early and sustained induction throughout the time course of head regeneration (indicated by a steady transition from negative to positive z-scores). Cluster 2 contained genes that were upregulated rapidly and transiently within the first 8 h of regeneration, when wound healing, immune responses and stem cell proliferation take place. Genes in cluster 3 were upregulated after 10 h of regeneration, when the blastema forms. At this stage, genes grouped into clusters 4 and 5 began to show decreased expression, with strongest down-regulation detected between 10 and 18 or 24 and 72 h, respectively, when cells undergo migration, differentiation, and patterning processes.

Of the total 1,142 differentially expressed genes clustered into the 5 temporal classes, 273 (23.9%) were associated with GO slim annotation terms, allowing us to search for functional categories significantly over- or under-represented among genes with dynamic expression patterns compared to the full set of loci (Additional file 10). Despite the sparsity of the preliminary annotation, we detected a significant overrepresentation of putative serine-type endopeptidases in cluster 5 and their putative inhibitors in cluster 3. In contrast, we found cluster 4 to be specifically enriched in regulators of synaptic transmission and putative membrane transporters.

**Figure 7:**
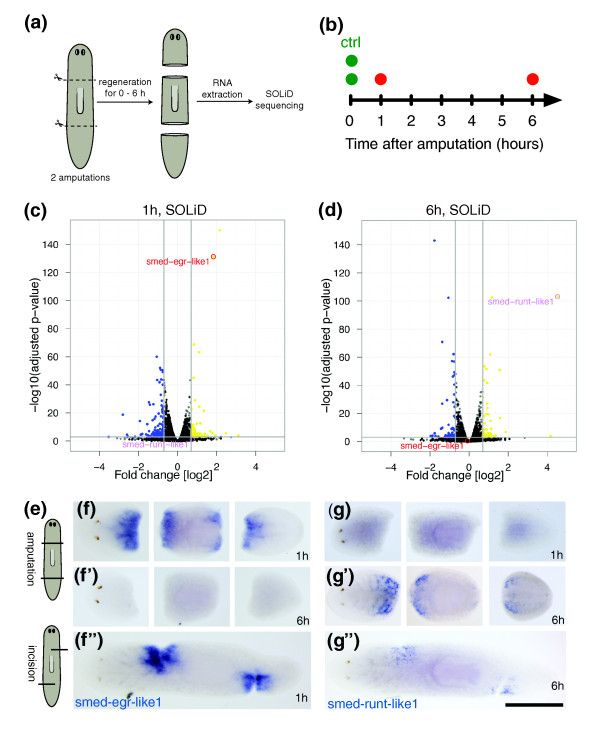
***Smed-egr-like1 *is rapidly induced during regeneration and wound healing**. **(a) **Schematic overview of the whole-worm sample collection approach for SOLiD expression profiling. **(b) **Sample collection timeline. Regenerating and intact control samples are indicated as red and green dots, respectively. **(c, d) **Volcano plots showing the negative decadic logarithm of the adjusted *P*-value and the observed log_2 _fold changes 1 h (c) and 6 h (d) after injury. The expression of *smed-egr-like1 *(red) and *smed-runt-like1 *(purple) are indicated. The horizontal grey line indicates an adjusted *P*-value cutoff of 0.001, vertical grey lines indicate log_2 _fold change threshold of -0.7 and 0.7. Significantly up- and downregulated genes are shown in yellow and blue, respectively. **(e) **Schematic diagram illustrating complete and incomplete cuts, triggering a regeneration or wound healing response, respectively, in the animals. **(f) **Whole mount *in situ *hybridization analysis of *smed-egr-like1 *mRNA in regenerating head, trunk, and tail fragments at 1 h (f) and 6 h (f') as well as a wounded animal at 1 h (f''). Anterior is left. Scale bar is approximately 2 mm. **(g) **Whole mount *in situ *hybridization analysis of *smed-runt-like1 *mRNA in regenerating head, trunk, and tail fragments at 1 h (g) and 6 h (g') as well as a wounded animal at 6 h (g''). Scale and orientation as in (f). *Smed*-*runt-like1 *is strongly induced in cells close to blastemas and around incisions at 6 h of regeneration.

To validate our findings and collect additional data during the early stages of regeneration, we performed a second independent RNAseq experiment aimed at identifying differential gene expression after amputation of both head and tail regions from strand-specific RNAseq libraries using SOLiD technology. We collected samples from planarians regenerating both head and tail regions at 0 h (control), 1 h, and 6 h after amputation, and prepared strand-specific whole transcriptome sequencing libraries from polyA RNA (Figure 7a, b). For each sample, we obtained between 59 and 64 million raw reads using SOLiD V3 chemistry. Of these reads, 46% could be mapped to the Illumina+ transcriptome (Additional file 8), demonstrating that our assembly provided sufficient coverage to serve as a reference for the analysis of data from other experimental samples or sequencing methodologies. Mapping the strand-specific SOLiD data revealed that most genes (15,739 out of 17,459, 90.1%) showed strong strand bias, with more than ten times more reads mapping to the forward than the reverse strand or vice versa. This provided further support for the success of our *de novo *assembly strategy and allowed us to discern the direction of transcription for the majority of loci.

### An EGR-like transcription factor is induced early in response to injury

Gene_5777 is within the group of early up-regulated genes in cluster 2 of the Illumina RNAseq time course (Figure 6c). Its expression was strongly induced within the first hour after decapitation (log2FC > 3), and dropped to low levels between 3 and 6 h, an expression change that was confirmed by SOLiD sequencing (Figure 7c, d). Sequence analysis revealed that Gene_5777 maps to genomic contig v31.019596 and encodes a putative EGR transcription factor, which we called Smed-EGR-like1 (accession number [GenBank: JF914965]). To test where in the animal *smed-egr-like1 *was expressed, we performed whole mount *in situ *hybridization of intact and regenerating animals. We did not detect *smed-egr-like1 *mRNA in either intact animals or in animals after 6 or 24 h of regeneration (Figure 7d-f, and data not shown), but the gene was strongly expressed in broad domains at both anterior and posterior blastemas 1 h after the cut (Figure 7f). To evaluate whether this up-regulation was caused by the loss of tissue (the result of a complete transverse cut through the animal) or in response to wounding alone, we performed a complementary experiment by creating small incisions in otherwise intact animals (schematic shown in Figure 7e). As early as 1 h after injury, *smed-egr-like1 *was strongly induced around the sites of incision, including cells located several cell diameters away from the wound (Figure 7f). These experiments validated the results of our RNAseq analysis and indicated that *smed-egr-like1 *is expressed in response to injury, potentially in response to a signal rapidly spreading from the site of tissue damage.

### A Runx transcription factor is required for proper regeneration of the visual system

**Figure 8:**
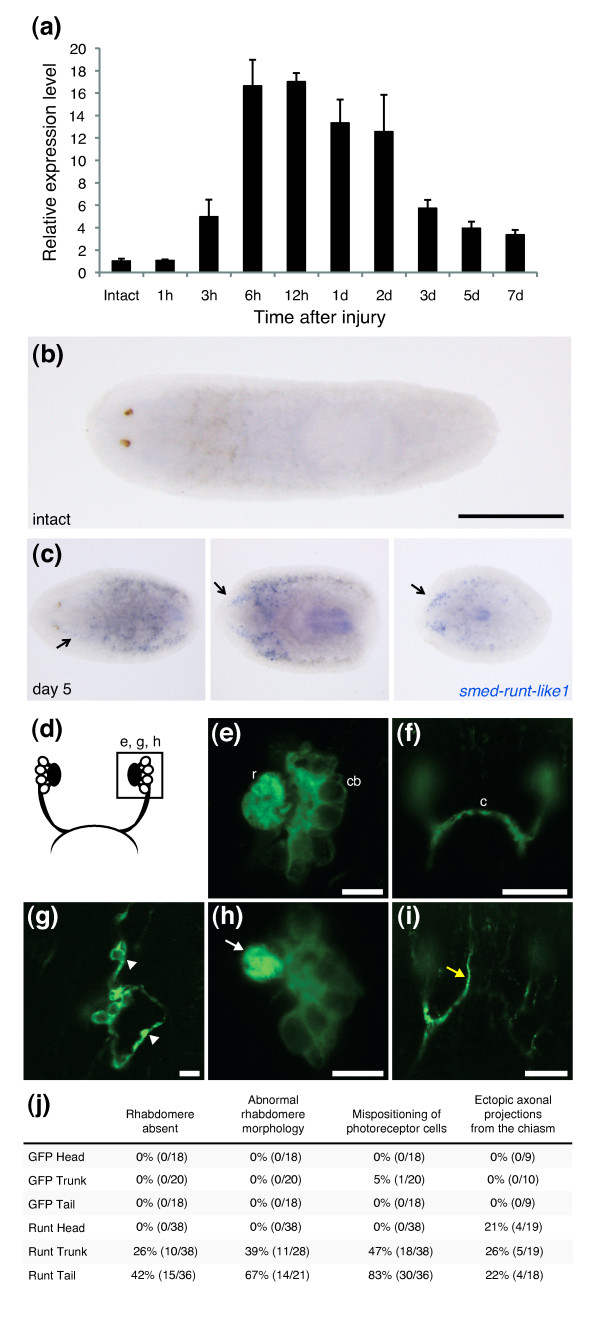
***Smed-runt-like1 *is induced during regeneration and controls patterning of photoreceptor neurons in the eye**. **(a) **qRT-PCR analysis of *smed-runt-like1 *expression in regenerating planarians at different time points of regeneration. Expression levels are normalized to intact levels. Error bars represent standard deviations of the mean of three biological replicates. **(b, c) ***In situ *hybridization analysis of *smed-runt-like1 *mRNA in intact animals (b) and (left to right) regenerating head, trunk, and tail fragments at 5 days after dissection (c). Note that *smed-runt-like1 *is not detected in intact animals, but it is induced in cells in proximity to the regenerating brain. Arrows point to regions of *smed-runt-like1 *expression. Anterior is left. Scale bar is approximately 2 mm. **(d-j) **Effect of *smed-runt-like1 *RNAi on the regenerating visual system. (d) Schematic drawing of the planarian visual system. Bipolar photoreceptor cells cluster to form a rhabdomeric structure on one end, and axonal projections, which reach out to the brain in a contra- or ipsilateral manner, on the other end. (e-i) Immunofluorescence analysis of photoreceptor neurons, 14 days after decapitation, in control (e, f) and *smed-runt-like1 *RNAi (g-i) animals stained with an anti-Arrestin antibody (kindly provided by H Orii). The regions depicted are indicated in (d). Note that RNAi animals show a variety of patterning defects, such as mispositioning of photoreceptor cells (g), abnormal rhabdomere morphology (h), and axon guidance defects (i). Arrowheads point to mispositioned cells, white arrow to a malformed rhabdomere, yellow arrow to a misrouted axon. c, optic chiasm; cb, cell bodies; r, rhabdomere. Scale bars = 10 mm in (e, g, h), 50 mm in (e, i). (j) Summary of *smed-runt-like1 *RNAi phenotypes. The number of animals scored is indicated in parentheses.

In contrast to *smed-egr-like1*, the amputation-induced expression of Gene_17538 was sustained for at least 3 days (Figure 6c, cluster 1). Consistent with our SOLiD and Illumina RNAseq data, the Gene_17538 transcript was undetectable in non-regenerating animals or within the first hour after injury (Figures 7g and 8b). However, at 6 h after decapitation, Gene_17538 mRNA was detected in laterally enriched, discrete cells in close proximity to the cut (Figure 7g). Gene_17538 expression was stronger in the more anterior tissue of the same fragment, indicating potential sensitivity of its expression to an anterior-to-posterior gradient.

To determine how long this gene was expressed during regeneration, we repeated and extended the time course using qRT-PCR. Gene_17538 was upregulated more than 5-fold as early as 2 to 3 h after decapitation, reached maximum induction (more than 16-fold) after 6 h, and maintained a high expression level during the first 48 h of regeneration, before slowly returning to near basal levels after 1 week (Figure 8a). Sequence analysis revealed that Gene_17538 maps to supercontig v31.001002 in the planarian genome and encodes a putative Runx transcription factor. Its predicted protein product shares 49% sequence identity with the *Drosophila *Lozenge protein in the conserved Runt domain (Additional file 11). We named Gene_17538 *smed-runt-like1 *(accession number [GenBank: JF720854]), as we found at least one more putative Runx transcription factor (Gene_20170), which mapped to genomic contigs v31.007764 and v31.028733 but did not seem differentially expressed at the time points tested (Additional file 7).

After 5 days, *smed-runt-like1*-positive cells could no longer be detected in posterior-facing blastemas. Instead, *smed-runt-like1 *was expressed in a subset of cells along the regenerating brain (Figure 8c) at the time when new photoreceptor neurons connect to the brain and the animal recovers vision [9]. Interestingly, after 5 days, *smed-runt-like1*-positive cells appeared also in trunk-regenerating head pieces, indicating a putative role in general tissue remodeling and re-scaling of the head during trunk-regeneration (Figure 8c).

To test for a functional role for *smed-runt-like1 *during head regeneration, we inhibited its expression through RNAi by injecting long double-stranded RNAs (dsRNAs) into the gut of planarians. mRNA levels of *smed-runt-like1 *were strongly reduced, but not completely eliminated after RNAi, possibly due to very rapid kinetics of its induction after decapitation (Additional file 11). However, using two independent dsRNAs, we observed reproducible defects in eye regeneration, with phenotypes including abnormal rhabdomere morphology, mispositioned photoreceptor neurons and misrouted axons (Figures 8g-j; Additional file 11). These phenotypes are consistent with the role of *lozenge *during fly larval development, where this gene is expressed in photoreceptor precursor cells [26] to control cell fate decisions and patterning of the visual system through transcriptional regulation of other transcription factors, such as the homeodomain-containing protein Prospero [27]. Whether *smed-runt-like1 *controls the development of eyes during planarian regeneration through similar mechanisms and target genes as in the fly remains the subject of future studies.

Consistent with the *smed-runt-like1 *expression pattern, axon guidance phenotypes were also observed in 21% of all trunk-regenerating head fragments (Figure 8j). This suggests that the developmental programs controlling regeneration might also be active during tissue remodeling and body re-scaling.

## Discussion

### Illumina versus 454 sequencing for transcript discovery

*De novo *transcriptome assembly from next generation sequencing data offers the opportunity to perform systematic, unbiased analysis of gene expression and its regulation. To date, transcribed sequences from *S. mediterranea *were mainly identified through analysis of EST clones [28] or 454 sequencing [17,18]. Additionally, differential gene expression studies of planarian gene expression were performed with cDNA microarrays, featuring up to 1,640 sequences [29,30].

In this study, we used short paired-end transcriptome reads from different stages of planarian head regeneration to both assemble a high-quality transcriptome dataset and observe quantitative, dynamic changes of gene expression. A rigorous comparison to publically available data, both computational predictions and 454 datasets, revealed that this strategy is suitable for the reconstruction of long transcripts, exceeding the maximum sequence length obtained by combining all publically available 454 sequence reads.

As opposed to earlier transcriptome assembly approaches [17,18], the assemblies presented in this study unambiguously identify different splice isoforms originating from the same locus, a prerequisite, for example, for the accurate summary of unique reads mapping to each locus or meaningful annotation statistics, including GO term frequency.

Reconstructing transcripts completely *de novo*, without the use of genomic scaffolds, allowed us to overcome several of the limitations posed by the fragmented *S. mediterranea *genome assembly. The unique alignment of short reads to the genome is hampered by its high A/T content (greater than 65%), its high repetitiveness, and the redundant representation of genomic locations on multiple supercontigs. The assembly of short reads into longer transcripts simplifies the alignment and offers novel opportunities to improve the genomic assembly by identifying supercontig-spanning genes (Figure 3; Additional file 3). Conversely, the fact that the vast majority of assembled sequences could be aligned to one (or more) genomic supercontigs validated the quality of our *de novo *strategy and highlights its usefulness as an independent source of information for a future consolidated annotation of the *S. mediterranea *genome.

The analysis of whole transcriptome libraries, generated without any normalization of transcript levels, allowed us to use the same datasets to compare quantitative changes in gene expression between samples. While earlier studies compared, for example, irradiated animals with controls [18,30] or different tissues [29], or used RNAseq to discover small RNAs [31], we performed a time-course of head-regeneration to study the dynamics of differential gene expression, presenting a first glimpse of the temporal coordination of wound healing and tissue regeneration in planarians.

### The head regeneration transcriptome

The *S. mediterranea *head regeneration transcriptome contains genes that are expressed in stem cells and somatic cells of the pre-pharyngeal region posterior to the eyes, and genes that are upregulated during the first three days of head regeneration. The tissues used for the preparation of the sequencing libraries contained parts of the brain and ventral nerve cords. As stem cells can be found nearly everywhere in the planarian body and the anterior branch of the planarian gut extends into the head region, stem cells and gut epithelium were also included in our samples. As a consequence, we found stem cell-specific genes, such as the *piwi *genes *smedwi-1 *and *-2 *(Gene_5983 and Gene_11) [32], as well as genes expressed in the digestive system, such as a *porcupine*-like gene (Gene_1606) [33]. We also detected a large number of putative and known brain- and/or nervous system-specific sequences. Among those were commonly used pan-neuronal markers, such as a *prohormone convertase 2 *(*PC2*; Gene_785) [34], and approximately 30 peptide prohormone-encoding genes, including several members of the *secreted peptide prohormone *(*spp*) and *neuropeptide Y prohormone *(*npy*) families [3]. We also identified markers for specific subsets of neurons that have been used in the planarian *Dugesia japonica *(*dj*), such as putative homologues of the *tryptophan hydroxylase *gene *djTPH *(Gene_15207), a component of serotonergic neurons [35], and the *tyrosine hydroxylase *gene *djTH *(Gene_14331), an enzyme-coding gene expressed in dopaminergic neurons [36]. Moreover, we detected a number of genes reported to be involved in axon guidance mechanisms during head regeneration, such as *netrin 1 *(Gene_5462) and its putative receptor (*netR*; Gene_2511) [37]. In addition to brain and stem cell-specific genes, we identified anterior homeobox genes, such as putative *djotxA *and *B *homologues (Gene_14050 and Gene_13168) [4], and the TALE class homeobox gene *smed-prep *(Gene_7075) [38]. These findings indicate that our head regeneration transcriptome contains a broad range of gene families with diverse functions that are expressed in the anterior region of the body during the first three days of head regeneration.

We also wanted to test whether our transcriptome set excluded posterior genes that are not expressed in the anterior region of the animal during homeostasis or regeneration. *Smed-abdBa*, for instance, is an AbdominalB-like homeobox protein expressed exclusively in the tail, and is slightly upregulated in the posterior blastema during regeneration [39]. Consistent with this, we could not assemble the *smed-abdBa *transcript from Illumina reads obtained from pre-pharyngeal samples, but detected expression of the corresponding MAKER transcript *mk4.003188.01 *in our SOLiD whole-animal dataset (Additional file 12). Similarly, we did not assemble any transcript corresponding to *smed-wnt1*, a Wnt family member expressed in a few cells along the posterior midline in intact animals [40,41]. This gene was previously shown to be mildly upregulated in a few cells in anterior blastemas between 12 and 24 hours of regeneration [41], demonstrating the limitations of our approach for very lowly expressed genes.

### Differentially regulated genes during head regeneration

Using our Illumina RNAseq approach, we identified hundreds of differentially regulated genes during different stages of head regeneration, and clustered them into groups according to their dynamic expression profiles (Figure 6c).

Among the genes that were strongly upregulated during the first few hours of regeneration were genes encoding putative homologues of mammalian inflammatory response genes. Gene_3164, for instance, encodes a putative tumor necrosis factor receptor (TNFR)-associated protein (TRAF). TRAFs interact with numerous members of the TNFR family and have a variety of tissue-specific functions. As TRAFs have been implicated in promoting the immune response after infections and wounding [42,43], it is possible that similar proteins are also involved in the regulation of immune responses after decapitation in planarians. Reported RNAi phenotypes of putative TRAF-like genes in the planarian species *D. japonica *are pleiotropic [44]. Thus, the functions of putative planarian TRAFs, as well as the different cell types and molecules that determine the nature of the planarian immune system, remain largely unknown.

Another gene from the same cluster, *smed-egr-like1*, was strongly upregulated between 30 minutes and 1 hour after wounding (Figures 5, 6, and 7), making it one of the earliest wound-induced planarian genes known to date. This gene encodes a putative zinc finger protein similar to members of the EGR gene family. EGRs are central regulators of early immune and inflammatory responses [45], and are rapidly activated following stress [46,47] through different stimulants, including growth factors [48], calcium [49] and oxygen reactive species [50]. In addition to a putative role in the activation of immune cells, planarian EGRs might be involved in the regulation of extracellular matrix remodeling during wound healing, as mammalian EGR-1 has been implicated in transcriptional regulation of collagens [51] and matrix metalloproteinases [52].

The putative Runx transcription factor gene *smed-runt-like1 *(Figure 6c, cluster 1) was also upregulated early, but maintained a high expression throughout the time course of regeneration. *Smed-runt-like1 *was expressed close to the wound within a few hours following amputation or injury (Figure 7), and could later be detected in the regenerating brain (Figure 8). This expression pattern would be consistent with two different roles during regeneration. At early stages, *smed-runt-like1 *might be involved in the response to injury. At later stages, when newly formed photoreceptor neurons connect to the brain, *smed-runt-like1 *might act in a process guiding photoreceptor neurons and their axons to their targets, as suggested by the axon guidance and cell mispositioning phenotypes in *smed-runt-like1 *RNAi animals (Figure 8g-j). These phenotypes are consistent with the role of the *Drosophila lozenge *gene in the developing fly eye [26]. The absence of an early regeneration phenotype might be due to the incomplete knockdown of *smed-runt-like1*, to which eye regeneration processes might be more sensitive. Another reason might be redundant expression of one or more additional regulators at early time points but not during eye patterning. Finally, it is possible that the transcriptional program regulating eye patterning might already start in the early phase of regeneration and thus require *smed-runt-like1 *transcriptional activation within the first 6 hours.

Stem cell migration to the wound takes place during the first 2 days of head regeneration, and an accumulation of stem cells is observed within 18 hours after decapitation [7]. Thus, we expected regulators of stem cell migration to be grouped in clusters 3 and 4. Indeed, we identified several genes encoding putative extracellular proteins, for example, the putative matrix metalloproteinase Gene_3022. In addition, GO analysis showed that putative inhibitors of serine-type endopeptidases were significantly enriched in cluster 3. Accordingly, in the cluster of genes, whose expression levels were strongly reduced after 24 hours of regeneration (cluster 5), we found enrichment of putative serine-type endopeptidases, indicating that extracellular matrix remodeling is important for regeneration during the time when stem cell migration takes place.

In cluster 4, we also found significant enrichment of putative regulators of synaptic transmission and membrane transporters. This supports a model in which nerve signaling might be important for early stages of planarian regeneration. Recently, it has been shown that ventral nerve cords can signal to regulate regeneration polarity in planarians [53], and nerve signaling promotes limb regeneration in axolotl [44,54].

In summary, our time course analysis indicates that a complex, dynamic transcriptional network, rapidly induced after wounding, controls head regeneration. This network might be triggered by initiation factors that launch a cascade of gene regulatory events that promote all important regenerative processes, such as wound healing, stem cell proliferation and migration, as well as differentiation and organ patterning.

## Conclusions

In this study we constructed a planarian head-regeneration transcriptome and generated time-resolved profiles of genes expressed during this process. Our approach allowed the use of the same dataset to efficiently assemble a transcriptome and to detect differential expression between samples. We highlighted differentially regulated genes, as well as clusters of genes with similar gene expression dynamics as a starting point for further investigations. Finally, we demonstrated that *smed-runt-like1 *is required for eye regeneration as a proof of principle that our approach allows for the identification of genes that are functionally relevant during planarian head regeneration.

## Materials and methods

### Planarians

All animals used in this study were asexual planarians of the species *S. mediterranea *(clonal line BCN-10) provided by E Saló.

### Library construction and Illumina paired-end sequencing

Planarians were starved for 1 week prior to decapitation with a razor blade. For the preparation of head regeneration sequencing libraries for Illumina paired-end sequencing, regeneration was allowed to occur for 16 different periods of time (30 minutes, and 1, 2, 3, 4, 5, 6, 8, 10, 12, 16, 18, 24, 36, 48, and 72 h). A second cut anterior to the pharynx (pre-pharyngeal cut, see Figure 1a, b) was performed to separate the anterior regenerating part from the rest of the body. Eight planarian pre-pharyngeal pieces from similar time points were pooled (library 1: 30 minutes and 1 h, four animals each; library 2: 2 h and 3 h, four animals each; library 3: 4, 5, 6, and 8 h, two animals each; library 4: 10, 12, 16, and 18 h, 2 animals each; library 5: 24, 36, 48, and 72 h, two animals each), and immediately deep-frozen. Sixteen non-regenerating pieces (0-h time point) served as two independent biological replicates (8 animals each; libraries 6 and 7). RNA was processed in two batches: material from library 6 was processed together with libraries 1 to 3, while library 7 was processed together with libraries 4 and 5.

Seven Illumina RNAseq libraries were prepared according to the mRNA-Seq Sample Preparation Kit (Illumina, part number 1004898 Rev.D). In brief, 0.5 mg of polyA RNA was isolated using the MicroPoly(A)Purist kit (Ambion, Austin, Texas, USA), fragmented and precipitated. First strand cDNA was synthesized using SuperScript II (Invitrogen, Carlsbad, California, USA) and the second strand was added using RNAseH and DNA Polymerase I. cDNA was purified with the QIAquick PCR purification kit (Qiagen, Hilden, Germany) and ends were repaired with Klenow DNA polymerase. The 3' ends were adenylated with Klenow exo (3' to 5' exo minus) enzyme and ligated to the sequencing adapters. cDNA templates were separated by Agarose gel electrophoresis and approximately 200-bp sequences were selected. Templates for sequencing were PCR amplified using Phusion DNA polymerase (Finnzymes Oy, Vantaa, Finland) and purified with the QIAquick PCR purification kit (Qiagen, Hilden, Germany). We performed 2 × 36-bp paired-end sequencing on a Genome Analyzer II instrument according to the manufacturer's instructions.

### Library construction and SOLiD sequencing

For the preparation of libraries for SOLiD strand-specific sequencing, five planarian trunks per sample were dissected from heads and tails and all pieces were allowed to regenerate for 1 or 6 h, prior to deep-freezing and RNA isolation (Figure 7a, b). Ten non-regenerating worms (0-h time point) served as two independent biological replicates (five animals each) and were processed in the same way. Total RNA was extracted using TRIzol (Invitrogen). Strand-specific sequencing libraries were prepared from polyA RNA selected material using Applied Biosystems 'Whole Transcriptome Library Preparation protocol' (part number 4409491 Rev. C) (Applied Biosystems, Carlsbad, California, USA). In brief, 1 mg of polyA RNA was isolated using the MicroPoly(A)Purist kit (Ambion), fragmented using RNAse III (Applied Biosystems) and column purified. SOLiD sequencing adapters were ligated to the RNA using components from the SOLiD Small RNA Expression Kit (Applied Biosystems). Afterwards, the RNA was reverse transcribed using ArrayScript Reverse Transcriptase and cDNA purified using the MinElute PCR purification kit (Qiagen). cDNA fragments were separated by electropheresis on Novex 6% TBE-urea gels, stained with SYBR Gold nucleic acid gel stain, and sequences between 100 and 200 nucleotides were selected. Afterwards, fragments were PCR amplified using AmpliTaq for 15 cycles to obtain templates for emulsion PCR and sequencing. Emulsion PCR and sequencing were performed according to the manufacturer's instructions and each sample was sequenced in one quadrant of a flow cell using SOLiD Version 3 chemistry.

### *De novo *assembly

Paired-end Illumina reads were filtered for low-base calling quality using a perl script (Additional file 13), retaining only reads with at least 20 nucleotides (-m 20) exceeding a quality threshold > 10 (-q 10), calculated as the average base quality over a moving window of length 5 (-w 2). The assembly was performed using velvet (ver. 1.0.18) [15] and oases (ver. 0.1.18) Separate runs were performed at different kmer lengths (19 to 31 bp) and the results were introduced as 'long reads' to a final Velvet/Oases assembly (kmer length 31 bp) to yield the initial Illumina assembly.

Previously published 454 sequencing reads [17,18] were obtained from NCBI's short read archive. 454 assembly was performed with Roche's Newbler 2.5 software using default settings for cDNA datasets (Roche Applied Science, Mannheim, Germany). We obtained 69,699 EST Sanger reads from *S. mediterranea *from NCBI's Unigene database, which were combined with the results of the 454 assembly, and served as additional 'long read' scaffolds to Velvet/Oases, yielding the final Illumina+ assembly. A minimum length cutoff of 200 bp was applied to all assemblies. All sequences from the Illumina+ assembly are provided in Additional file 14.

### Sequence analysis and annotation

For comparison with previously identified transcripts, we obtained all *S. mediterranea *cDNA sequences from NCBI's GenBank, excluded EST sequences, as they had been used for the Illumina+ assembly, and retained all 125 cDNAs annotated as containing complete coding sequences. To identify the best reciprocal blat hits between this set of sequences and the different assemblies, a blat score cutoff of 50 was applied. To calculate ortholog hit ratios, we compared the longest isoforms of all assemblies with protein sequences from *S. mansoni *(ver. 4.0) [55] using blastx (e-value cutoff 10e-3) [19,56].

Transcripts spanning multiple genomic supercontigs were identified in a multi-step, semi-automated procedure. First, only transcripts with a minimum length of 1 kb were considered. For gene models with multiple isoforms, the longest isoform was evaluated. Second, each transcript was split into non-overlapping 0.5-kb subsequences, which were aligned to the genomic supercontigs individually using blat. For each subsequence, the best mapping result was retained, requiring at least 450 out of 500 (90%) perfectly matched bases; 1,449 transcripts, whose subsequences mapped to at least two different supercontigs, were retained as putative supercontig-spanning sequences. Third, the 1,449 full-length candidate transcripts were re-mapped to the genomic supercontigs using blastn (e-value cutoff 10E-40) and to the NCBI non-redundant protein database (e-value cutoff 10E-3). Schematic representations of the blastn/blastx alignments were inspected manually. Transcripts were considered to be supercontig-joining only, when one or more junctions between genomic fragments were independently supported by overlapping, continuous homology to a known protein. Based on these criteria, 413 high-confidence supercontig-joining transcripts were identified (Additional file 3)

To associate sequences with descriptions and GO terms, we aligned the longest isoform of each assembled locus to NCBI's non-redundant protein datase (nr) using blastx (e-value cutoff 10e-3) and selected up to 20 homologous sequences as input for annotation with Blast2GO [22] using default settings.

### Differential expression analysis

Illumina and SOLiD reads were mapped to a sequence database containing the longest isoform of each Illumina+ sequence using bowtie [23] or Bioscope v1.3 (Applied Biosystems), respectively. Paired-end Illumina reads were mapped to the transcriptome using a three step procedure. First, we mapped both forward and reverse reads together (as pairs). We accepted only uniquely aligned pairs, where both reads mapped in the correct orientation to the same transcript. Read pairs not mapped at this step were split into forward and reverse reads and aligned separately. Second, we mapped the forward read, accepting only unique matches. Third, for those read pairs mapped in neither step 1 nor step 2, we mapped the reverse read, again accepting only unique matches.

Only unique bowtie (options '-m 1 --best -strata') and high-quality Bioscope alignments (mapping quality > 20) were considered. Only SOLiD reads originating from the transcribed strand, determined as the strand accumulating the largest number of mapped reads, were included in the analysis. To map reads to MAKER predictions, Illumina and SOLiD data were mapped to genomic supercontigs with Tophat [14] or Bioscope v1.3, respectively, and unique counts were assigned to each locus using the HTSeq Python package [57]. Read coverage was visualized using Gbrowse2 [58].

Counts were normalized by calculating the median ratio of each sample to the geometric mean of all samples as implemented in the R/Bioconductor edgeR package (scaling method: RLE) [25]. Significant differential expression from SOLiD data was identified using a negative binomial distribution with common dispersion estimated through the quantile-adjusted conditional maximum likelihood (qCML) method [59] from the two control samples. Illumina samples were collected in two experimental batches (batch 1: libraries 1 to 3 and control library 6; batch 2: libraries 4 and 5 and control library 7), which were analyzed separately. To identify differentially regulated genes, each regenerating sample was compared to its corresponding control sample using a Poisson distribution. All gene expression changes were calculated relative to the expression of the respective gene in the controls (time point 0 h). We only considered genes as significantly regulated that passed both of the following two criteria simultaneously at one or more time points: a statistical test (adjusted *P*-value < 0.001) and a log_2 _fold change > 0.7 or < -0.7.

Clusters displaying similar temporal changes were identified among genes showing significant differential expression at one or more time points by k-means support (KMS) clustering using a Eucledian distance measure, as implemented in the MeV analysis tool [60]. Expression changes were converted to z-scores and 100 iterations of k-means clustering performed. All differentially expressed genes were reproducibly grouped together into the same five classes in at least 80 out of 100 (80%) clustering runs.

### RNA interference

dsRNAs were synthesized as described [61]. Three pulses of 32.2 nl of a solution of 1.5 μg/μl *smed-runt-like1 *or *gfp *dsRNA were injected ventrally into the gut of planarians every day for two rounds of three consecutive days using a Drummond Scientific Nanoject injector (Broomall, PA, USA). All animals were cut 1 day after the last injection and fixed, after 14 days of regeneration, for whole-mount immunostaining.

### Whole-mount immunostaining

Planarians were treated with ice-cold 2% HCl in Holtfreter's solution for 3 minutes at room temperature and were then fixed in ice-cold Carnoy (60% ethanol, 30% chloroform, 10% acetic acid) for 2 h at 4°C. The animals were kept in ice-cold 100% methanol for 1 h at -20°C, bleached overnight in 6% H_2_O_2 _in methanol, and were then rehydrated through a gradient series of methanol/phosphate-buffered saline with Tween 20 (PBST) washes (75%, 50%, 25%) for 10 minutes each at room temperature. Following two 10-minute PBST washes, animals were blocked in 1% bovine serum albumin in PBST for 2 h at room temperature. Anti-Arrestin [62] was used at a 1:2,000 dilution at 4°C for 18 h. Eight washes of 1 h each with PBST at room temperature were carried out before blocking with 1% bovine serum albumin in PBST for 1 h at room temperature. Alexa Fluor 488 goat anti-mouse IgG secondary antibody (Life Technologies, Carlsbad, California, USA) was used at 1:400. Planarians were washed 6 × 1 h with PBST and then post-fixed for 1 h in 4% paraformaldehyde/PBST at room temperature. Animals were mounted in Vectashield (Vector Laboratories, Burlingame, California, USA) and observed through a Zeiss LSM510 Meta confocal microscope.

### Whole mount *in situ *hybridization

Animals that had been starved for 1 week were cut completely or incompletely by small incision as schematically described in Figure 7b, then left to regenerate or heal for the indicated time periods. Whole-mount *in situ *hybridization was carried out as previously described [4,63]. Primers used to synthesize DIG-labeled riboprobes were 5'-taatacgactcactatagggGTAATCCGTCGATGAATAGCG-3' and 5'-atttaggtgacactatagCTTTGATGGGATTGCGAGTT-3' (*smed-runt-like1*), and 5'-taatacgactcactatagggACAGGATATACTCCGTTGCCA-3' and 5'-atttaggtgacactatagATTCGAAATTGACTTTTCTCGC-3' (*smed-egr-like1*). Samples were observed through a Leica M165 FC stereomicroscope.

### qRT-PCR

Total RNA was extracted using the TRIzol reagent (Invitrogen), and cDNA synthesized using the SuperScriptIII First-Strand Synthesis Kit (Invitrogen). qRT-PCR reactions were performed using the TaqMan Universal PCR Master Mix (Applied Biosystems) and transcript levels were determined using the Real-Time PCR System 7500 (Applied Biosystems). Three biological replicates were measured in triplicate and reactions lacking reverse transcriptase served as negative controls. Clone H.55.12e (accession number AY068123) [32] was used as an internal reference gene. Primers are listed in Additional file 15.

### RT-PCR

Total RNA was extracted and cDNA was synthesized as described above. Primers listed in Additional file 5b and the following PCR conditions were used: 1 minute at 94°C, 13 cycles of touch-down PCR (30 s at 94°C, 40 s at the annealing temperature (68 to 56°C, decreasing in increments of 1°C/cycle) and 1 minute at 72°C), 22 cycles at a constant annealing temperature (30 s at 94°C, 40 s at 55°C and 1 minute at 72°C), and 10 minutes extension at 72°C.

### Accession numbers

All next generation sequencing data are available through the ArrayExpress repository at the European Bioinformatics Institute (accession number [E-MTAB-607]). The GenBank accession numbers for *smed-runt-like1 *and *smed*-*egr-like1 *are [GenBank:JF720854] and [GenBank:JF914965], respectively.

## Abbreviations

bp: base pair; CNS: central nervous system; *dj*: *Dugesia japonica*; dsRNA: double-stranded RNA; EGR: early growth response gene family; EST: expressed sequence tag; GO: gene ontology; nr: non-redundant; PBST: phosphate-buffered saline with Tween 20; PCR: polymerase chain reaction; qRT-PCR: quantitative reverse transcriptase PCR; RNAi: RNA interference; TNFR: tumor necrosis factor receptor; TRAF: tumor necrosis factor receptor associated protein.

## Authors' contributions

KB designed the study and wrote the manuscript. MB designed the study. TS designed the study, performed SOLiD sequencing and analyzed the next generation sequencing data and wrote the manuscript. MCV performed the experiments and wrote the manuscript. SO performed the experiments and wrote the manuscript. All authors have read and approved the manuscript for publication.

## Supplementary Material

Additional file 1 **Transcriptome assembly from public 454 data**. **(a-c) **Schematic overview of 454 transcriptome assembly approaches. Publicly available 454 reads were assembled either separately (a, b) or as a combined dataset (c) using the Newbler 2.5 assembler. Colors indicate the three different assemblies: yellow, Abril *et al. *[17] (a); orange Blythe *et al. *[18] (b); red, combined (c). Quality metrics shown include the shortest and longest sequences in each assembly, as well as N50, for which 50% of all bases are contained in sequences at least as long as N50. **(d) **Kernel densities of the length distributions for the assembled sequences. For multi-isoform loci, only the longest isoform was considered. Colors as in (a-c). **(e) **Kernel densities of ortholog hit ratios obtained by comparing sequences from the different assemblies or computational prediction to the *Schistosoma mansoni *proteome using blastx. For multi-isoform loci, only the longest isoform was considered. Colors as in (a-c). Click here for file

Additional file 2 **Genomic supercontigs**. This histogram shows the sequence length distribution of all genomic *S. mediterranea *supercontigs (version 3.1 [13]). The red line indicates a sequence length of 10 kb. Click here for file

Additional file 3 **Identification of supercontig-spanning transcripts**. Illustration of 413 high-confidence supercontig-joining transcripts identified, with a description of the procedure. Click here for file

Additional file 4 **Experimental validation of the continuity of genomic supercontigs v31.005068 and v31.000152**. **(a) **Agarose gel electrophoresis showing the PCR amplicon produced with forward and reverse primers annealing to supercontigs v31.005068 and v31.000152, respectively. **(b) **The amplicon sequence was verified by Sanger sequencing. **(c) **Blastn results showing the 5' end of the sequence aligning to supercontig v31.000152 and the 3' end matching supercontig v31.005068. Click here for file

Additional file 5 **Experimental validation of detected transcripts by RT-PCR**. **(a) **Agarose gel electrophoresis of amplicons amplified using primers designed against 14 different sequences from the Illumina+ assembly and two control sequences. **(b) **Primer sequences used in the PCRs. Click here for file

Additional file 6 **Blastx comparison of *de novo *transcriptome sequences with the NCBI non-redundant protein database**. **(a) **Distribution of similarity detected in the best blastx hit for each transcript (blastx e-value < 10^-3^). **(b) **Distribution of blastx e-values for the best blastx hit for each transcript. For multi-isoform loci, only the longest isoform was used as a blastx query. Click here for file

Additional file 7 **Illumina+ assembly annotations and expression data**. A spreadsheet containing the length, annotation and differential gene expression data for all sequences of the Illumina+ assembly. Click here for file

Additional file 8 **Mapping statistics of Illumina and SOLiD raw reads**. Statistics of mapping raw Illumina and SOLiD reads onto the Illumina+ assembly. Click here for file

Additional file 9 **MA plots illustrating differential gene expression from both Illumina and SOLiD data**. For the longest isoform of each locus from the Illumina+ assembly, the expression fold change (log_2 _scale) relative to the control (0 h) is plotted against its log average abundance (MA plot). Statistically significant up- or down-regulation (adjusted *P*-value < 0.001 and log_2 _fold change > 0.7 or < -0.7 (red lines)) is indicated in yellow and blue, respectively. Genes chosen for qRT-PCR validation (Figure 5) are labeled. **(a-e) **MA plots show a comparison of Illumina transcriptome sequencing (RNAseq) data from 0.5 to 1 h samples (a), 2 to 3 h samples (b), 4 to 8 h samples (c), 10 to 18 h samples (d) and 24 to 72 h (e) relative to controls. **(f, g) **SOLiD reads from control or regeneration samples were aligned to the genomic supercontigs with Bioscope. MA plots show a comparison of SOLiD RNAseq data from 1 h sample (f) and 6 h sample (g) relative to controls. Click here for file

Additional file 10 **Gene ontology enrichment analysis for each temporal expression class**. Gene ontology term enrichment and depletion of each cluster compared to the full set of annotated sequences using GOSeq. Click here for file

Additional file 11 **Conservation, knockdown efficiency, and RNAi phenotype of *smed-runt-like1***. **(a) **Alignment (ClustalX) of Runt domains of proteins from vertebrate and invertebrate species: *Danio rerio *(Runt-related transcription factor 3, NP_571679); *Homo sapiens *(Runt-related transcription factor 2, EAX04279); *Hydra magnipapillata *(Runx, XP_002165633); *Drosophila melanogaster *(Lozenge, NP_001096919); *Schmidtea mediterranea *(Smed-Runt-like1, JF720854). The planarian Runt domain shares at least 45% sequence identity with Runt domains from other organisms. **(b) **qRT-PCR analysis of *smed-runt-like1 *expression levels in intact and regenerating planarians 6 h after head and tail dissection. Prior to dissection, animals had been injected with three pulses of 32.2 nl of a 1.5 μg/μl dsRNA solution containing either dsRNAs against *smed-runt-like1 *or a control gene (*gfp*), for three days in a row for two consecutive weeks (day 1 to 3, 8 to 10; cut on day 11). Expression levels were normalized against those of a housekeeping gene (AY068123). Error bars represent standard deviations of the mean of three independent biological replicates of five worms each. Note that the *smed-runt-like1 *mRNA levels are reduced but not completely abolished upon RNAi in regenerating animals. **(c) **Immunofluorescence analysis (anti-Arrestin) of photoreceptor neurons in regenerating control and *smed-runt-like1 *RNAi animals at day 14 after decapitation. Note that *smed-runt-like1 *RNAi animals have severe eye patterning defects. Animals were treated as described in the Materials and methods section of the main text. Click here for file

Additional file 12 **Expression data mapped onto computational gene predictions**. Differential gene expression data for all gene models predicted by MAKER. Click here for file

Additional file 13 **Custom perl script used for quality filtering of Illumina reads**. Custom perl script used for quality filtering of Illumina reads. Click here for file

Additional file 14 **Illumina+ assembly sequences in fasta format**. Compressed .fasta file containing all Illumina+ assembly sequences. Click here for file

Additional file 15 **Primers used for experimental validation of expression profiles by qRT-PCR**. Primer sequences used for qRT-PCR analysis. Click here for file
