# Supplementary figures and images for: The head-regeneration transcriptome of the planarian Schmidtea mediterranea
Source: Genome Biol. 2011 Aug 16;12(8):R76. doi: 10.1186/gb-2011-12-8-r76 (PMC3245616; doi:10.1186/gb-2011-12-8-r76)

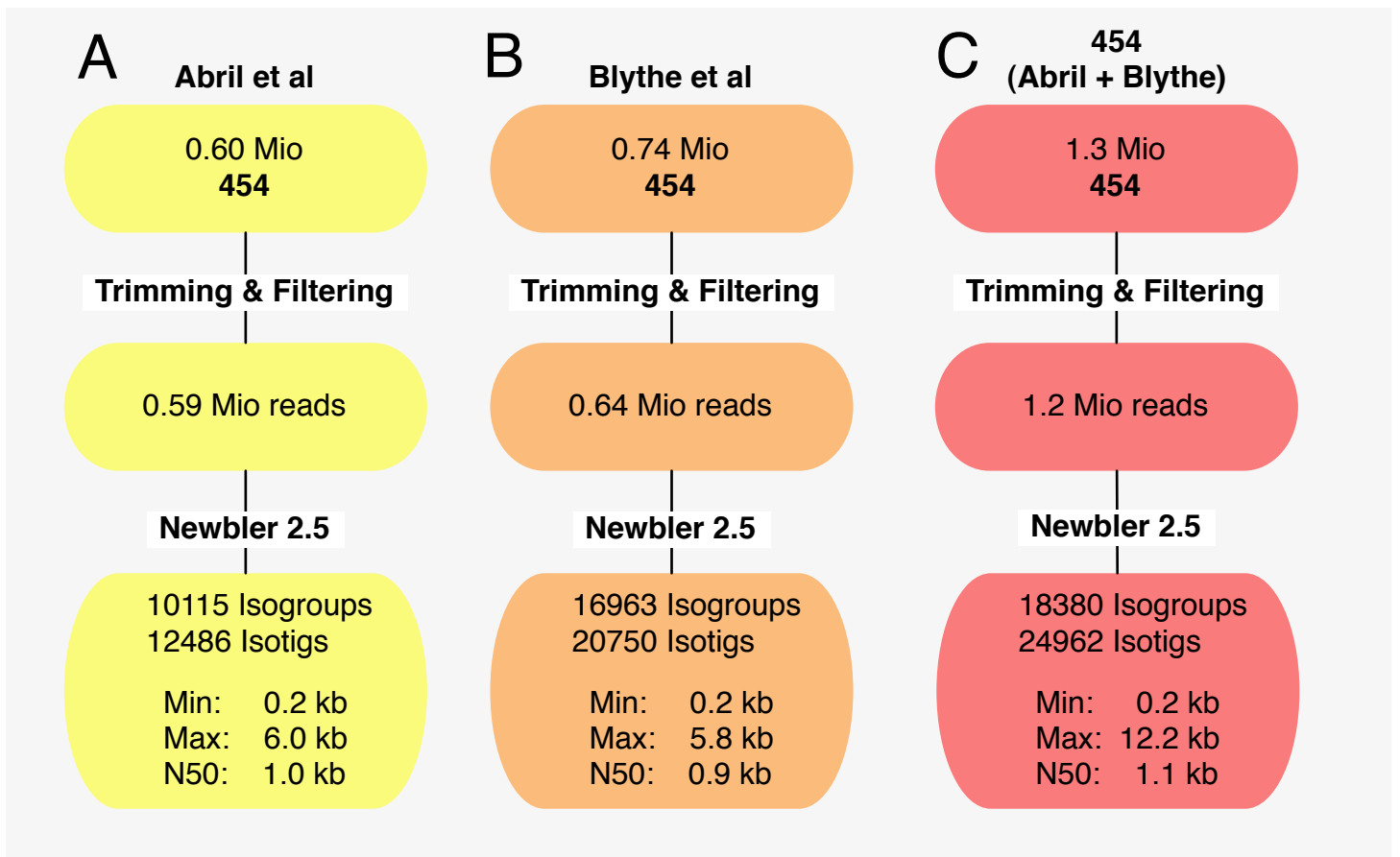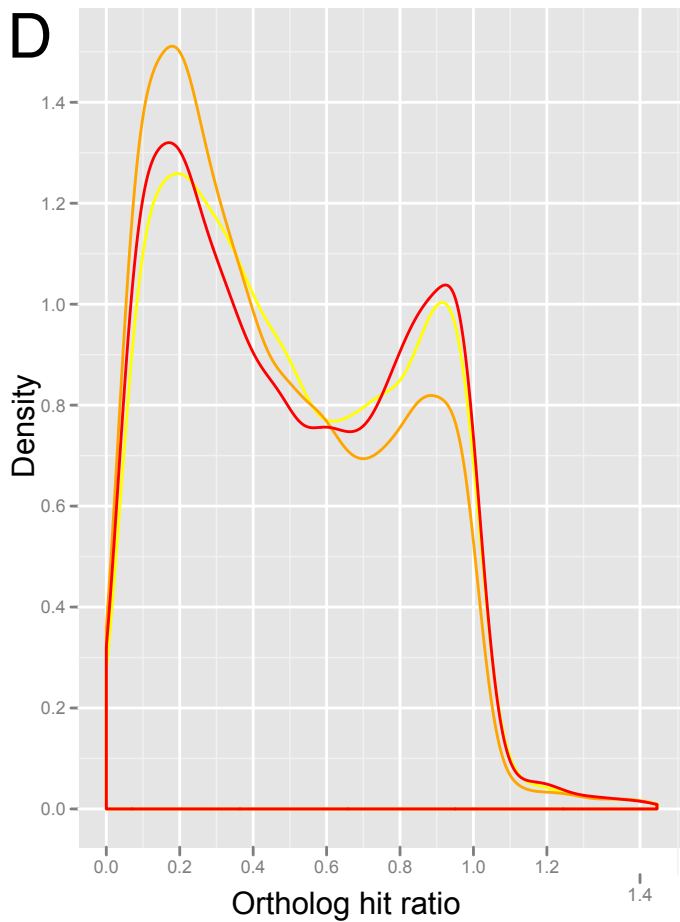

**Dataset**

Abril  
Blythe  
454 (Abril + Blythe)

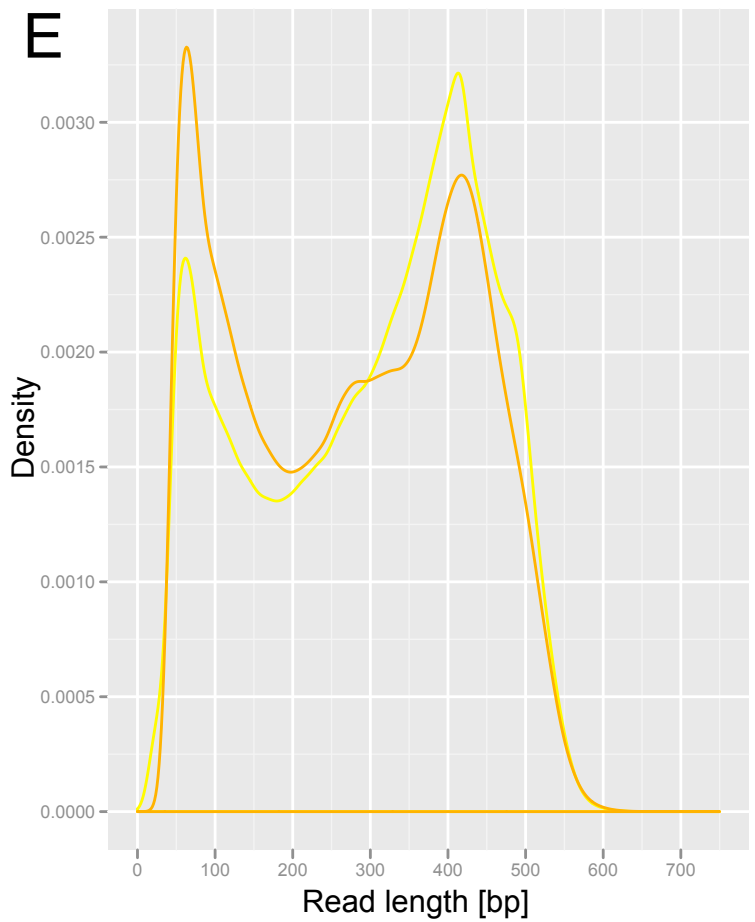

**Dataset**

Abril  
Blythe

Supplement: Additional file 1 — Transcriptome assembly from public 454 data. (a-c) Schematic overview of 454 transcriptome assembly approaches. Publicly available 454 reads were assembled either separately (a, b) or as a combined dataset (c) using the Newbler 2.5 assembler. Colors indicate the three different assemblies: yellow, Abril et al. [17] (a); orange Blythe et al. [18] (b); red, combined (c). Quality metrics shown include the shortest and longest sequences in each assembly, as well as N50, for which 50% of all bases are contained in sequences at least as long as N50. (d) Kernel densities of the length distributions for the assembled sequences. For multi-isoform loci, only the longest isoform was considered. Colors as in (a-c). (e) Kernel densities of ortholog hit ratios obtained by comparing sequences from the different assemblies or computational prediction to the Schistosoma mansoni proteome using blastx. For multi-isoform loci, only the longest isoform was considered. Colors as in (a-c). [file gb-2011-12-8-r76-S1.PDF]

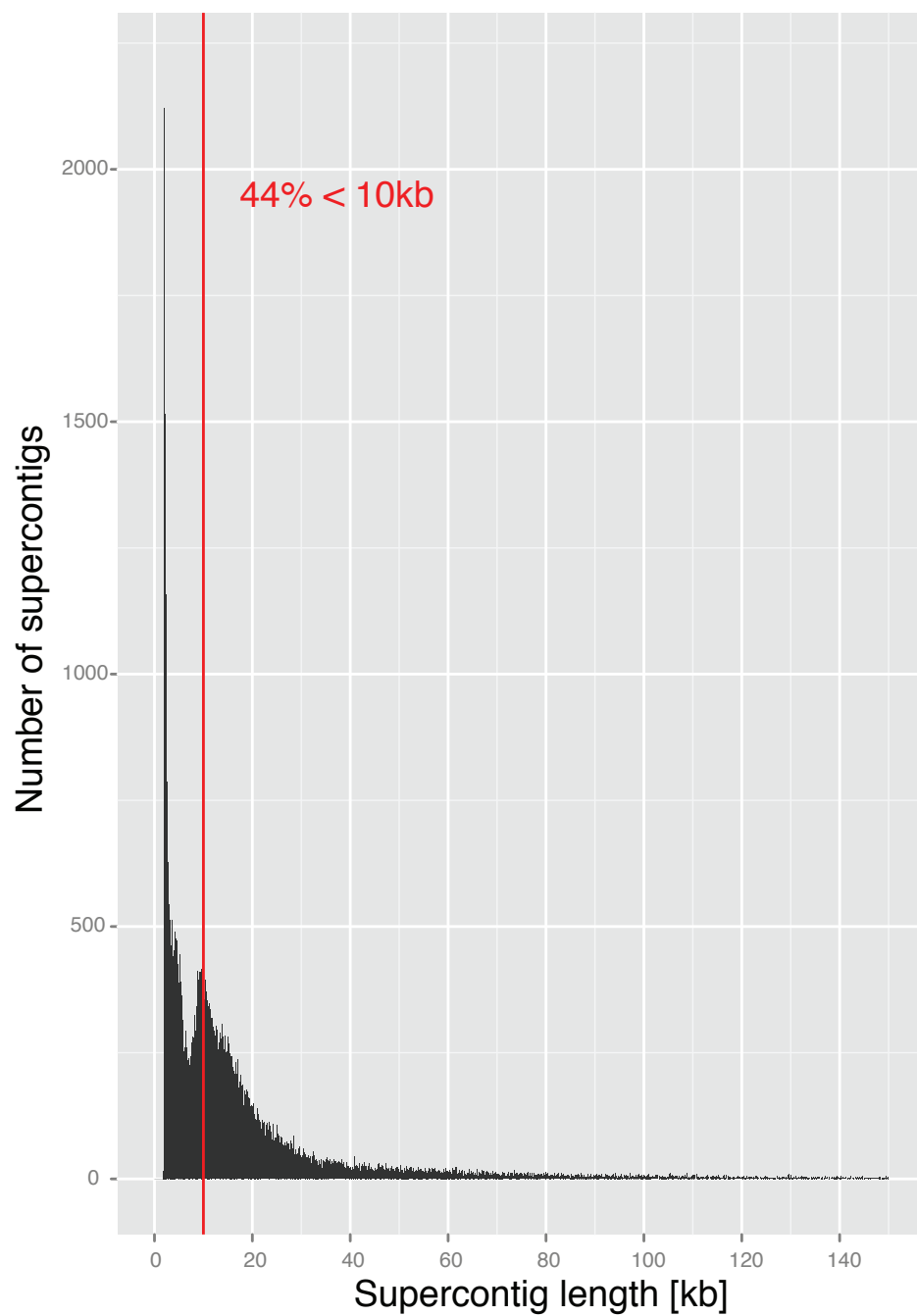

Supplement: Additional file 2 — Genomic supercontigs. This histogram shows the sequence length distribution of all genomic S. mediterranea supercontigs (version 3.1 [13]). The red line indicates a sequence length of 10 kb. [file gb-2011-12-8-r76-S2.PDF]

Similarity of top blastx hits

A

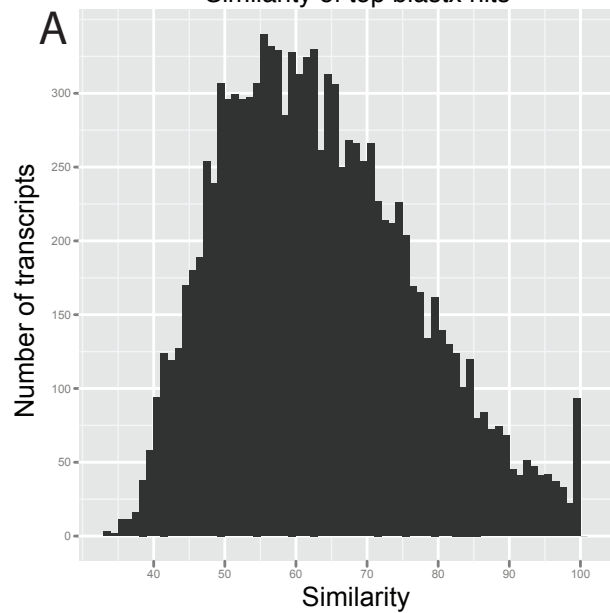

E-values of top blastx hits

B

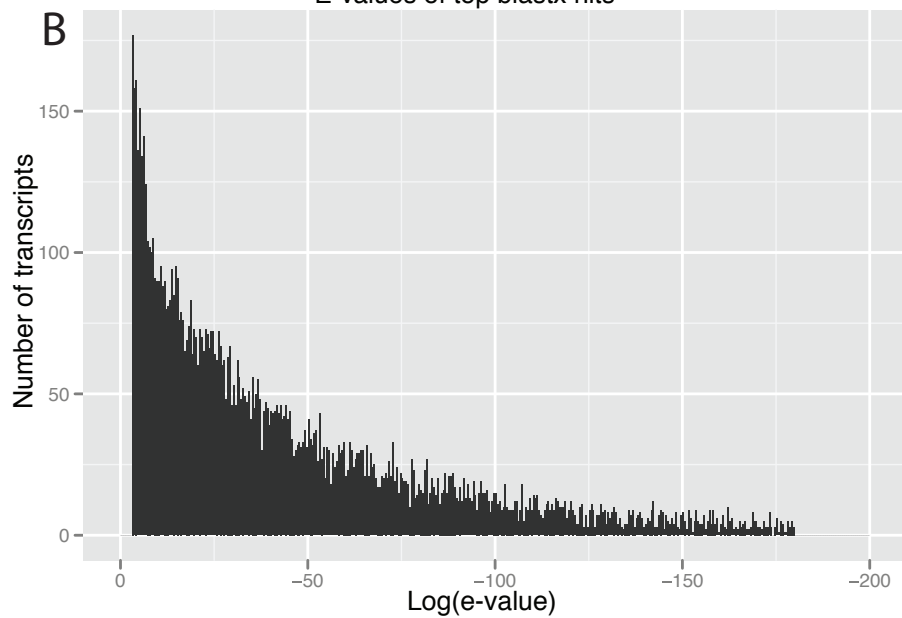

Supplement: Additional file 6 — Blastx comparison of de novo transcriptome sequences with the NCBI non-redundant protein database. (a) Distribution of similarity detected in the best blastx hit for each transcript (blastx e-value < 10-3). (b) Distribution of blastx e-values for the best blastx hit for each transcript. For multi-isoform loci, only the longest isoform was used as a blastx query. [file gb-2011-12-8-r76-S6.PDF]

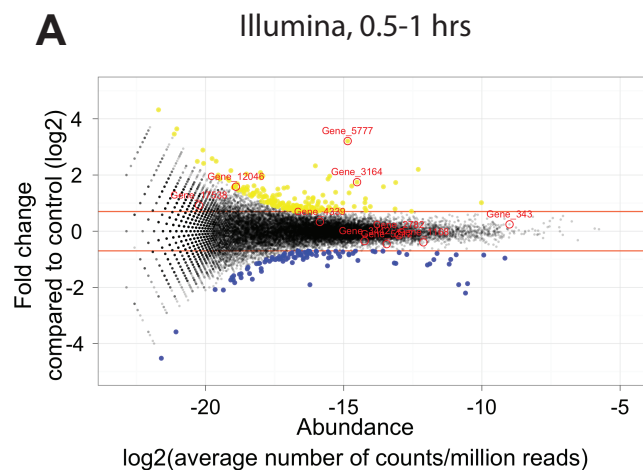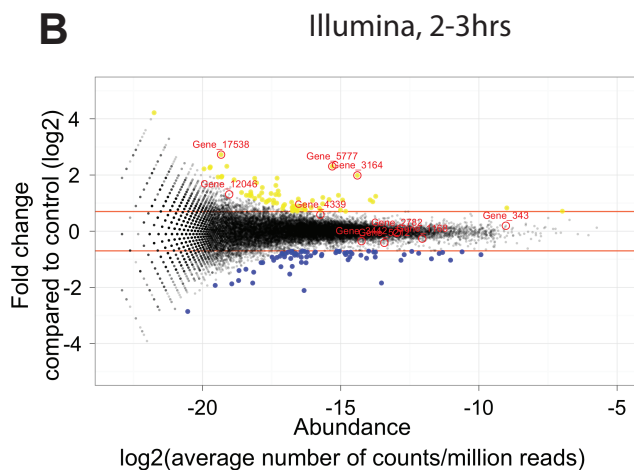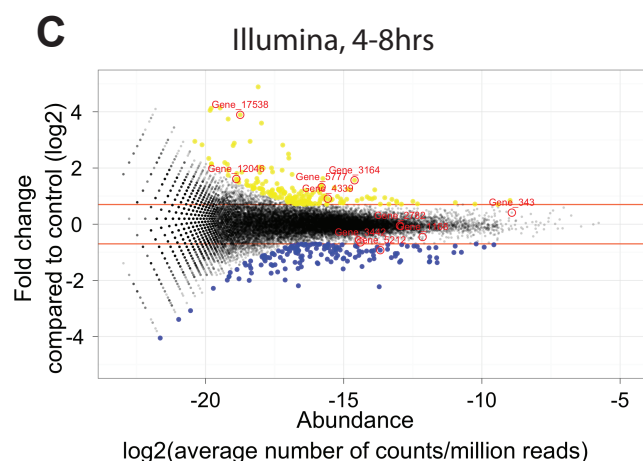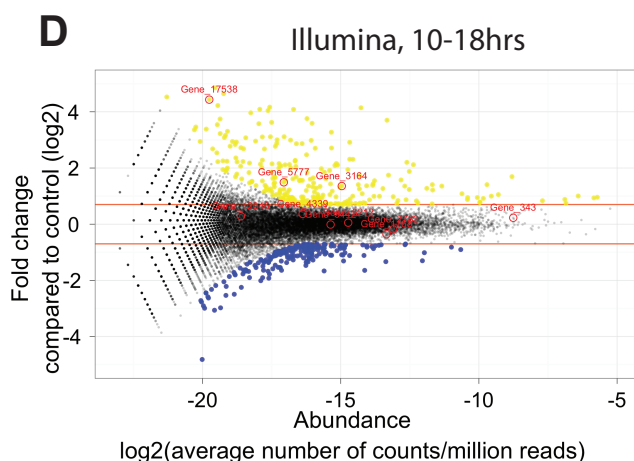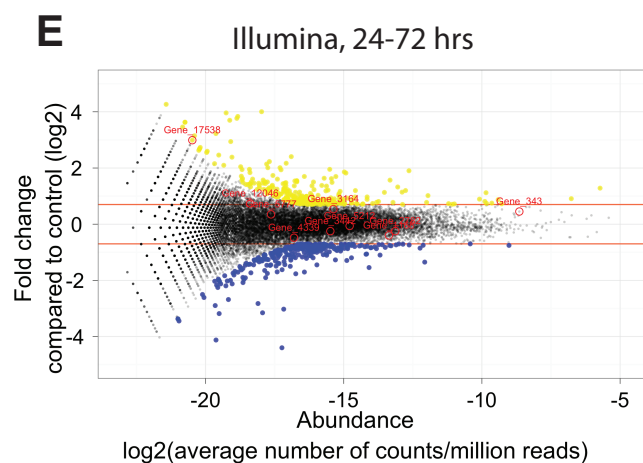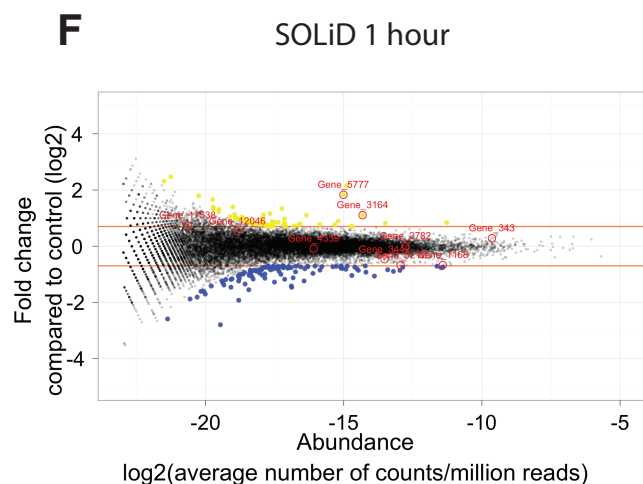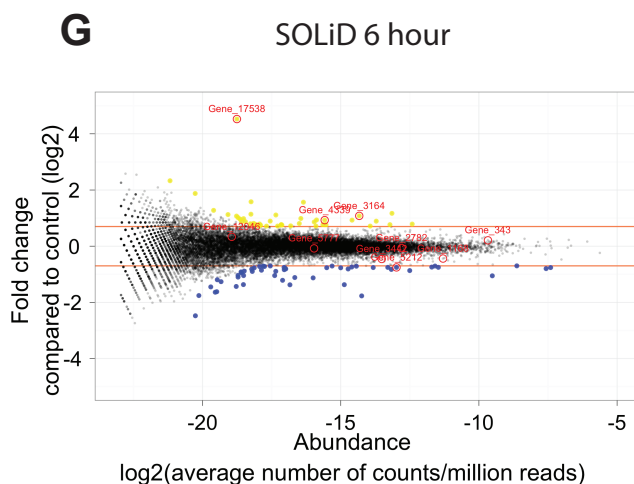

Supplement: Additional file 9 — MA plots illustrating differential gene expression from both Illumina and SOLiD data. For the longest isoform of each locus from the Illumina+ assembly, the expression fold change (log2 scale) relative to the control (0 h) is plotted against its log average abundance (MA plot). Statistically significant up- or down-regulation (adjusted P-value < 0.001 and log2 fold change > 0.7 or < -0.7 (red lines)) is indicated in yellow and blue, respectively. Genes chosen for qRT-PCR validation (Figure 5) are labeled. (a-e) MA plots show a comparison of Illumina transcriptome sequencing (RNAseq) data from 0.5 to 1 h samples (a), 2 to 3 h samples (b), 4 to 8 h samples (c), 10 to 18 h samples (d) and 24 to 72 h (e) relative to controls. (f, g) SOLiD reads from control or regeneration samples were aligned to the genomic supercontigs with Bioscope. MA plots show a comparison of SOLiD RNAseq data from 1 h sample (f) and 6 h sample (g) relative to controls. [file gb-2011-12-8-r76-S9.PDF]
