# Supplementary material for: The head-regeneration transcriptome of the planarian Schmidtea mediterranea
Source: Genome Biol. 2011 Aug 16;12(8):R76. doi: 10.1186/gb-2011-12-8-r76 (PMC3245616; doi:10.1186/gb-2011-12-8-r76)

Identification of supercontig-spanning transcripts

Transcripts spanning multiple genomic supercontigs were identified in a multi-step, semi-automated procedure:

1.) Only transcripts with a minimum length of 1 kb were considered. For gene models with multiple isoforms, the longest isoform was evaluated.

2.) Each transcript was split into non-overlapping 0.5 kb subsequences, which were aligned to the genomic supercontigs individually using blat. For each subsequence, the best mapping result was retained, requiring at least 450/500 (90%) perfectly matched bases over 450/500 (90%) of its length. 1449 transcripts, whose subsequences mapped to at least two different supercontigs, were retained as putative supercontig-spanning sequences.

3.) The 1449 full-length candidate transcripts were re-mapped to the genomic supercontigs using blastn (e-value cutoff 10E-40) and to the NCBI non-redundant protein database (e-value cutoff 10E-3). Schematic representations of the blastn/blastx alignments were inspected manually. Transcripts were considered to be supercontig-joining only, when one or more junctions between genomic fragments were independently supported by overlapping, continuous homology to a known protein. Based on these criteria, 413 high-confidence supercontig-joining transcripts were identified (see below).

E-value color scale for all alignments shown below

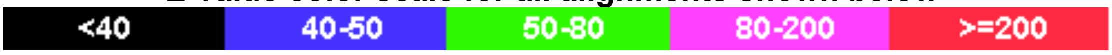

Annotation of the schematic alignments:

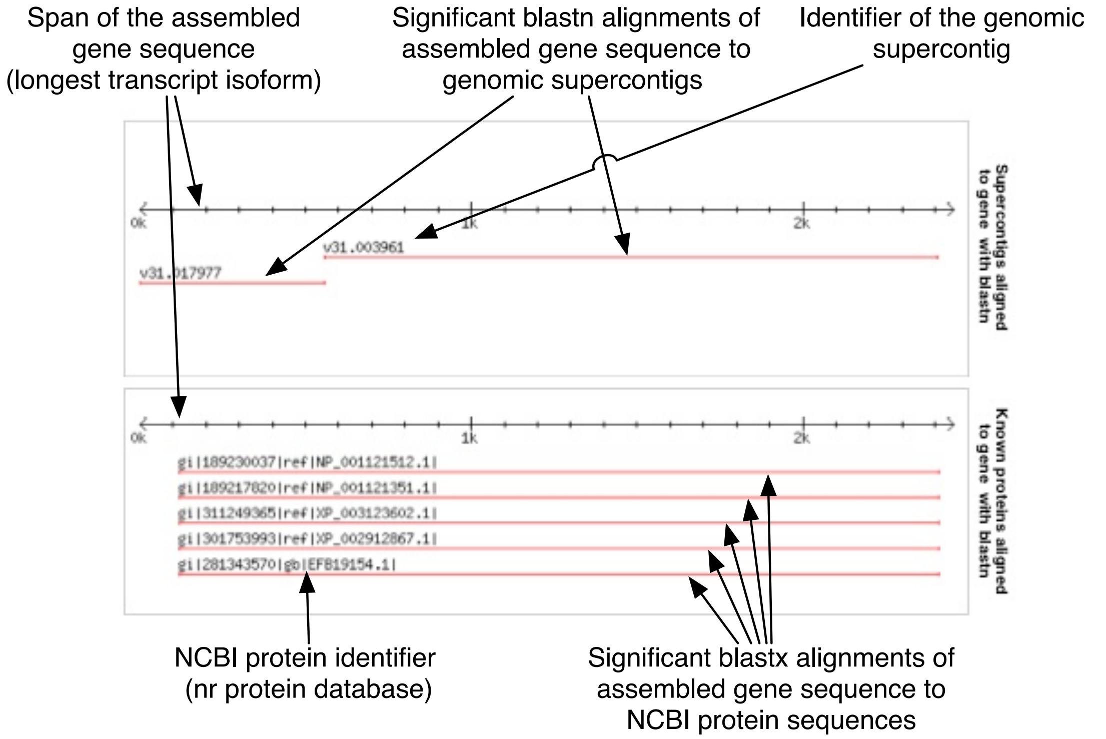

Gene_10019

Supercontigs mapped to this gene

v31.004218+, v31.010310+, v31.016491-, v31.026963+, v31.027166+

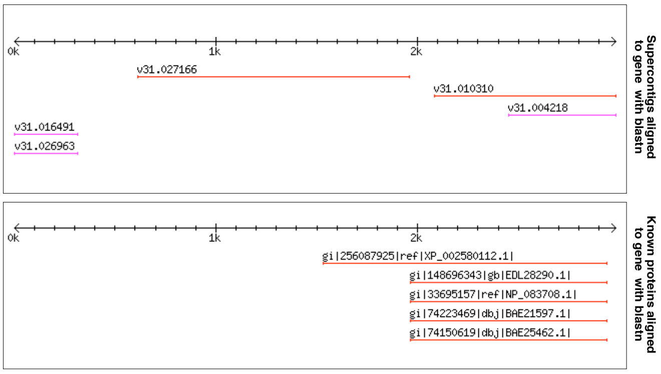

Gene_10185

Supercontigs mapped to this gene

v31.005072+, v31.008752-, v31.012049-, v31.020704-

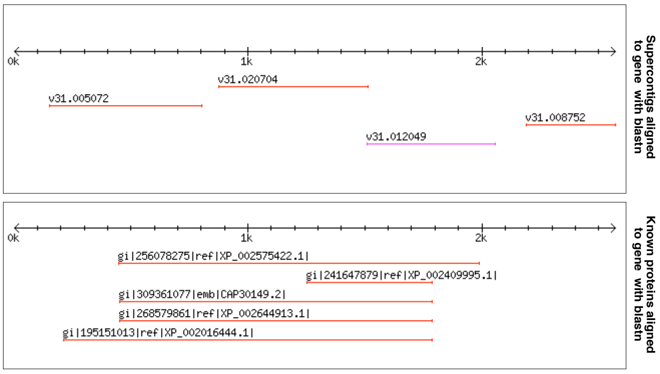

Gene_10092

Supercontigs mapped to this gene

v31.000966+, v31.005560+, v31.008968+, v31.021831-

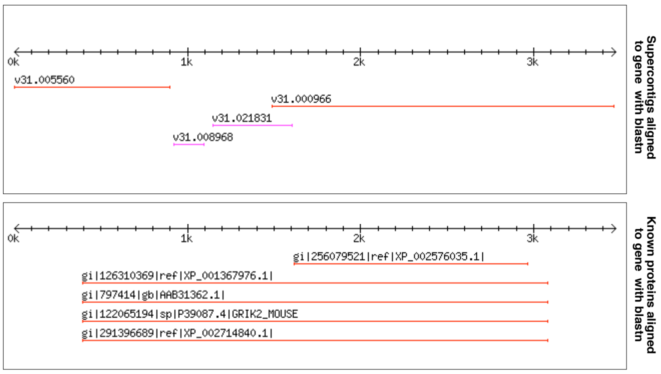

Gene_10218

Supercontigs mapped to this gene

v31.010378-, v31.011495-, v31.022697+, v31.022788-, v31.024215+, v31.025845-, v31.028102-, v31.035415-, v31.047159-

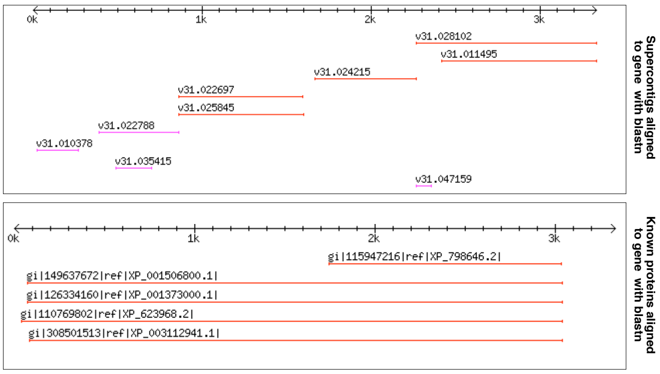

Gene_01024

Supercontigs mapped to this gene

v31.002149+, v31.003093-, v31.023586+

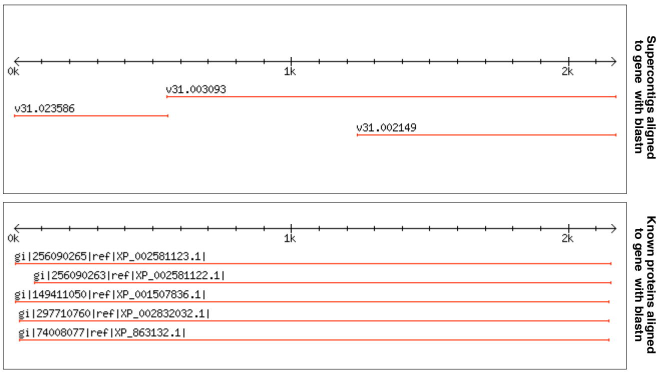

Gene_01033

Supercontigs mapped to this gene

v31.000152+, v31.005068-

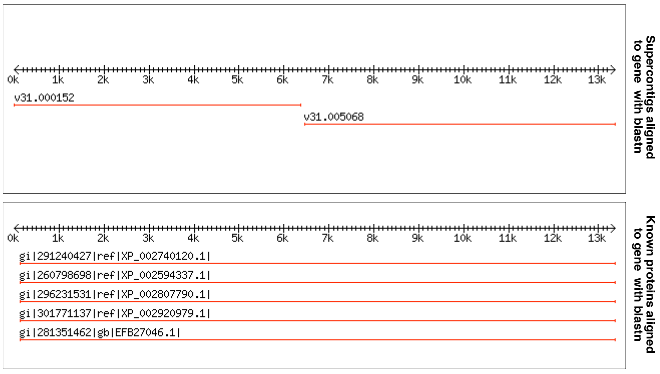

Gene_01030

Supercontigs mapped to this gene

v31.004852-, v31.005434-, v31.005639-, v31.006220+, v31.008429+, v31.008692-, v31.012414+, v31.014934-, v31.018275+, v31.043975-

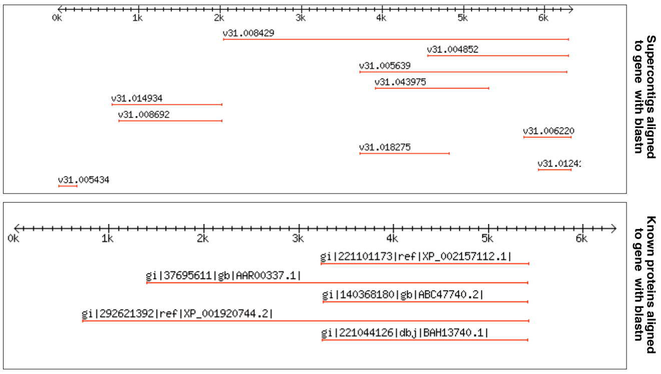

Gene_10355

Supercontigs mapped to this gene

v31.007788-, v31.018634+, v31.032217+

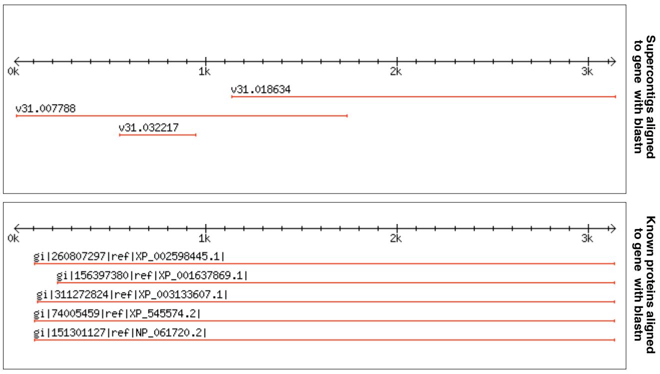

Gene_10368

Supercontigs mapped to this gene

v31.006204-, v31.014078+, v31.018011+, v31.037083+

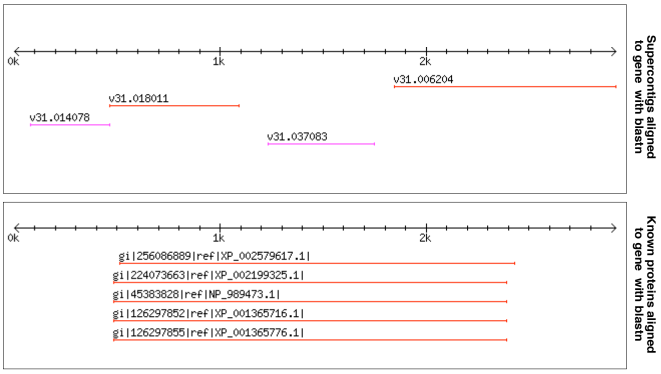

Gene_10667

Supercontigs mapped to this gene

v31.001964-, v31.002777-

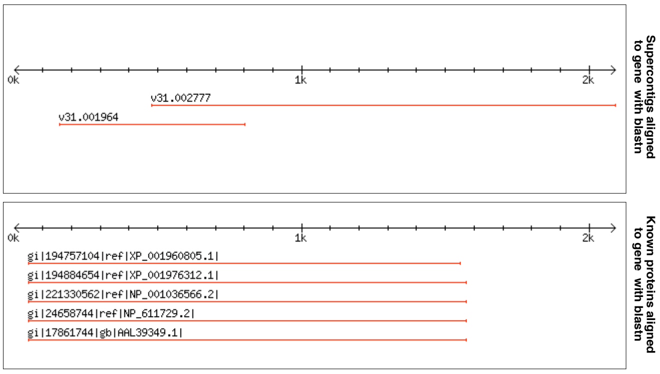

Gene_10518

Supercontigs mapped to this gene

v31.004595-, v31.005286-, v31.019381-, v31.023806-, v31.033092-

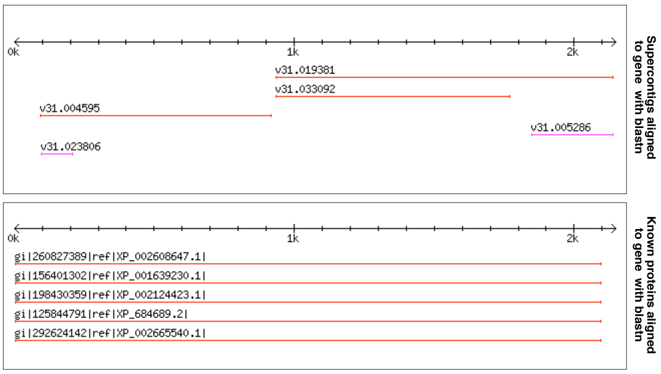

Gene_01083

Supercontigs mapped to this gene

v31.021618-, v31.025020-

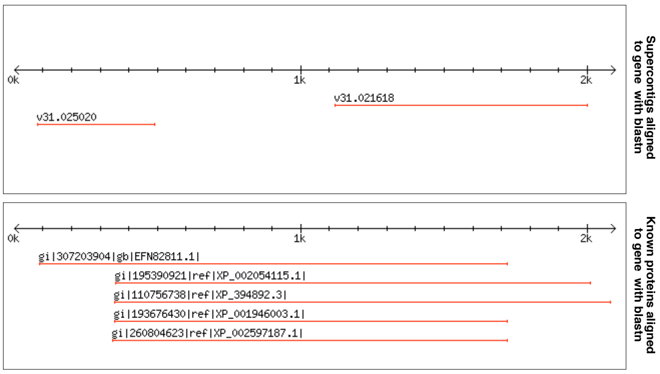

Gene_01085

Supercontigs mapped to this gene

v31.015768-, v31.028346+, v31.028726-

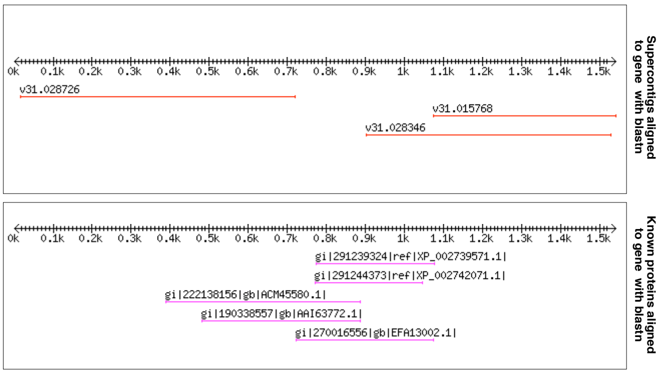

Gene_01093

Supercontigs mapped to this gene

v31.004236-, v31.004742-, v31.015927-

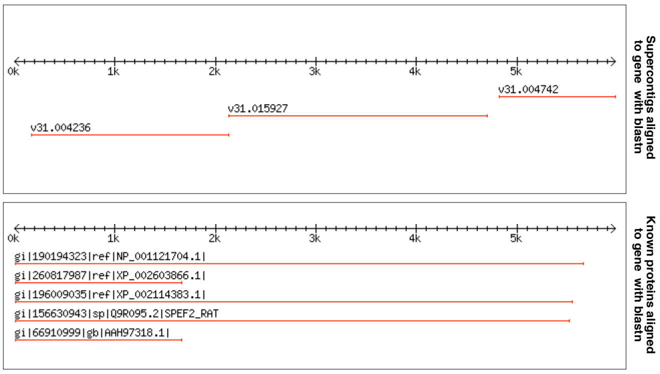

Gene_10850

Supercontigs mapped to this gene

v31.001179+, v31.001281-

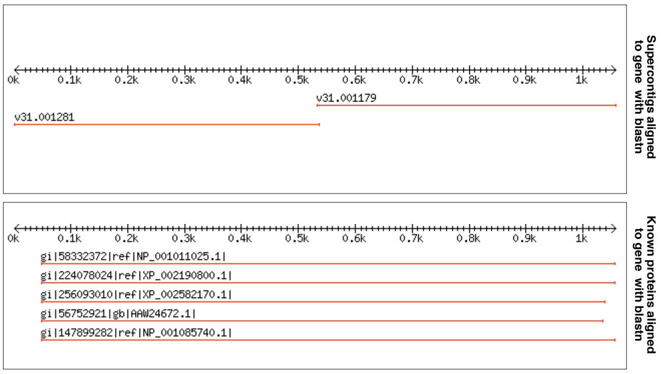

Gene_11059

Supercontigs mapped to this gene

v31.007836+, v31.016581-

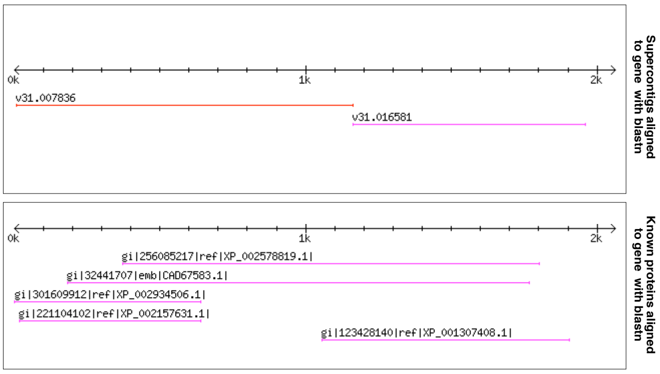

Gene_11077

Supercontigs mapped to this gene
v31.007475+, v31.020457+, v31.023208+, v31.066546-

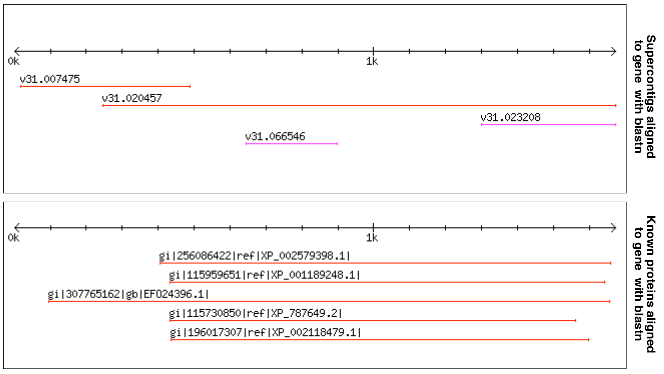

Gene_11152

Supercontigs mapped to this gene
v31.009098+, v31.018992-, v31.037342+

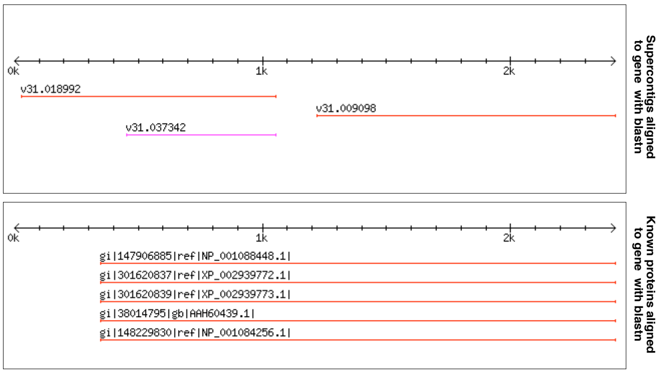

Gene_01111

Supercontigs mapped to this gene
v31.008673+, v31.012276+

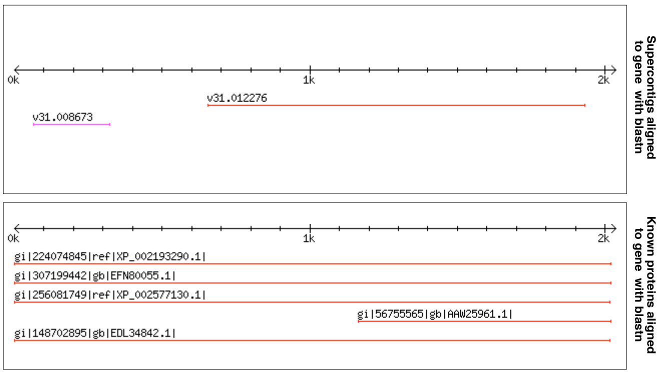

Gene_01121

Supercontigs mapped to this gene
v31.011965+, v31.013168-, v31.024968-, v31.035519+

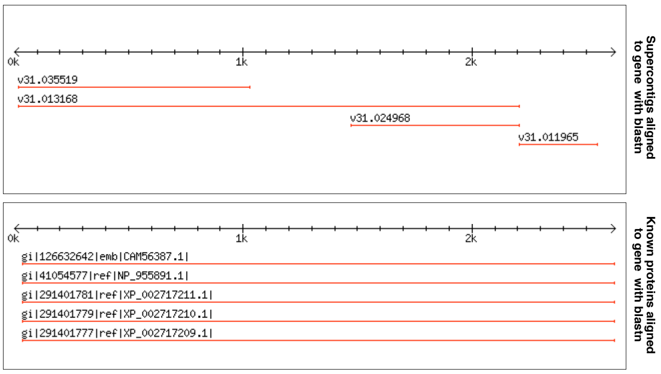

Gene_11306

Supercontigs mapped to this gene
v31.001681-, v31.009324+, v31.011183+, v31.012172+

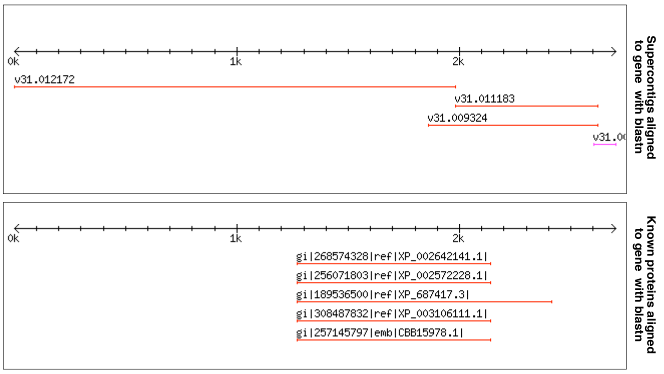

Gene_11507

Supercontigs mapped to this gene
v31.003961-, v31.017977-

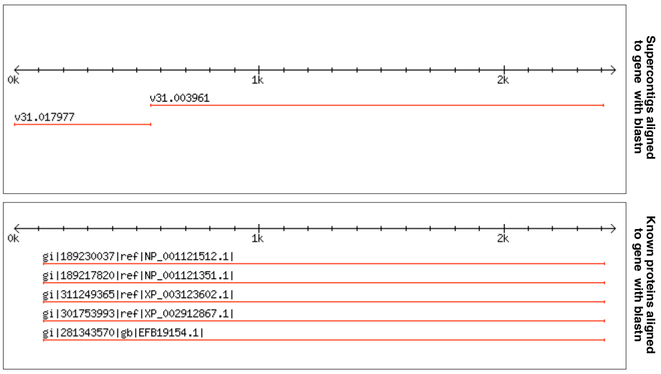

Gene_11313

Supercontigs mapped to this gene
v31.009046-, v31.014747+, v31.025831-, v31.031847+, v31.039135-

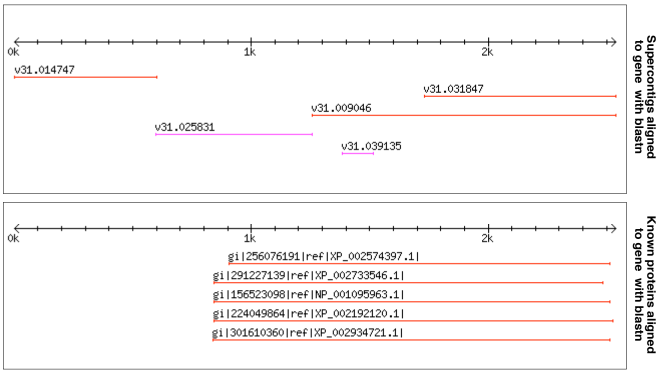

Gene_01153

Supercontigs mapped to this gene
v31.005212+, v31.018720+, v31.023390+, v31.042143-

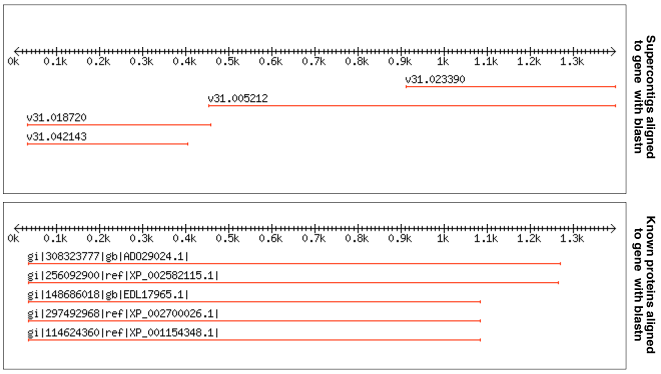

Gene_01158

Supercontigs mapped to this gene

v31.009844-, v31.011582-, v31.014604+, v31.017347-, v31.020880+, v31.020880-

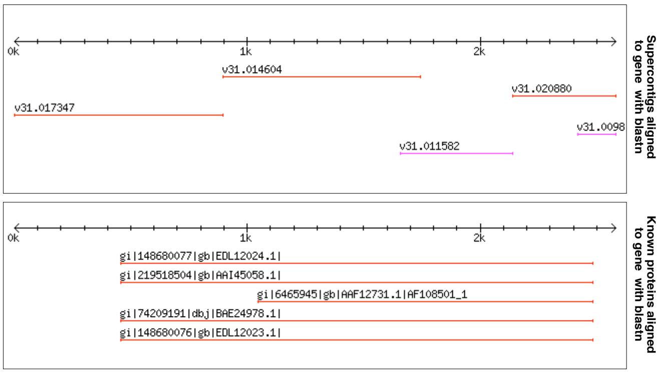

Gene_11725

Supercontigs mapped to this gene

v31.001440+, v31.002200-, v31.018073-

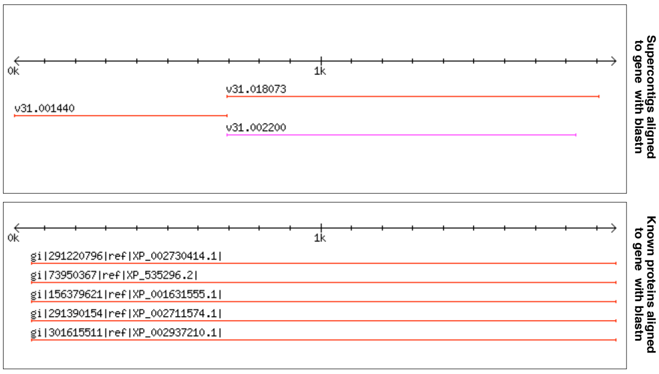

Gene_11703

Supercontigs mapped to this gene

v31.007610-, v31.016428+, v31.024340-

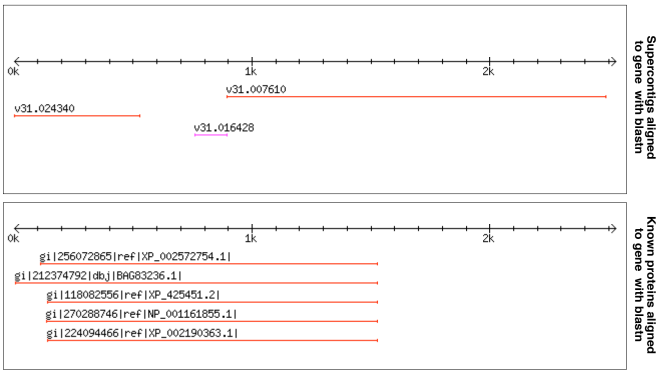

Gene_11743

Supercontigs mapped to this gene

v31.017167+, v31.018308-, v31.027091-, v31.027972-

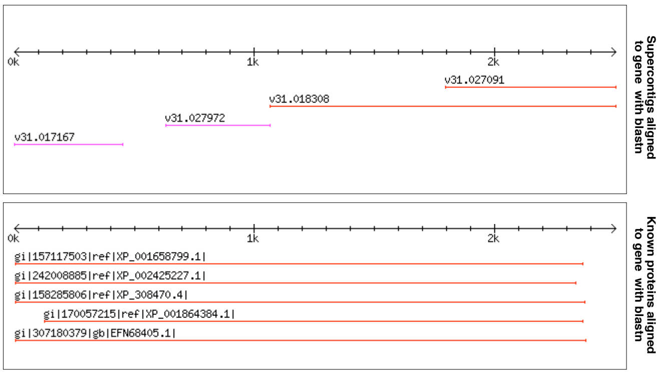

Gene_01175

Supercontigs mapped to this gene

v31.014525+, v31.017269-

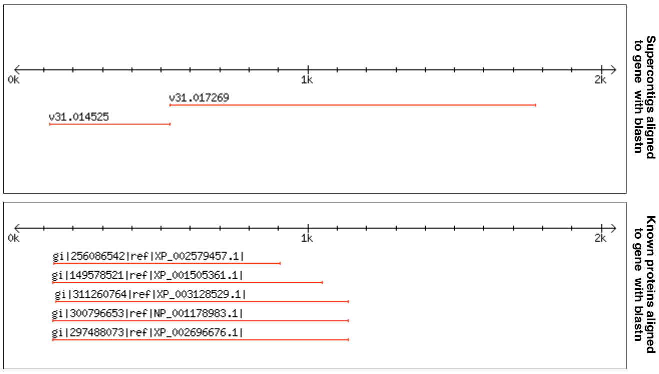

Gene_01201

Supercontigs mapped to this gene

v31.014945-, v31.035350+

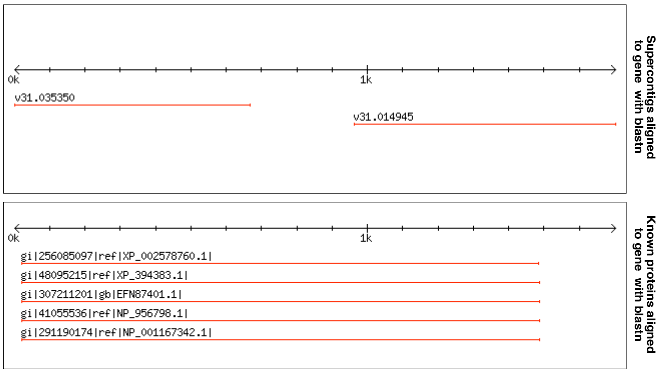

Gene_11877

Supercontigs mapped to this gene

v31.004193-, v31.018480+

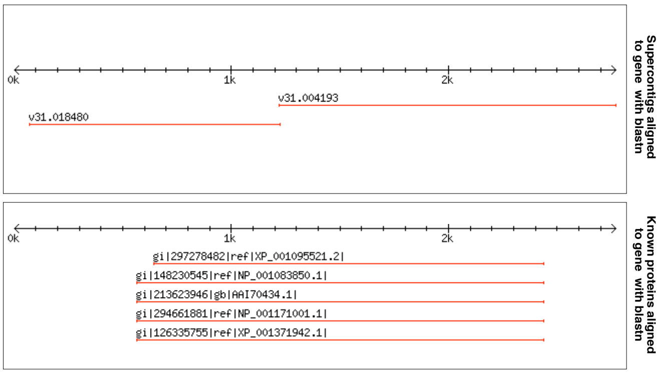

Gene_01259

Supercontigs mapped to this gene

v31.001683+, v31.004208-

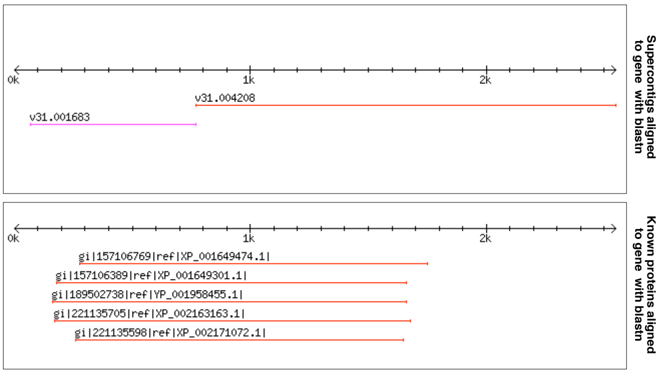

Gene_01273

Supercontigs mapped to this gene

v31.000421+, v31.001170-, v31.002904+

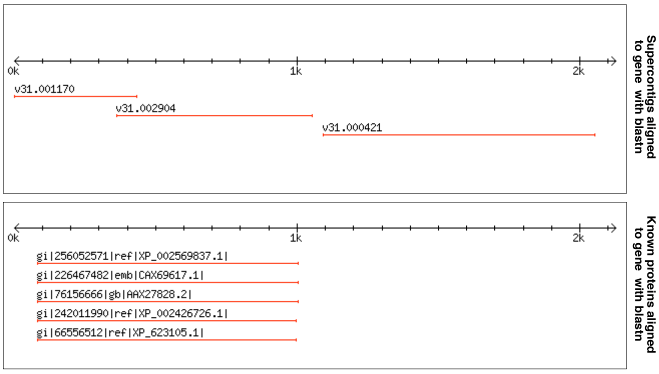

Gene_01291

Supercontigs mapped to this gene

v31.000388-, v31.001737-, v31.002569-, v31.002815-

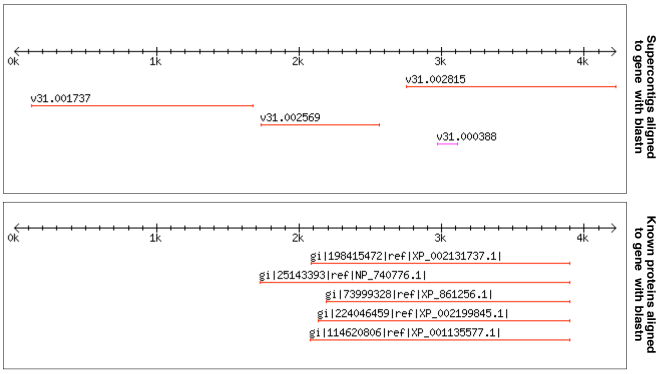

Gene_01280

Supercontigs mapped to this gene

v31.006245-, v31.013956-, v31.015455-, v31.019845-, v31.023959-, v31.024957-, v31.043206+

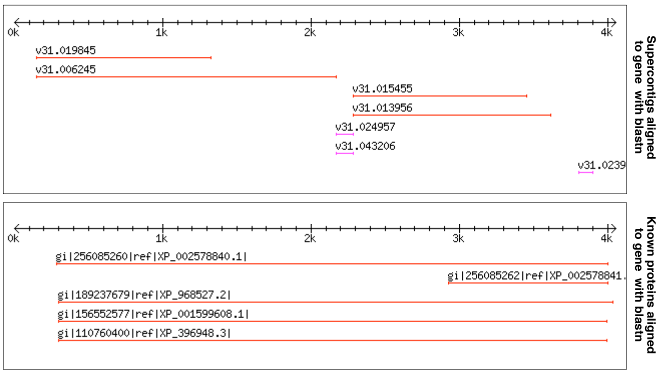

Gene_01297

Supercontigs mapped to this gene

v31.005008-, v31.009511-, v31.015096-, v31.015296-, v31.017929-, v31.021835+, v31.033952+, v31.037522-, v31.041505-

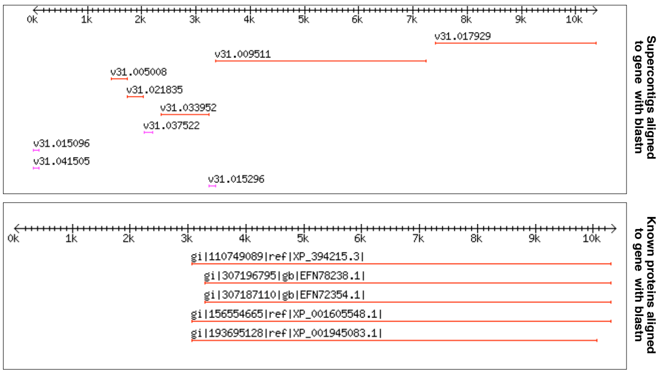

Gene_01306

Supercontigs mapped to this gene

v31.000563+, v31.005800-

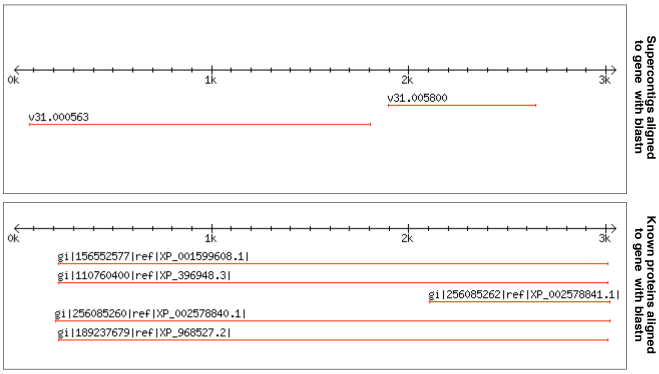

Gene_01322

Supercontigs mapped to this gene

v31.001972-, v31.005873+

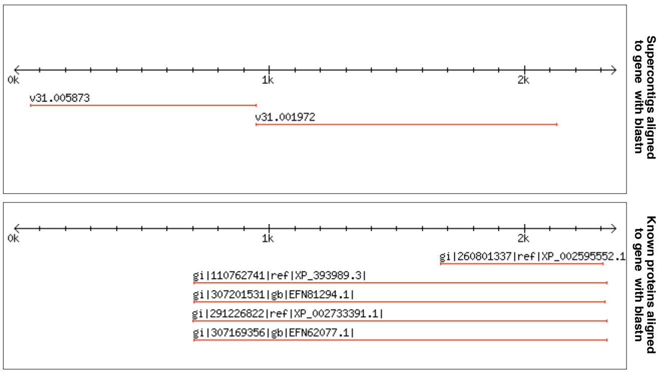

Gene_01313

Supercontigs mapped to this gene

v31.003104+, v31.010639+, v31.013056+, v31.014184+

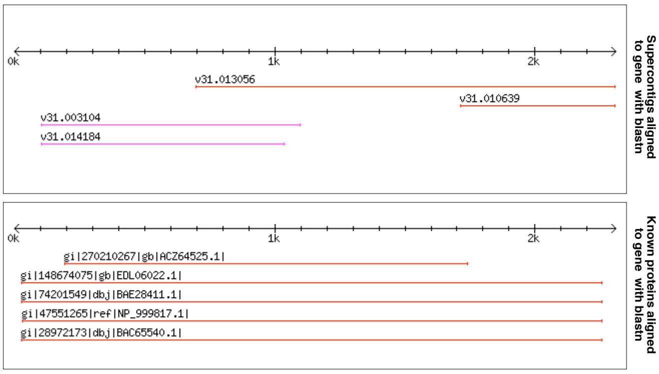

Gene_13233

Supercontigs mapped to this gene

v31.003392-, v31.004736+, v31.021180-

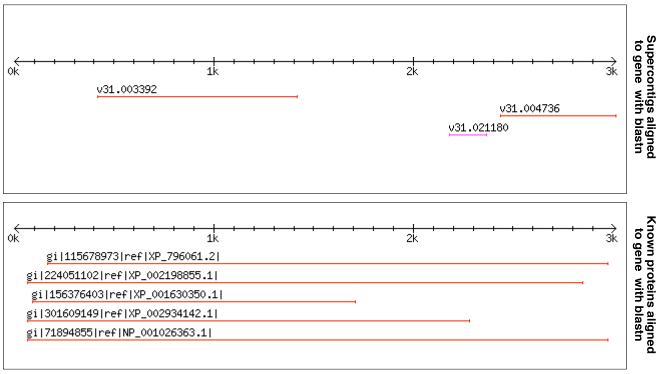

Gene_01329

Supercontigs mapped to this gene
v31.000564+, v31.014486+

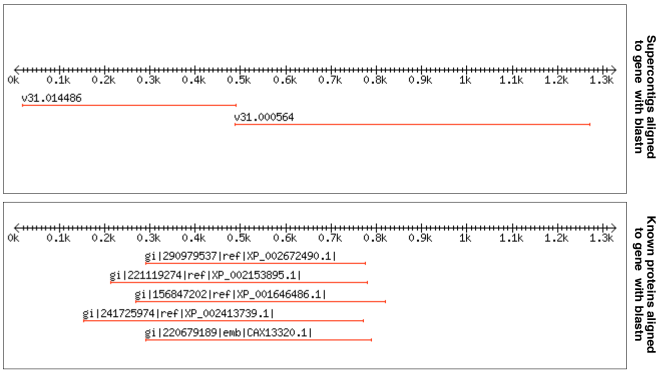

Gene_01354

Supercontigs mapped to this gene
v31.005108-, v31.020993-, v31.023503+, v31.037407-, v31.064718-

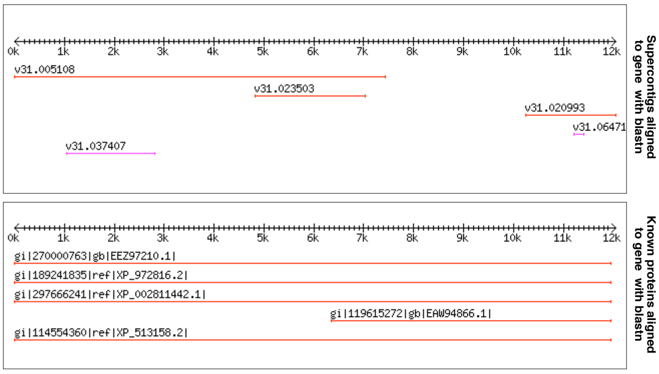

Gene_01330

Supercontigs mapped to this gene
v31.010483-, v31.017599-, v31.018777+, v31.020811-

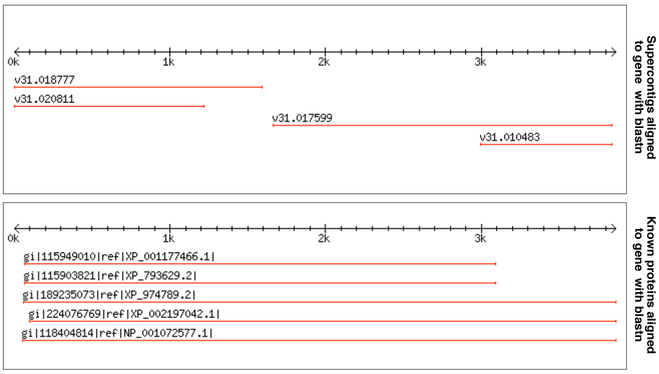

Gene_01358

Supercontigs mapped to this gene
v31.000686-, v31.002668-

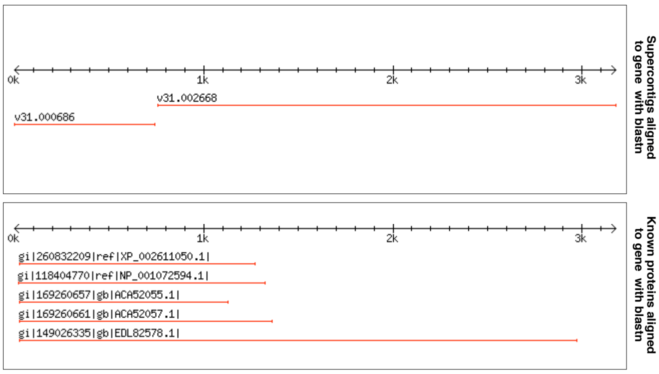

Gene_01393

Supercontigs mapped to this gene
v31.000624+, v31.008480+, v31.025309-

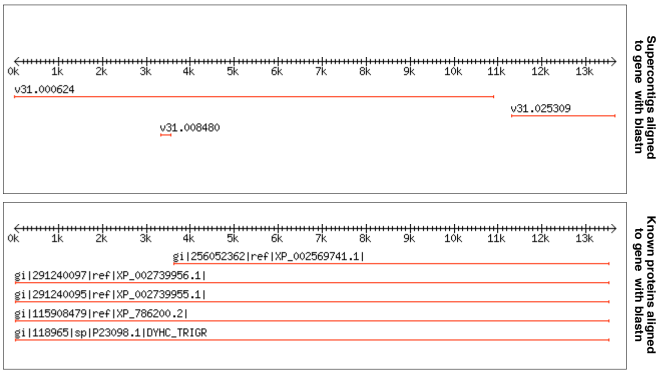

Gene_00141

Supercontigs mapped to this gene
v31.001097+, v31.001574-, v31.007730+, v31.015160+

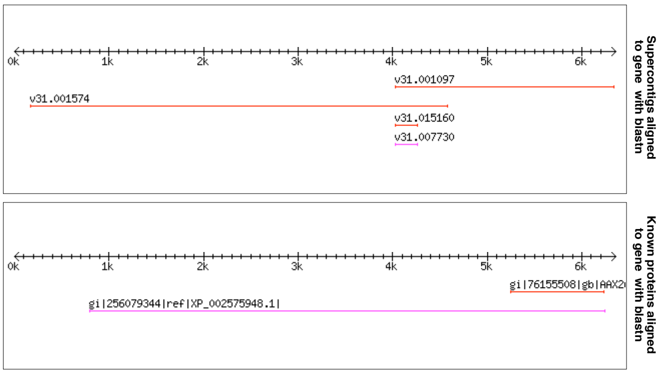

Gene_01400

Supercontigs mapped to this gene
v31.000060-, v31.008212+, v31.008391+

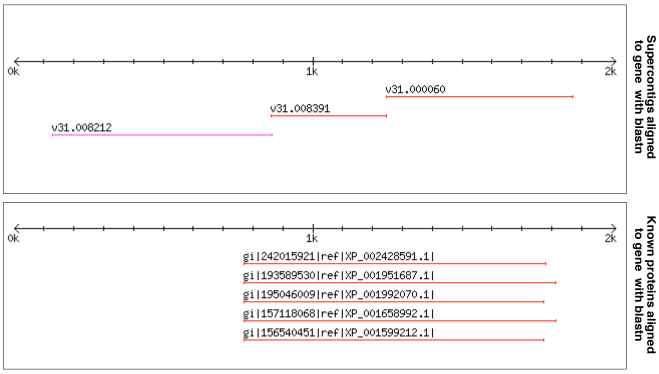

Gene_01412

Supercontigs mapped to this gene
v31.012234-, v31.016028-, v31.019161+, v31.027999-, v31.031302+, v31.039675+, v31.076890-

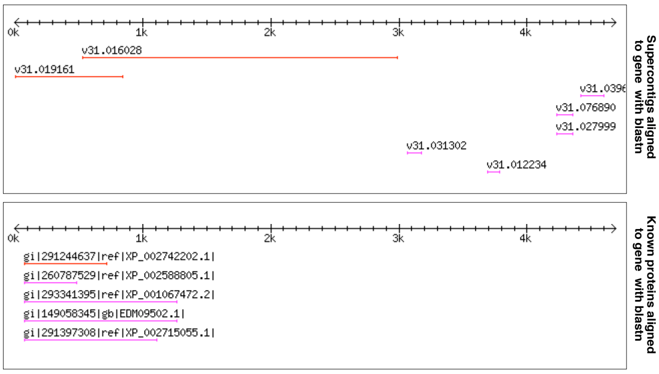

Gene_01413

Supercontigs mapped to this gene

v31.000601+, v31.002944-

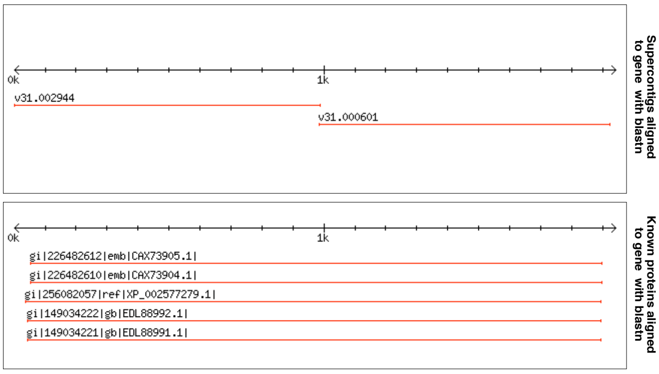

Gene_01415

Supercontigs mapped to this gene

v31.000595-, v31.001431+

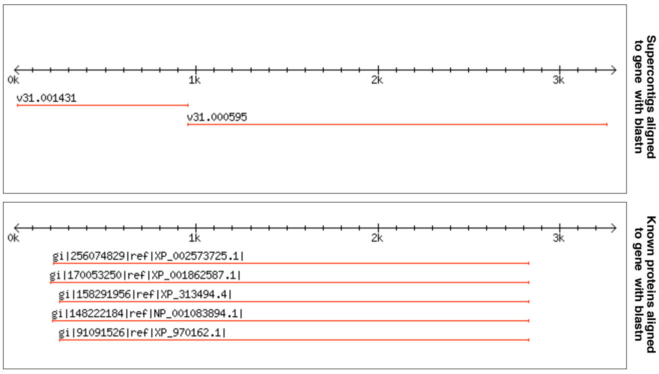

Gene_01414

Supercontigs mapped to this gene

v31.005380-, v31.006051-, v31.006461-, v31.013416+, v31.017651+, v31.023980-, v31.032568+, v31.032595+, v31.036449-

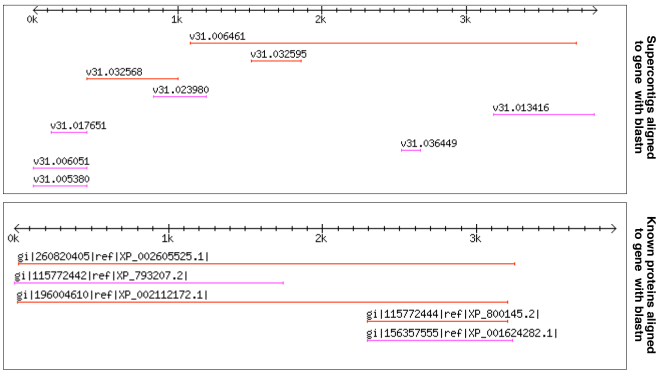

Gene_01420

Supercontigs mapped to this gene

v31.011136-, v31.016419-, v31.019885+, v31.024440-, v31.025556+, v31.026195+, v31.027121-, v31.046453+

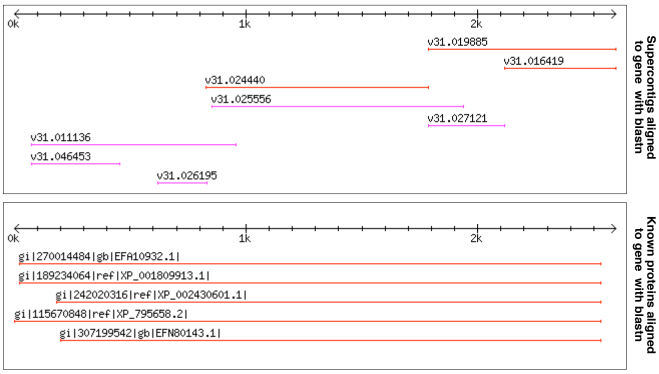

Gene_01443

Supercontigs mapped to this gene

v31.001225-, v31.004081+, v31.005632+

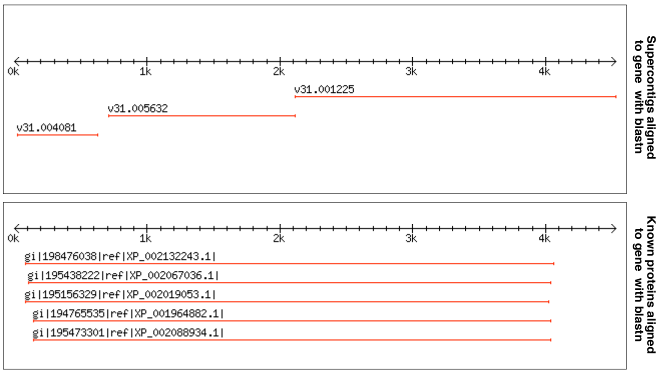

Gene_01499

Supercontigs mapped to this gene

v31.003675+, v31.006555+, v31.017982+, v31.036899+

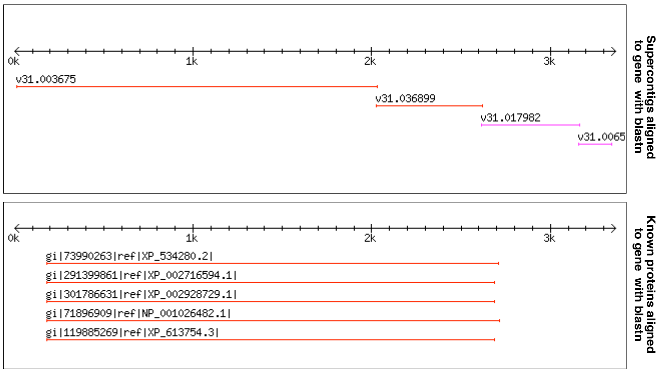

Gene_00147

Supercontigs mapped to this gene

v31.015180+, v31.015657+, v31.020267-, v31.023004+

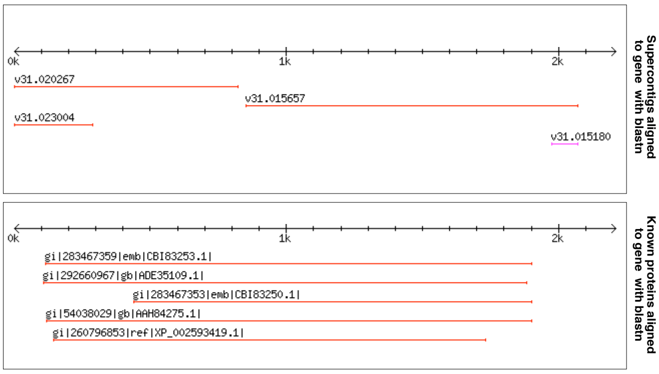

Gene_00150

Supercontigs mapped to this gene

v31.001933+, v31.002929+

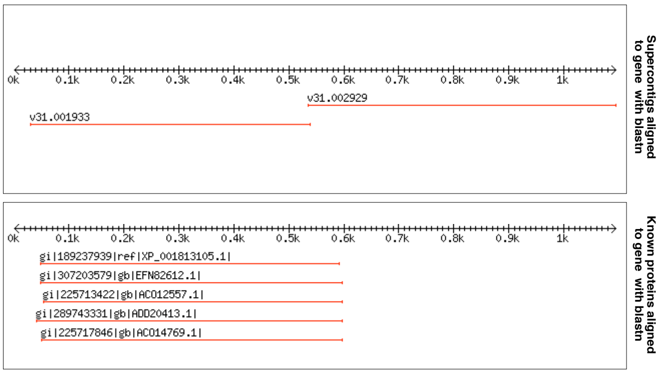

Gene_01513

Supercontigs mapped to this gene

v31.022638-, v31.022688+, v31.027159-, v31.035352-

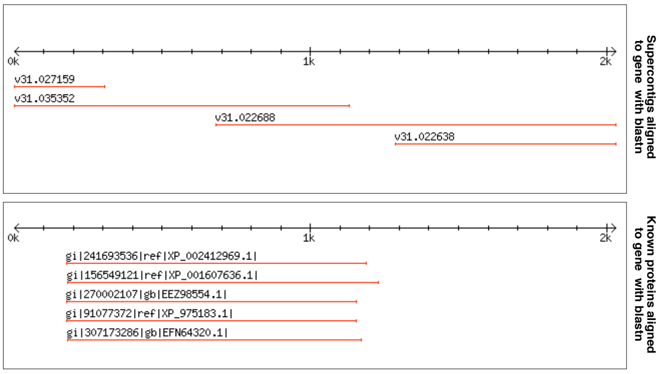

Gene_01561

Supercontigs mapped to this gene

v31.002630+, v31.003056+

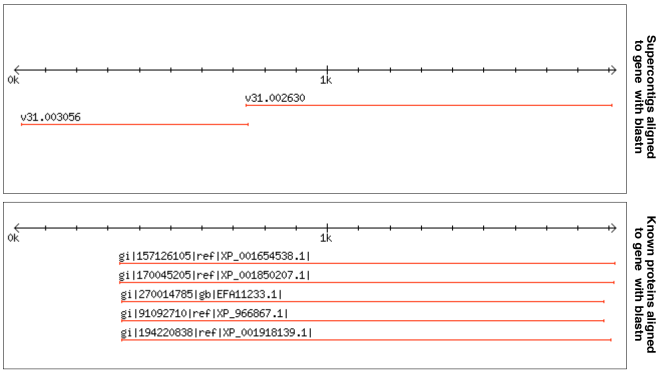

Gene_01517

Supercontigs mapped to this gene

v31.003839+, v31.007006-

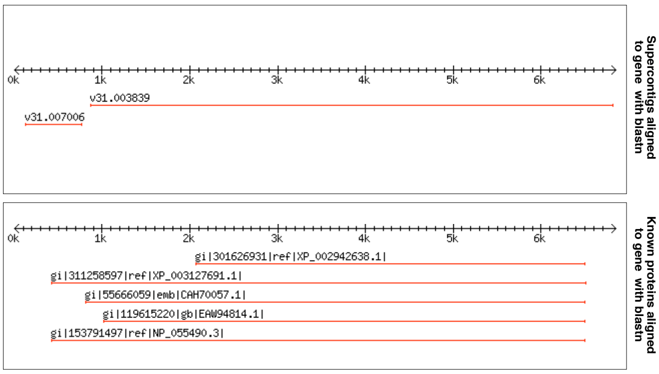

Gene_01592

Supercontigs mapped to this gene

v31.002703+, v31.007023+

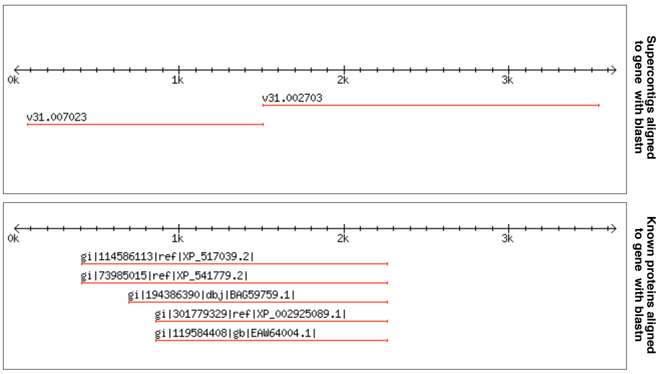

Gene_01598

Supercontigs mapped to this gene

v31.008104-, v31.013804+, v31.021262+

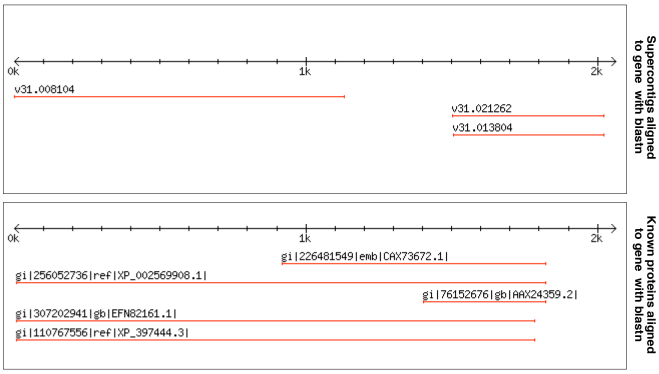

Gene_01628

Supercontigs mapped to this gene

v31.000963+, v31.004059-

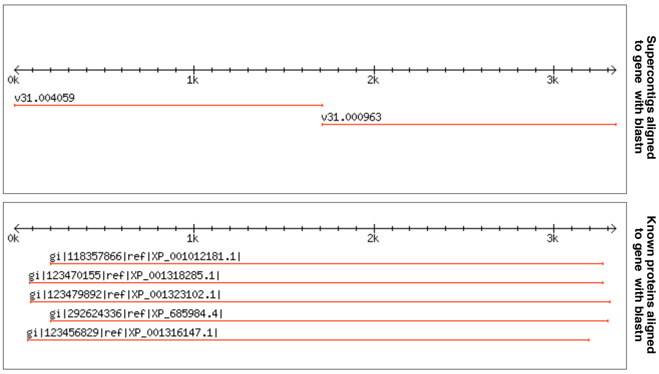

Gene_00016

Supercontigs mapped to this gene

v31.001610-, v31.002393+, v31.009998+, v31.015149-, v31.025540-

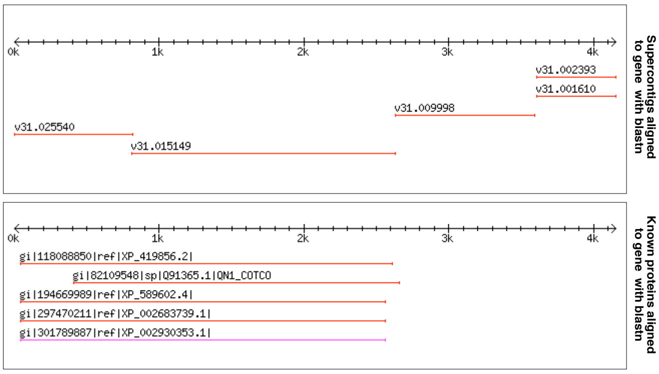

Gene_01650

Supercontigs mapped to this gene

v31.003684+, v31.005112+

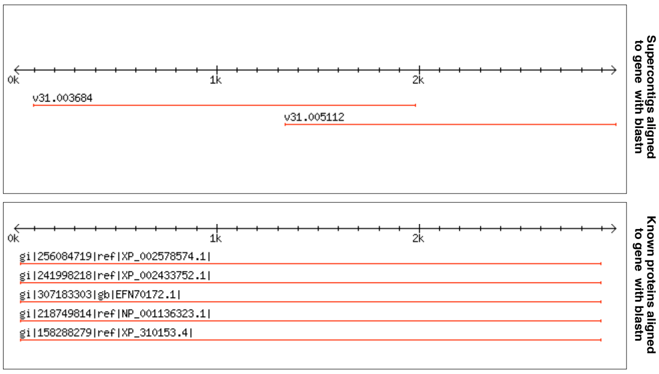

Gene_01713

Supercontigs mapped to this gene
v31.007128+, v31.008820-, v31.011281-, v31.015495+, v31.018296+, v31.019034+, v31.022093+, v31.025537-

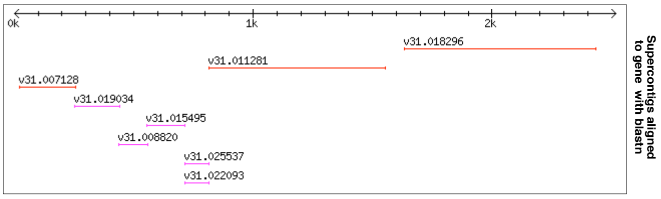

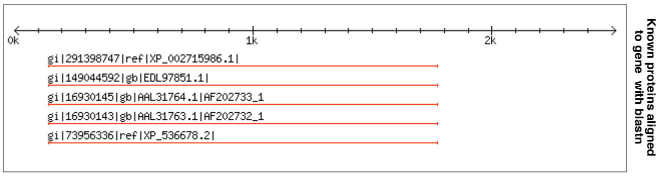

Gene_01721

Supercontigs mapped to this gene
v31.001014+, v31.010013-, v31.010241+, v31.011511+, v31.021398+, v31.024469+, v31.040271-, v31.043998-

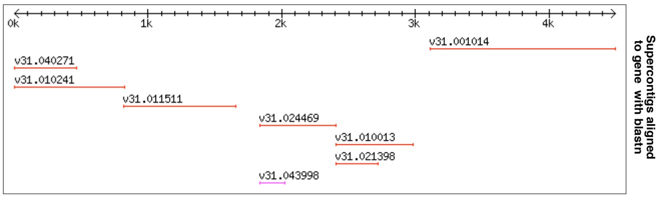

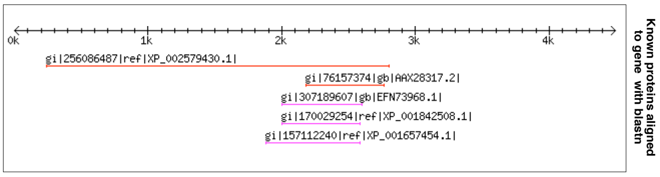

Gene_01715

Supercontigs mapped to this gene
v31.002856-, v31.003811+

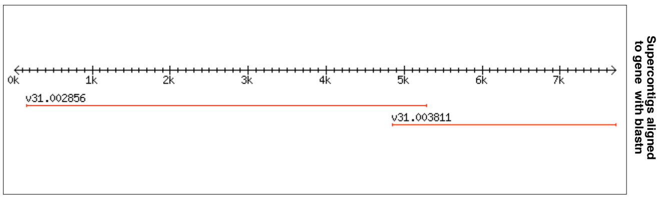

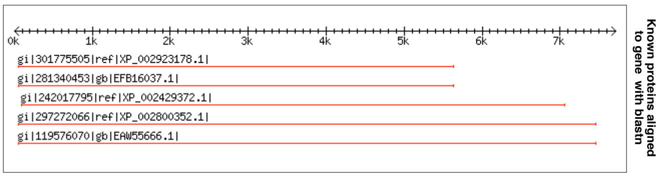

Gene_01751

Supercontigs mapped to this gene
v31.001967+, v31.007040+

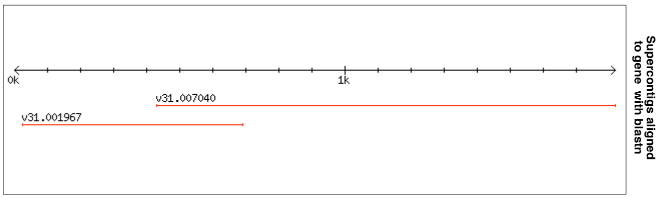

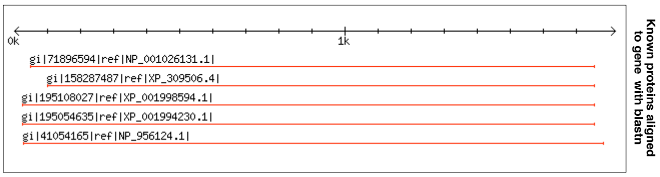

Gene_01783

Supercontigs mapped to this gene
v31.002238+, v31.003662+, v31.015635+

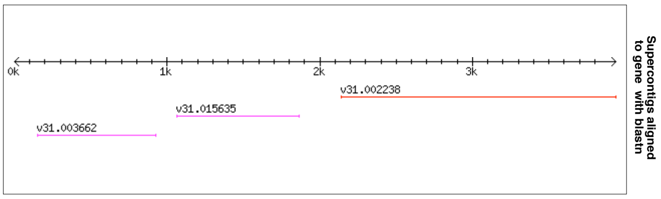

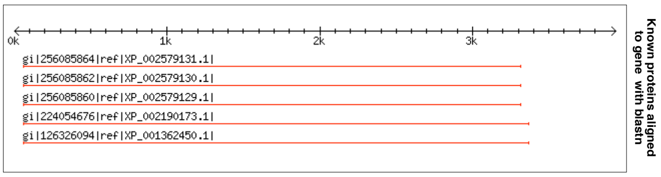

Gene_01813

Supercontigs mapped to this gene
v31.002068-, v31.021301+, v31.032314+

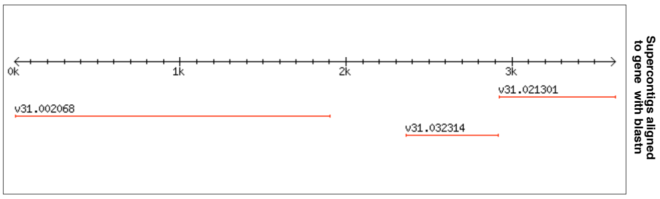

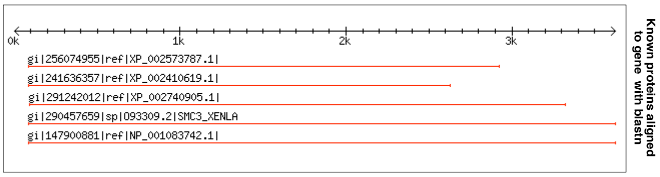

Gene_01812

Supercontigs mapped to this gene
v31.002284-, v31.002458+

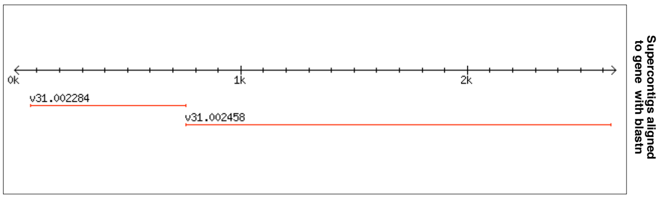

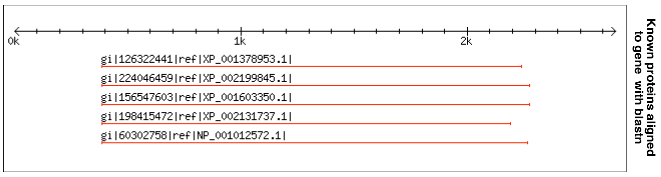

Gene_01814

Supercontigs mapped to this gene
v31.001992-, v31.007626+

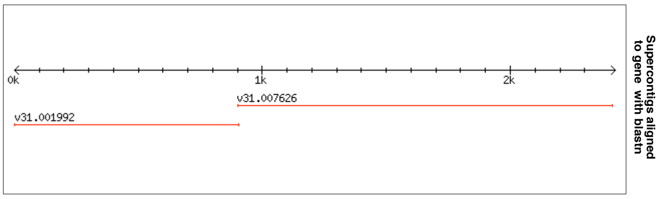

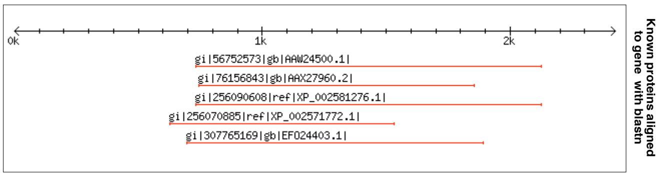

Gene_01823

Supercontigs mapped to this gene

v31.000001-, v31.000310+, v31.000841+, v31.004033+, v31.004421+, v31.008583-, v31.011591+, v31.012273+, v31.020847+, v31.030242-

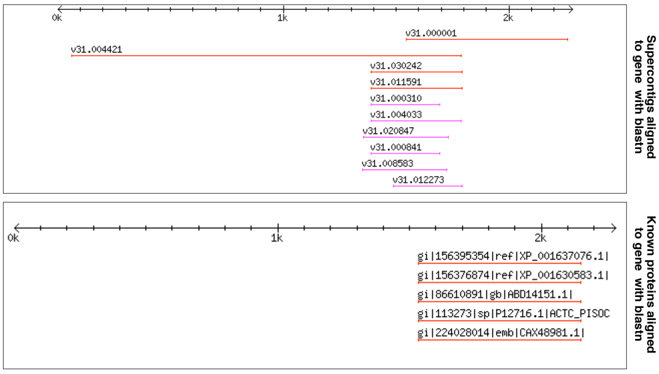

Gene_01845

Supercontigs mapped to this gene

v31.001016-, v31.009630+, v31.013301+

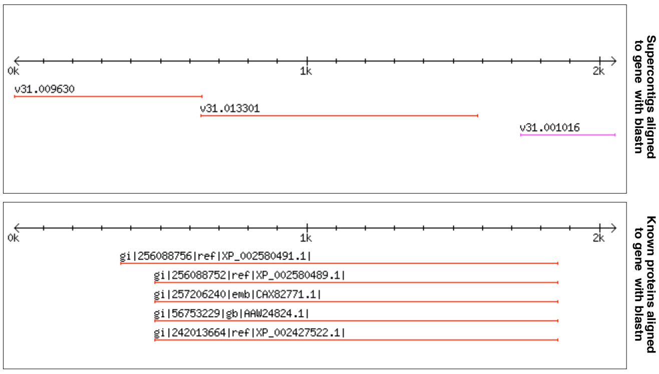

Gene_01840

Supercontigs mapped to this gene

v31.001734-, v31.002813-

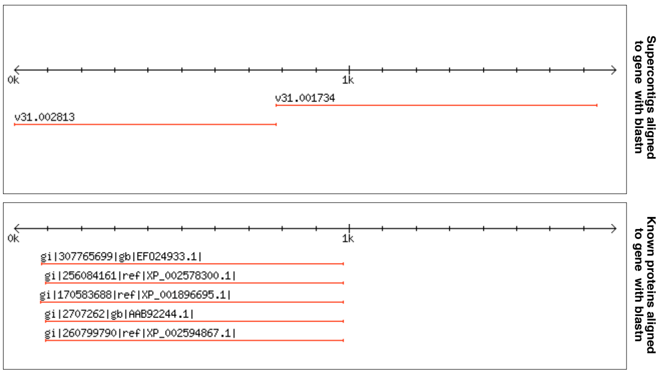

Gene_01856

Supercontigs mapped to this gene

v31.004146+, v31.004252+, v31.011618+, v31.018300+, v31.024981+, v31.026897+, v31.027127+

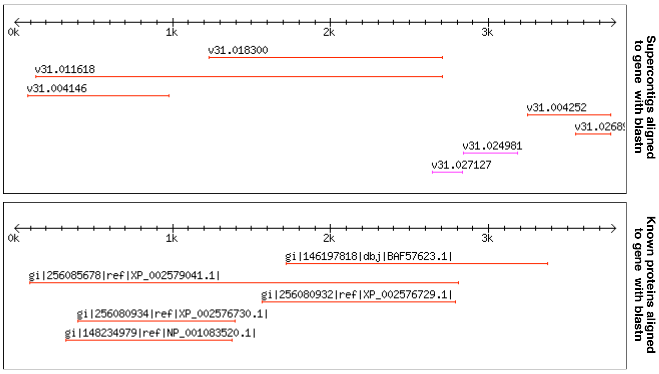

Gene_01878

Supercontigs mapped to this gene

v31.009686-, v31.010928-, v31.014014+, v31.023668-

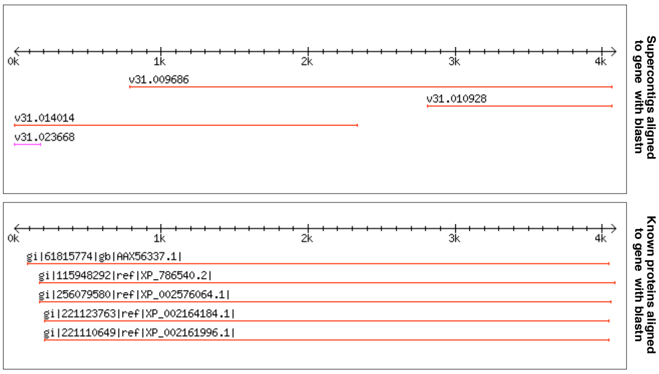

Gene_01903

Supercontigs mapped to this gene

v31.003161-, v31.005338+

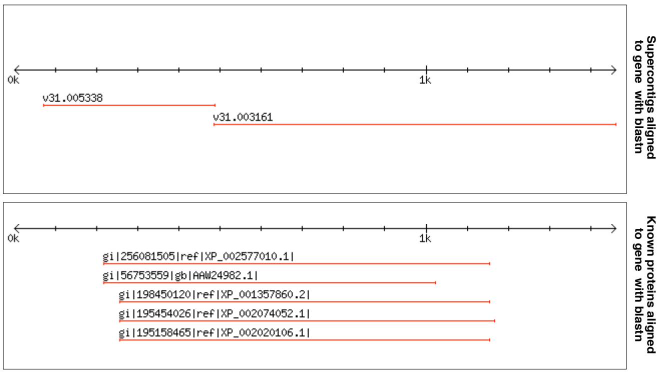

Gene_01891

Supercontigs mapped to this gene

v31.005463+, v31.009269-

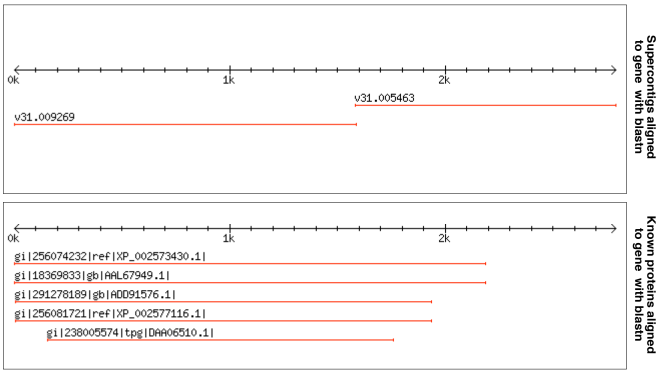

Gene_01909

Supercontigs mapped to this gene

v31.001443+, v31.006889+, v31.007497-, v31.009287+, v31.020008-, v31.024125+, v31.027331-, v31.029152-, v31.045825-

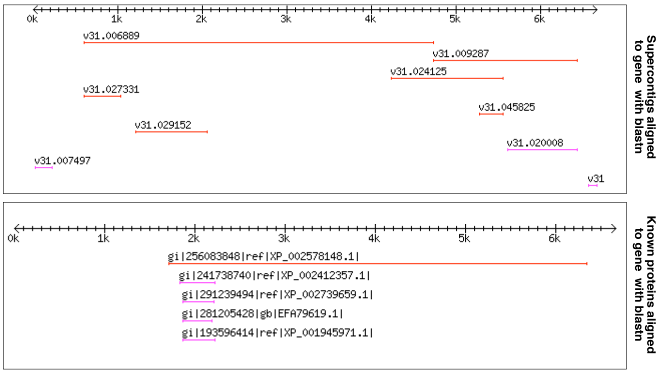

Gene_01919

Supercontigs mapped to this gene

v31.000209-, v31.005498-

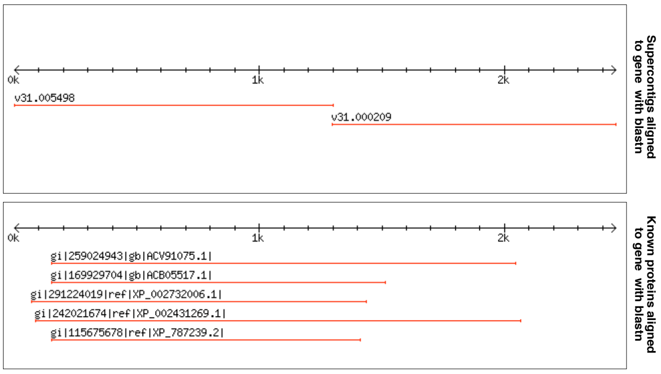

Gene_19815

Supercontigs mapped to this gene

v31.004981+, v31.019898+

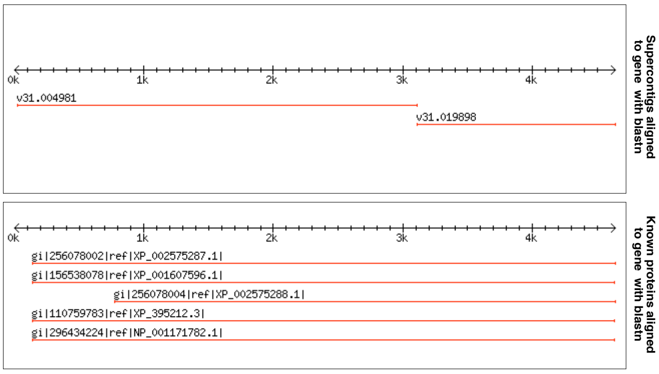

Gene_01979

Supercontigs mapped to this gene

v31.005680-, v31.007607-, v31.018881-

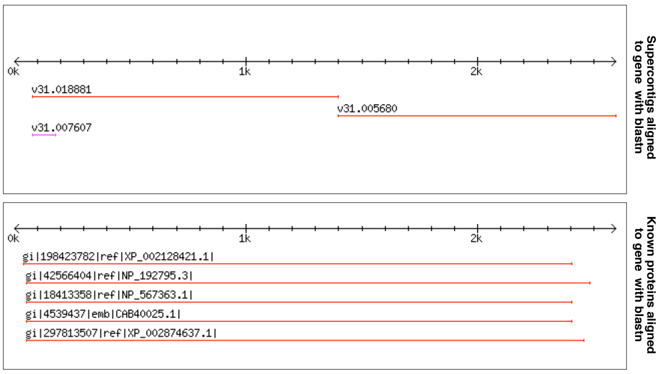

Gene_01983

Supercontigs mapped to this gene

v31.000587+, v31.006634+

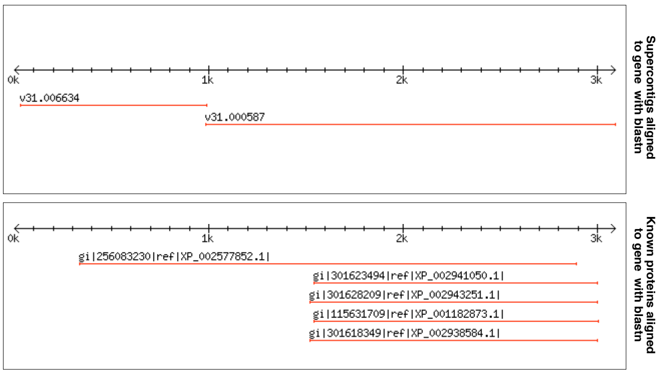

Gene_19831

Supercontigs mapped to this gene

v31.009503-, v31.012347+, v31.013426-, v31.016429+, v31.028824+

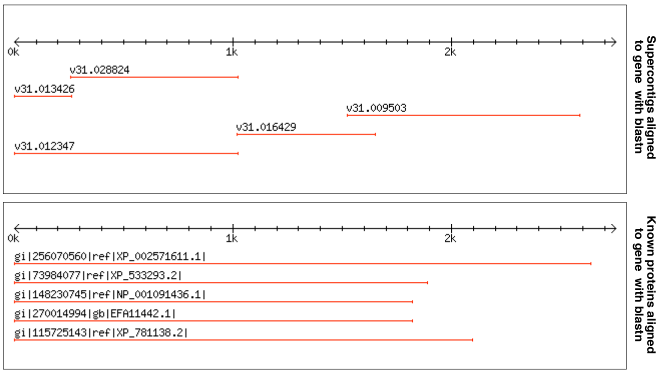

Gene_19847

Supercontigs mapped to this gene

v31.002672+, v31.002809-

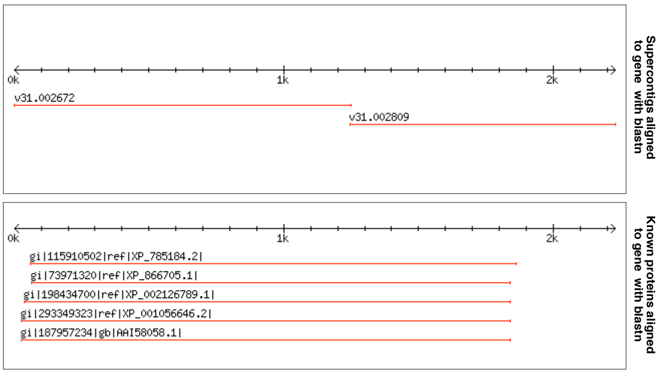

Gene_19838

Supercontigs mapped to this gene

v31.002446-, v31.005570+, v31.010573+, v31.016198+

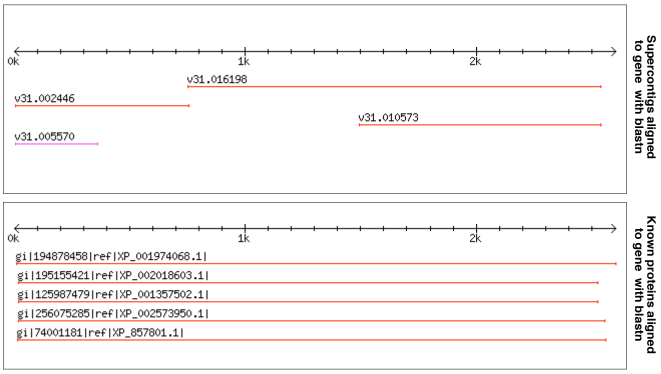

Gene_00202

Supercontigs mapped to this gene

v31.000523-, v31.001395-, v31.003366-, v31.005084+, v31.006259+, v31.007080-, v31.011151-, v31.014118+, v31.016590-, v31.027138+

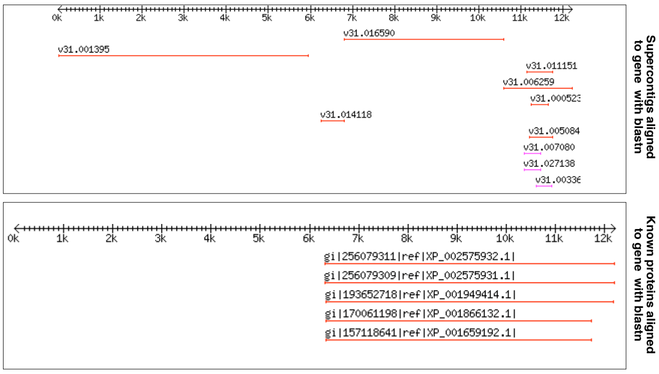

Gene_02075

Supercontigs mapped to this gene

v31.006798+, v31.008450+, v31.020473+, v31.023689+

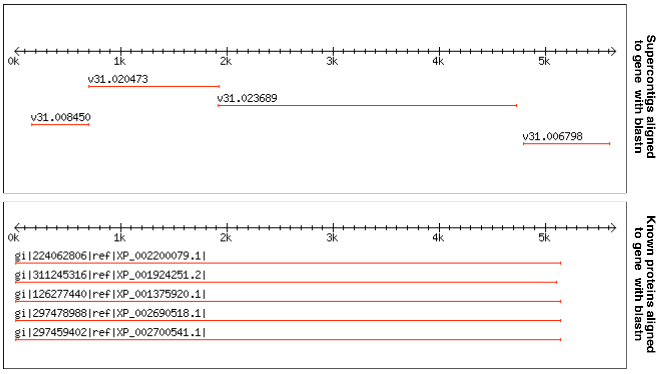

Gene_02095

Supercontigs mapped to this gene

v31.004640+, v31.004843-, v31.013795-, v31.027327-, v31.049703-

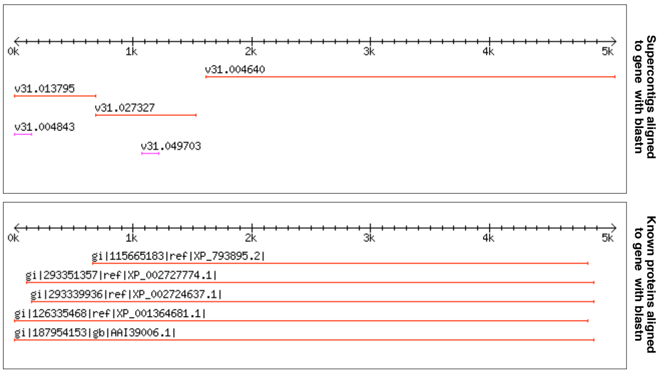

Gene_02079

Supercontigs mapped to this gene

v31.006486-, v31.007069-, v31.009491+, v31.019772+, v31.019784+, v31.019940+, v31.020168-, v31.024044+, v31.024825+, v31.025718-

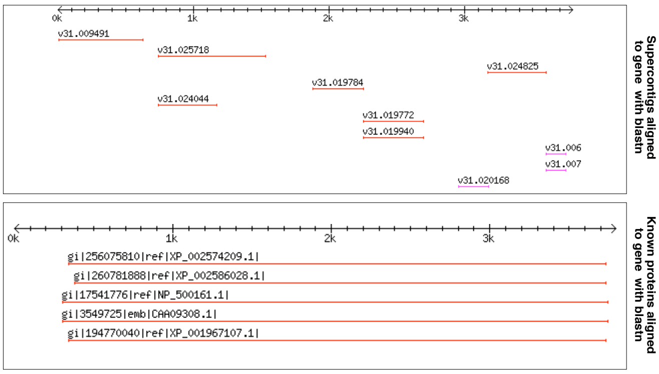

Gene_02099

Supercontigs mapped to this gene

v31.000489+, v31.001543+, v31.012081+, v31.015544-

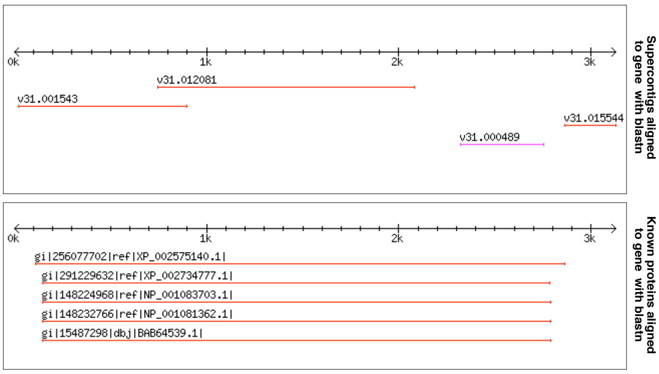

Gene_00212

Supercontigs mapped to this gene

v31.005369+, v31.007345+, v31.012754-, v31.013738-, v31.018463+, v31.039844+

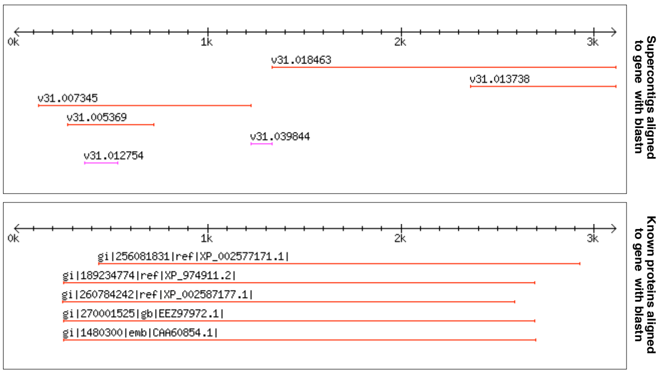

Gene_00213

Supercontigs mapped to this gene

v31.003516+, v31.003672+, v31.003672-, v31.004786+, v31.004938+, v31.010628+, v31.012380+, v31.013247+, v31.016608-, v31.024864+, v31.025617+

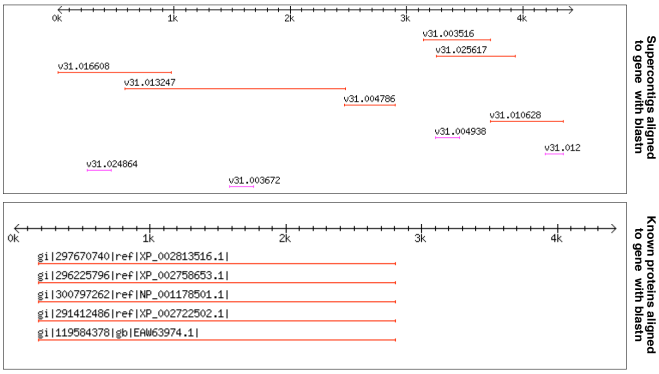

Gene_02122

Supercontigs mapped to this gene

v31.000708+, v31.001172-, v31.001633+, v31.004928+, v31.014335+

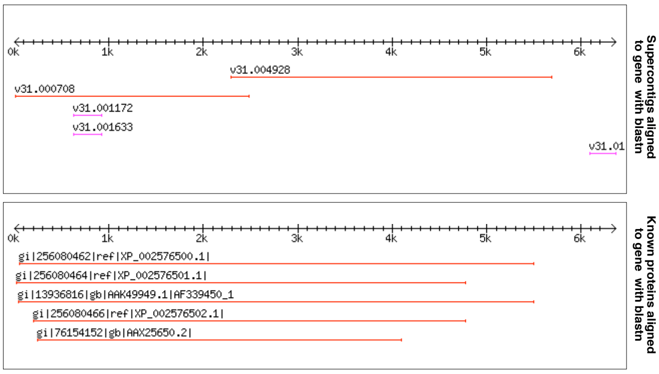

Gene_02157

Supercontigs mapped to this gene

v31.000669-, v31.005136-, v31.009183+

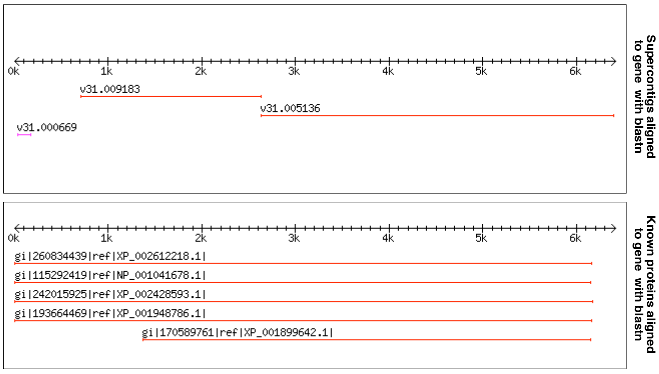

Gene_02166

Supercontigs mapped to this gene

v31.015076-, v31.015460-, v31.059192-

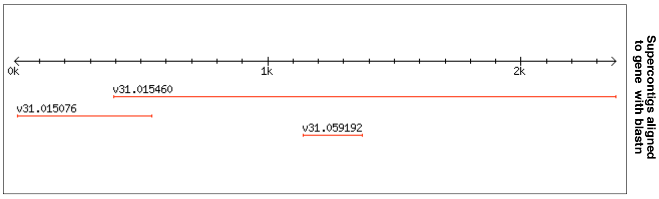

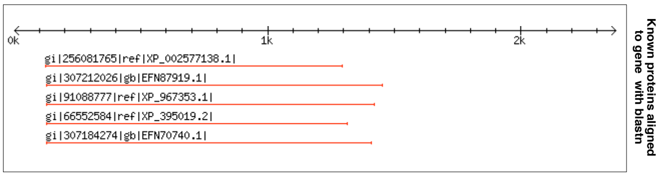

Gene_00222

Supercontigs mapped to this gene

v31.009951+, v31.016785-, v31.021417+, v31.021904+, v31.048246-

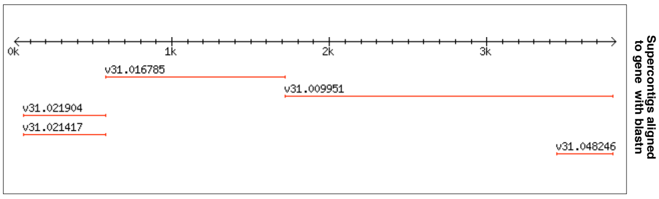

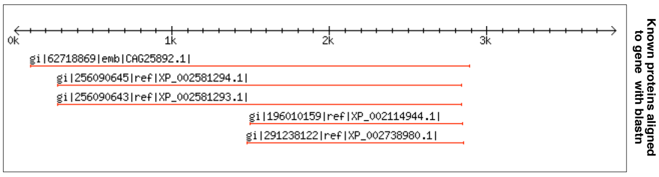

Gene_02209

Supercontigs mapped to this gene

v31.008929+, v31.013869+, v31.013974+, v31.025071+

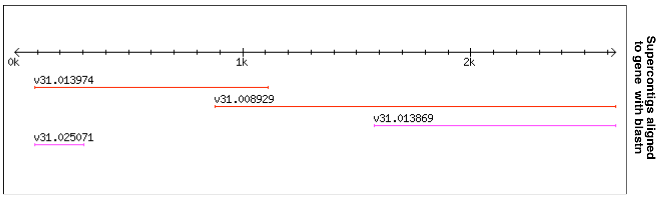

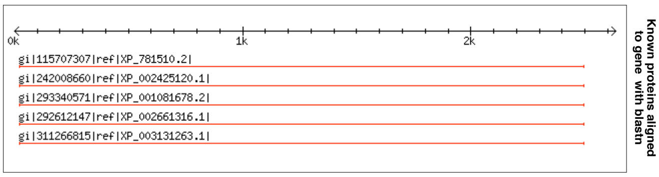

Gene_02229

Supercontigs mapped to this gene

v31.000294-, v31.007073+, v31.011020-, v31.023625-

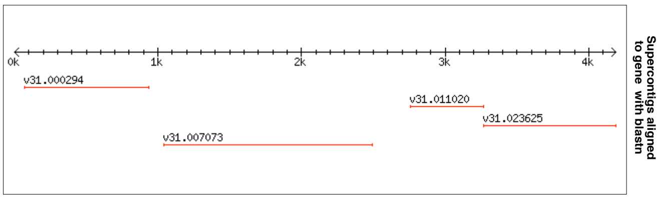

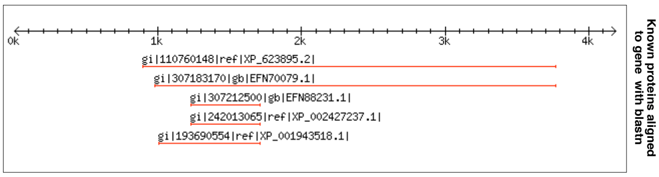

Gene_02238

Supercontigs mapped to this gene

v31.014372-, v31.015707-, v31.021827-, v31.026665-

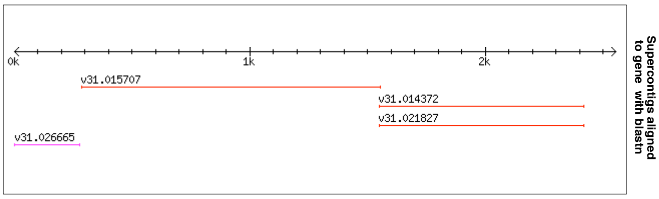

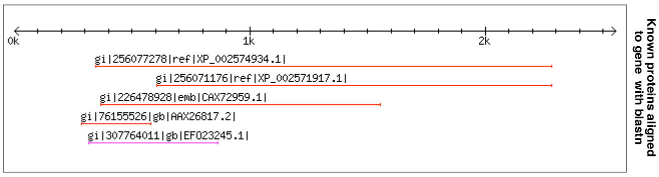

Gene_02299

Supercontigs mapped to this gene

v31.001786-, v31.004686-

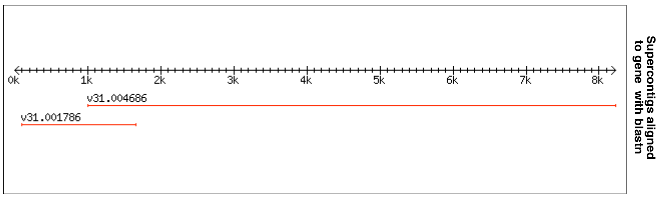

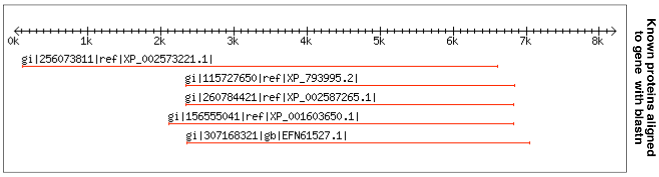

Gene_02287

Supercontigs mapped to this gene

v31.002315-, v31.013034+, v31.014059+, v31.029439-, v31.053417-

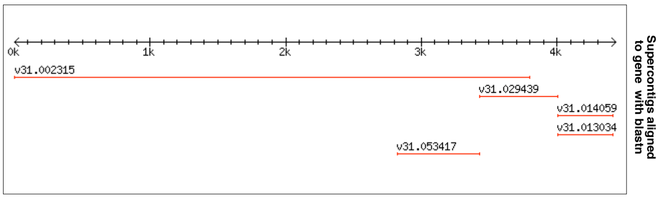

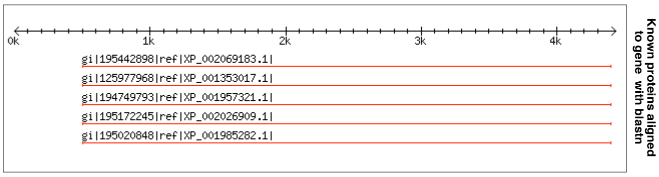

Gene_02318

Supercontigs mapped to this gene

v31.001646-, v31.014057-, v31.021086-, v31.026209-, v31.027892+

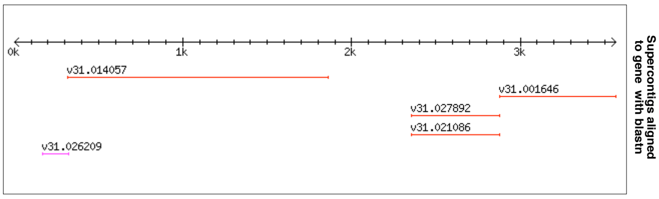

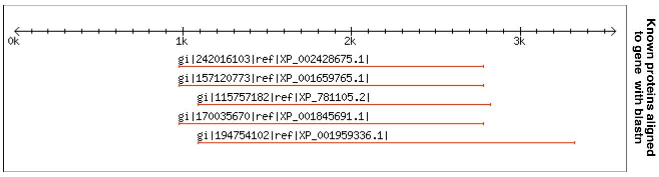

Gene_02322

Supercontigs mapped to this gene

v31.003498-, v31.006706+, v31.017918+

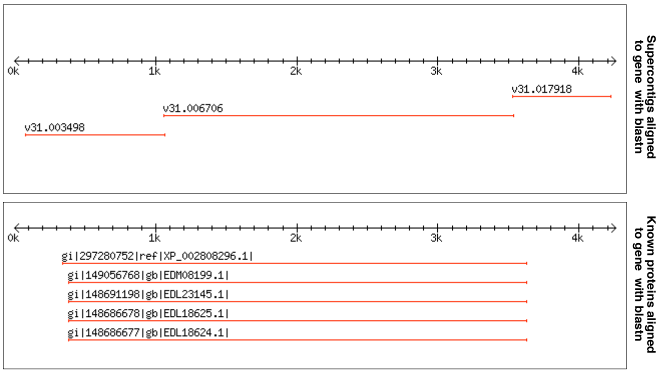

Gene_02329

Supercontigs mapped to this gene

v31.004846-, v31.008652-, v31.011406-, v31.033754-, v31.039038-

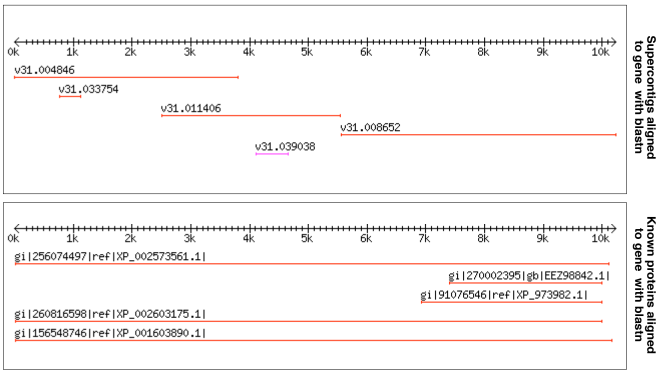

Gene_02375

Supercontigs mapped to this gene

v31.000550+, v31.003969-

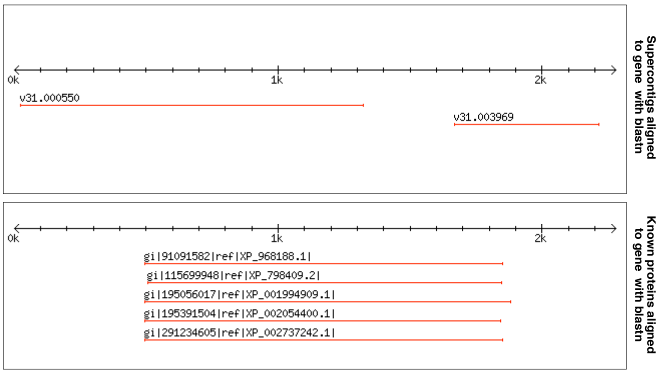

Gene_02381

Supercontigs mapped to this gene

v31.001852-, v31.003060-

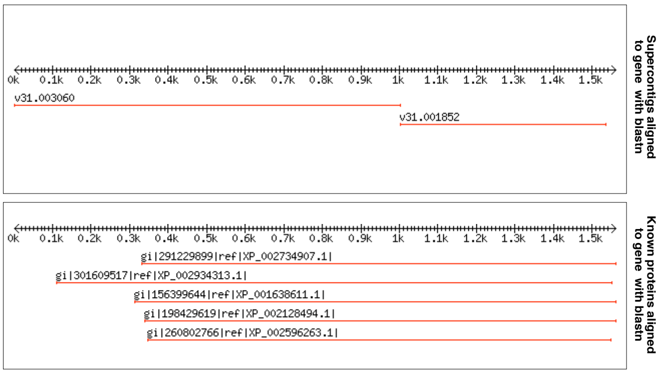

Gene_02351

Supercontigs mapped to this gene

v31.005786+, v31.008306-, v31.010089-

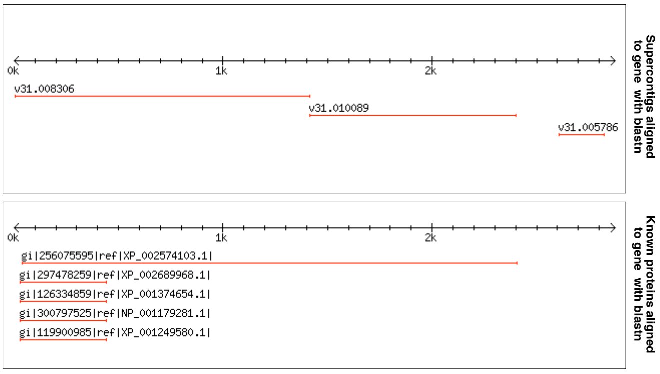

Gene_02368

Supercontigs mapped to this gene

v31.001006-, v31.002558-

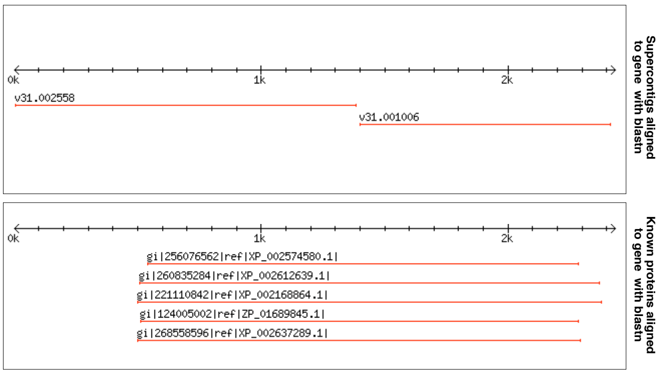

Gene_02382

Supercontigs mapped to this gene

v31.002341-, v31.005819-

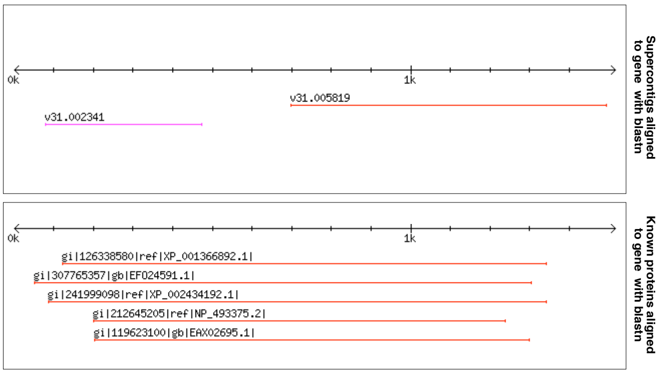

Gene_02387

Supercontigs mapped to this gene

v31.001752+, v31.006273+, v31.009732+, v31.019066-

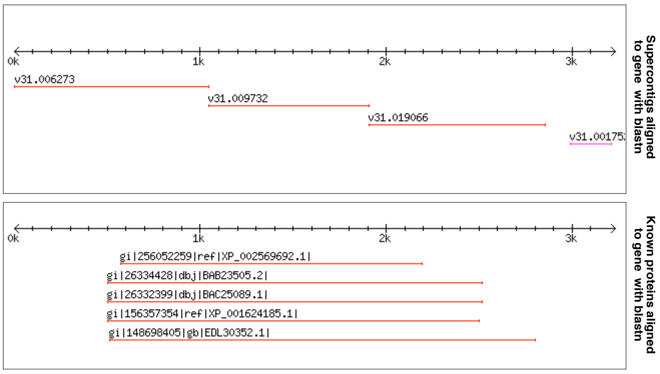

Gene_02396

Supercontigs mapped to this gene

v31.000344+, v31.008323+

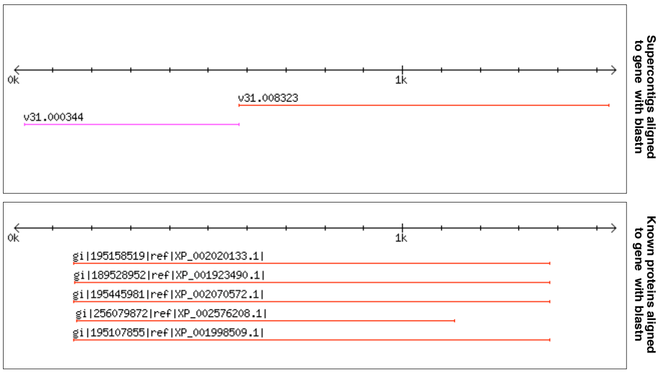

Gene_02411

Supercontigs mapped to this gene

v31.000649-, v31.000768+, v31.014044+

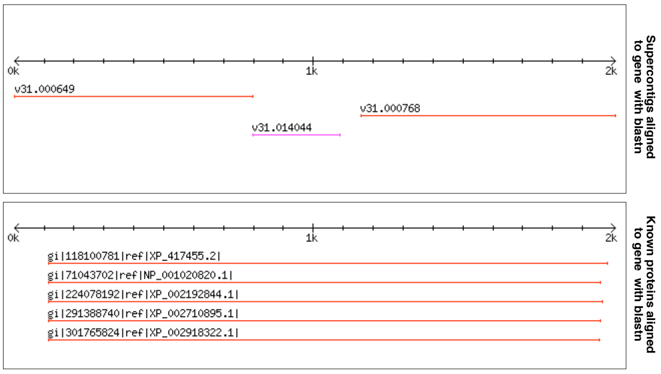

Gene_02406

Supercontigs mapped to this gene

v31.000160-, v31.001839-, v31.015373+, v31.037618-

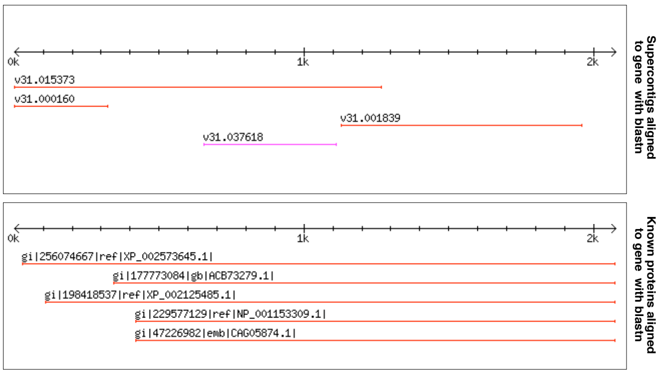

Gene_02424

Supercontigs mapped to this gene

v31.002948+, v31.010473-, v31.028097-

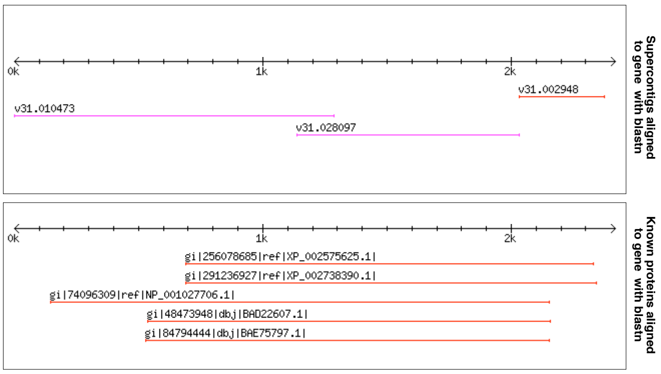

Gene_02445

Supercontigs mapped to this gene

v31.005673-, v31.007341+, v31.007636-

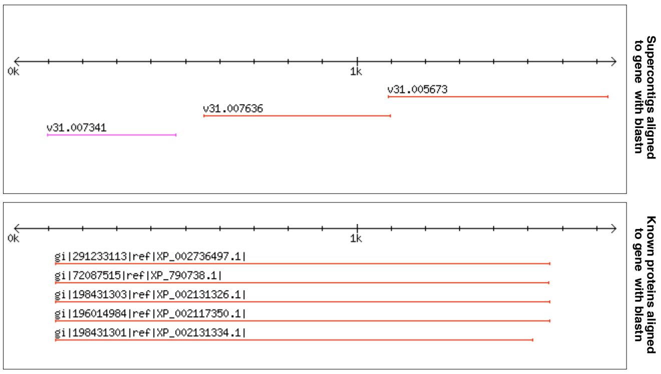

Gene_02487

Supercontigs mapped to this gene

v31.000673-, v31.001018-, v31.006557+

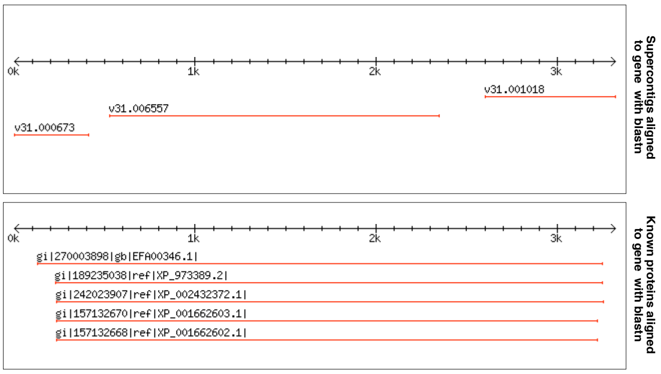

Gene_02484

Supercontigs mapped to this gene

v31.002331-, v31.004675-, v31.028353+

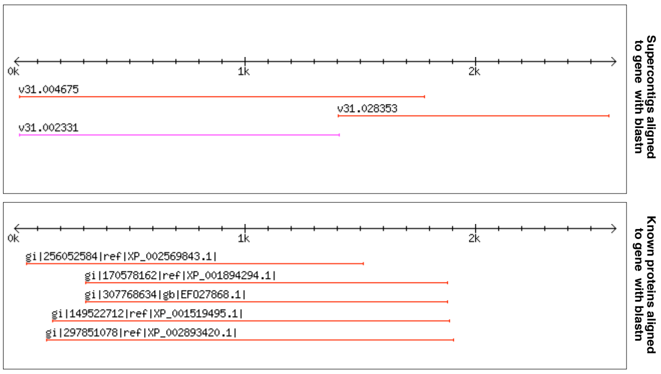

Gene_02502

Supercontigs mapped to this gene

v31.000067+, v31.003200-

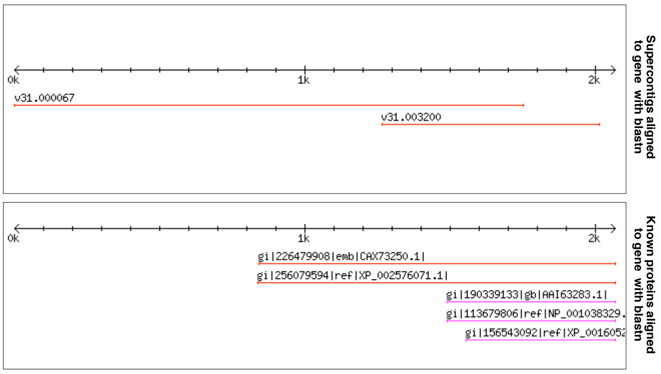

Gene_02508

Supercontigs mapped to this gene

v31.010521-, v31.010529+, v31.011184+, v31.013465+, v31.015240+, v31.023752-, v31.025329-, v31.027388-, v31.028013+, v31.044102-

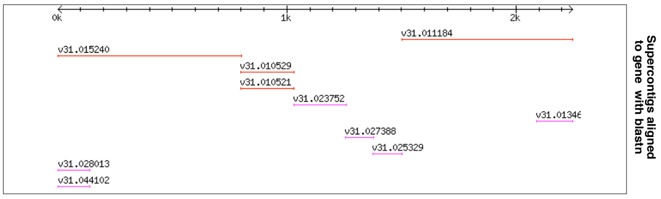

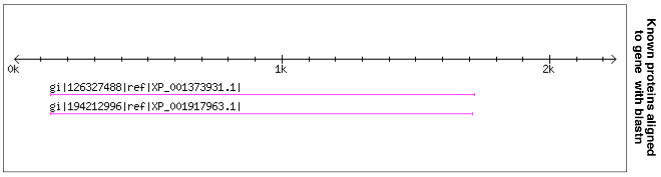

Gene_02541

Supercontigs mapped to this gene

v31.000326+, v31.003323-

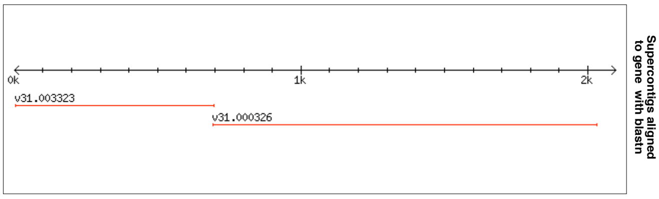

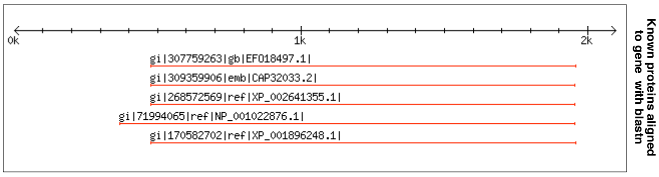

Gene_02521

Supercontigs mapped to this gene

v31.002801-, v31.008535-, v31.013588+, v31.019981+

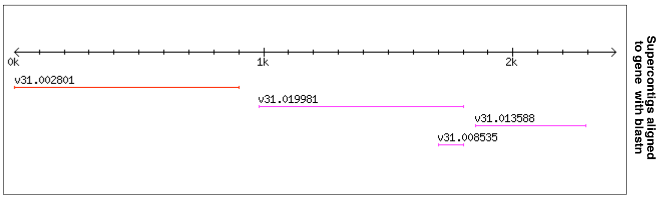

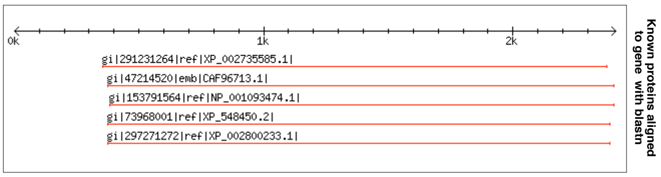

Gene_00255

Supercontigs mapped to this gene

v31.010650-, v31.011402-, v31.035446+

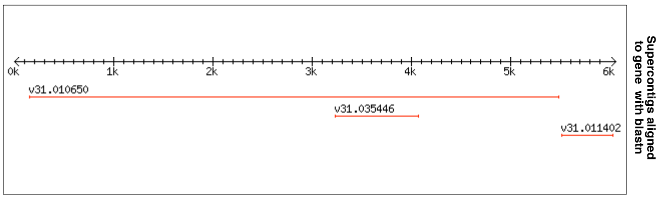

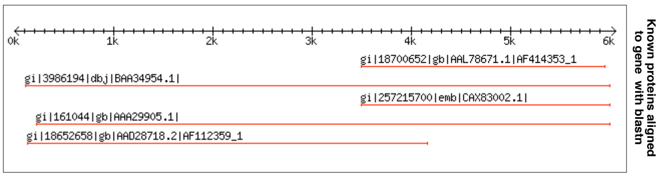

Gene_02596

Supercontigs mapped to this gene

v31.007317-, v31.014108+, v31.020911-

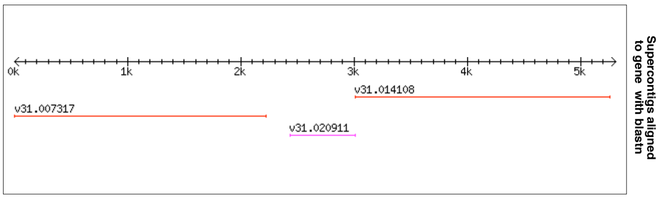

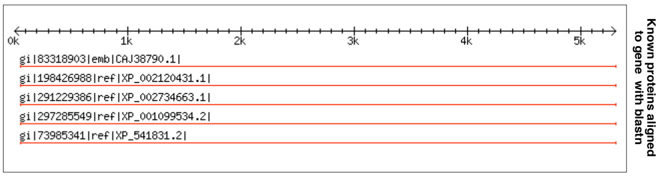

Gene_00264

Supercontigs mapped to this gene

v31.001747-, v31.004747+, v31.017494+

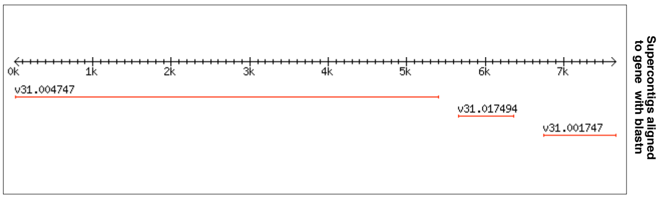

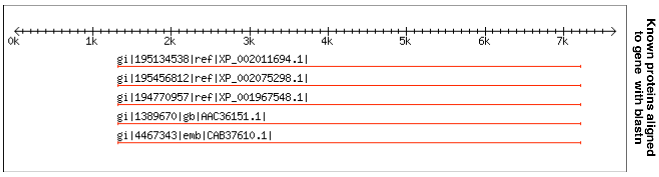

Gene_02607

Supercontigs mapped to this gene

v31.000444-, v31.008809+, v31.014883-

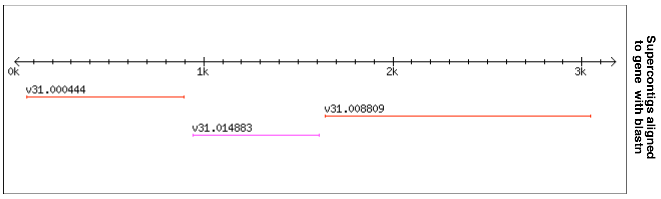

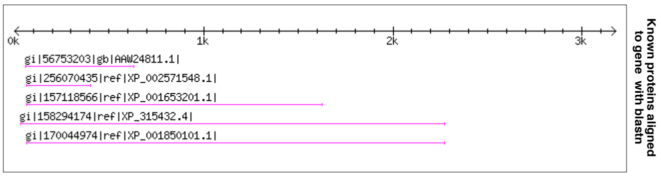

Gene_02644

Supercontigs mapped to this gene

v31.022631-, v31.023120+, v31.023210+, v31.023396-, v31.023630+, v31.024643-, v31.025195+, v31.026228+, v31.027077+, v31.028183-

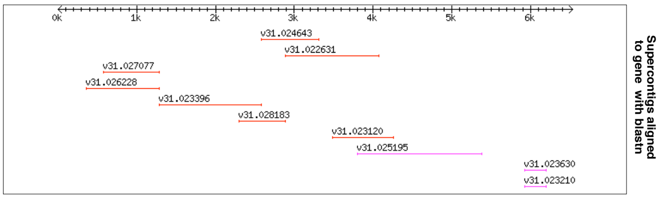

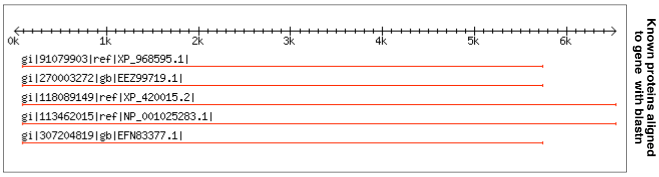

Gene_02649

Supercontigs mapped to this gene
v31.000009-, v31.006092+

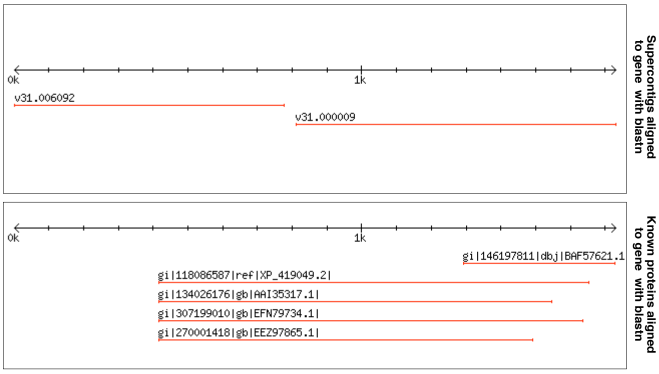

Gene_02769

Supercontigs mapped to this gene
v31.010197+, v31.012430-, v31.014607+, v31.028673+

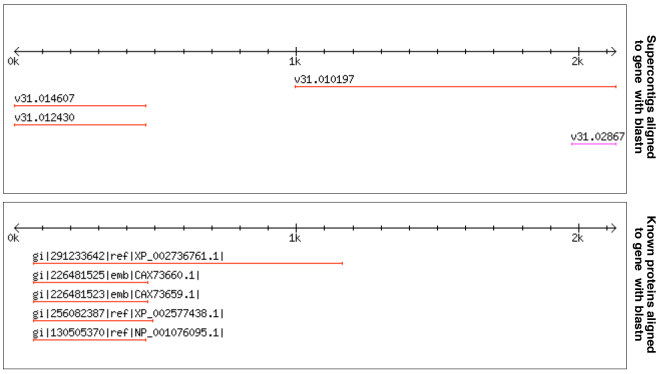

Gene_02691

Supercontigs mapped to this gene
v31.001111-, v31.006739+, v31.017073+

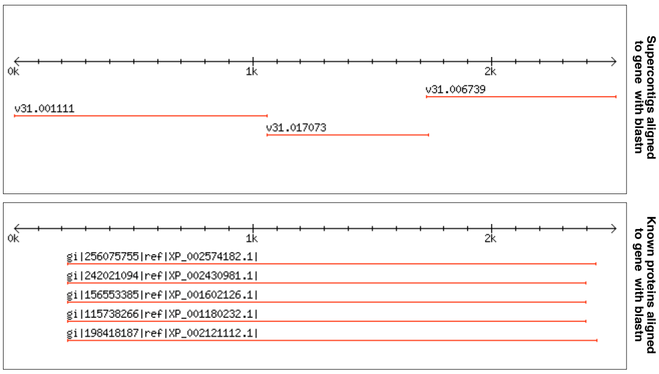

Gene_02770

Supercontigs mapped to this gene
v31.002777-, v31.005582+, v31.013902-, v31.021089-

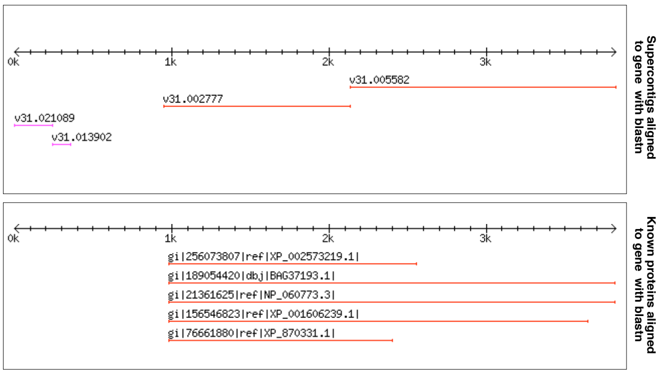

Gene_02773

Supercontigs mapped to this gene
v31.000250-, v31.002109-

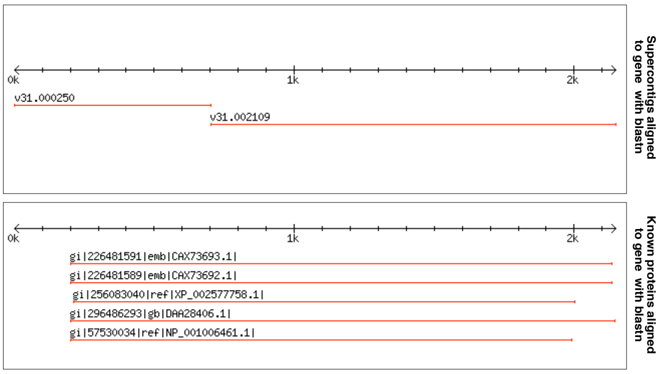

Gene_02826

Supercontigs mapped to this gene
v31.005749+, v31.009085+, v31.021165-

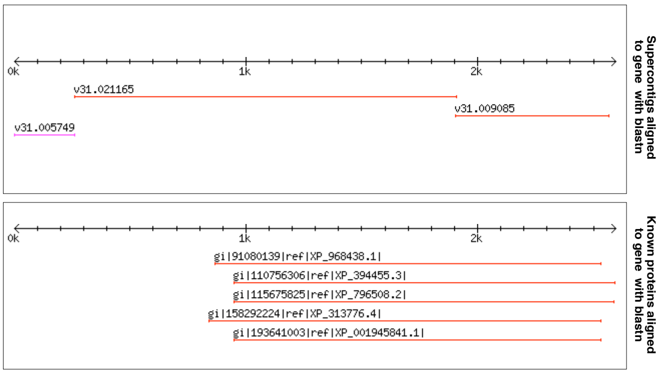

Gene_02815

Supercontigs mapped to this gene
v31.007438-, v31.014520-, v31.016387-, v31.019873+, v31.020819+, v31.027050-

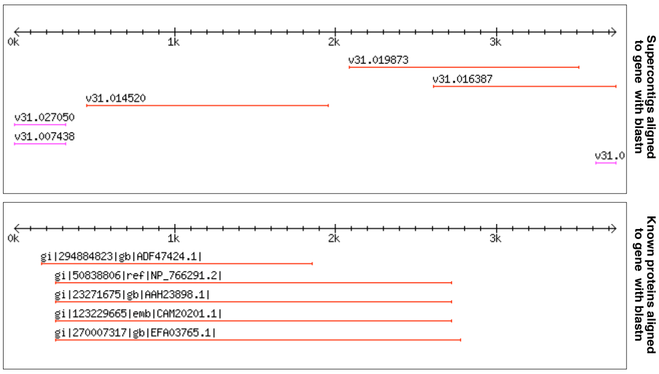

Gene_02965

Supercontigs mapped to this gene
v31.002694+, v31.016247+, v31.016738+, v31.019963+, v31.020640+, v31.022430+, v31.022738-, v31.028598+, v31.038473-, v31.040241-

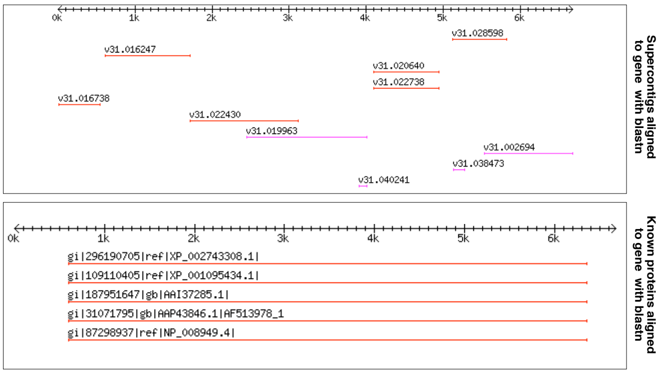

Gene_02990

Supercontigs mapped to this gene

v31.005104-, v31.006357-, v31.013048+

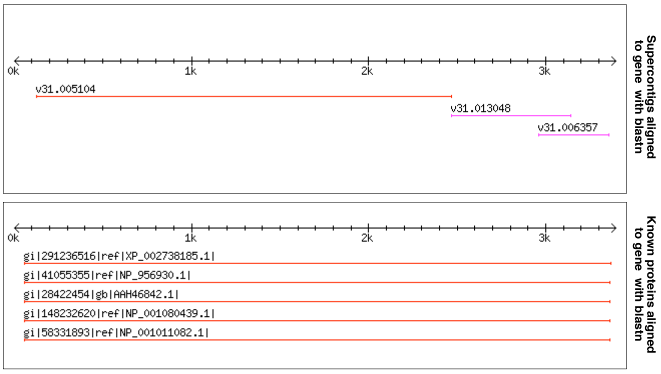

Gene_03089

Supercontigs mapped to this gene

v31.006628-, v31.010023+, v31.019282-, v31.020642-, v31.025554-, v31.026739+

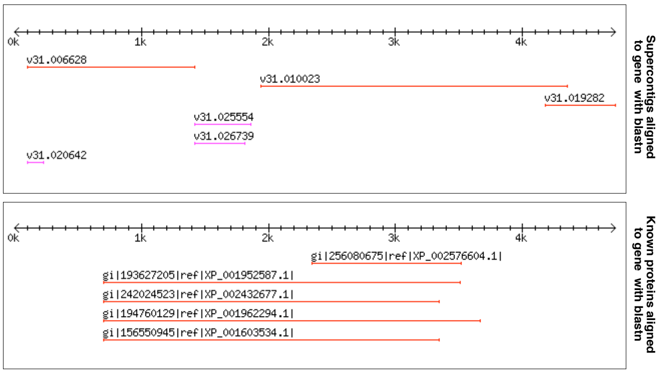

Gene_03074

Supercontigs mapped to this gene

v31.002394-, v31.005819-

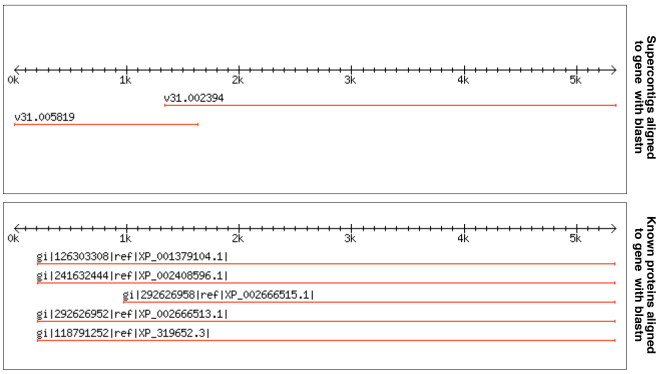

Gene_03097

Supercontigs mapped to this gene

v31.014905+, v31.017901+, v31.020241-, v31.027744-

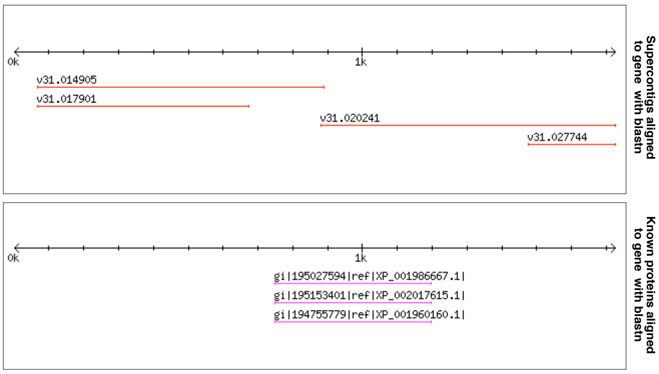

Gene_00031

Supercontigs mapped to this gene

v31.002855-, v31.004045-

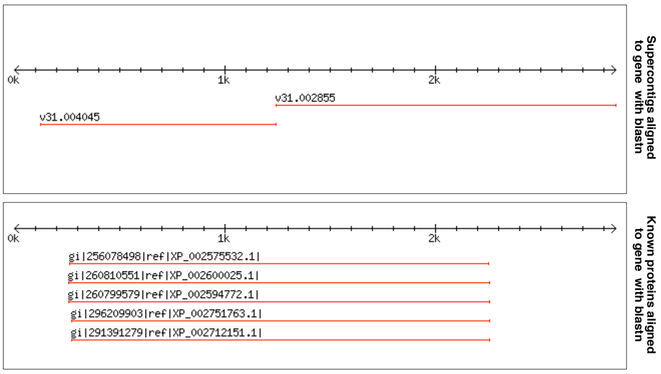

Gene_03108

Supercontigs mapped to this gene

v31.003173-, v31.006034-, v31.006604-, v31.011090+, v31.048660-

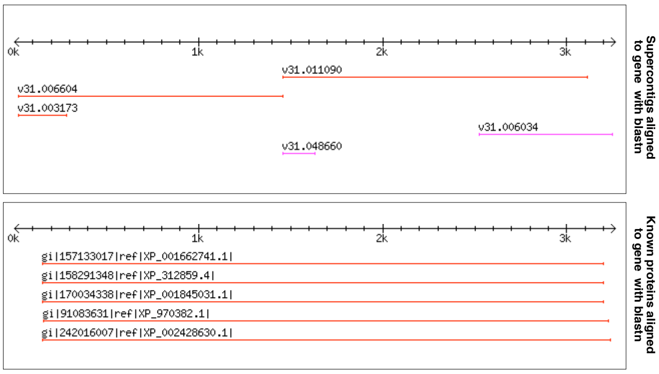

Gene_03102

Supercontigs mapped to this gene

v31.003622+, v31.006413+

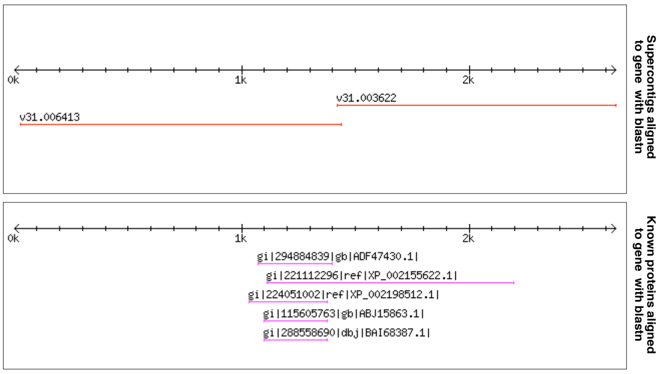

Gene_03117

Supercontigs mapped to this gene

v31.013707+, v31.015023-

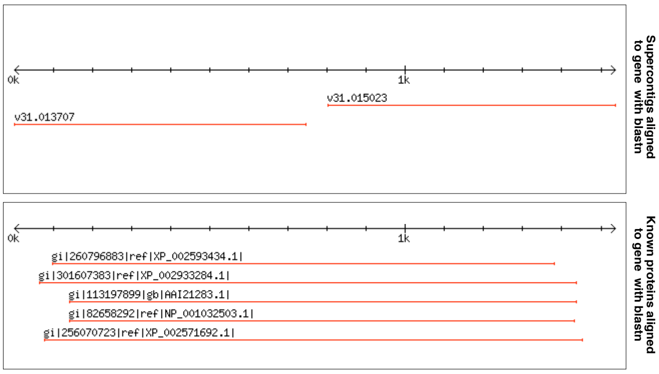

Gene_03122

Supercontigs mapped to this gene

v31.010718+, v31.013656-, v31.019705-, v31.021472-, v31.026004+

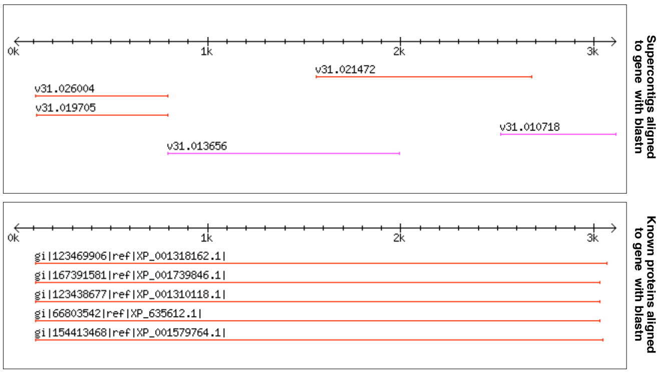

Gene_03196

Supercontigs mapped to this gene

v31.007906-, v31.008190+, v31.010045-, v31.014834-, v31.019064+, v31.019431+, v31.021691-

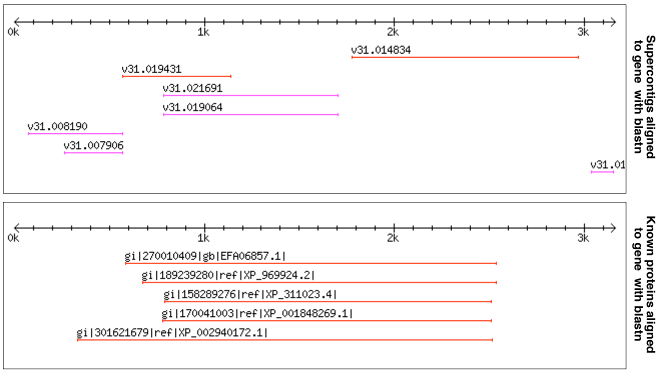

Gene_03141

Supercontigs mapped to this gene

v31.000079-, v31.000302+, v31.000374+, v31.000541-, v31.000824+, v31.001027+, v31.003082+, v31.003087+, v31.020071-, v31.033436+

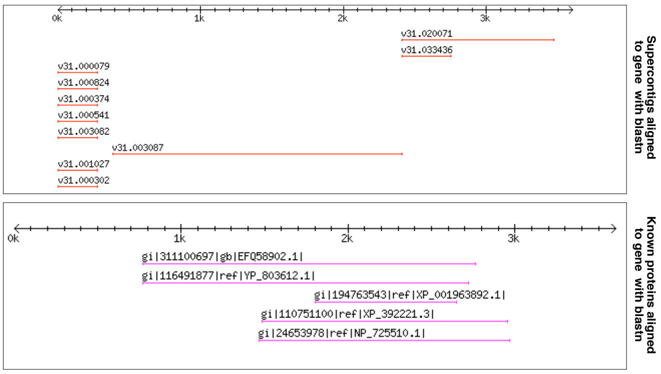

Gene_03207

Supercontigs mapped to this gene

v31.000422-, v31.006467+, v31.008090+, v31.008090-

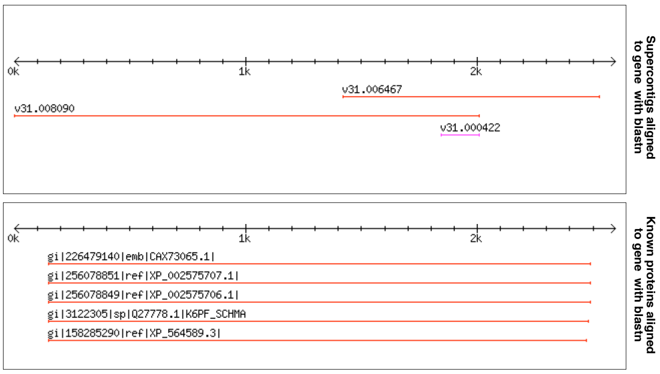

Gene_03213

Supercontigs mapped to this gene

v31.000593-, v31.004735+, v31.010251-, v31.015962-

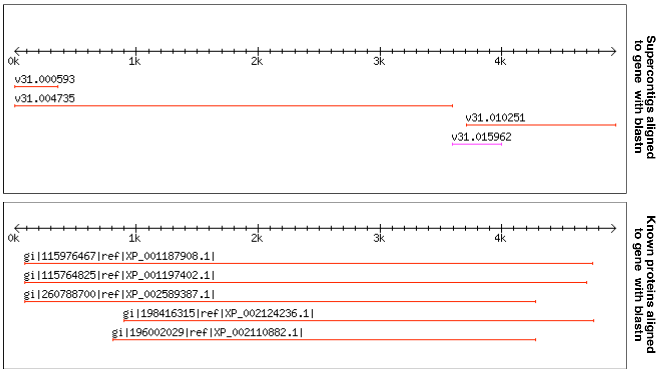

Gene_03260

Supercontigs mapped to this gene

v31.000051-, v31.007172+, v31.025264+

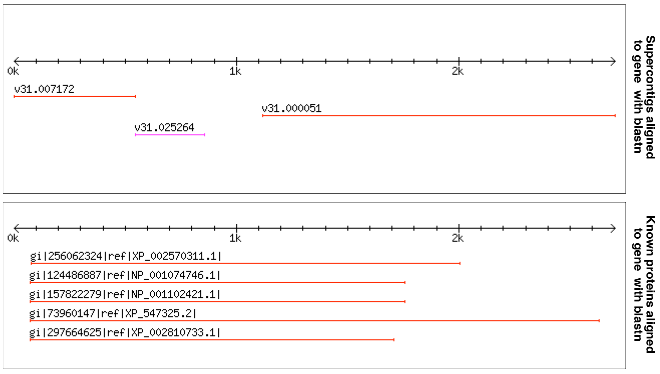

Gene_00322

Supercontigs mapped to this gene

v31.003969-, v31.006753+

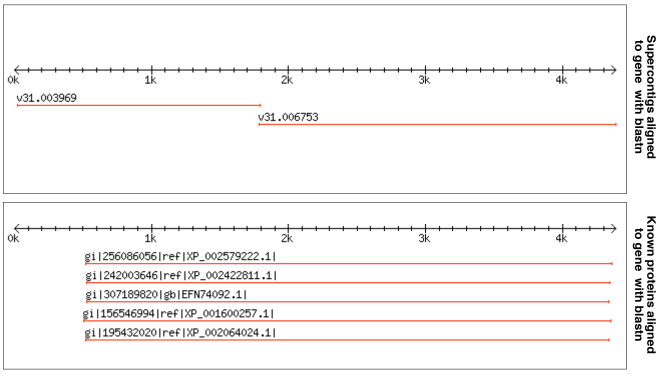

Gene_03268

Supercontigs mapped to this gene

v31.000656-, v31.007060+, v31.008160-

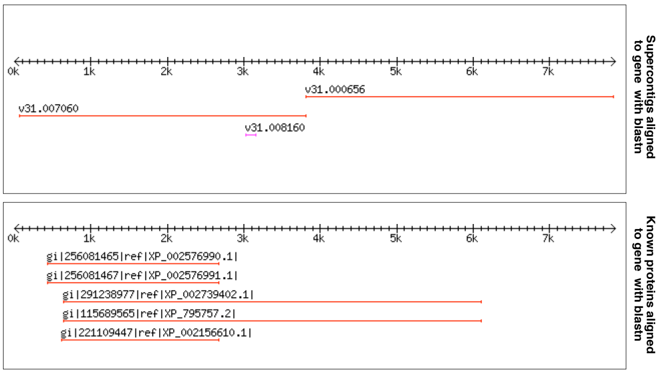

Gene_03279

Supercontigs mapped to this gene

v31.005350-, v31.010495+

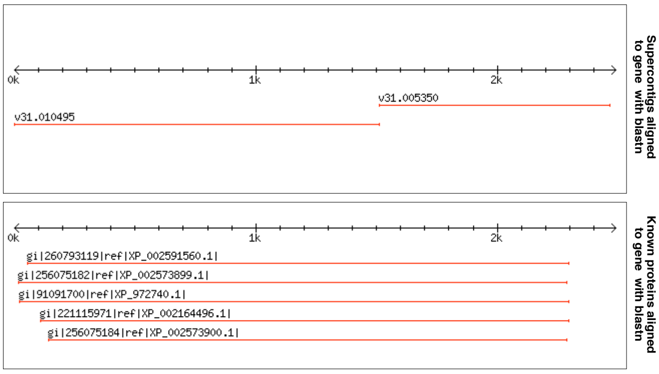

Gene_03293

Supercontigs mapped to this gene

v31.001526-, v31.006045-, v31.026118+, v31.040938-

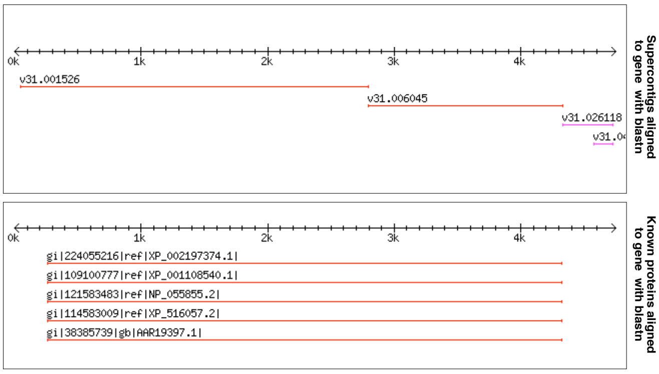

Gene_03284

Supercontigs mapped to this gene

v31.008370+, v31.011712+, v31.015369-

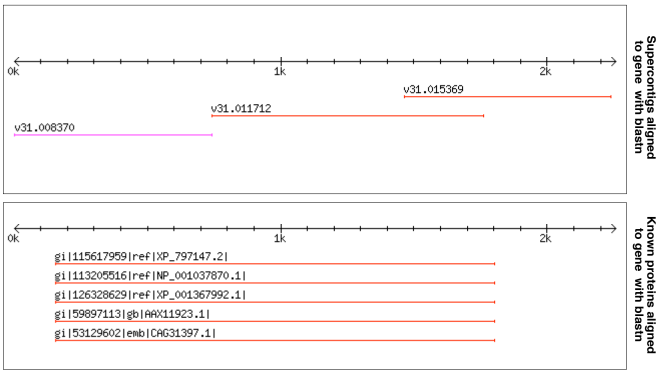

Gene_03304

Supercontigs mapped to this gene

v31.003430-, v31.006306+, v31.009702+, v31.010742+, v31.013177-, v31.016463-, v31.016703-, v31.017229+, v31.029701+, v31.031400+

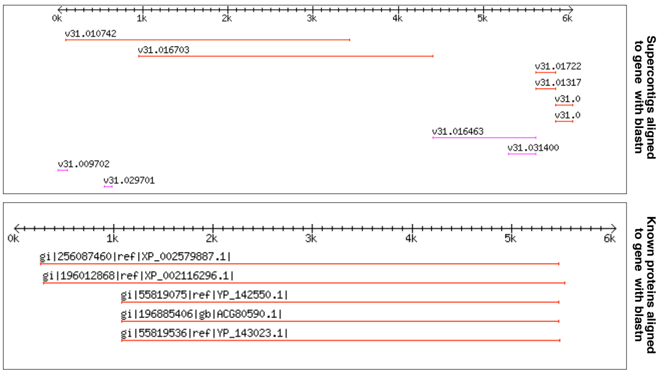

Gene_03305

Supercontigs mapped to this gene

v31.014045-, v31.021288+, v31.021359-, v31.025671+, v31.033799-, v31.036567-

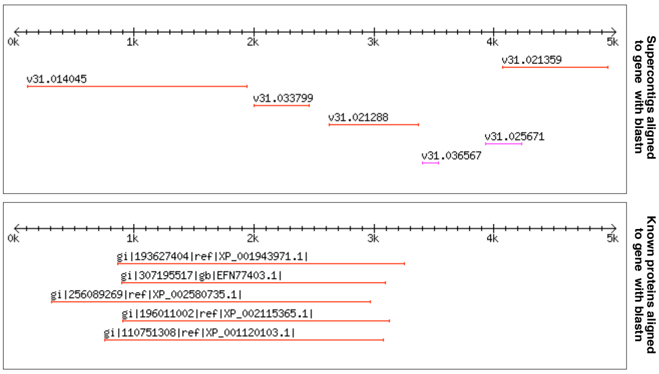

Gene_00339

Supercontigs mapped to this gene

v31.020341-, v31.025446+, v31.058050+

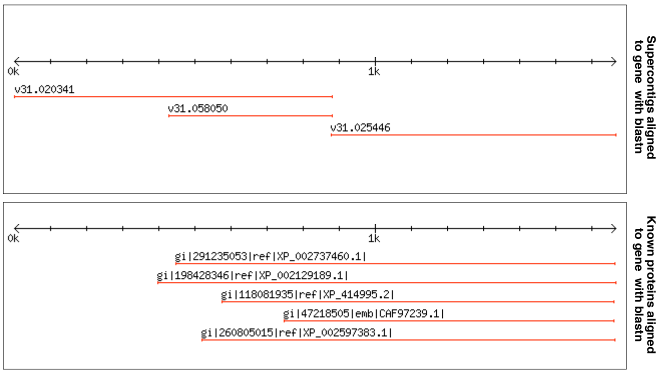

Gene_03355

Supercontigs mapped to this gene

v31.013549-, v31.014535-, v31.016531+, v31.023345+

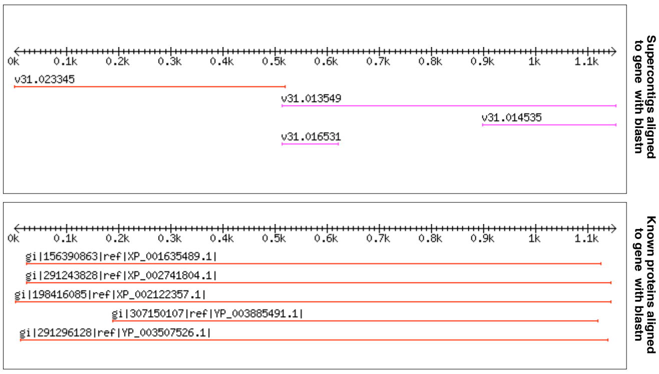

Gene_03399

Supercontigs mapped to this gene

v31.002792+, v31.006394-, v31.010555-, v31.021008-, v31.041194-

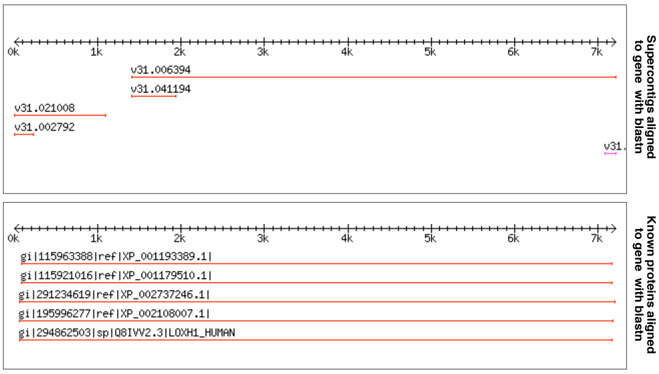

Gene_03467

Supercontigs mapped to this gene

v31.009256-, v31.011238+

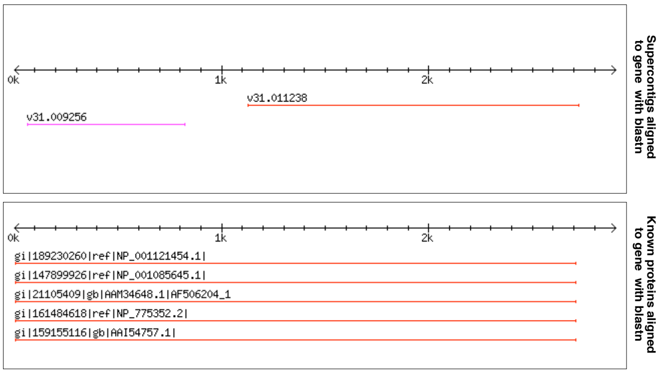

Gene_03490

Supercontigs mapped to this gene

v31.002749-, v31.003678-, v31.039239+

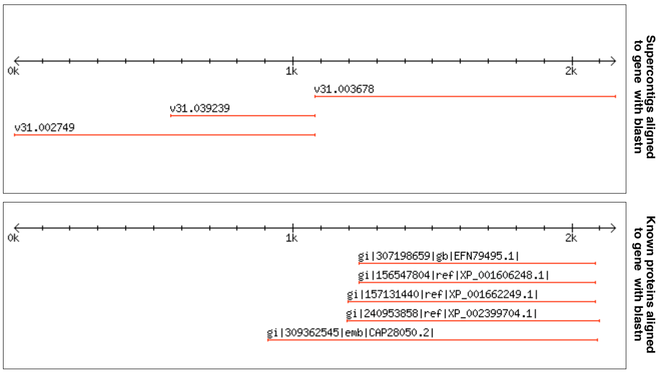

Gene_00347

Supercontigs mapped to this gene

v31.002893-, v31.013349-

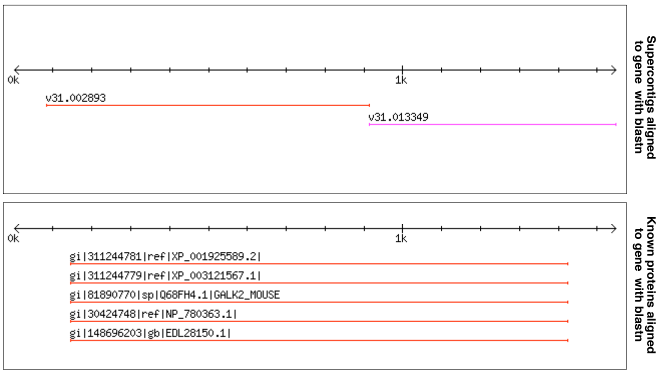

Gene_03509

Supercontigs mapped to this gene

v31.011930-, v31.016680-, v31.021943-, v31.022034+, v31.023648+

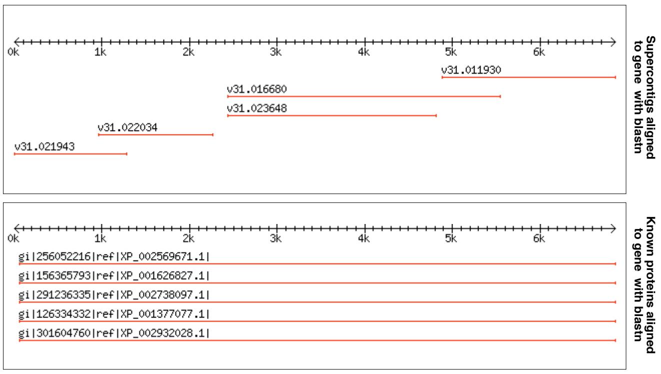

Gene_00355

Supercontigs mapped to this gene

v31.001623+, v31.012174-, v31.017197+

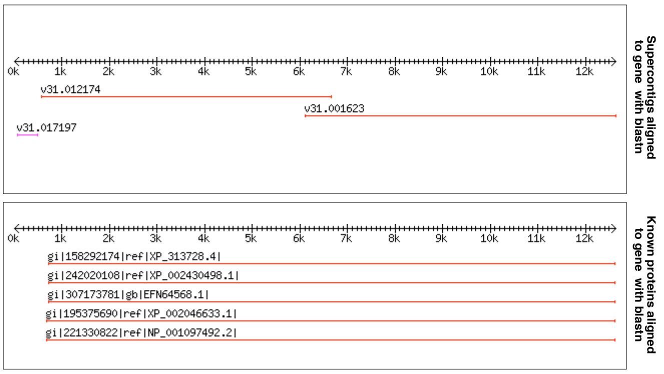

Gene_03559

Supercontigs mapped to this gene

v31.001504-, v31.019597-

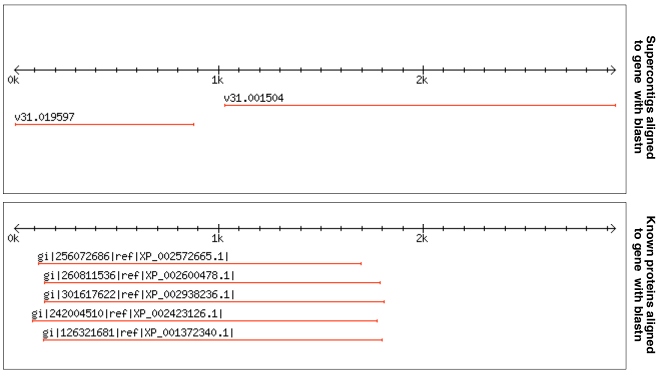

Gene_03556

Supercontigs mapped to this gene

v31.003702-, v31.004920-

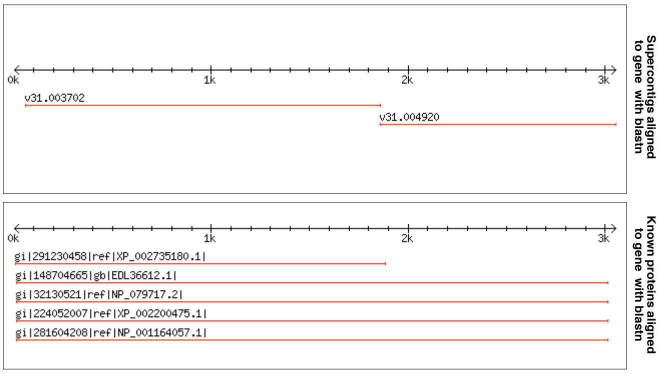

Gene_00358

Supercontigs mapped to this gene

v31.003049+, v31.003354-

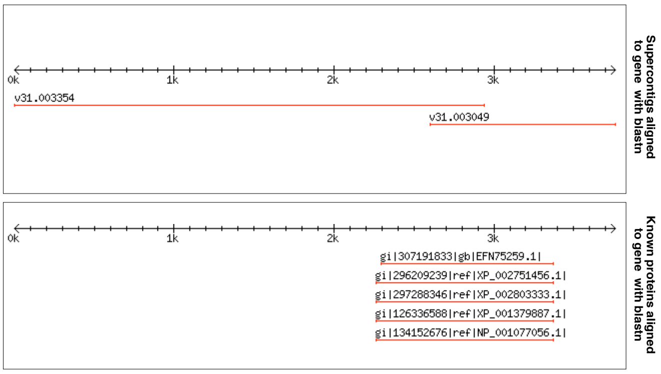

Gene_03589

Supercontigs mapped to this gene

v31.004803-, v31.006727-, v31.011254+

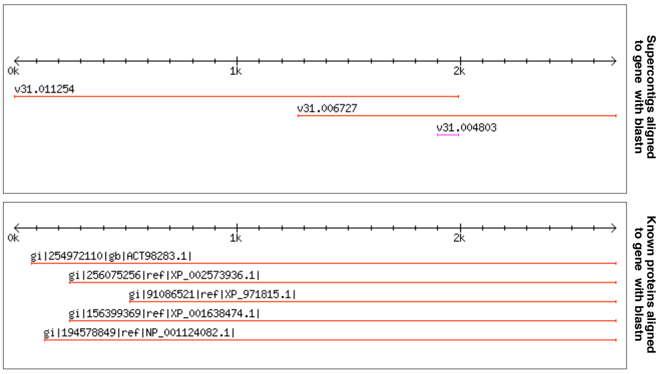

Gene_03617

Supercontigs mapped to this gene

v31.011930-, v31.013460+, v31.014443+, v31.020158-, v31.024407+, v31.026626+, v31.026699+, v31.032851+, v31.039381-, v31.050697-

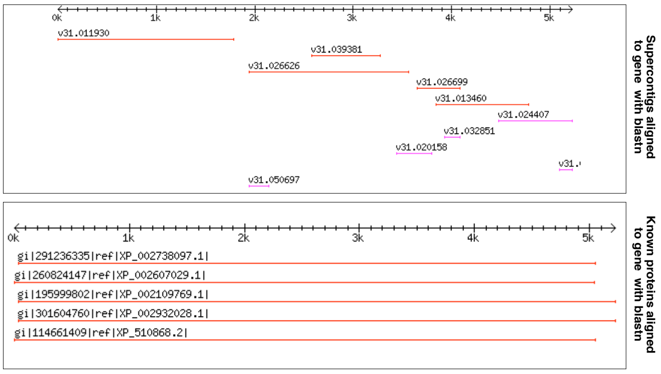

Gene_03593

Supercontigs mapped to this gene

v31.004888-, v31.017323-, v31.019154-, v31.023392-

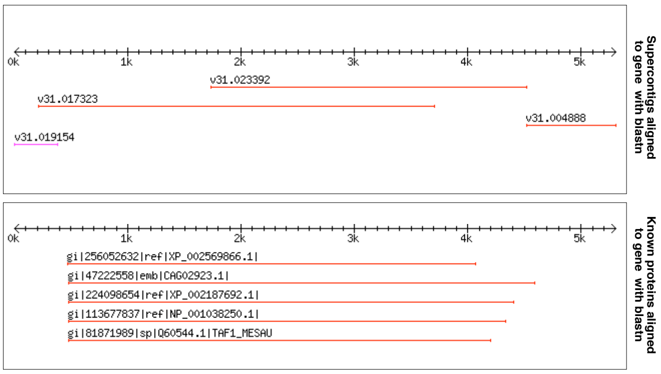

Gene_03624

Supercontigs mapped to this gene

v31.005250-, v31.010152-, v31.015498+, v31.029709+

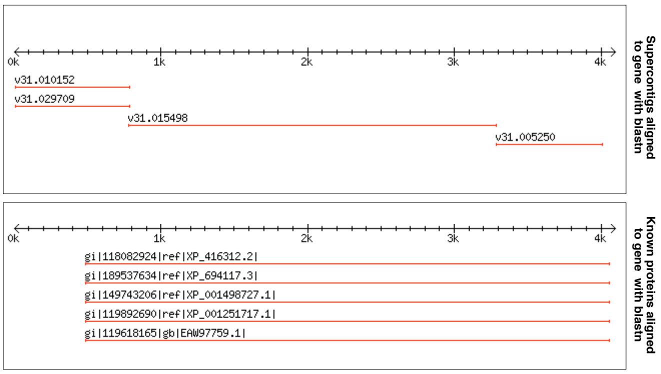

Gene_00365

Supercontigs mapped to this gene

v31.002101-, v31.003687+, v31.013433+, v31.025894-, v31.041533-

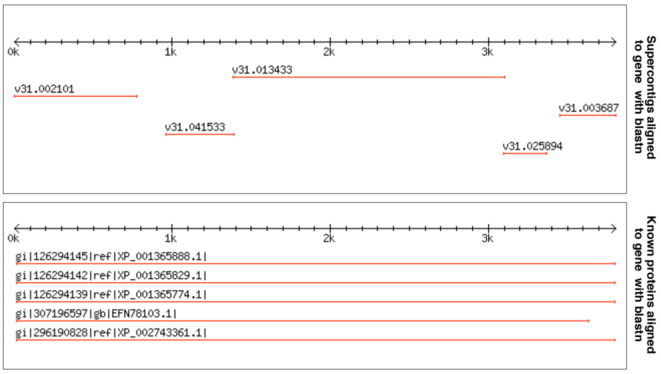

Gene_03688

Supercontigs mapped to this gene

v31.001228+, v31.005130-

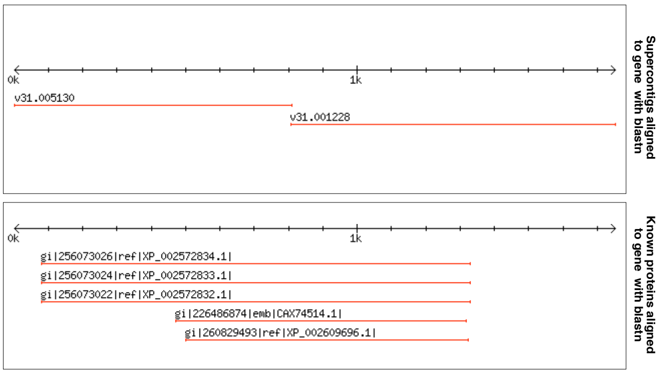

Gene_03682

Supercontigs mapped to this gene

v31.000132-, v31.002352+

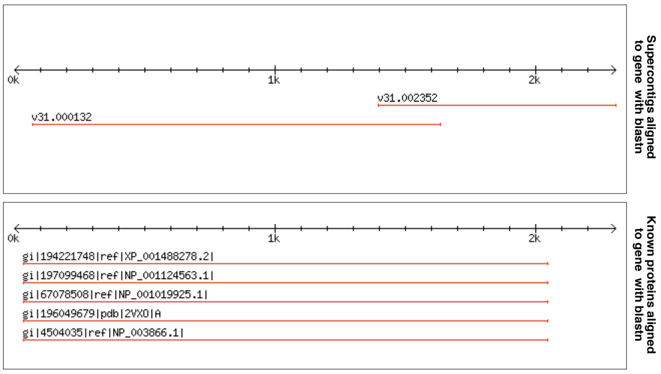

Gene_03692

Supercontigs mapped to this gene

v31.003611-, v31.024789-, v31.025867-, v31.044070-

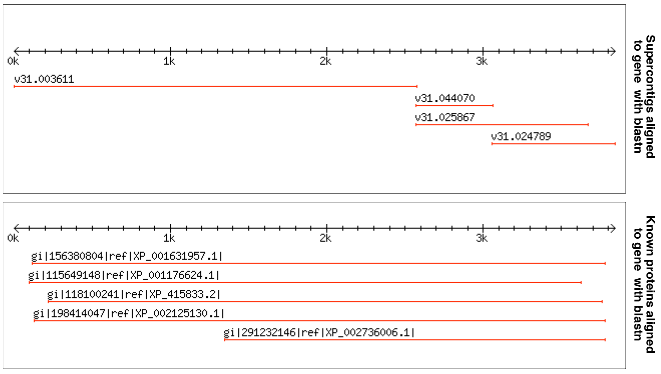

Gene_03706

Supercontigs mapped to this gene

v31.002533+, v31.003468-, v31.011318-, v31.013463-, v31.017780+, v31.022109-

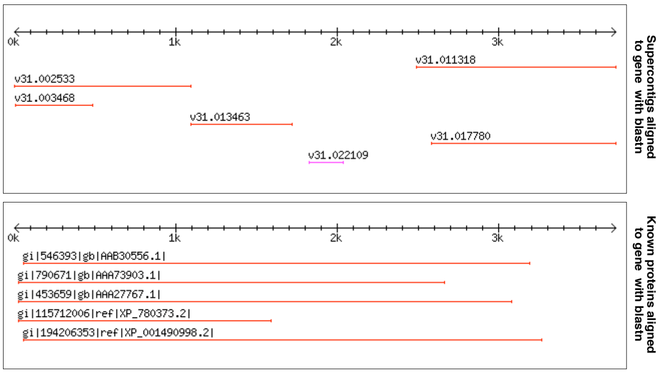

Gene_03721

Supercontigs mapped to this gene

v31.011277+, v31.013938-, v31.015873-, v31.017755-, v31.020788-

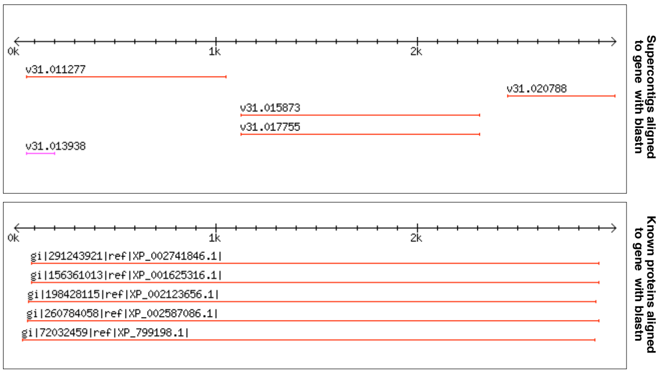

Gene_00372

Supercontigs mapped to this gene

v31.003744+, v31.008247+, v31.012654+, v31.031854-

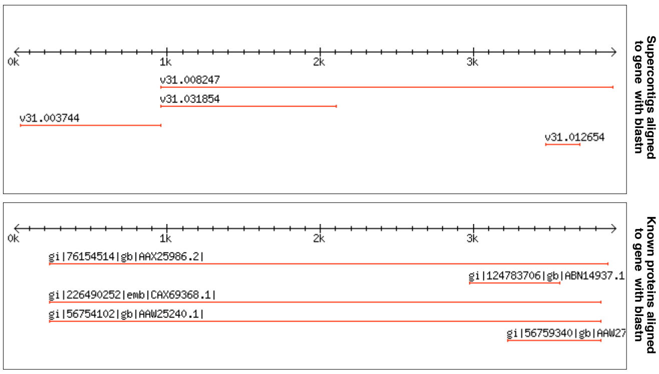

Gene_03752

Supercontigs mapped to this gene

v31.001457-, v31.024681-

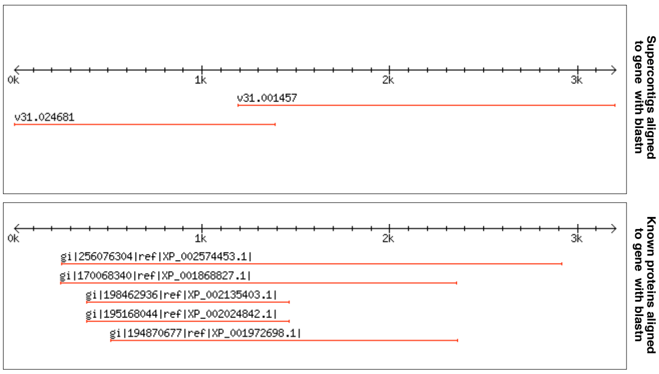

Gene_00381

Supercontigs mapped to this gene

v31.011860+, v31.014447+, v31.019357-, v31.019379-, v31.025737-, v31.030765+, v31.053429+

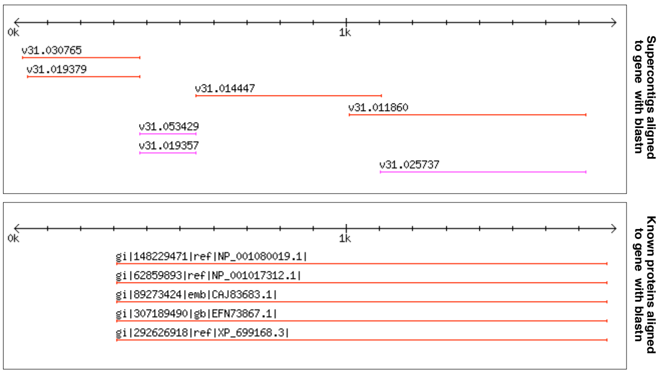

Gene_03818

Supercontigs mapped to this gene

v31.002169-, v31.003868-

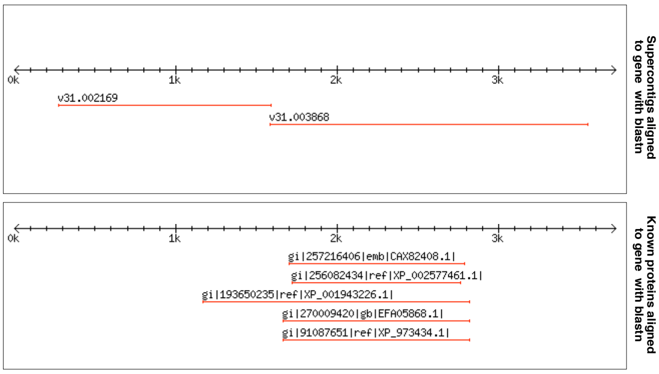

Gene_03810

Supercontigs mapped to this gene

v31.002344-, v31.004376-, v31.025145-

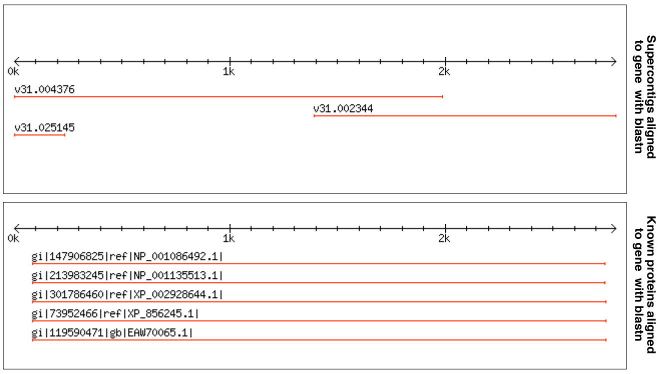

Gene_03861

Supercontigs mapped to this gene

v31.009181+, v31.012998-, v31.022388-, v31.025645+

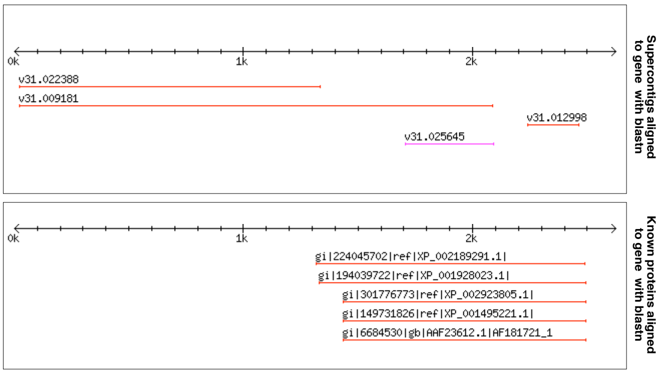

Gene_00388

Supercontigs mapped to this gene

v31.006141+, v31.009646-, v31.017290-, v31.021439+, v31.023323+, v31.026199+, v31.038976+

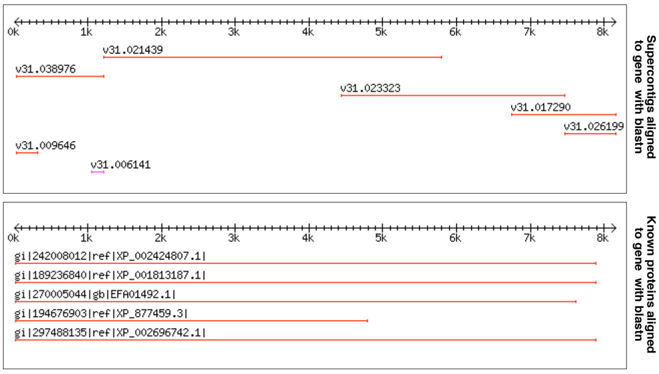

Gene_03908

Supercontigs mapped to this gene

v31.004874+, v31.008061-

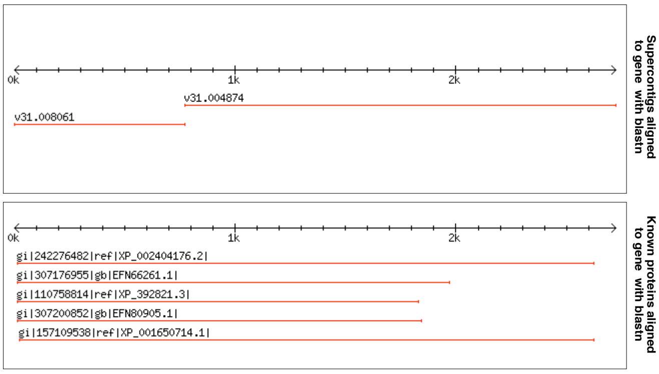

Gene_03891

Supercontigs mapped to this gene

v31.001722+, v31.013958-, v31.025814-

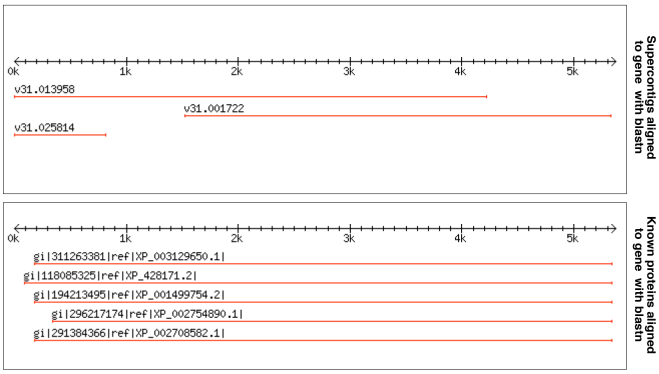

Gene_03909

Supercontigs mapped to this gene

v31.002608-, v31.007213-, v31.012312-, v31.018508-, v31.036973-

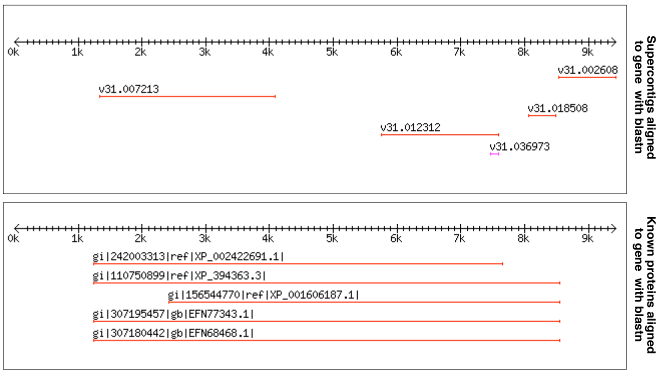

Gene_00392

Supercontigs mapped to this gene

v31.001384-, v31.002244+, v31.002305+, v31.005023-, v31.010145+, v31.023928+

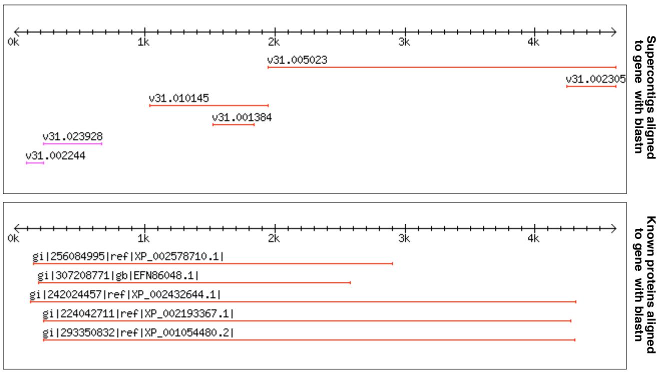

Gene_03987

Supercontigs mapped to this gene

v31.001376-, v31.003471+, v31.003952+

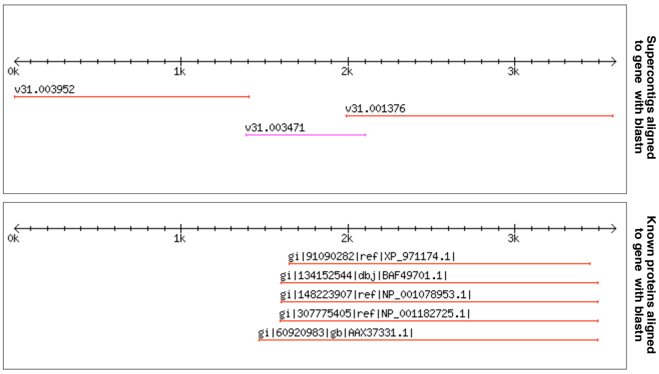

Gene_03957

Supercontigs mapped to this gene

v31.005771-, v31.012002-, v31.043745+

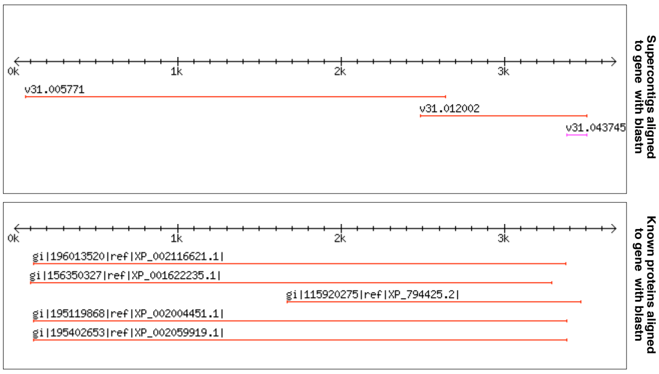

Gene_00399

Supercontigs mapped to this gene

v31.005928-, v31.007309+, v31.016936+

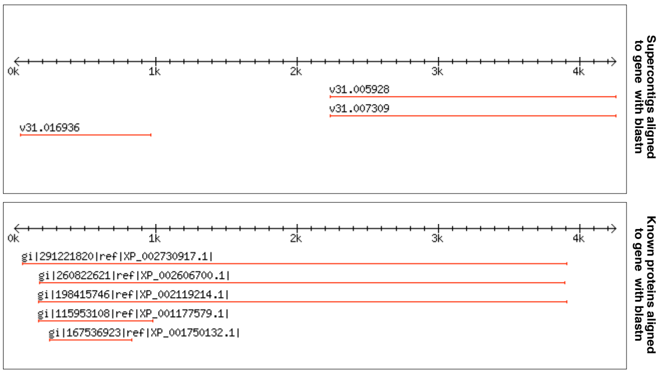

Gene_04016

Supercontigs mapped to this gene

v31.005503-, v31.021269+, v31.024671+

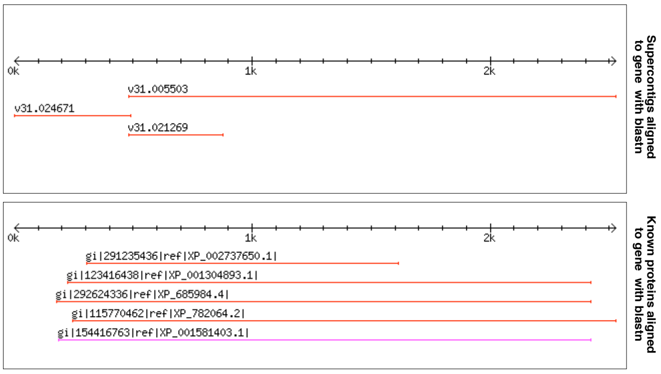

Gene_04035

Supercontigs mapped to this gene

v31.003540+, v31.023154+

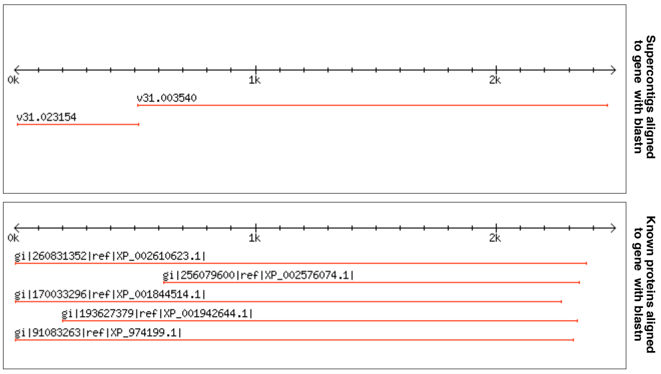

Gene_04020

Supercontigs mapped to this gene

v31.000695+, v31.001409-

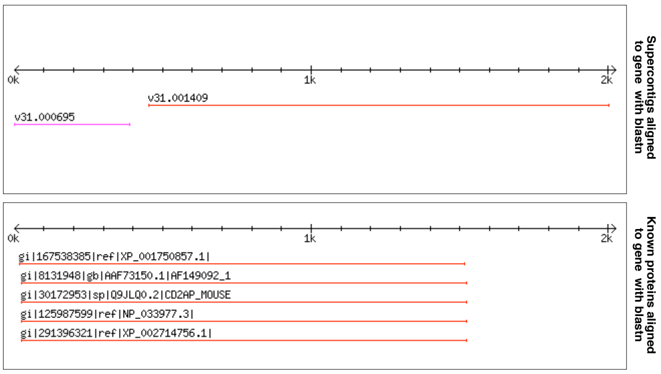

Gene_04038

Supercontigs mapped to this gene

v31.001705+, v31.003080+

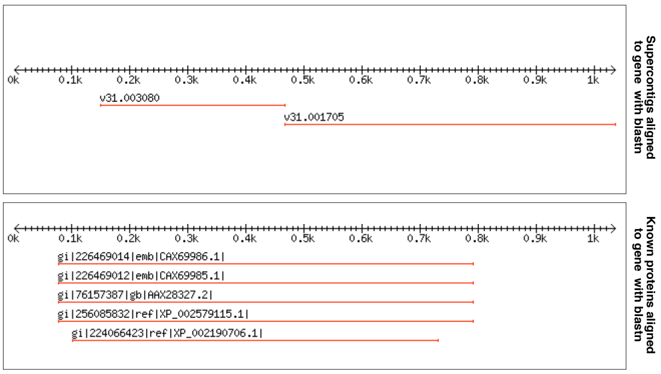

Gene_00406

Supercontigs mapped to this gene

v31.003365+, v31.005542+, v31.006962+, v31.011388+, v31.012033-, v31.015628+, v31.017130+, v31.021055-, v31.021856+, v31.036135-

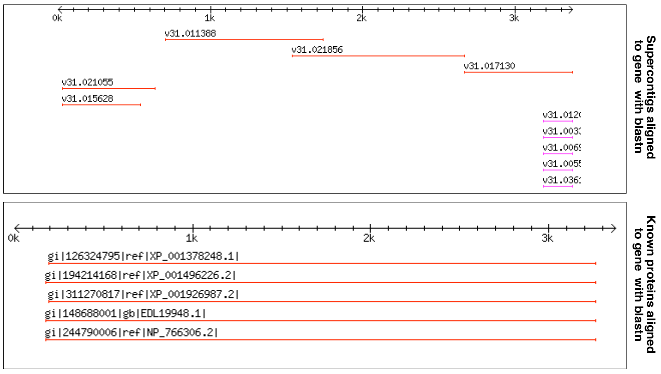

Gene_04112

Supercontigs mapped to this gene

v31.004354-, v31.010861+

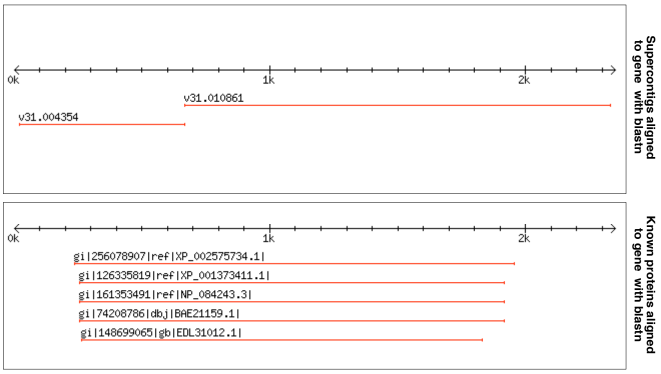

Gene_04102

Supercontigs mapped to this gene

v31.007189+, v31.011521-, v31.019625-, v31.022961-, v31.027764-, v31.029843+

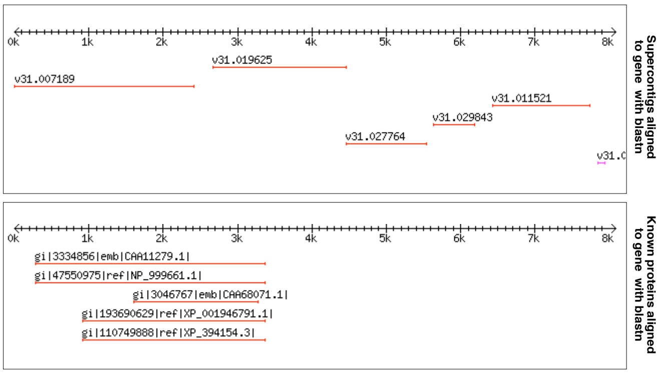

Gene_04117

Supercontigs mapped to this gene

v31.002291-, v31.009667-

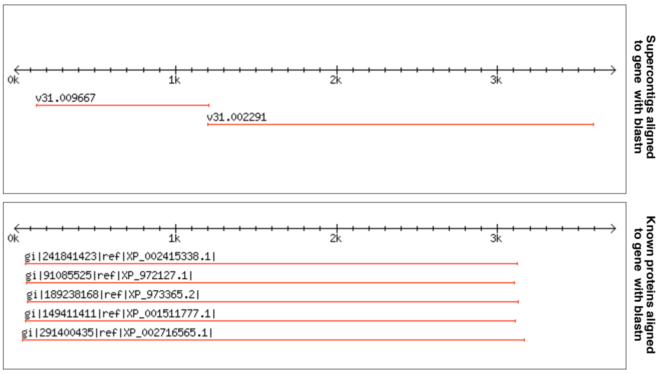

Gene_04123

Supercontigs mapped to this gene
v31.000689-, v31.001874+, v31.005337-

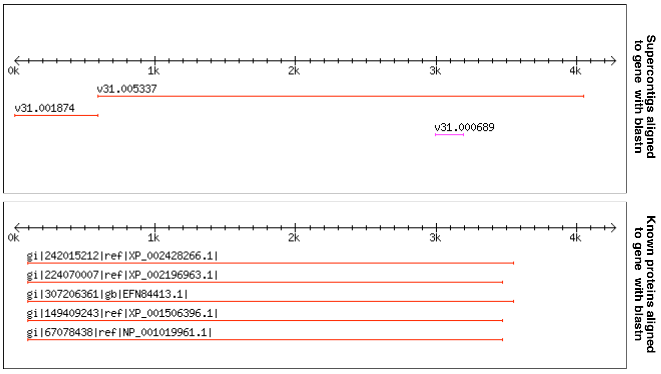

Gene_04179

Supercontigs mapped to this gene
v31.002894+, v31.004036-

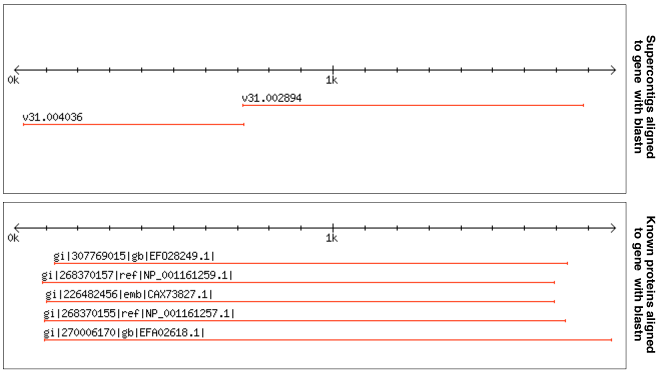

Gene_04139

Supercontigs mapped to this gene
v31.003936-, v31.004676+

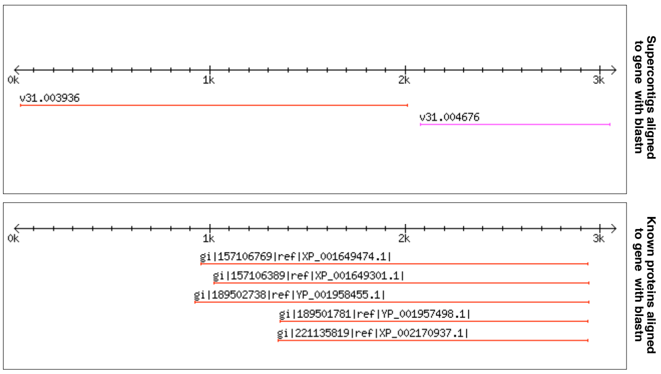

Gene_00419

Supercontigs mapped to this gene
v31.014140+, v31.015554+, v31.016070-, v31.032484-, v31.035562+

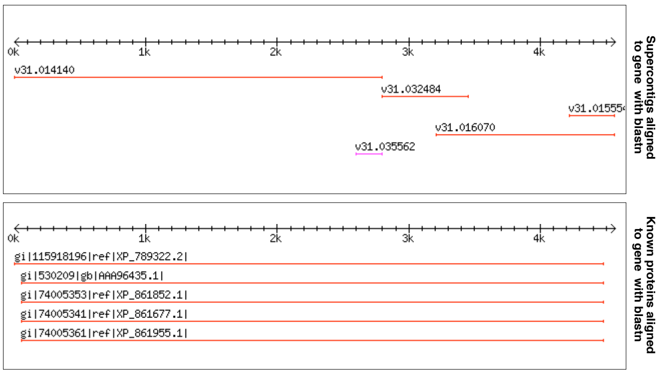

Gene_04191

Supercontigs mapped to this gene
v31.002299-, v31.007750+, v31.021250-

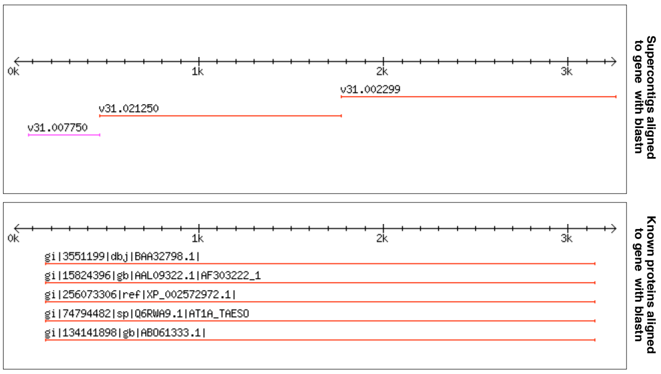

Gene_04223

Supercontigs mapped to this gene
v31.009011-, v31.015554+, v31.018267-, v31.026864+, v31.031853-, v31.040346-

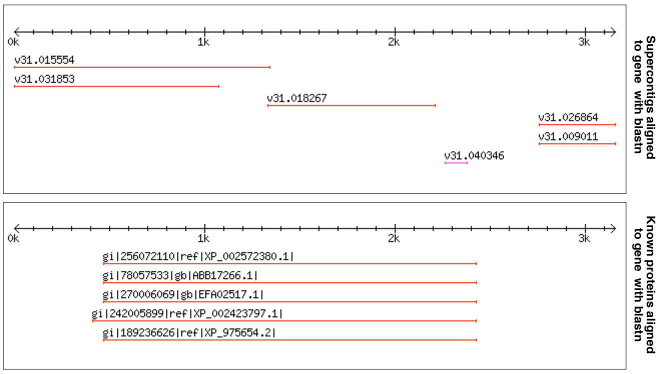

Gene_04215

Supercontigs mapped to this gene
v31.002238+, v31.007096-, v31.015585-, v31.035338+, v31.038443+

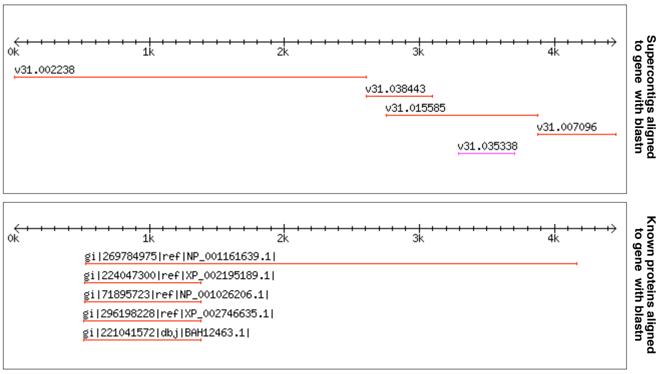

Gene_00424

Supercontigs mapped to this gene
v31.000896-, v31.007040+

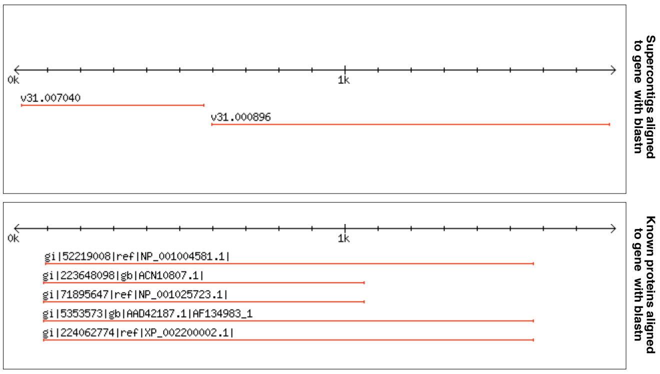

Gene_04257

Supercontigs mapped to this gene

v31.003221-, v31.003924+, v31.011577-

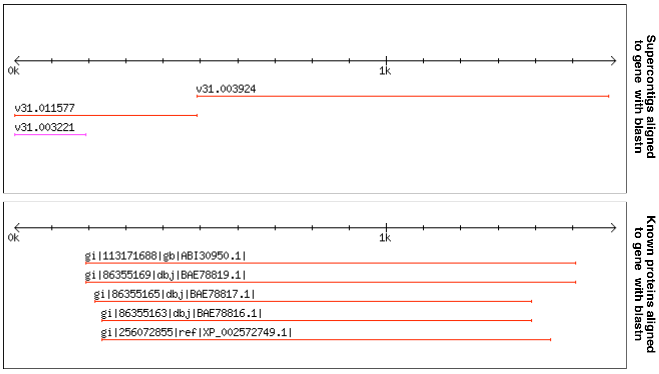

Gene_04269

Supercontigs mapped to this gene

v31.003742+, v31.013368+, v31.021685+, v31.022220-, v31.024887-

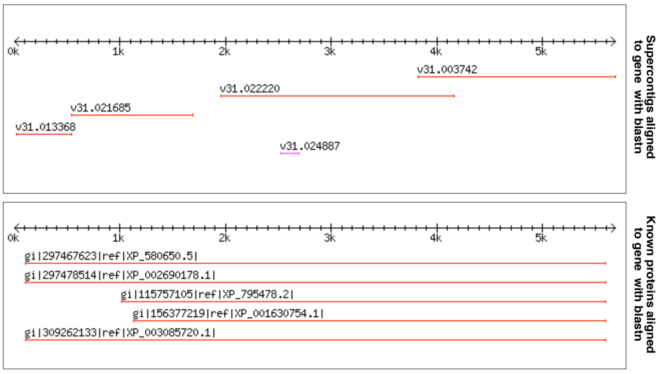

Gene_04258

Supercontigs mapped to this gene

v31.000865+, v31.001396+

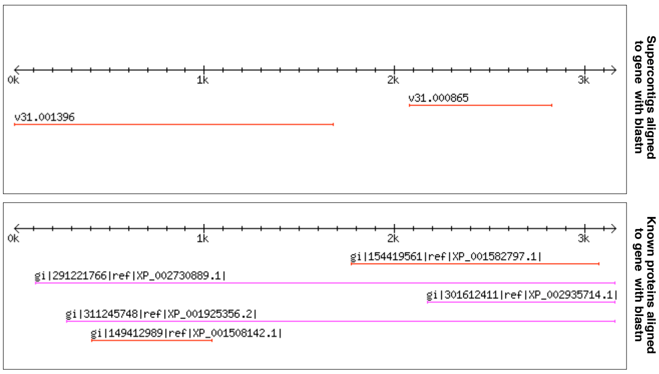

Gene_04293

Supercontigs mapped to this gene

v31.005122-, v31.017536-

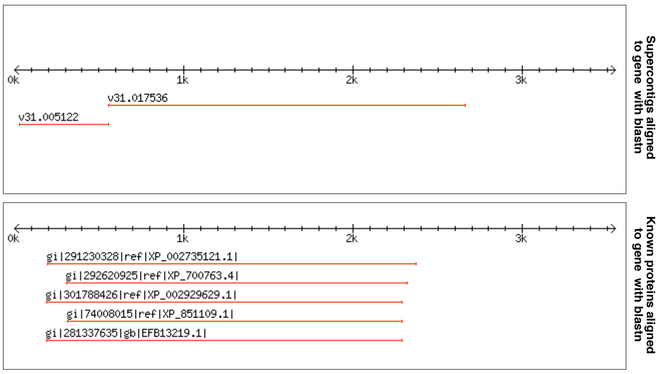

Gene_00431

Supercontigs mapped to this gene

v31.002654+, v31.003329+, v31.009693-, v31.009876-, v31.012677-

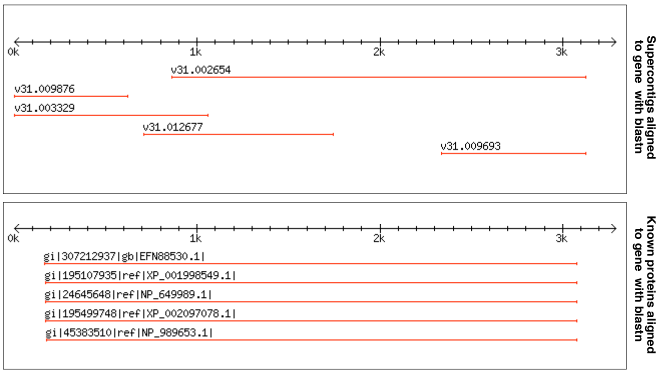

Gene_04314

Supercontigs mapped to this gene

v31.001274-, v31.015555-, v31.022420-, v31.024086+

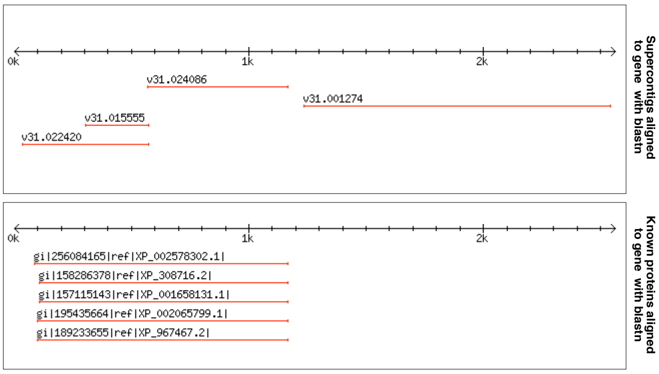

Gene_04312

Supercontigs mapped to this gene

v31.015776+, v31.016154-, v31.016970+, v31.022777+, v31.022876-

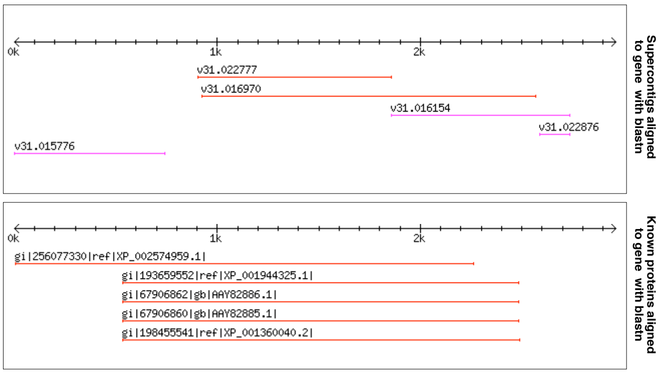

Gene_04319

Supercontigs mapped to this gene

v31.008476-, v31.014250+, v31.039248+, v31.042469+

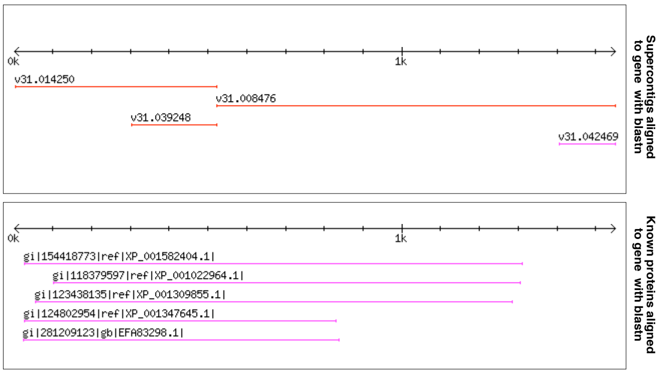

Gene_04320

Supercontigs mapped to this gene
v31.001155-, v31.002141-

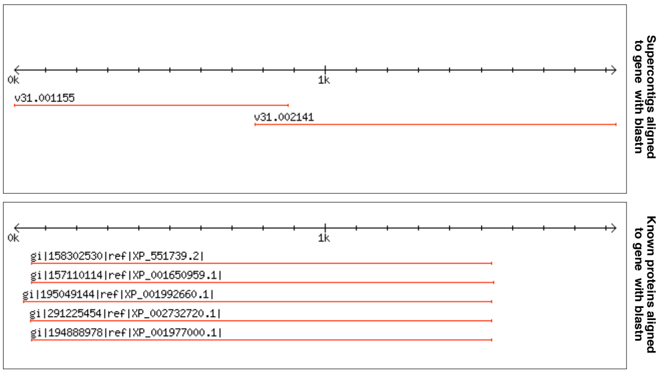

Gene_04393

Supercontigs mapped to this gene
v31.006281+, v31.016797+, v31.019696-, v31.051622+

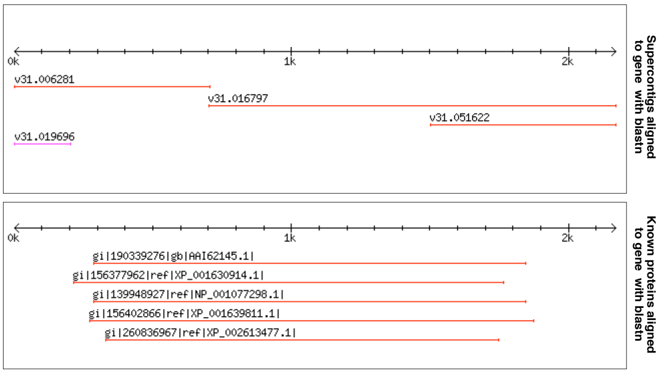

Gene_04387

Supercontigs mapped to this gene
v31.000983-, v31.004907-

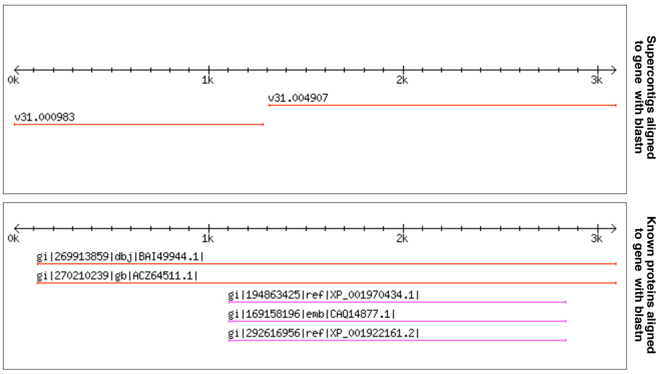

Gene_04416

Supercontigs mapped to this gene
v31.007900+, v31.008159-

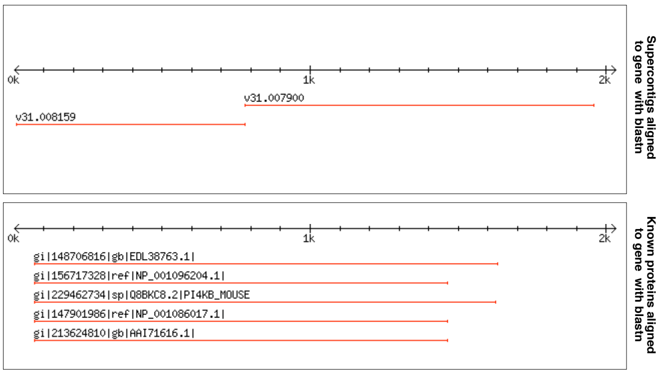

Gene_04419

Supercontigs mapped to this gene
v31.006201+, v31.015944-, v31.019809-

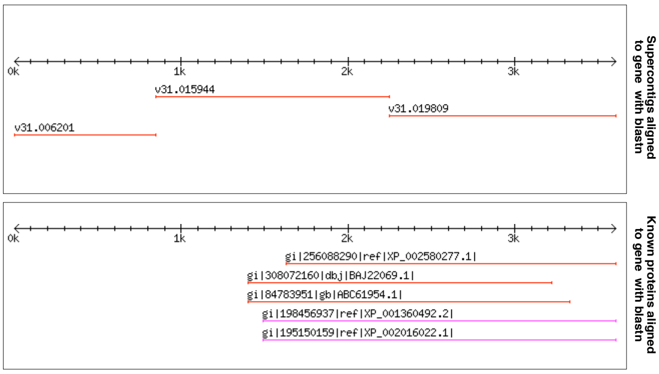

Gene_04464

Supercontigs mapped to this gene
v31.007166-, v31.007904+, v31.017196-

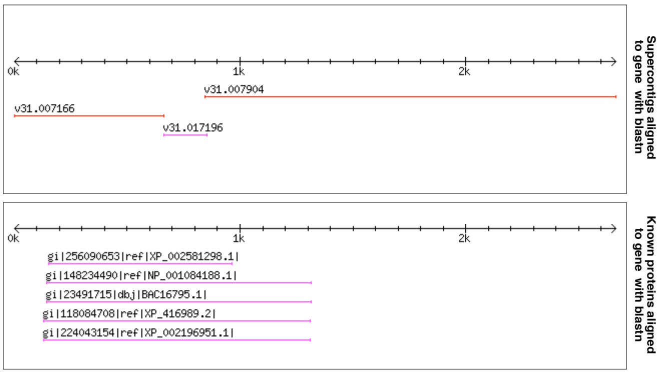

Gene_04438

Supercontigs mapped to this gene
v31.001986+, v31.002290-

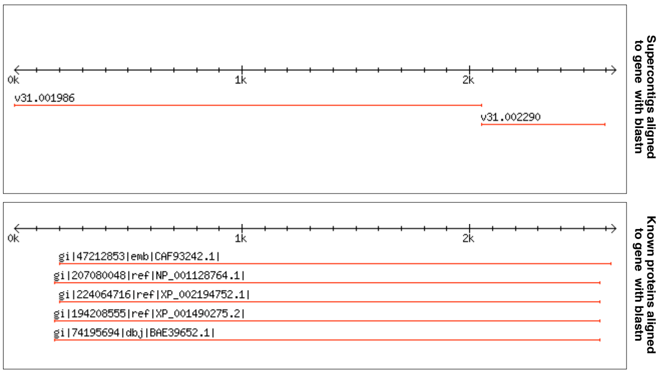

Gene_04471

Supercontigs mapped to this gene
v31.006915-, v31.010593-, v31.011705+, v31.019762+,
v31.020467-, v31.021067-, v31.023802-

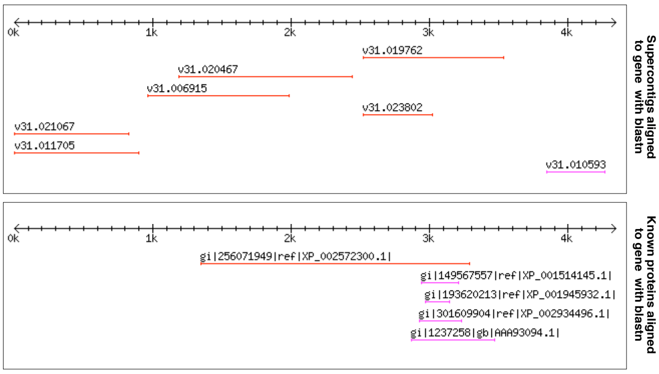

Gene_04488

Supercontigs mapped to this gene

v31.000870+, v31.011285+

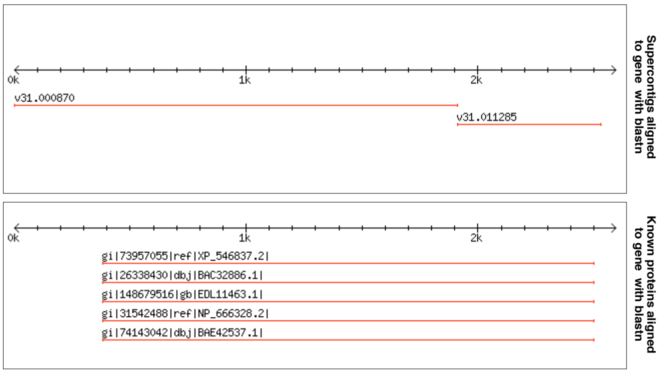

Gene_04519

Supercontigs mapped to this gene

v31.004787+, v31.004796-, v31.010536-, v31.026207-, v31.052699-, v31.078820-

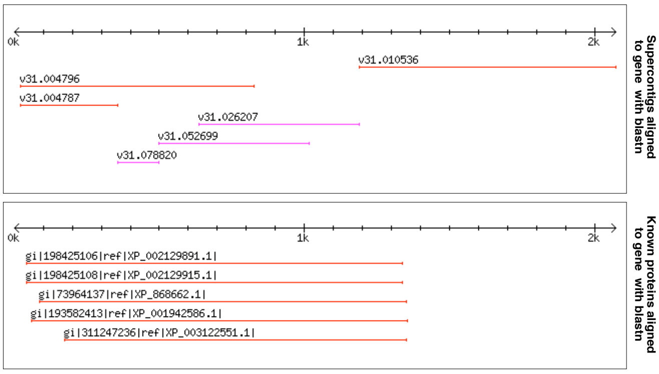

Gene_04501

Supercontigs mapped to this gene

v31.000154-, v31.012825-

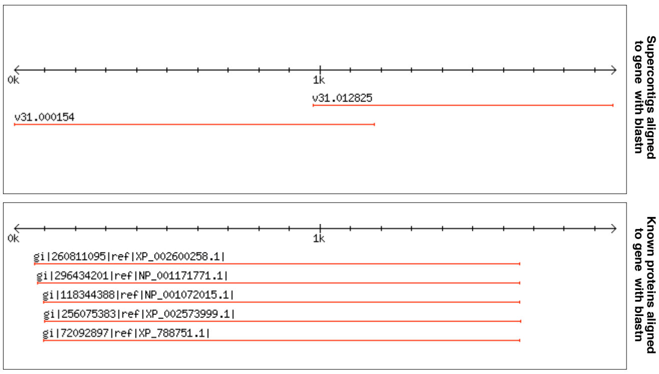

Gene_04523

Supercontigs mapped to this gene

v31.004750-, v31.013197+, v31.015582+, v31.019727-, v31.050082-

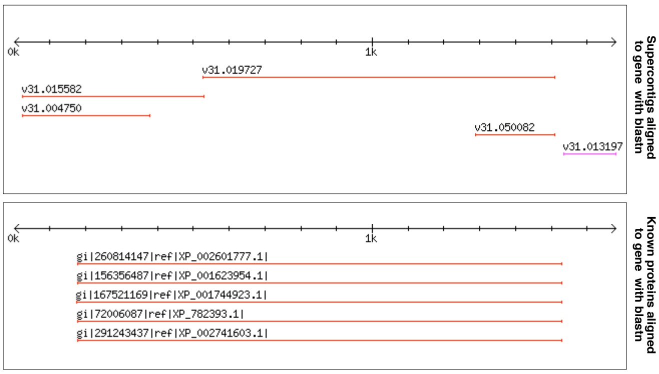

Gene_04543

Supercontigs mapped to this gene

v31.011274+, v31.015595-, v31.023671-

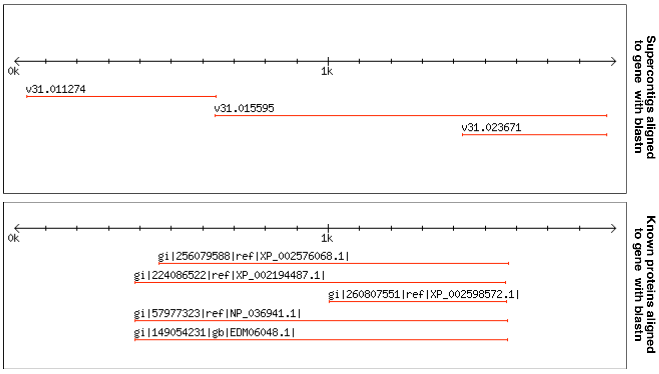

Gene_04564

Supercontigs mapped to this gene

v31.003324+, v31.007006+

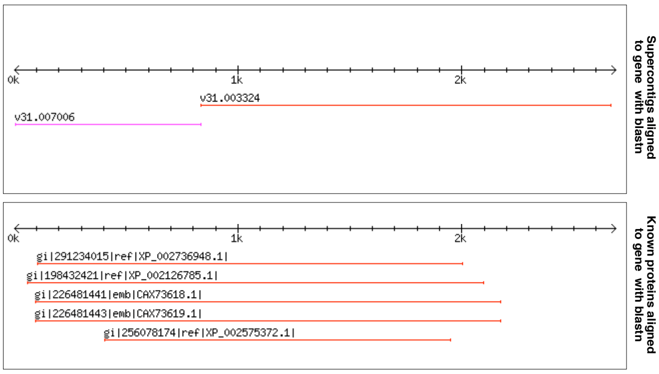

Gene_04563

Supercontigs mapped to this gene

v31.001325-, v31.007864-, v31.007995-

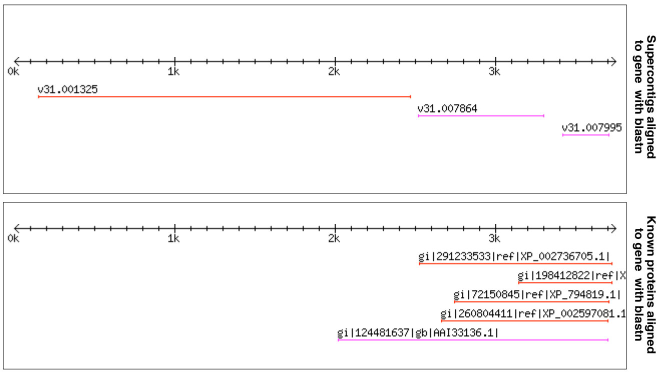

Gene_04595

Supercontigs mapped to this gene

v31.002536+, v31.003806-

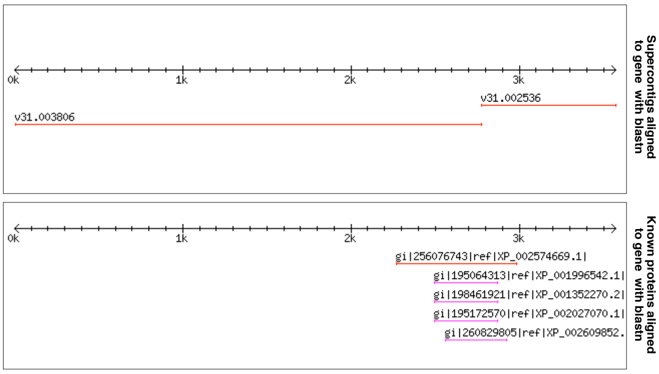

Gene_00046

Supercontigs mapped to this gene
v31.002286-, v31.007194+

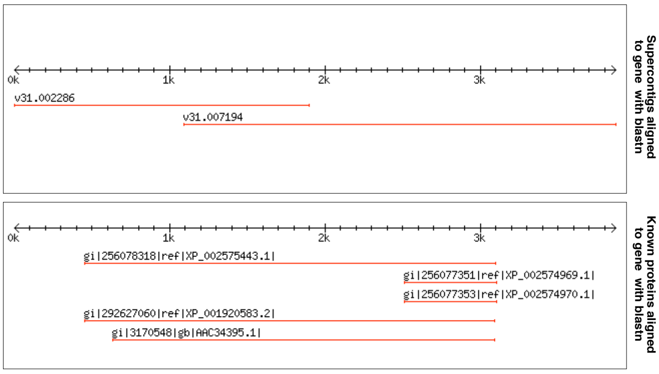

Gene_04631

Supercontigs mapped to this gene
v31.002853-, v31.010046+

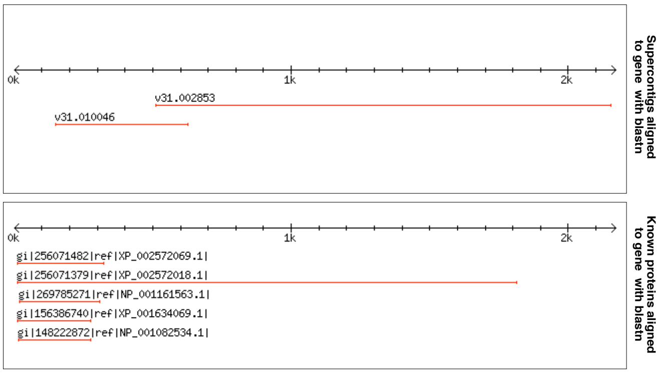

Gene_04605

Supercontigs mapped to this gene
v31.000418+, v31.000676-

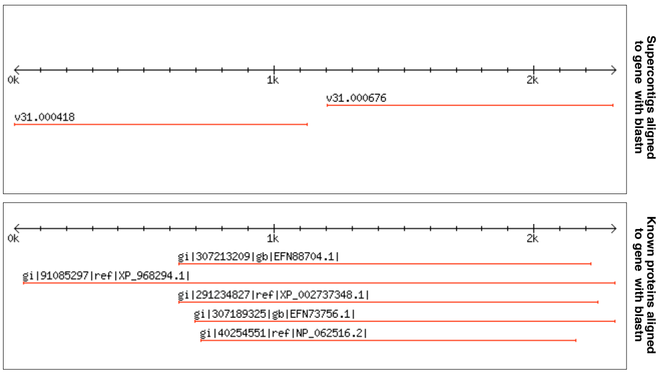

Gene_04644

Supercontigs mapped to this gene
v31.004233+, v31.006391+, v31.015148-, v31.050677-

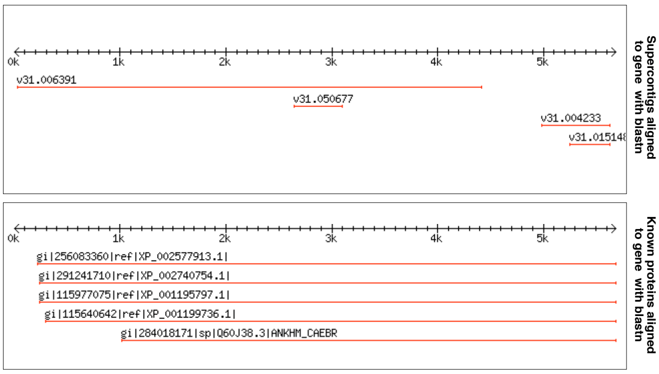

Gene_04690

Supercontigs mapped to this gene
v31.000121+, v31.005302+

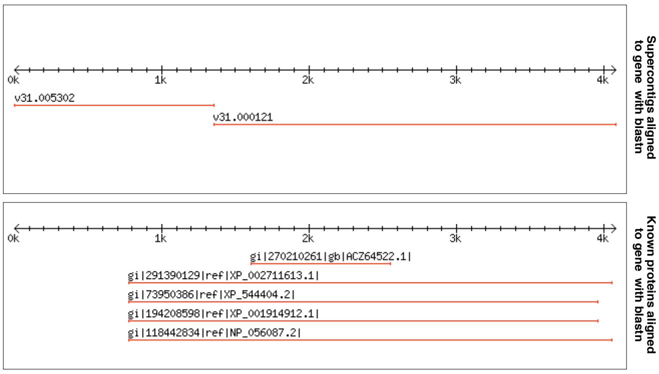

Gene_04757

Supercontigs mapped to this gene
v31.011887+, v31.019526+, v31.026779-

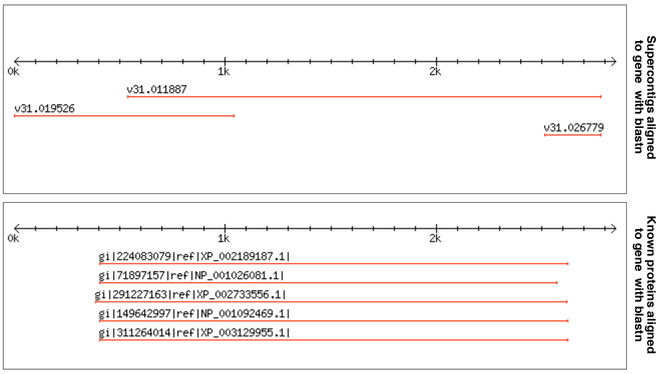

Gene_00470

Supercontigs mapped to this gene
v31.002285+, v31.009130-, v31.020174-

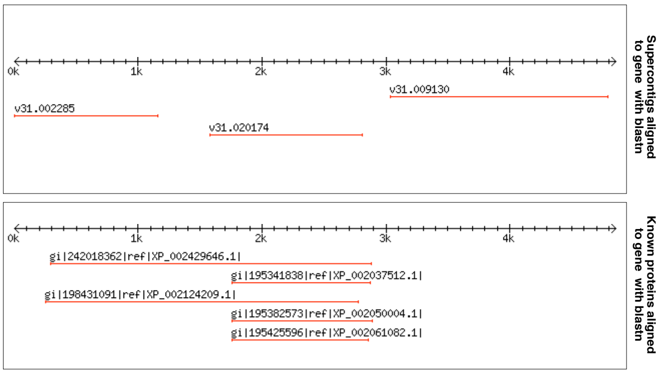

Gene_00479

Supercontigs mapped to this gene
v31.005840-, v31.017311+, v31.031546+

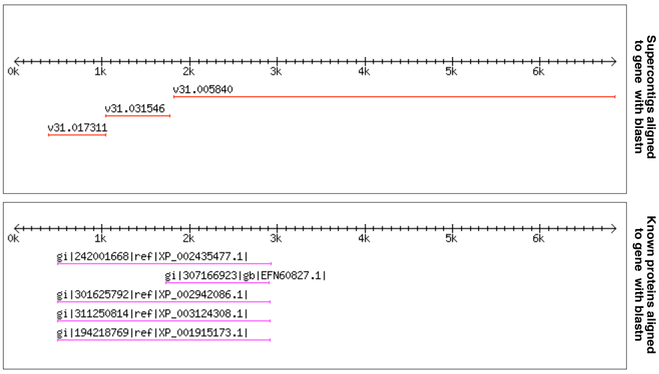

Gene_04792

Supercontigs mapped to this gene
v31.000297+, v31.010169-

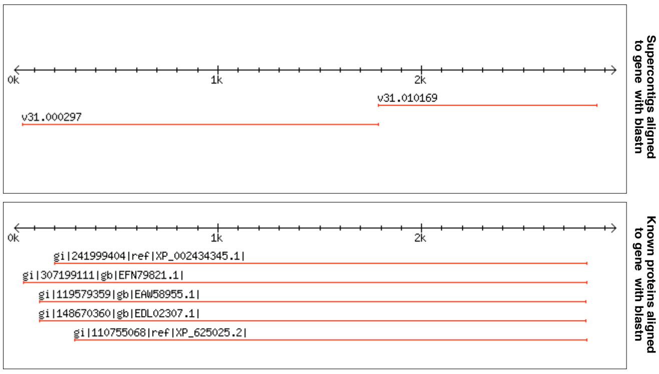

Gene_04807

Supercontigs mapped to this gene
v31.001444+, v31.013529+

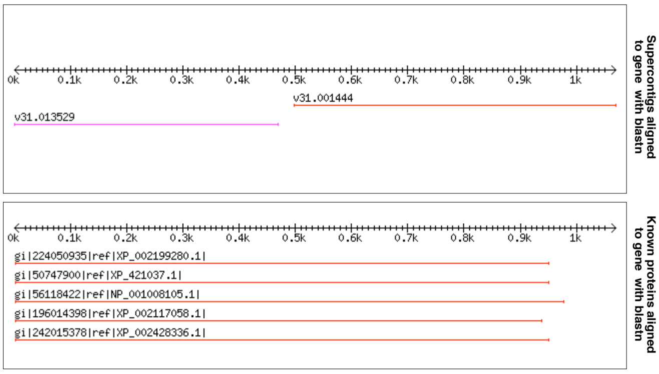

Gene_04803

Supercontigs mapped to this gene
v31.002359+, v31.002535-

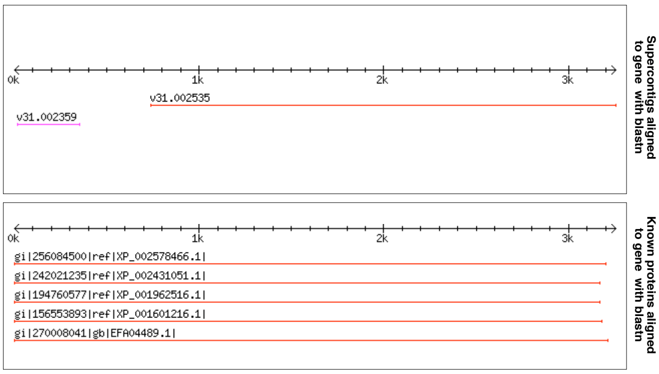

Gene_04813

Supercontigs mapped to this gene
v31.006686-, v31.013405+, v31.021616+, v31.023641-, v31.024311-, v31.026133-, v31.044345+

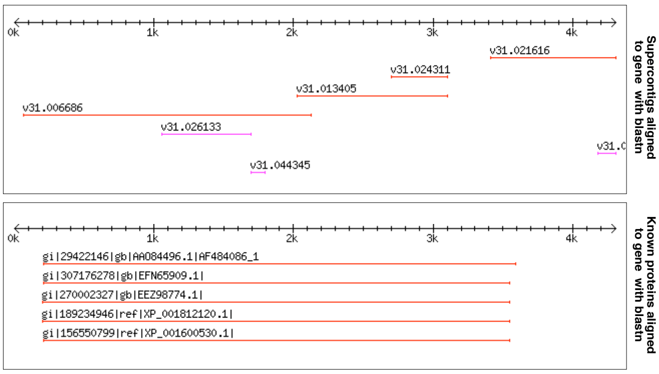

Gene_04824

Supercontigs mapped to this gene
v31.005652-, v31.009161+, v31.018372-, v31.021305-

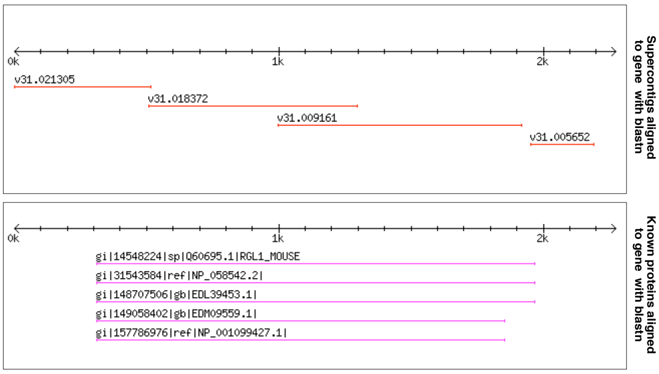

Gene_04851

Supercontigs mapped to this gene
v31.000453-, v31.003956+

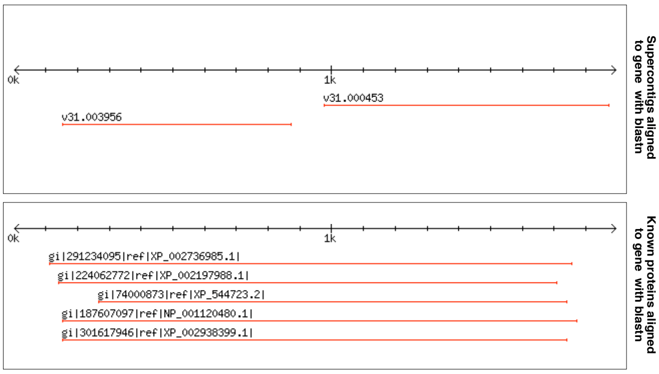

Gene_04850

Supercontigs mapped to this gene
v31.010155-, v31.010238+, v31.015131+, v31.022848-

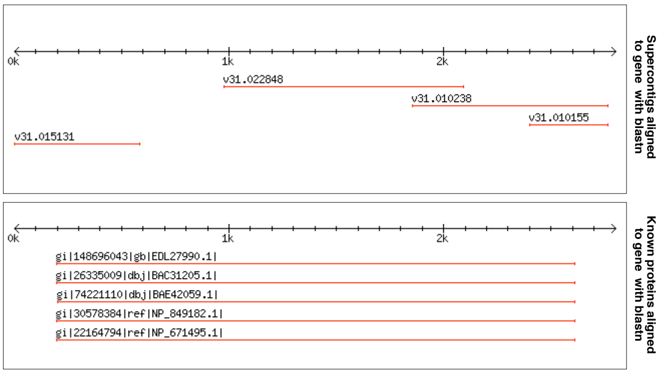

Gene_04853

Supercontigs mapped to this gene
v31.003232-, v31.006104-

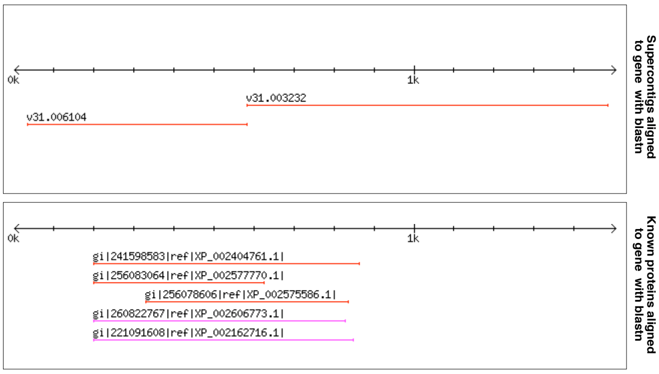

Gene_04872

Supercontigs mapped to this gene

v31.002136-, v31.011640-

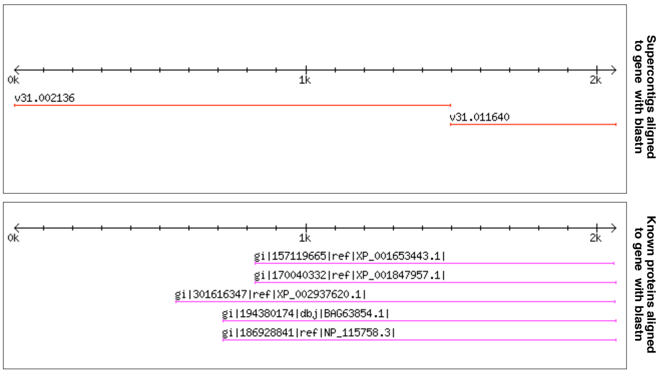

Gene_04917

Supercontigs mapped to this gene

v31.000102+, v31.000320+, v31.004540+

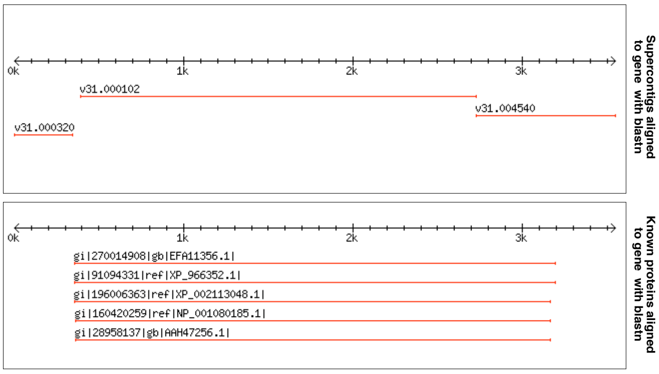

Gene_04873

Supercontigs mapped to this gene

v31.004340-, v31.004889-

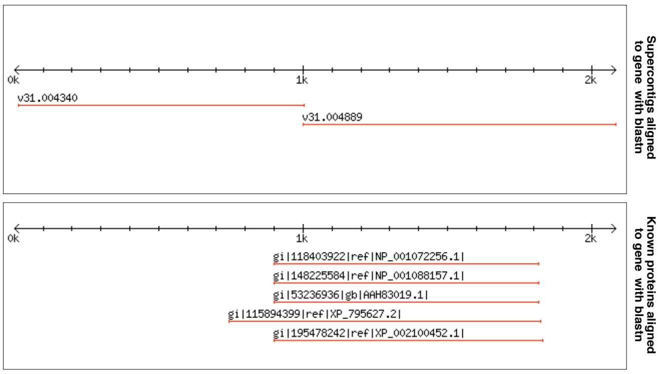

Gene_04947

Supercontigs mapped to this gene

v31.007076+, v31.010725+, v31.044688+

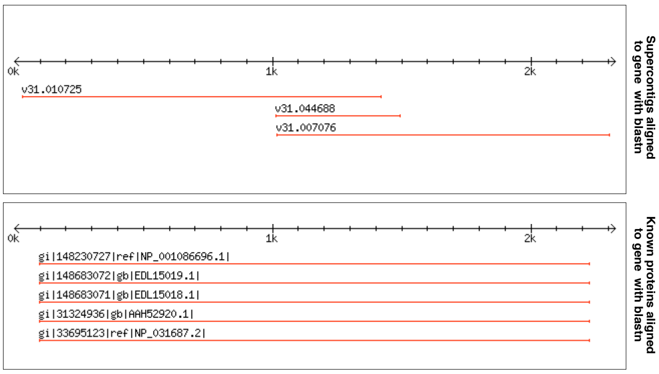

Gene_04981

Supercontigs mapped to this gene

v31.000503+, v31.003433+

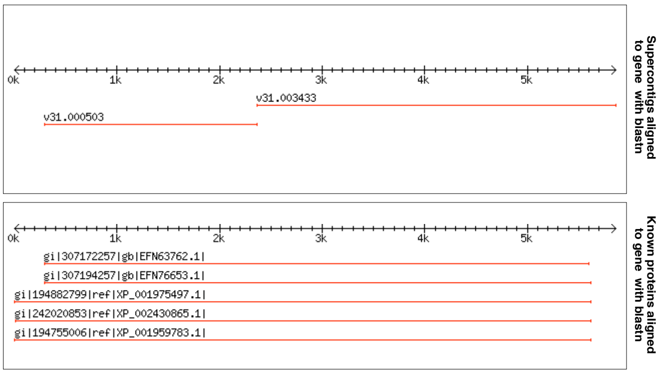

Gene_05043

Supercontigs mapped to this gene

v31.018361-, v31.019078-

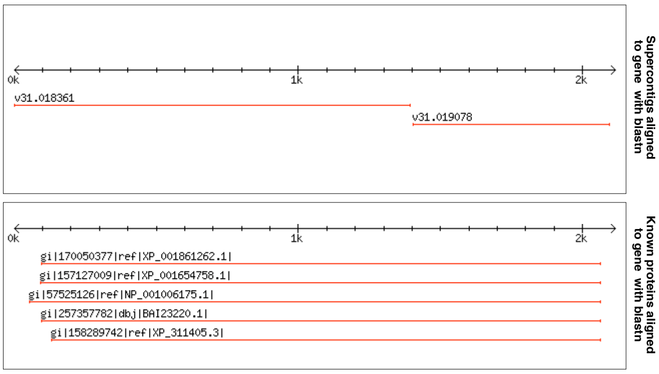

Gene_05017

Supercontigs mapped to this gene

v31.000741+, v31.000957-

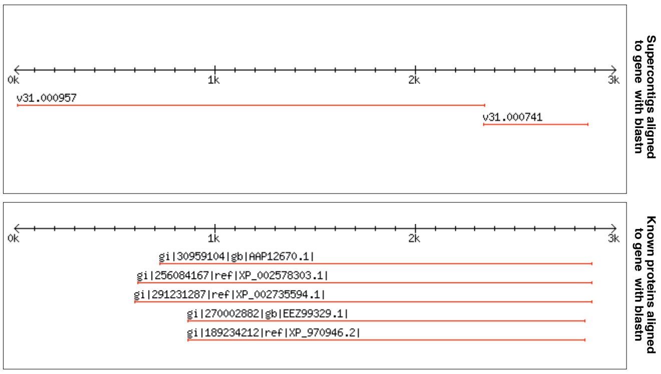

Gene_05048

Supercontigs mapped to this gene

v31.016496-, v31.020124-, v31.024887+, v31.039649+

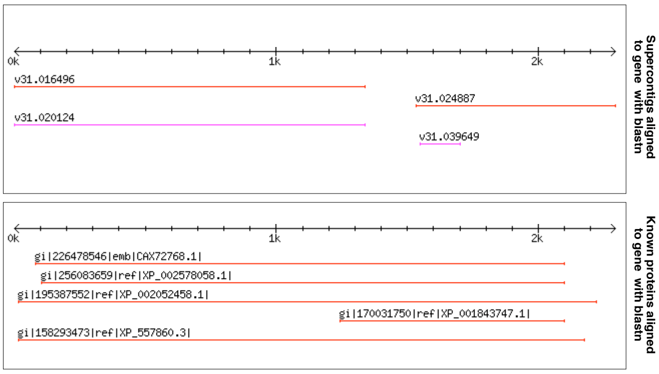

Gene_05050

Supercontigs mapped to this gene

v31.001043+, v31.001698-, v31.006077-, v31.009835+

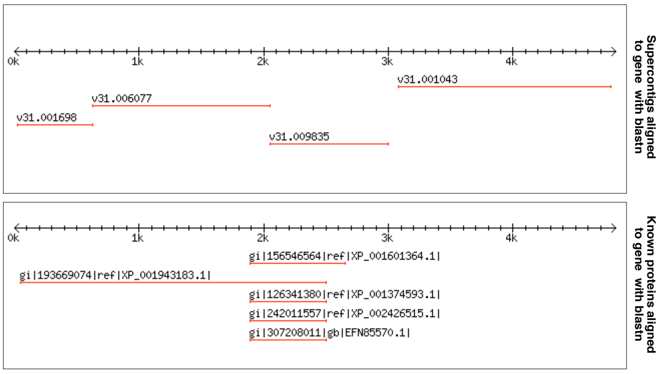

Gene_05058

Supercontigs mapped to this gene

v31.001634-, v31.004723-

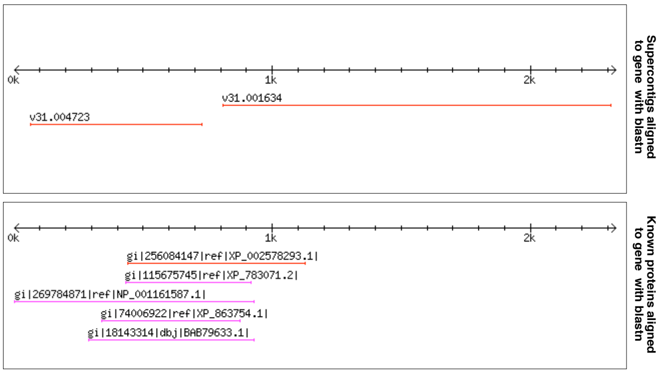

Gene_05055

Supercontigs mapped to this gene

v31.001594-, v31.015490-, v31.045120+

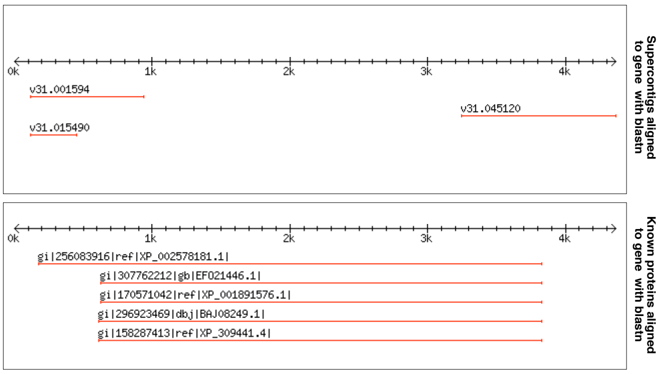

Gene_05121

Supercontigs mapped to this gene

v31.000577-, v31.004588-, v31.006977+

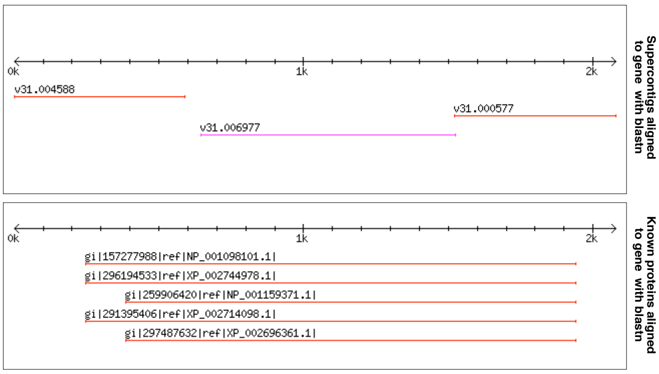

Gene_05137

Supercontigs mapped to this gene

v31.001585-, v31.003220-, v31.027624+

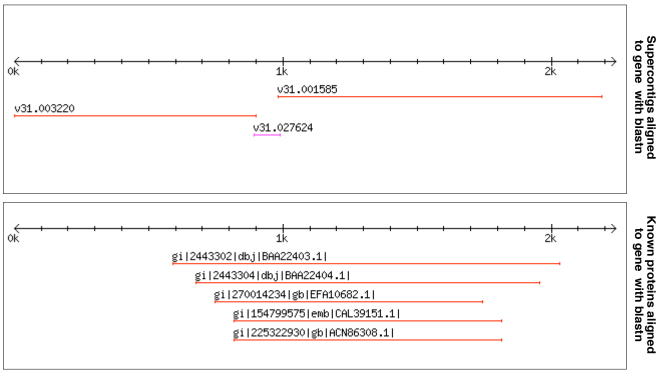

Gene_05170

Supercontigs mapped to this gene

v31.005629+, v31.019897+

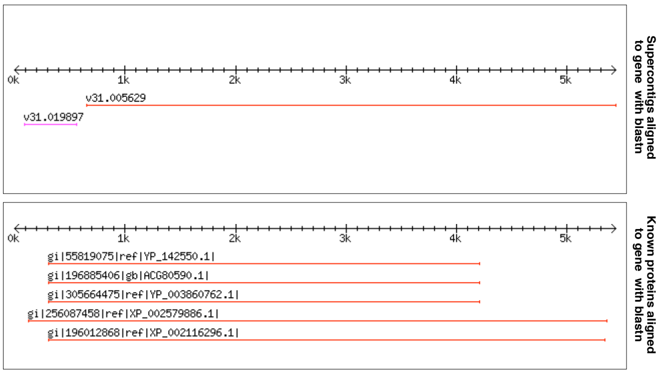

Gene_00514

Supercontigs mapped to this gene

v31.005685-, v31.006247+, v31.013907+, v31.029716+, v31.033983+

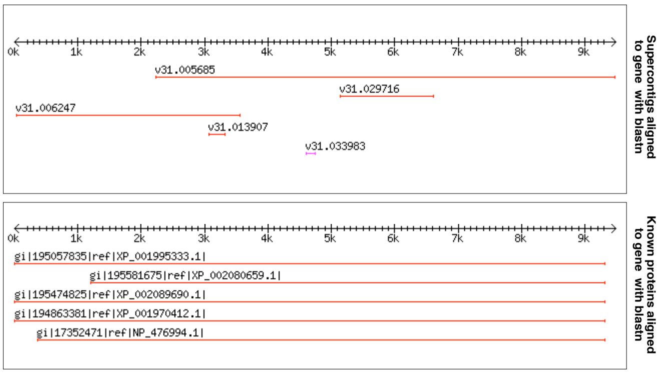

Gene_05193

Supercontigs mapped to this gene

v31.000424-, v31.000569-

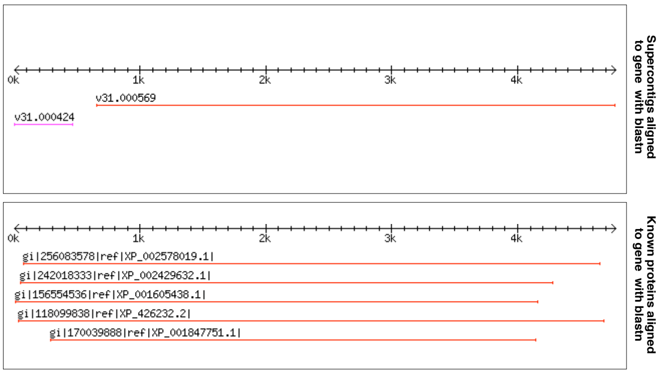

Gene_05214

Supercontigs mapped to this gene

v31.008558+, v31.015307-, v31.018862-, v31.019603+, v31.023248-, v31.025536-

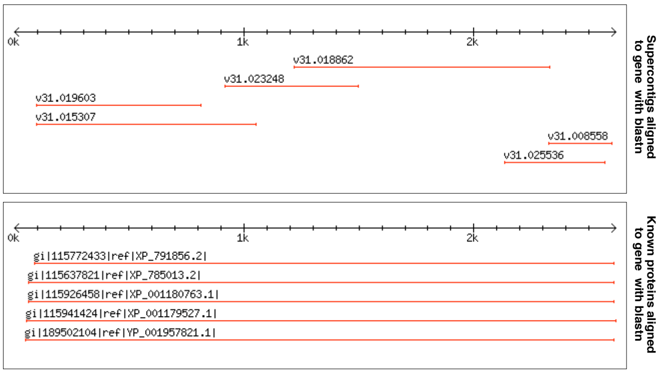

Gene_05240

Supercontigs mapped to this gene

v31.000834-, v31.002364-

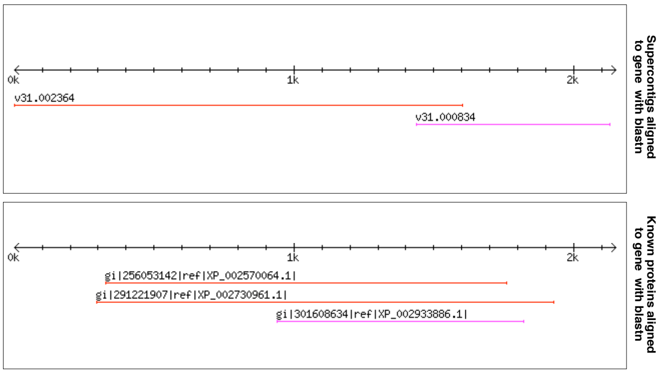

Gene_05224

Supercontigs mapped to this gene

v31.005028+, v31.006698+, v31.007408+, v31.021755+

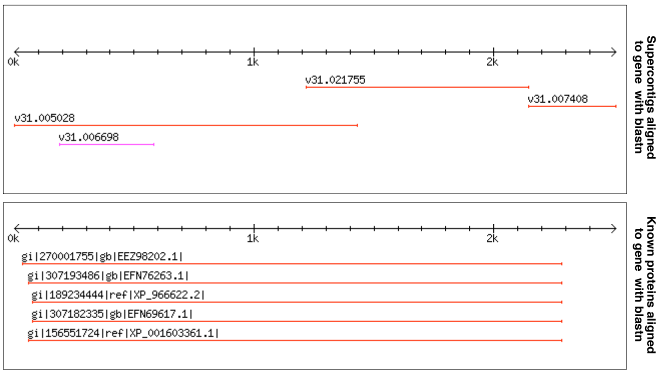

Gene_05241

Supercontigs mapped to this gene

v31.000053+, v31.000728+

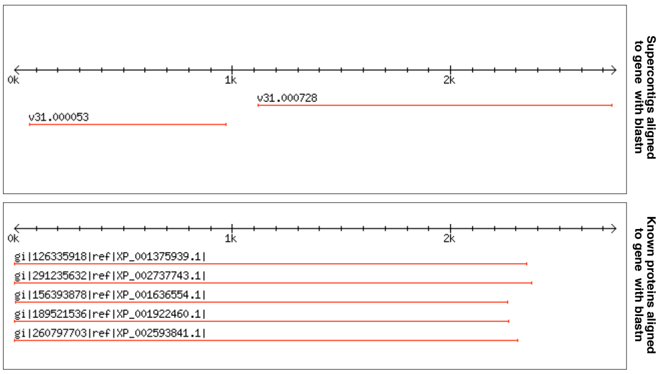

Gene_05257

Supercontigs mapped to this gene

v31.003377-, v31.004267+, v31.015147+

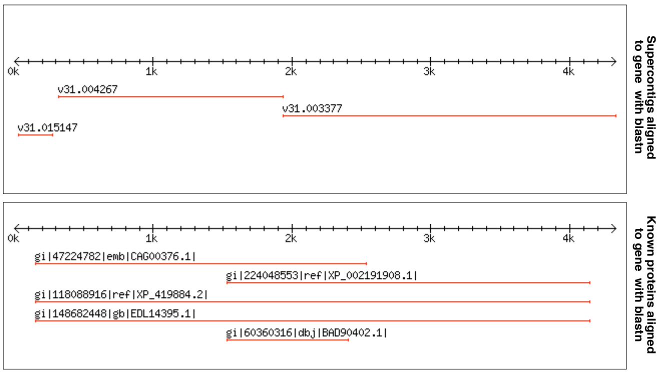

Gene_05327

Supercontigs mapped to this gene

v31.003801+, v31.006788-, v31.016348+, v31.016540-

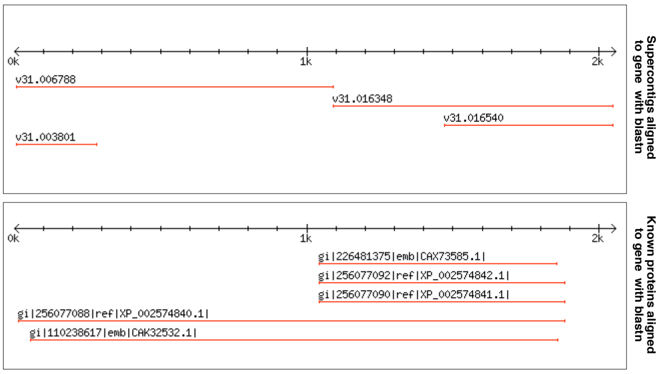

Gene_05288

Supercontigs mapped to this gene

v31.001591-, v31.002475+, v31.008740+

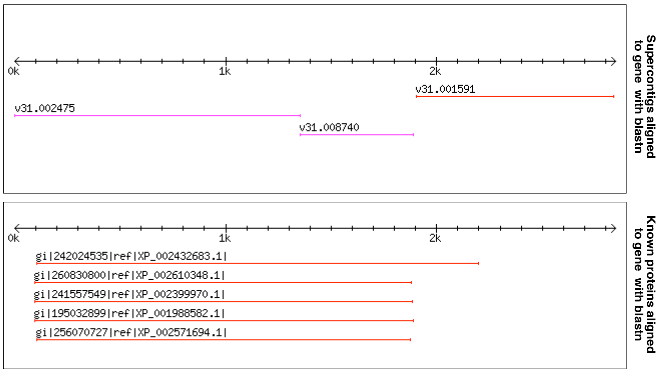

Gene_05351

Supercontigs mapped to this gene

v31.005922-, v31.006142+, v31.020734-

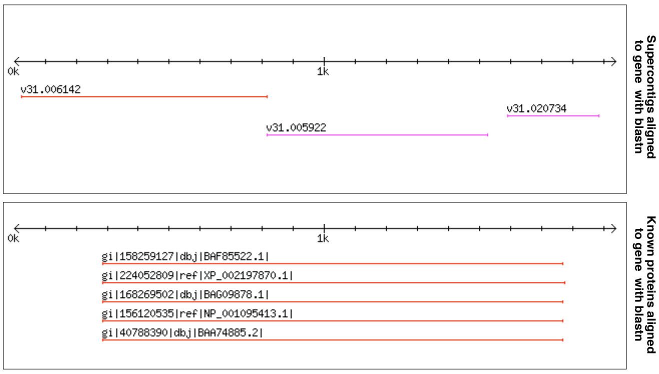

Gene_05384

Supercontigs mapped to this gene

v31.004097-, v31.007690-, v31.011457-

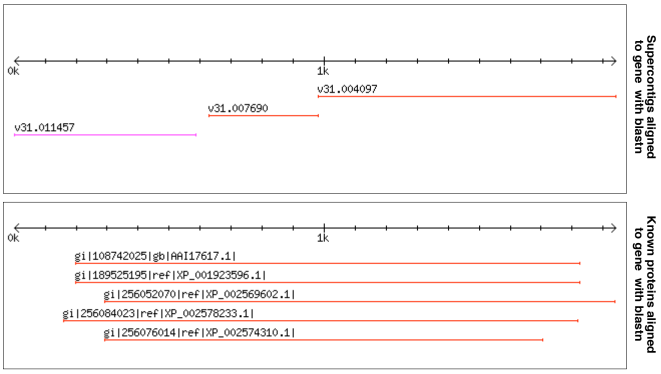

Gene_05391

Supercontigs mapped to this gene

v31.010187-, v31.018397+, v31.019504+, v31.020633+, v31.023251+, v31.038801+

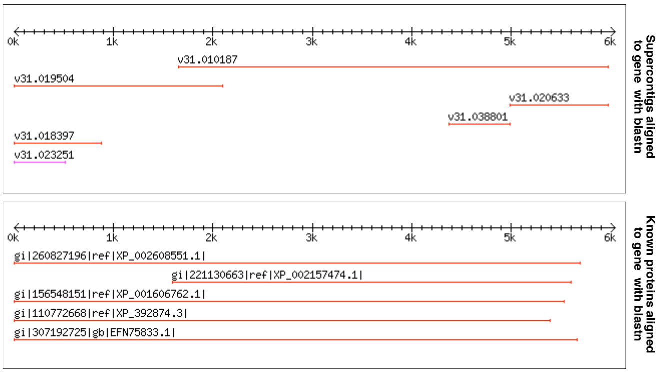

Gene_05387

Supercontigs mapped to this gene

v31.000344+, v31.004514+, v31.016111+

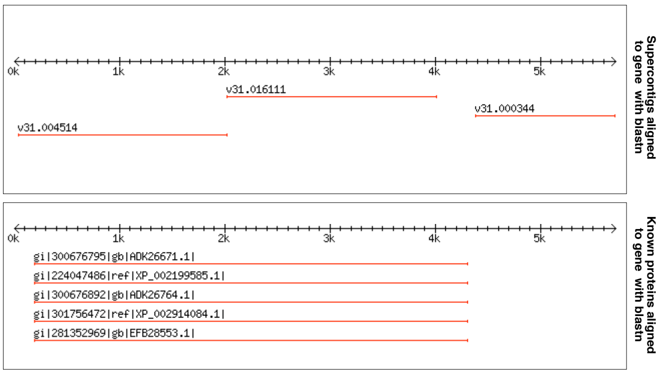

Gene_05437

Supercontigs mapped to this gene

v31.007254-, v31.009594-, v31.012884-, v31.014961+, v31.039044-

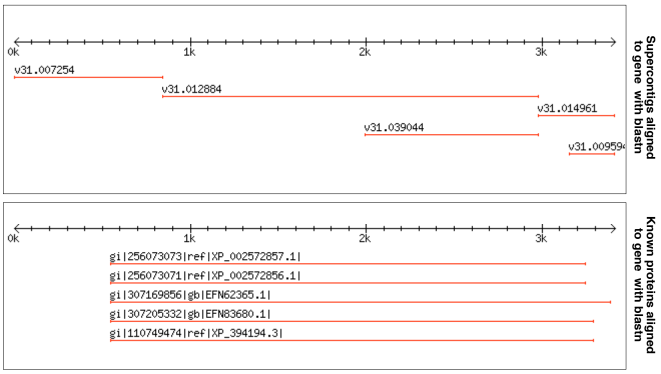

Gene_05452

Supercontigs mapped to this gene

v31.009177-, v31.018236+, v31.021870+

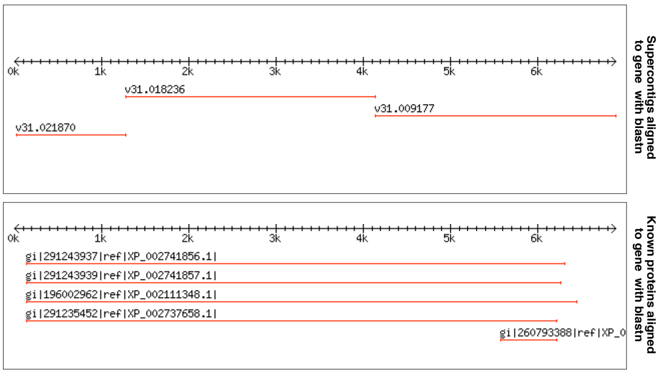

Gene_05467

Supercontigs mapped to this gene

v31.008004-, v31.010820-, v31.011353-, v31.033852+, v31.041658-

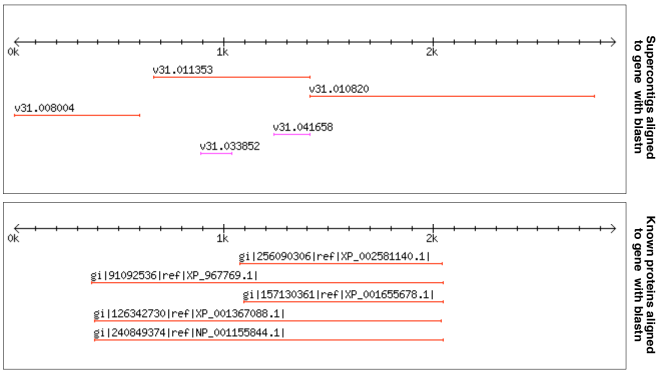

Gene_05456

Supercontigs mapped to this gene

v31.001112-, v31.004188+, v31.016001-, v31.020682-, v31.022787+, v31.026812+

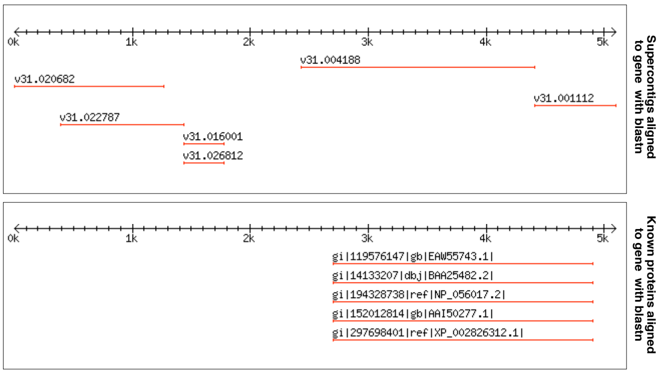

Gene_05530

Supercontigs mapped to this gene

v31.001231-, v31.004168+, v31.013427-, v31.018796-

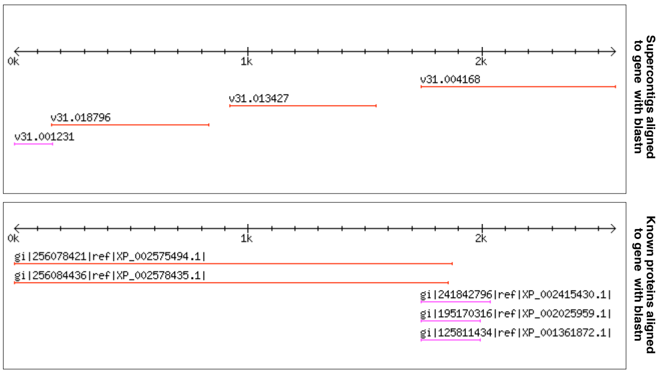

Gene_05540

Supercontigs mapped to this gene
v31.011730+, v31.012882-, v31.015134-, v31.018281-, v31.027393-

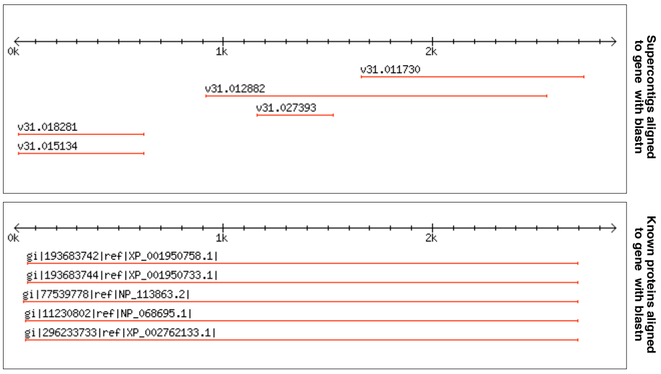

Gene_05553

Supercontigs mapped to this gene
v31.004299-, v31.006111+, v31.014446-

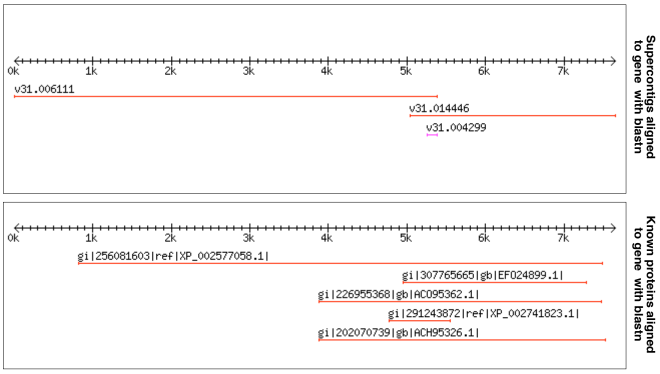

Gene_05548

Supercontigs mapped to this gene
v31.003449+, v31.003856-, v31.005159+, v31.026813+

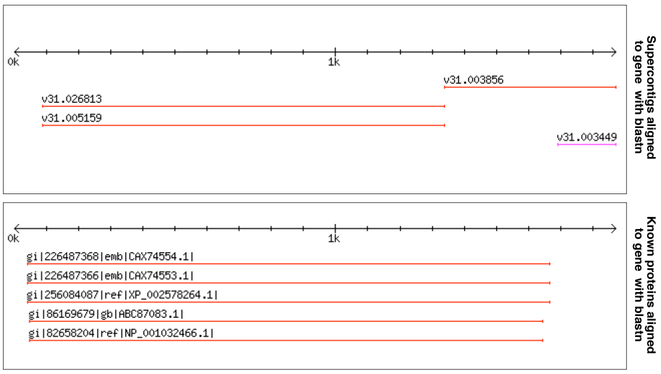

Gene_05587

Supercontigs mapped to this gene
v31.000973+, v31.002029+

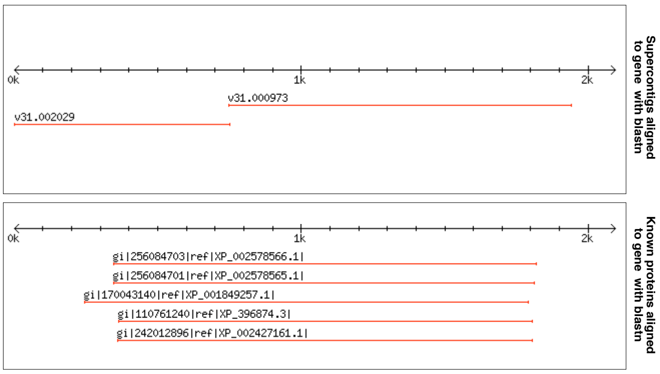

Gene_05613

Supercontigs mapped to this gene
v31.004963-, v31.007079+, v31.013951+, v31.015716-, v31.018520+, v31.029169+, v31.044057-

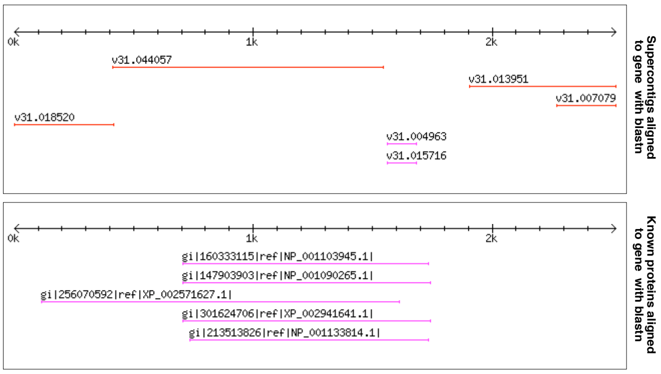

Gene_05655

Supercontigs mapped to this gene
v31.007599+, v31.012715-, v31.019865-, v31.020133-, v31.021025+, v31.024209+, v31.031543-, v31.035301+, v31.042532-

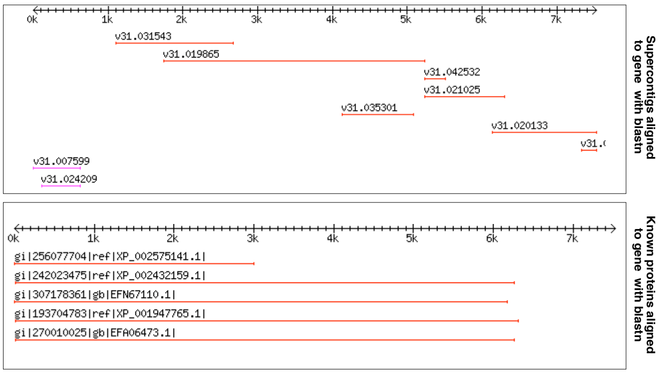

Gene_05634

Supercontigs mapped to this gene
v31.000446+, v31.006687+

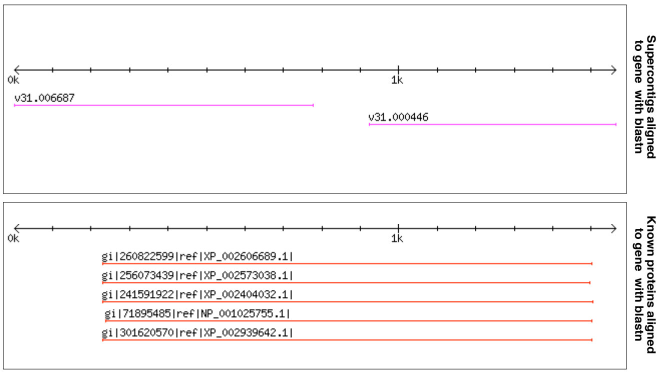

Gene_05672

Supercontigs mapped to this gene
v31.004210+, v31.011259-

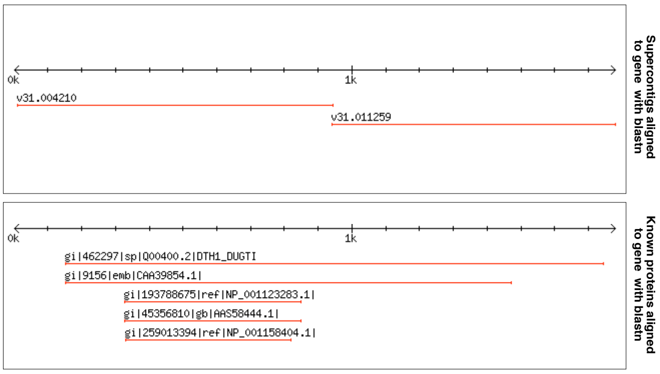

Gene_05705

Supercontigs mapped to this gene
v31.000438+, v31.003750+, v31.004526-

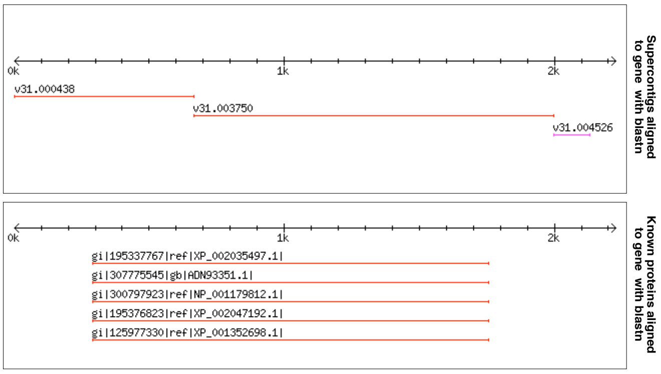

Gene_05741

Supercontigs mapped to this gene
v31.005560+, v31.010451-

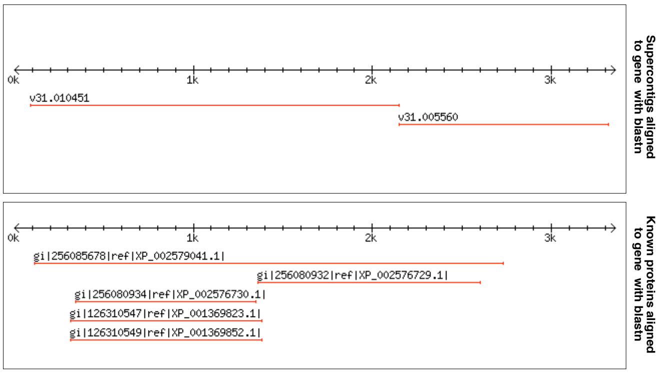

Gene_05732

Supercontigs mapped to this gene
v31.014570-, v31.024673+, v31.026299+

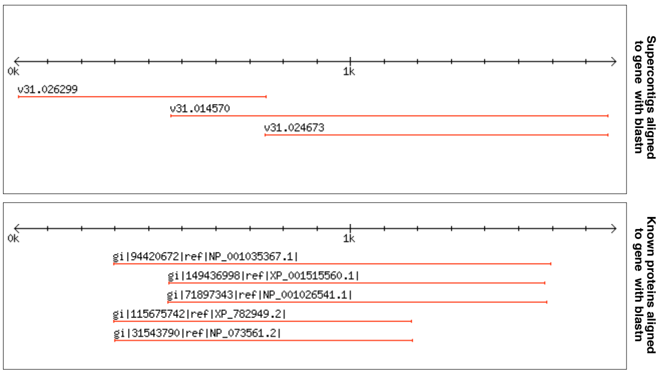

Gene_05744

Supercontigs mapped to this gene
v31.005764+, v31.007578+, v31.027994-

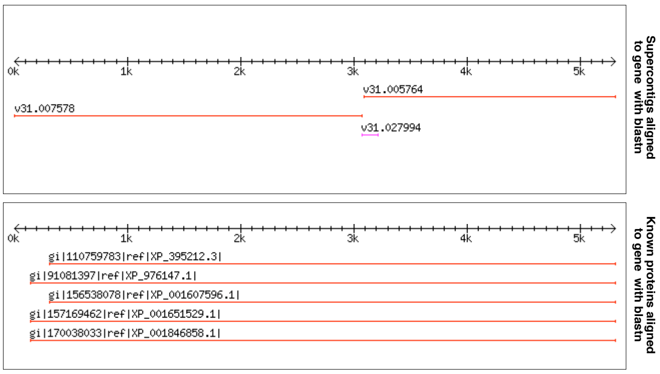

Gene_05779

Supercontigs mapped to this gene
v31.000223-, v31.011189+

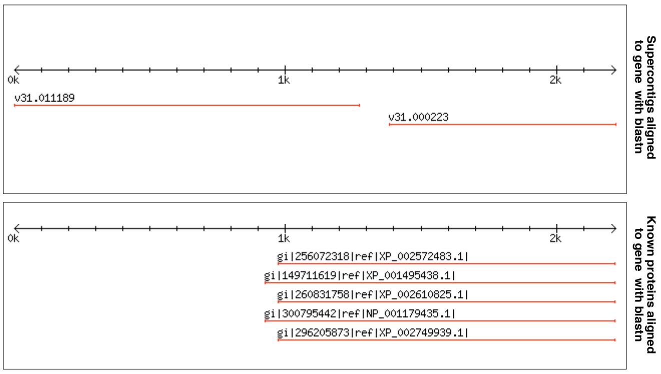

Gene_05785

Supercontigs mapped to this gene
v31.002087-, v31.007035+, v31.007035-

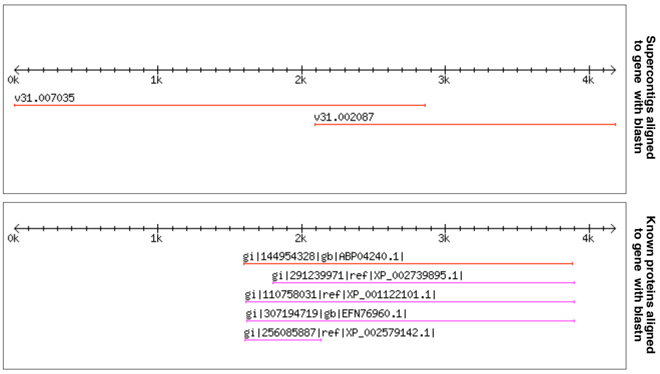

Gene_05780

Supercontigs mapped to this gene
v31.000234-, v31.003591-

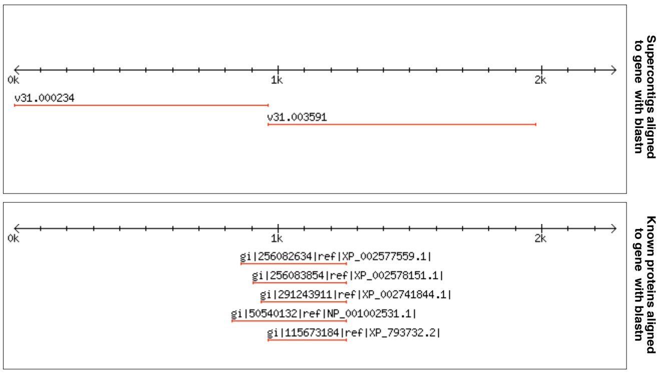

Gene_00580

Supercontigs mapped to this gene
v31.001963-, v31.004010+, v31.015622+, v31.024243+, v31.029778-

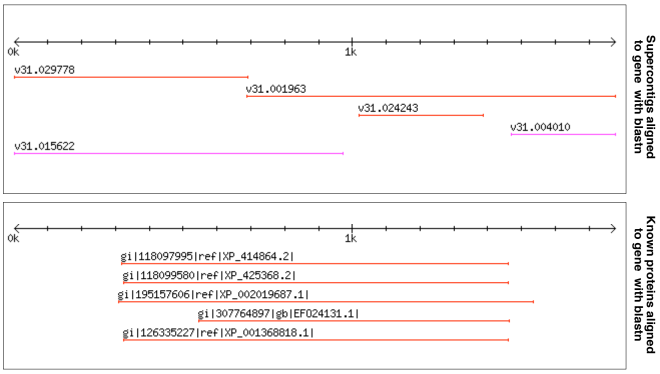

Gene_05825

Supercontigs mapped to this gene

v31.002463+, v31.003906-

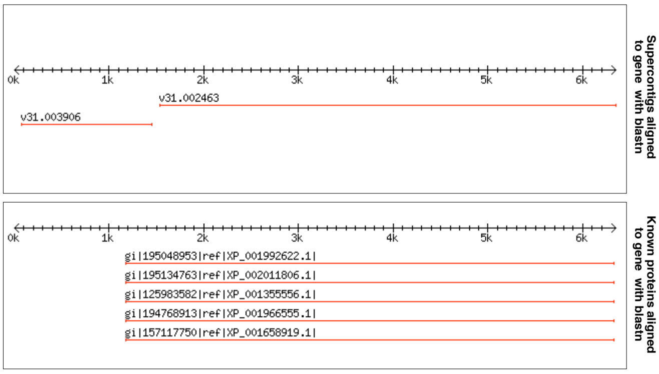

Gene_05896

Supercontigs mapped to this gene

v31.010040-, v31.015082-, v31.017098+

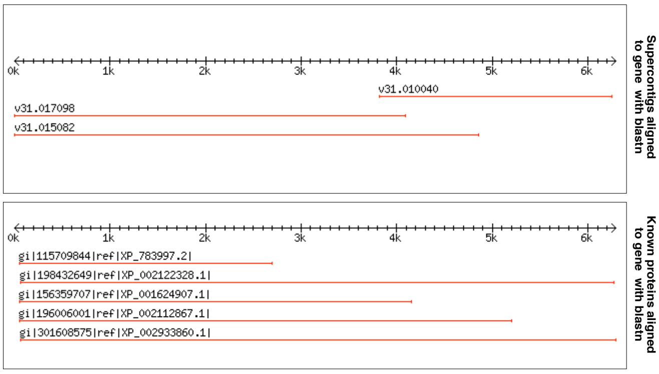

Gene_00584

Supercontigs mapped to this gene

v31.000889-, v31.003614+, v31.012084+, v31.026837+

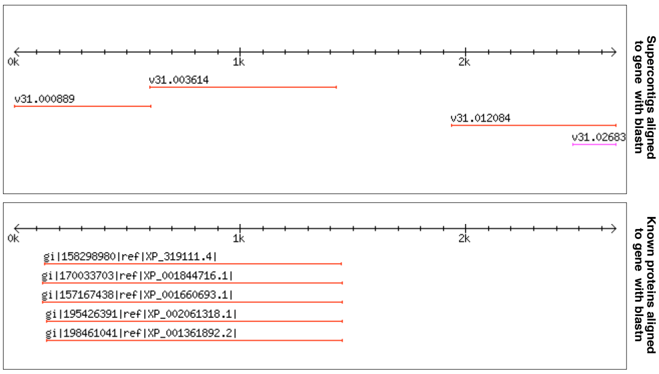

Gene_05905

Supercontigs mapped to this gene

v31.000453-, v31.004705+, v31.026402-, v31.031387+, v31.039710+

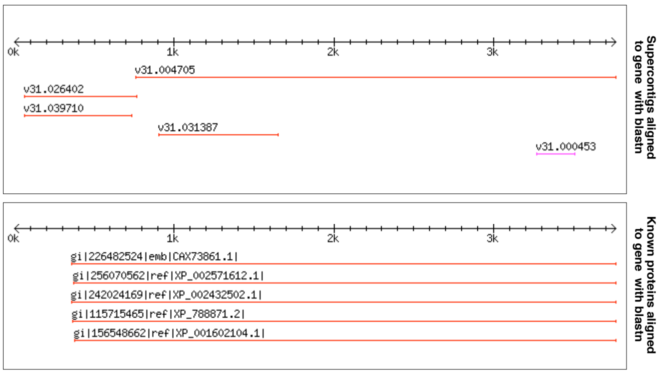

Gene_05923

Supercontigs mapped to this gene

v31.009286-, v31.014709-, v31.021023-, v31.027131-

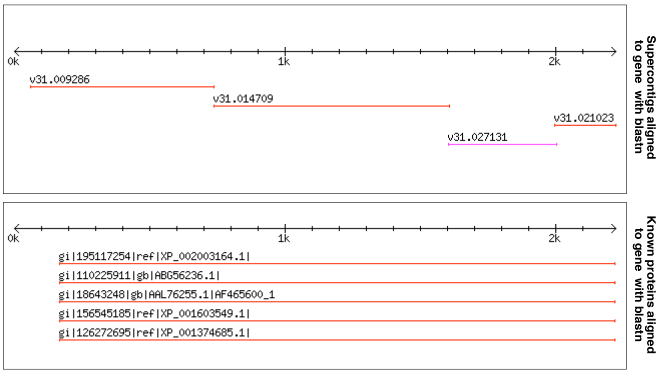

Gene_06025

Supercontigs mapped to this gene

v31.000843+, v31.003246+

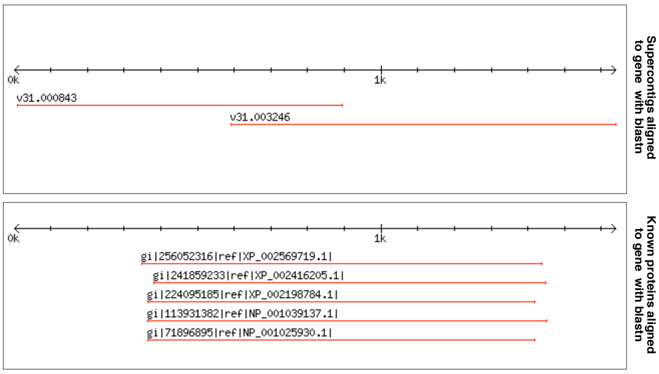

Gene_05959

Supercontigs mapped to this gene

v31.010230-, v31.020603-, v31.020629-, v31.023626-, v31.061801-

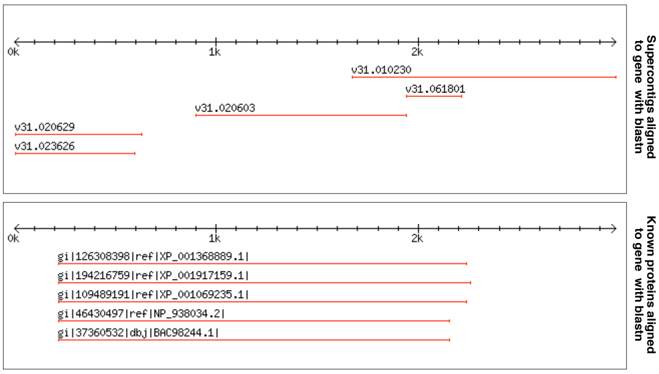

Gene_06113

Supercontigs mapped to this gene

v31.001106+, v31.006052-

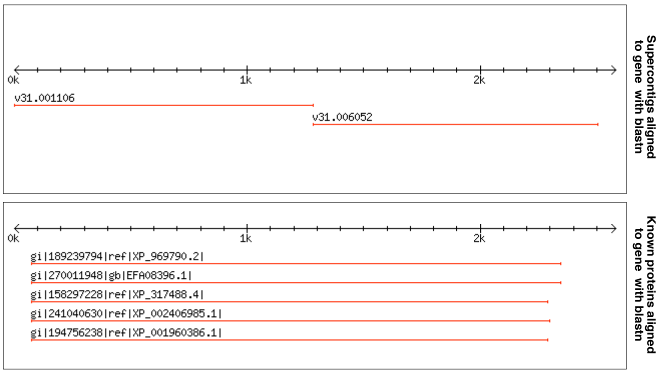

Gene_06146

Supercontigs mapped to this gene

v31.000723+, v31.011713-, v31.016170-

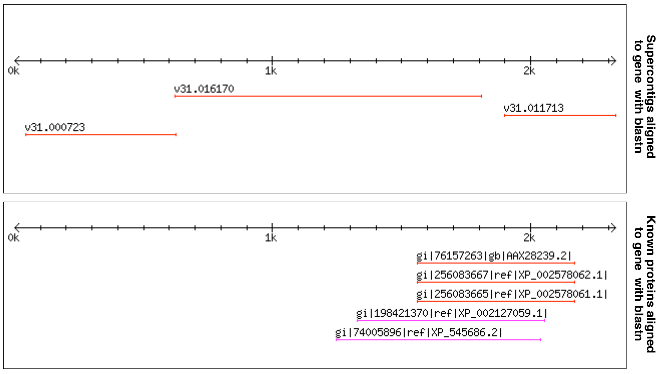

Gene_06200

Supercontigs mapped to this gene

v31.003636+, v31.014514-

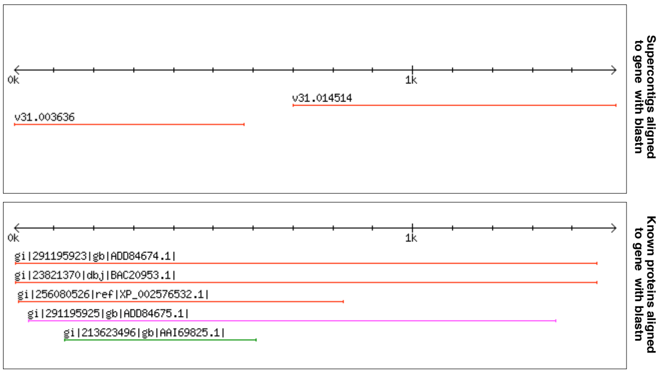

Gene_06186

Supercontigs mapped to this gene

v31.000005+, v31.000512+

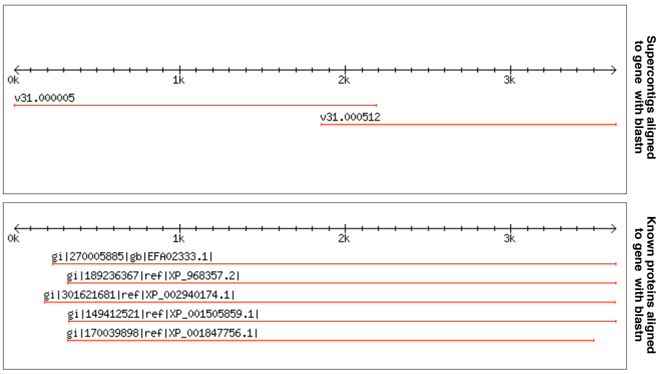

Gene_06207

Supercontigs mapped to this gene

v31.004437-, v31.005392-

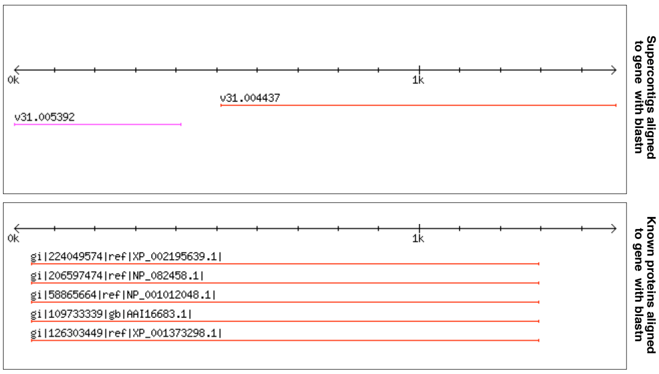

Gene_00623

Supercontigs mapped to this gene

v31.008930+, v31.014285-, v31.020537-, v31.024324+, v31.035218+

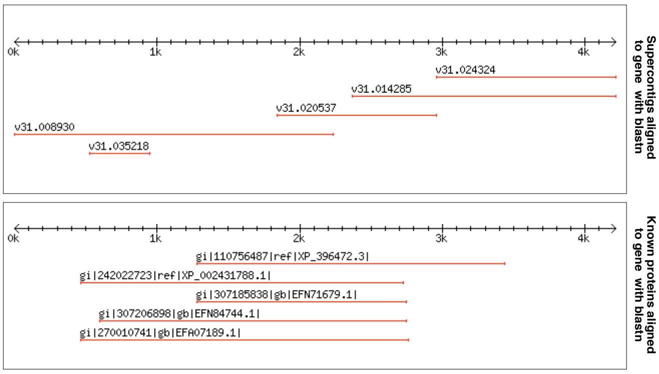

Gene_06248

Supercontigs mapped to this gene

v31.005231+, v31.012878-, v31.018755-, v31.024627-, v31.053692+

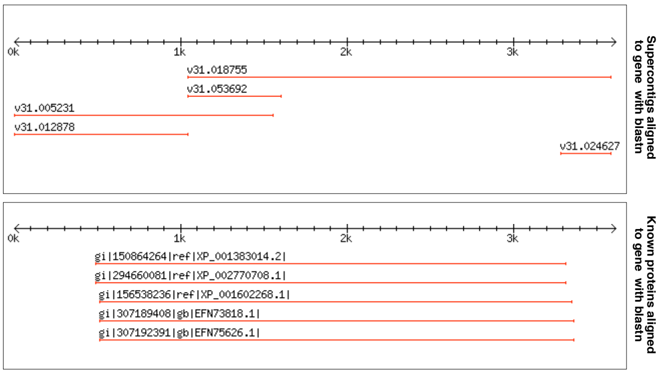

Gene_06237

Supercontigs mapped to this gene

v31.000030-, v31.005452+

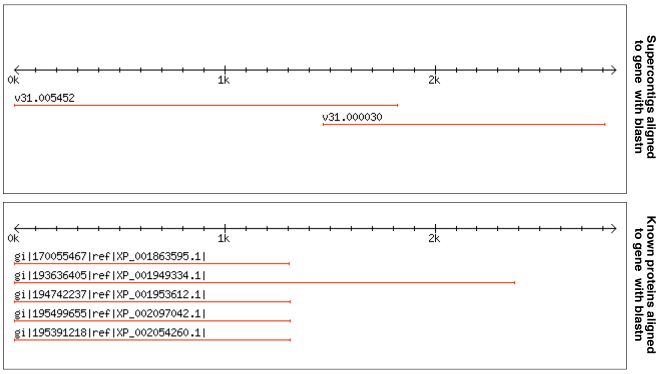

Gene_06322

Supercontigs mapped to this gene

v31.000427+, v31.000521-, v31.000804-, v31.001402+, v31.002151+, v31.003030-, v31.004503+, v31.005489-, v31.008594+, v31.053550+

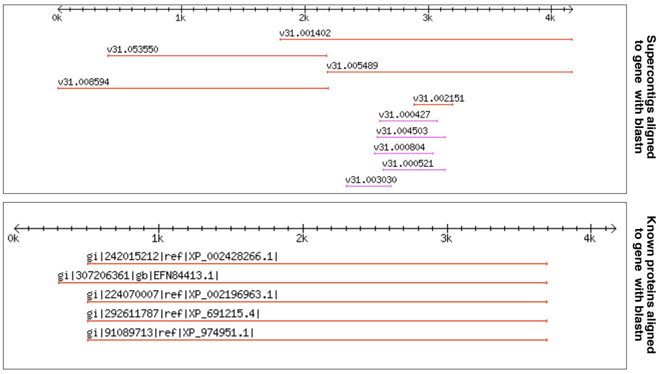

Gene_06390

Supercontigs mapped to this gene
v31.002342+, v31.013443-

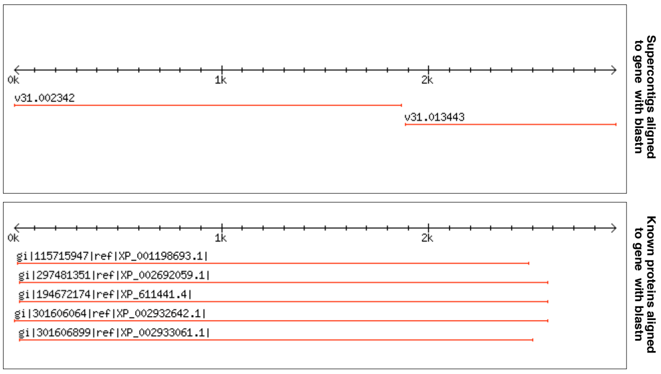

Gene_06466

Supercontigs mapped to this gene
v31.003398+, v31.007081+, v31.029613-, v31.032876+, v31.035059+, v31.044803-

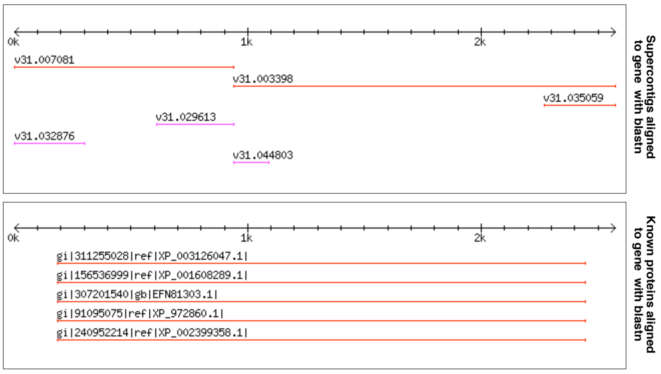

Gene_06409

Supercontigs mapped to this gene
v31.004282+, v31.009184-, v31.029821+

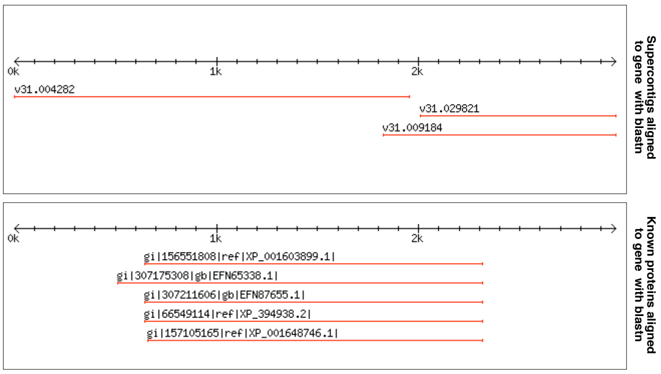

Gene_06496

Supercontigs mapped to this gene
v31.013196+, v31.017795+, v31.019325-, v31.033188+, v31.038146-

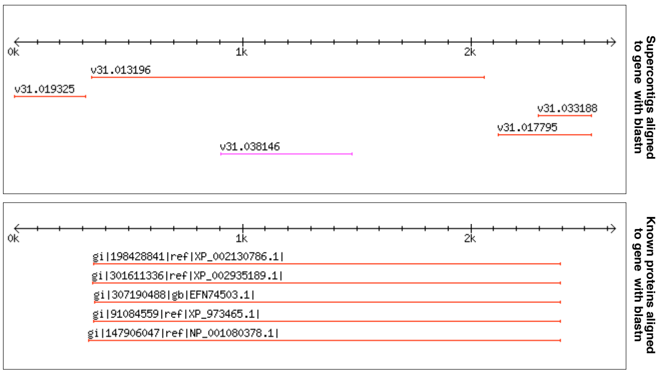

Gene_06523

Supercontigs mapped to this gene
v31.013717-, v31.017944+, v31.024749+, v31.027784+, v31.030858+

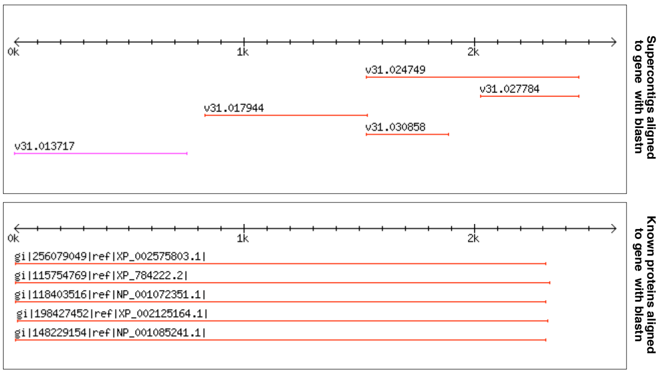

Gene_00662

Supercontigs mapped to this gene
v31.011446-, v31.017597+, v31.026268+, v31.040595+, v31.048661+

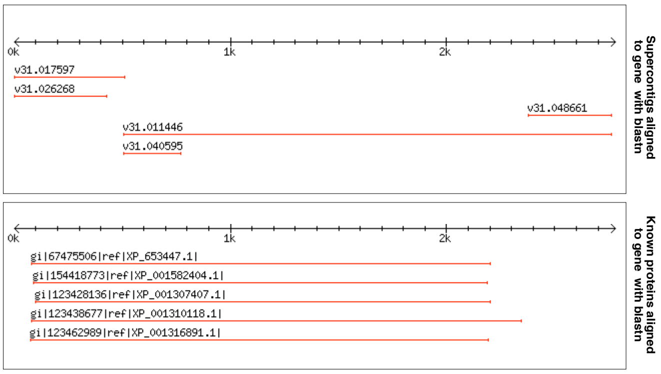

Gene_06540

Supercontigs mapped to this gene
v31.004848+, v31.008398+, v31.008398-, v31.040749+

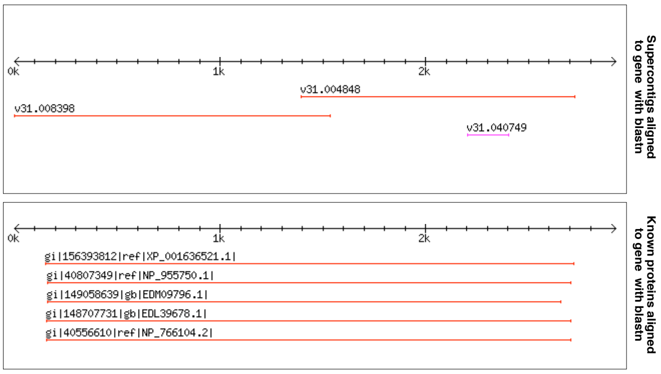

Gene_06621

Supercontigs mapped to this gene
v31.001207-, v31.003196+

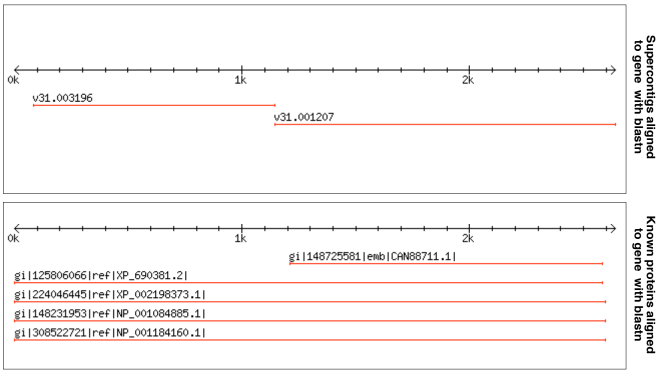

Gene_06625

Supercontigs mapped to this gene
v31.000221+, v31.001305+, v31.002624+, v31.002722-, v31.003068+, v31.003829-, v31.007549+, v31.026523-

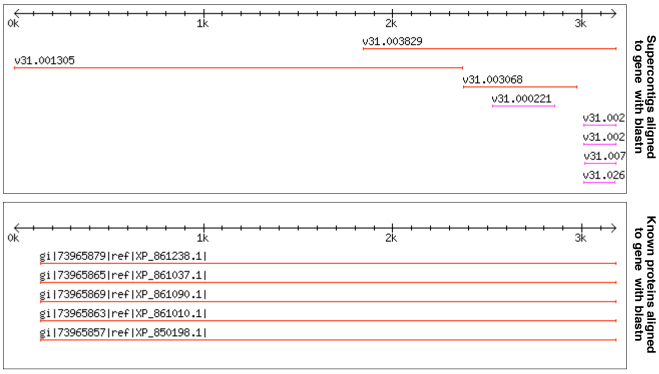

Gene_00067

Supercontigs mapped to this gene
v31.012229+, v31.013204+, v31.017858-, v31.053649+

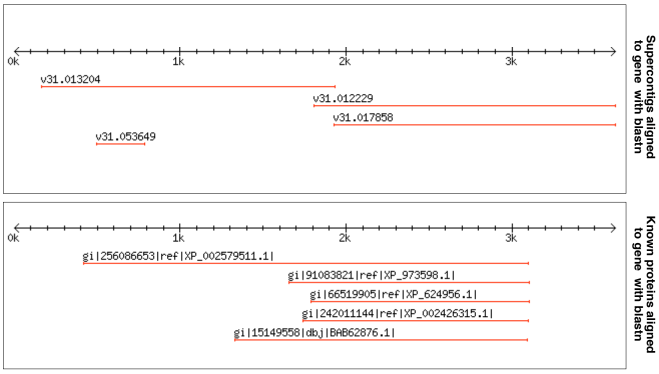

Gene_06657

Supercontigs mapped to this gene
v31.001010+, v31.002406+

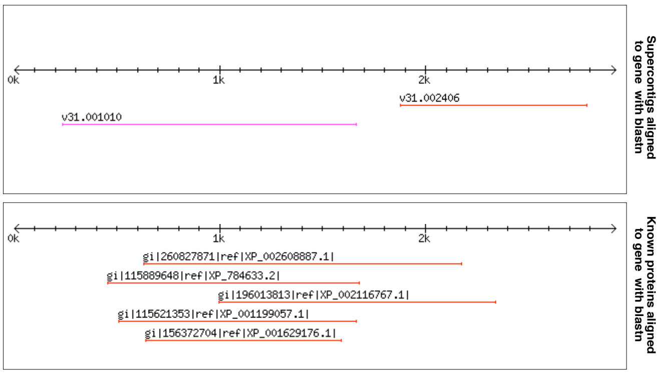

Gene_00670

Supercontigs mapped to this gene
v31.004912-, v31.005538+

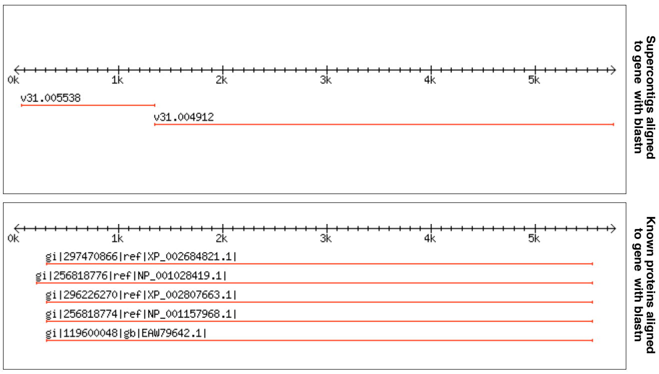

Gene_06700

Supercontigs mapped to this gene
v31.000381+, v31.011001+

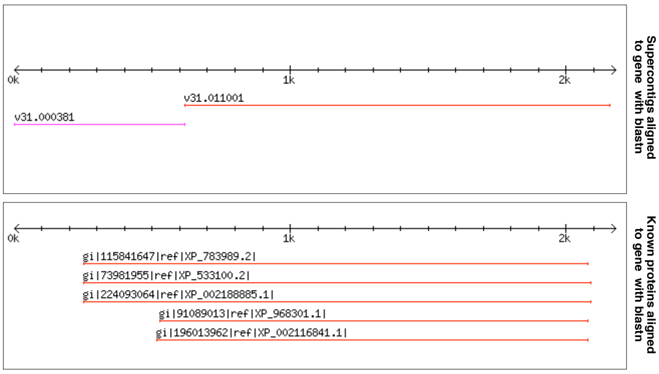

Gene_06737

Supercontigs mapped to this gene
v31.008428+, v31.017717+, v31.021043-, v31.026638+, v31.028911+, v31.030598-, v31.035133-, v31.044223+, v31.055957+

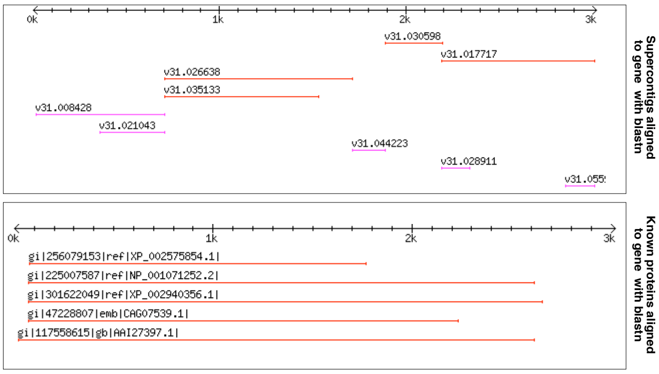

Gene_06734

Supercontigs mapped to this gene
v31.001292+, v31.019652+, v31.021817+, v31.029424-

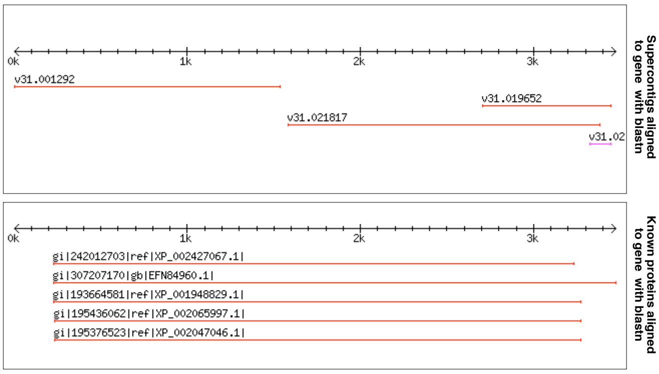

Gene_06774

Supercontigs mapped to this gene
v31.000377-, v31.003350+

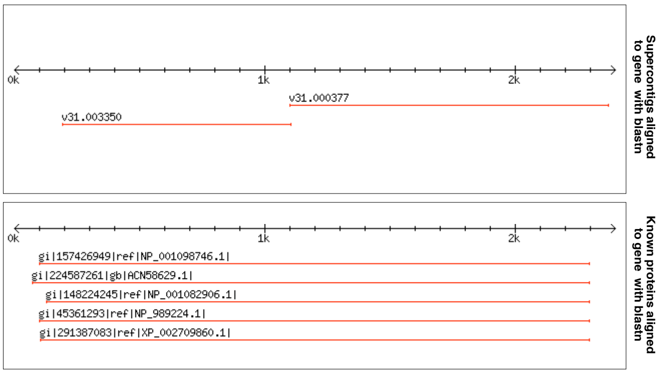

Gene_06882

Supercontigs mapped to this gene
v31.002902-, v31.011851-, v31.021918-

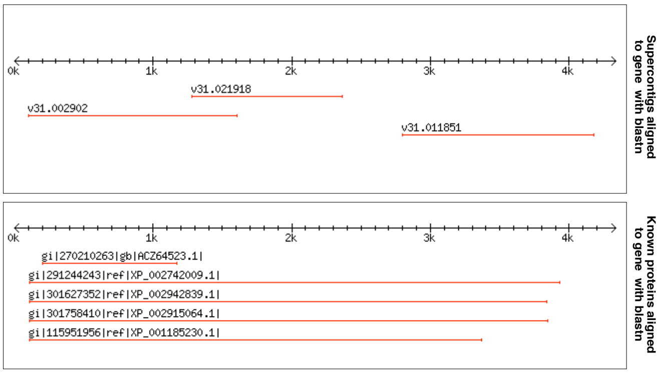

Gene_06946

Supercontigs mapped to this gene
v31.000418+, v31.000859+, v31.004779+, v31.011230+

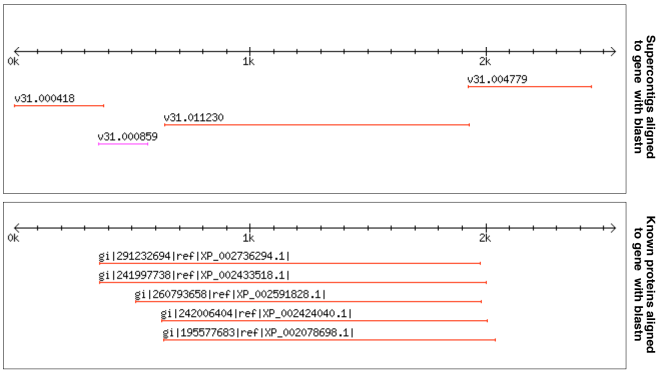

Gene_06919

Supercontigs mapped to this gene
v31.002465+, v31.009404-

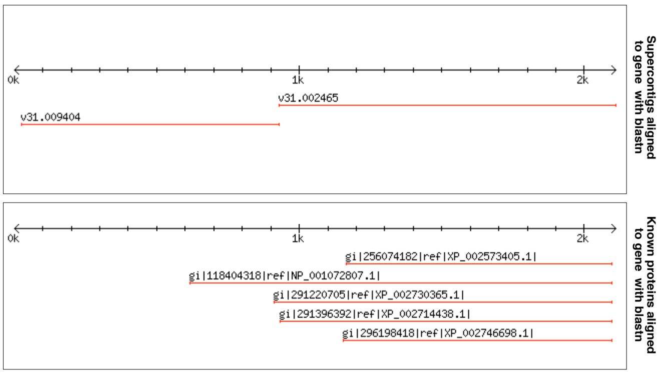

Gene_06954

Supercontigs mapped to this gene
v31.001384-, v31.003949+, v31.004877-

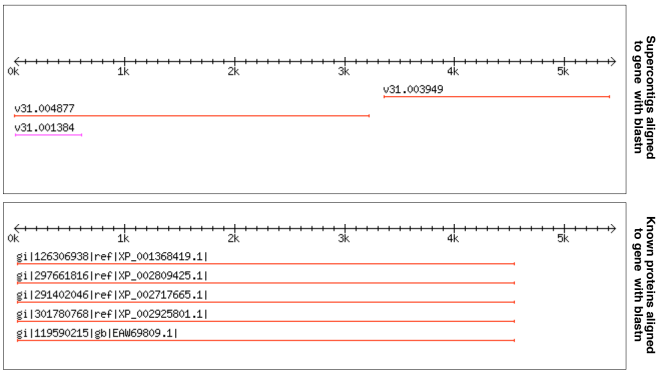

Gene_06970

Supercontigs mapped to this gene
v31.006357+, v31.012321+, v31.017647+, v31.019065+, v31.030335-

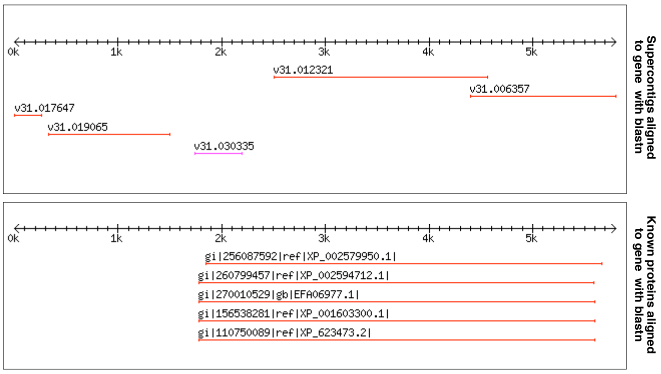

Gene_06992

Supercontigs mapped to this gene
v31.004578+, v31.009882+, v31.015539+, v31.029122+

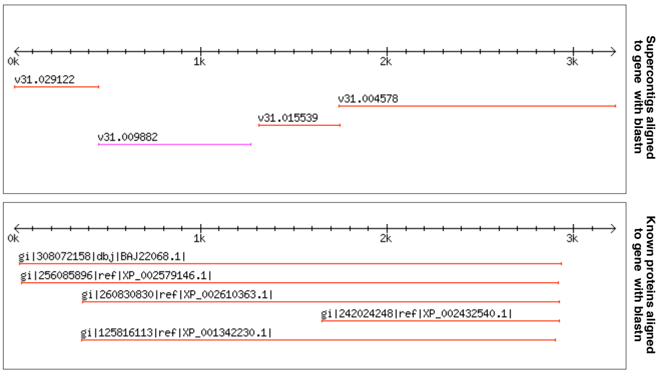

Gene_00698

Supercontigs mapped to this gene
v31.000736-, v31.008529+, v31.015912-

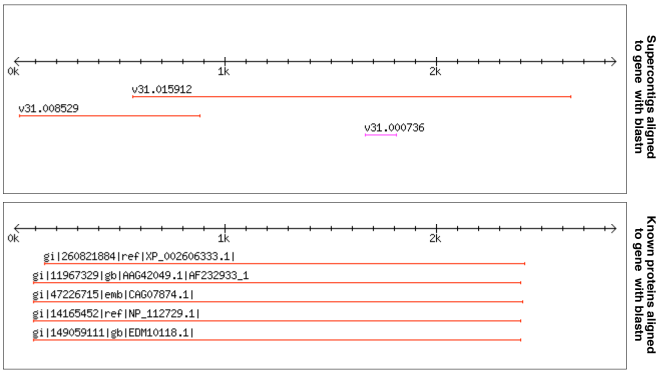

Gene_07001

Supercontigs mapped to this gene
v31.001227+, v31.003535+, v31.015577-

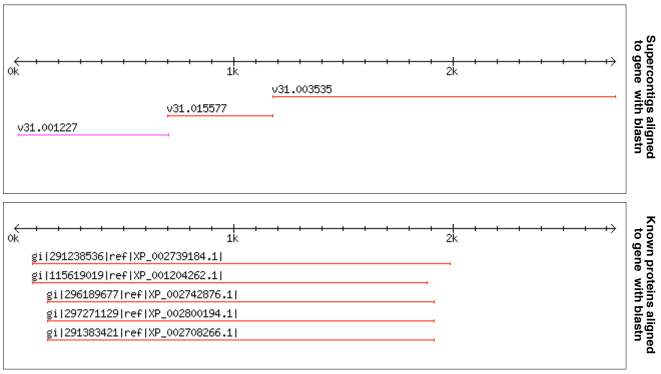

Gene_07006

Supercontigs mapped to this gene
v31.006299-, v31.008146-, v31.026218-

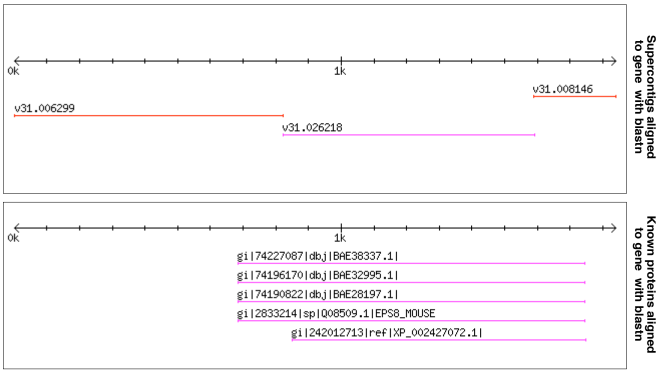

Gene_07050

Supercontigs mapped to this gene
v31.002275-, v31.010463-

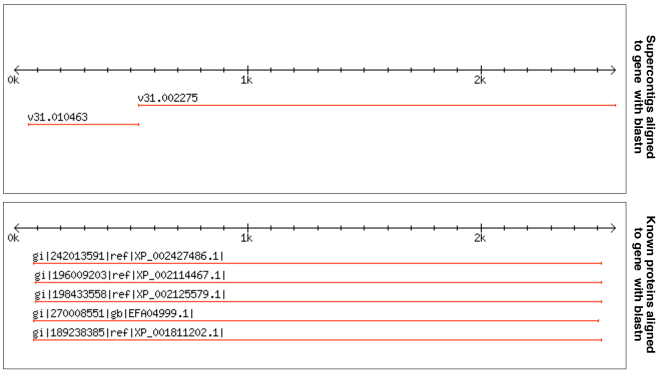

Gene_00701

Supercontigs mapped to this gene
v31.004407+, v31.006238-, v31.016792+, v31.021194+

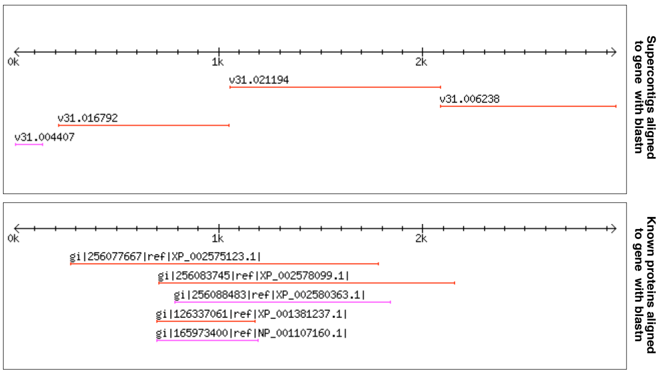

Gene_07057

Supercontigs mapped to this gene
v31.000218+, v31.001563-

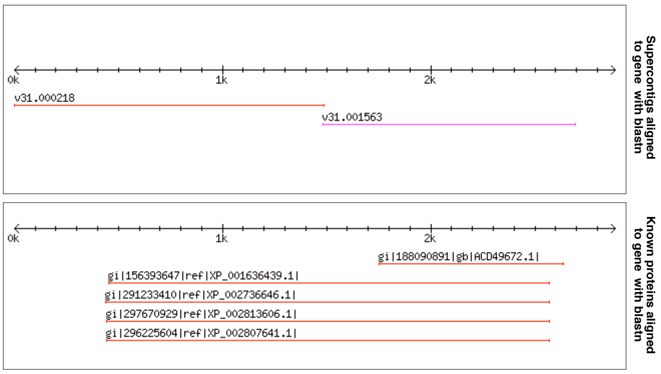

Gene_07075

Supercontigs mapped to this gene
v31.018898-, v31.020093-

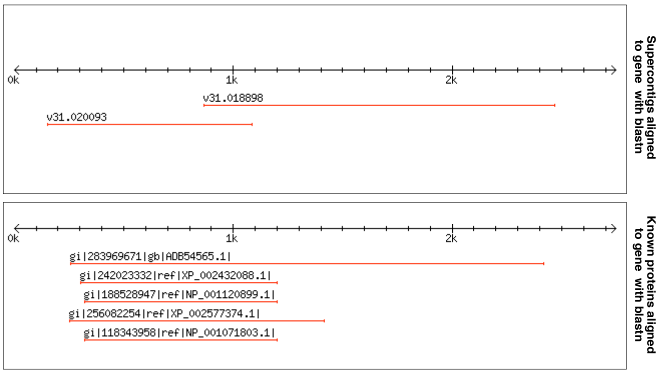

Gene_07080

Supercontigs mapped to this gene
v31.003013+, v31.003957+, v31.011764-

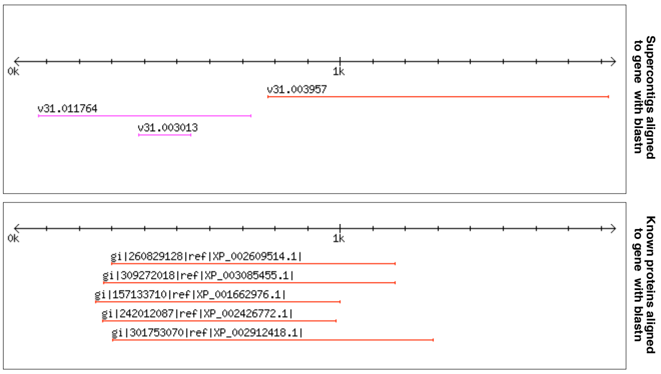

Gene_07076

Supercontigs mapped to this gene
v31.001012-, v31.003510+

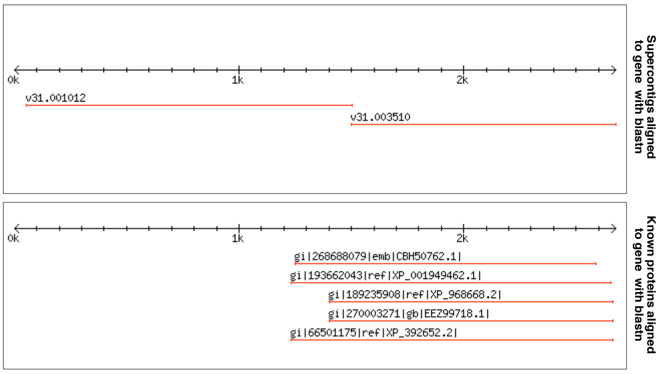

Gene_07088

Supercontigs mapped to this gene
v31.006690-, v31.010984+, v31.012428-, v31.018588+,
v31.022894+, v31.026953+, v31.028630+, v31.029181-

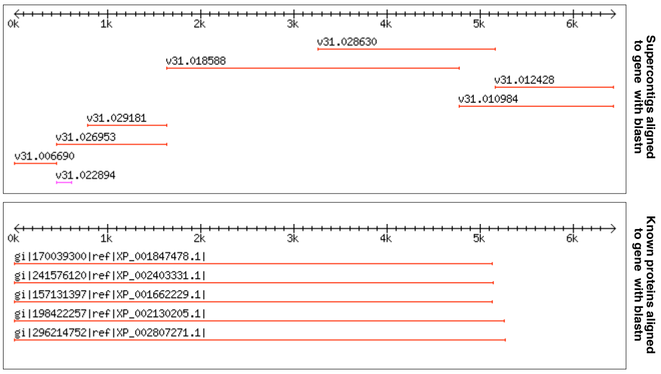

Gene_07098

Supercontigs mapped to this gene

v31.007246-, v31.018634+, v31.032945+, v31.054782-

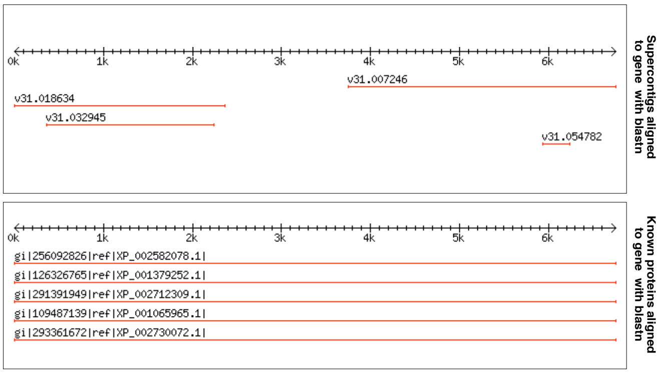

Gene_00715

Supercontigs mapped to this gene

v31.001739-, v31.018141+

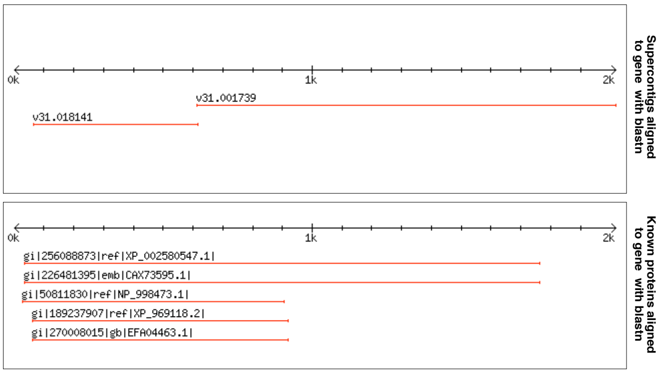

Gene_07125

Supercontigs mapped to this gene

v31.002343+, v31.007804-

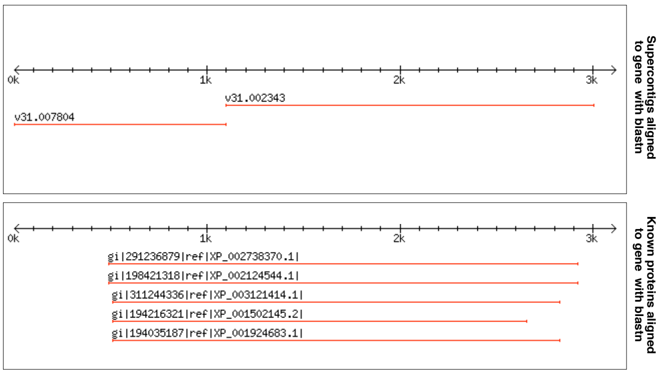

Gene_07169

Supercontigs mapped to this gene

v31.000405+, v31.007689+, v31.018098+

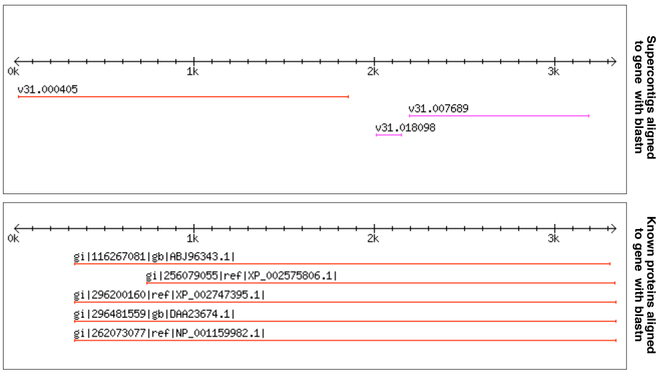

Gene_07175

Supercontigs mapped to this gene

v31.004629-, v31.005468-

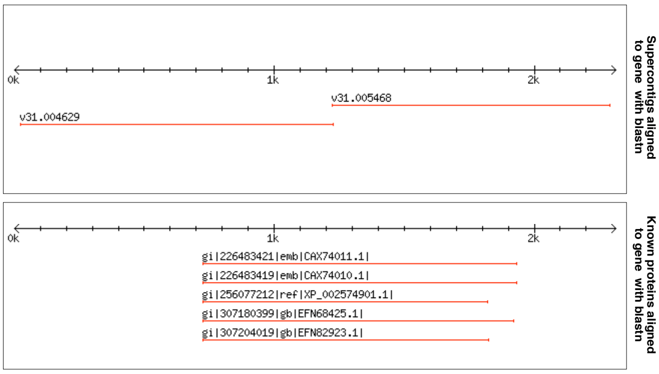

Gene_00730

Supercontigs mapped to this gene

v31.001048-, v31.014553-

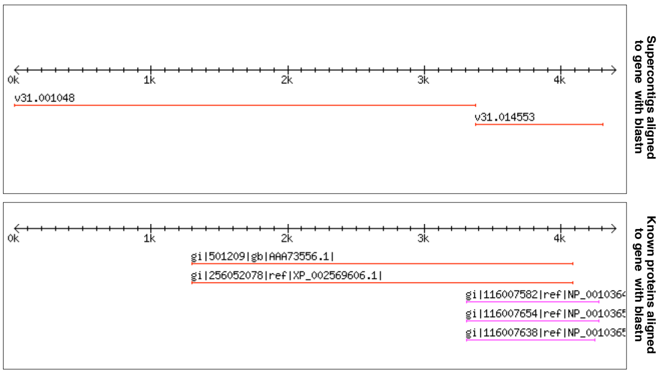

Gene_07202

Supercontigs mapped to this gene

v31.002655-, v31.007913-, v31.010683-

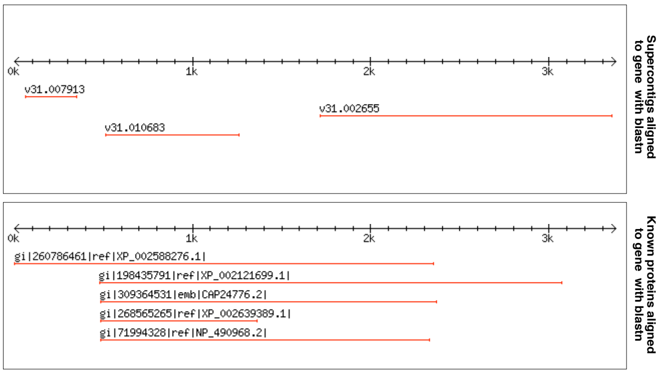

Gene_00736

Supercontigs mapped to this gene

v31.009223-, v31.011112-, v31.013095-, v31.017250-, v31.022022+, v31.023292+

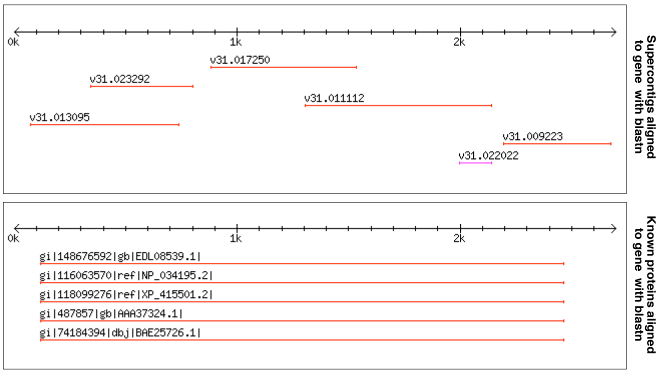

Gene_07371

Supercontigs mapped to this gene
v31.000581+, v31.001138-

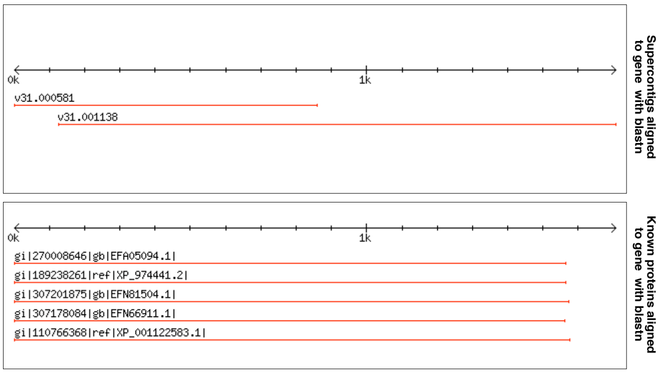

Gene_07426

Supercontigs mapped to this gene
v31.006651-, v31.012570-

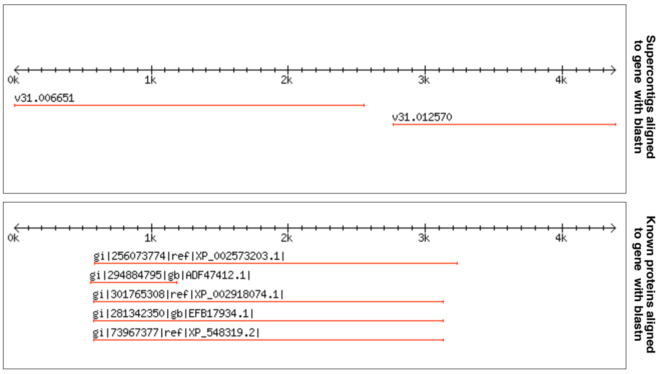

Gene_07400

Supercontigs mapped to this gene
v31.001801+, v31.003946-, v31.004775+

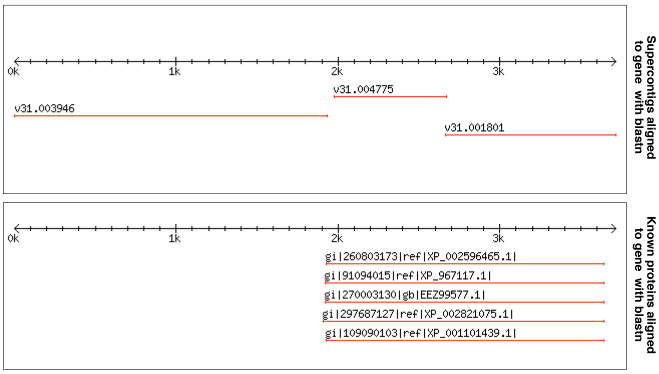

Gene_00743

Supercontigs mapped to this gene
v31.004214-, v31.017986-

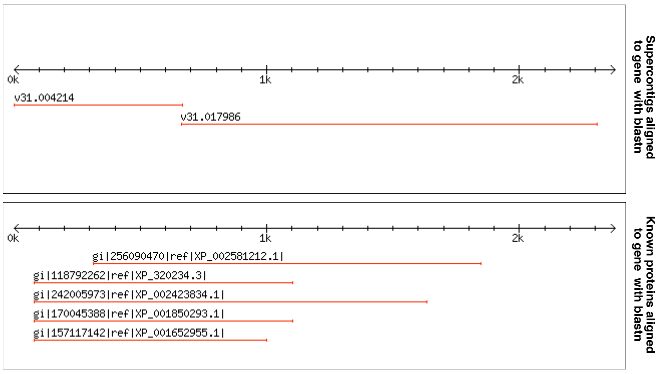

Gene_07563

Supercontigs mapped to this gene
v31.000109-, v31.002343+

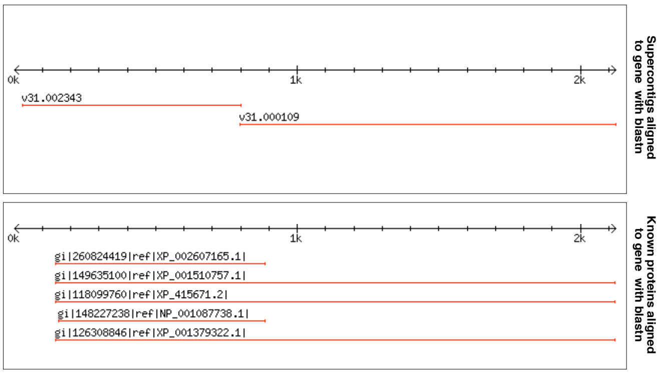

Gene_07601

Supercontigs mapped to this gene
v31.000435+, v31.000823+

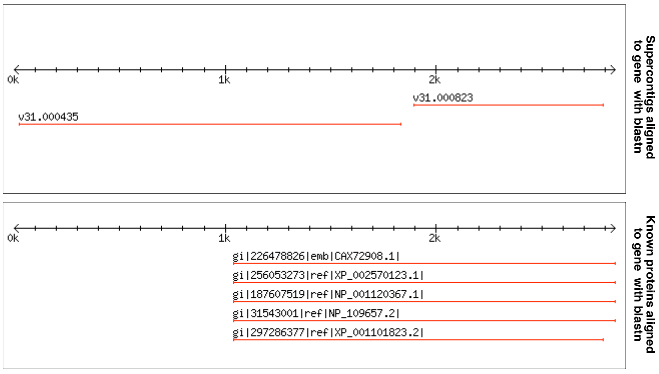

Gene_07590

Supercontigs mapped to this gene
v31.002563+, v31.015610+, v31.017180+, v31.025816+, v31.028471+, v31.030773-, v31.035617-, v31.056175-

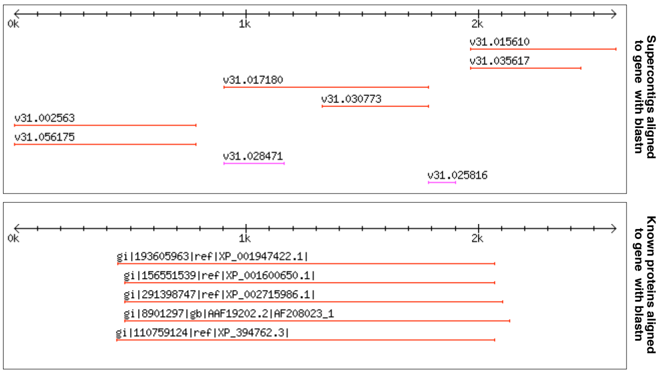

Gene_07602

Supercontigs mapped to this gene
v31.000202-, v31.000388-

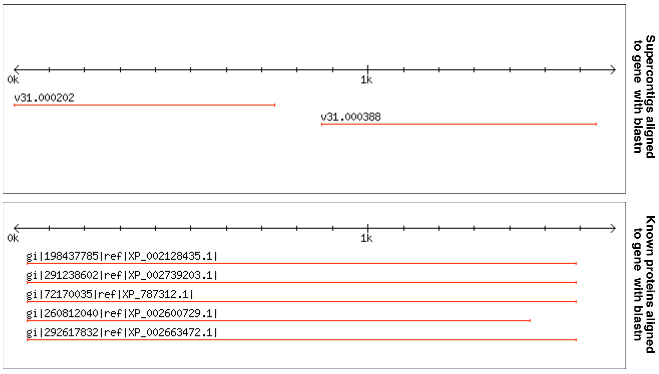

Gene_07603

Supercontigs mapped to this gene

v31.006234-, v31.008467+, v31.010357+, v31.012753-, v31.023897+, v31.036348+

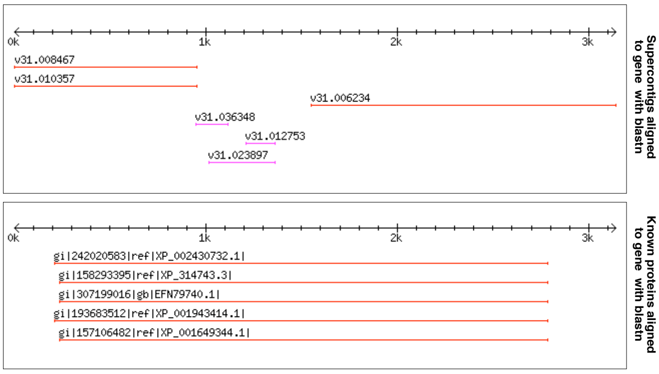

Gene_07668

Supercontigs mapped to this gene

v31.010583+, v31.011198+, v31.018219-, v31.028120-, v31.030766-

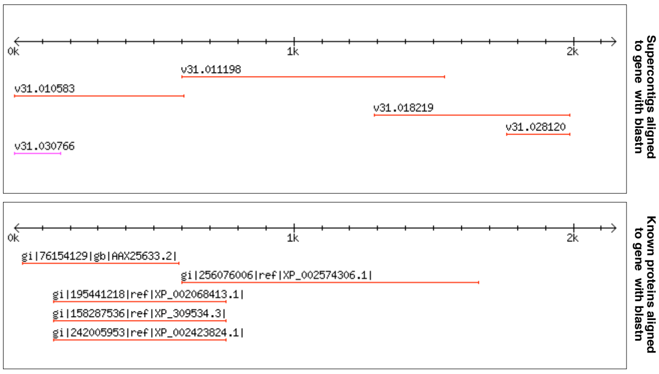

Gene_07604

Supercontigs mapped to this gene

v31.003186-, v31.006118-, v31.009124-, v31.023422-, v31.031277-

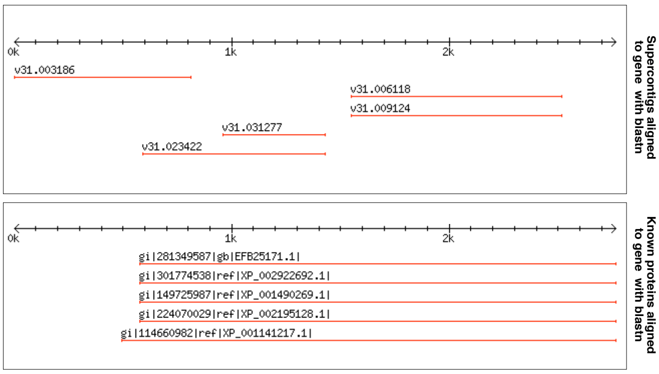

Gene_07701

Supercontigs mapped to this gene

v31.001109+, v31.014287+

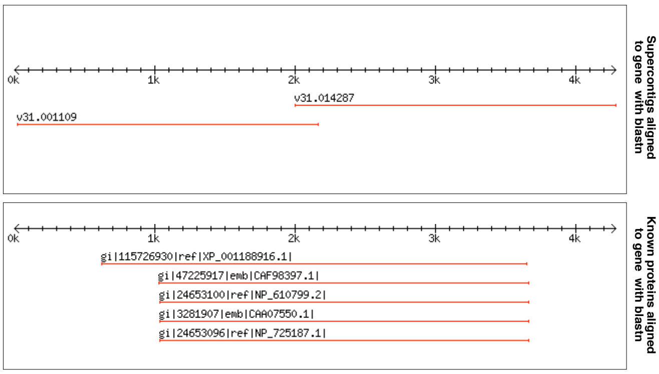

Gene_07735

Supercontigs mapped to this gene

v31.001621+, v31.004290-

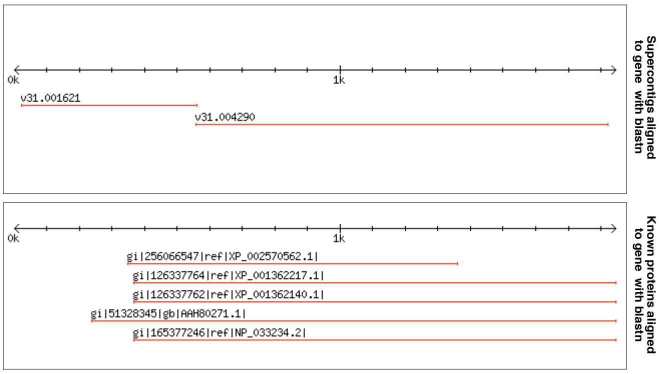

Gene_07795

Supercontigs mapped to this gene

v31.003859-, v31.012184+

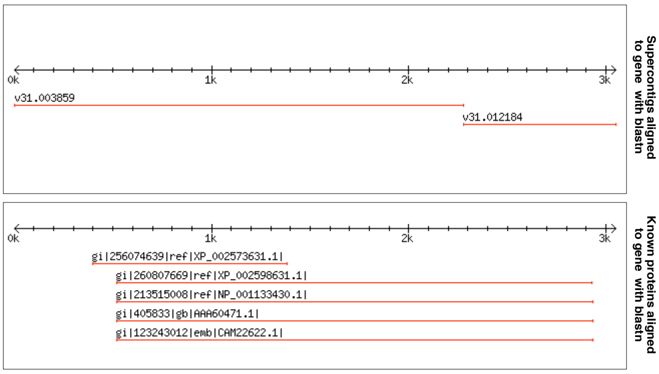

Gene_07778

Supercontigs mapped to this gene

v31.000192+, v31.010128-

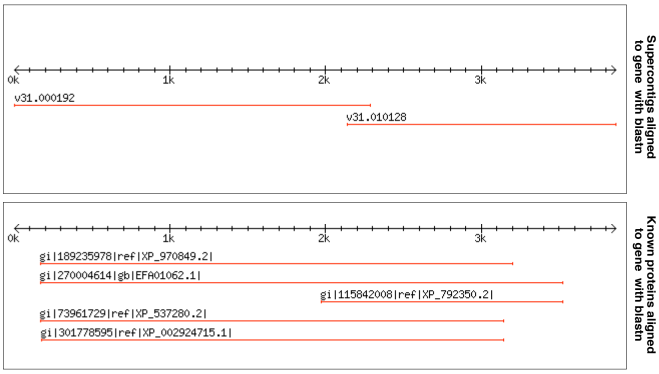

Gene_07815

Supercontigs mapped to this gene

v31.008079+, v31.012012-, v31.014142+, v31.015568+, v31.017314+, v31.018660+, v31.019588-, v31.021354+, v31.023883+

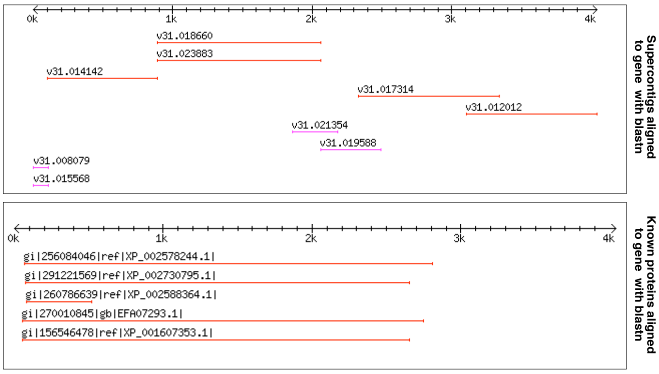

Gene_07823

Supercontigs mapped to this gene

v31.006906-, v31.007970-

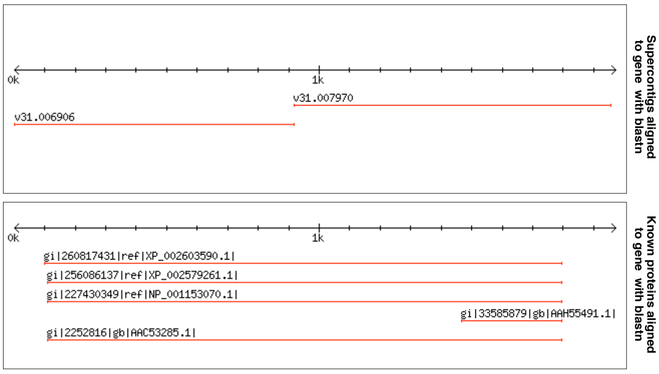

Gene_07979

Supercontigs mapped to this gene

v31.004348+, v31.027702-

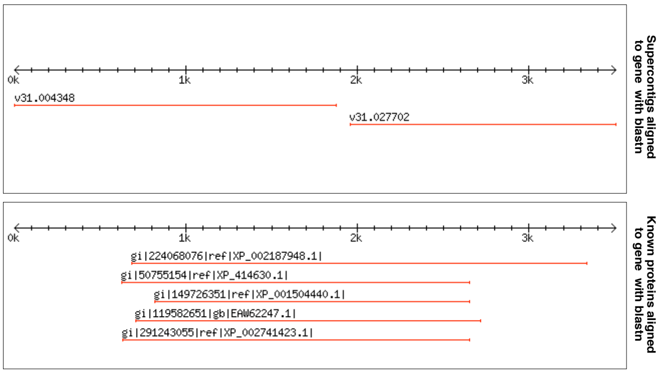

Gene_00795

Supercontigs mapped to this gene

v31.005335+, v31.007044+, v31.012168+, v31.014316-, v31.031573-, v31.032530+

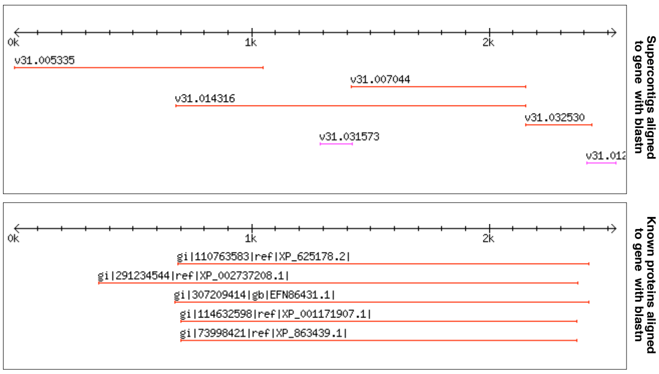

Gene_00080

Supercontigs mapped to this gene

v31.005868+, v31.016424-

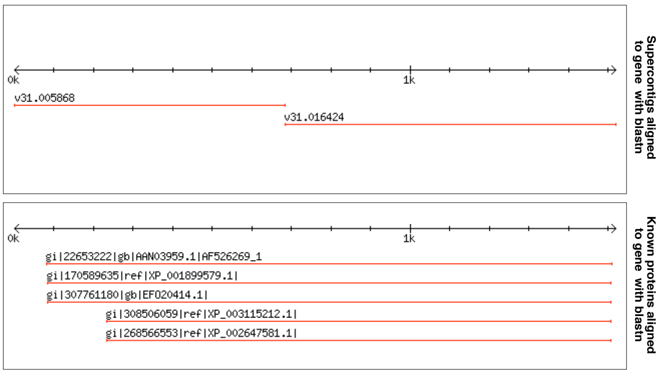

Gene_00802

Supercontigs mapped to this gene

v31.004716+, v31.006666+, v31.032424+, v31.041120+

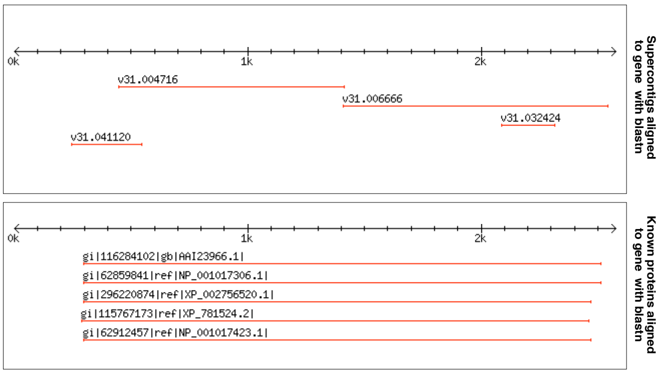

Gene_08227

Supercontigs mapped to this gene

v31.003069+, v31.010912+

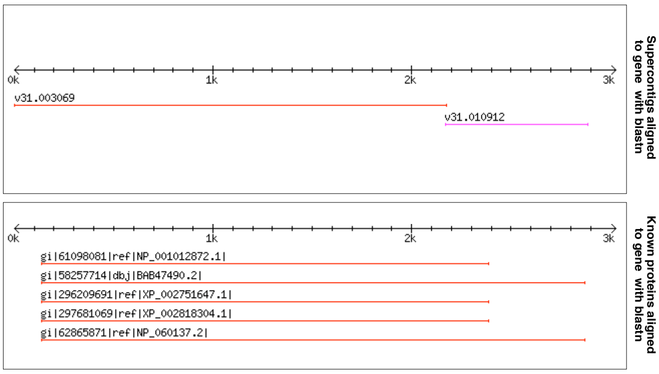

Gene_08087

Supercontigs mapped to this gene

v31.007155+, v31.009383+, v31.017082+, v31.021321-, v31.035441-

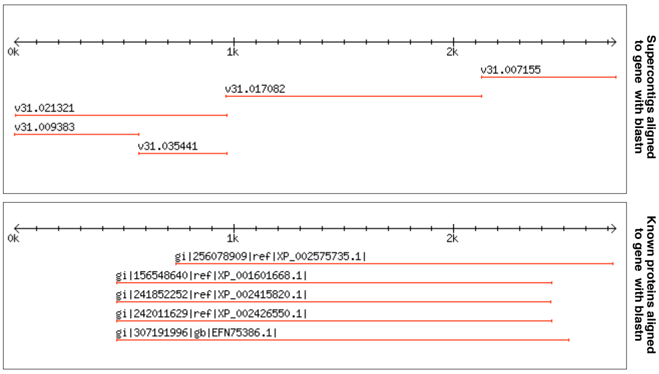

Gene_00827

Supercontigs mapped to this gene

v31.001187-, v31.008848-, v31.009799+, v31.011303+, v31.017586+, v31.027021-

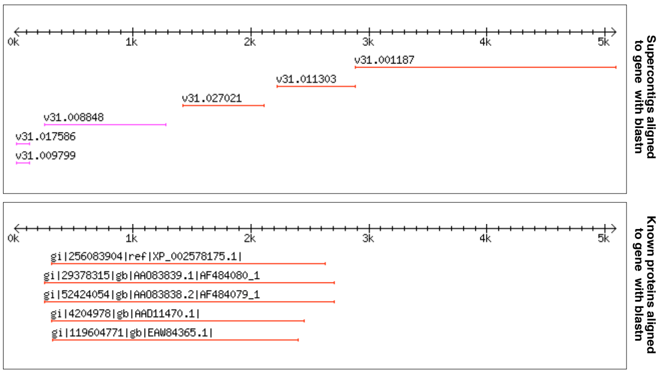

Gene_08396

Supercontigs mapped to this gene

v31.011451-, v31.011569-, v31.017437-, v31.033477+

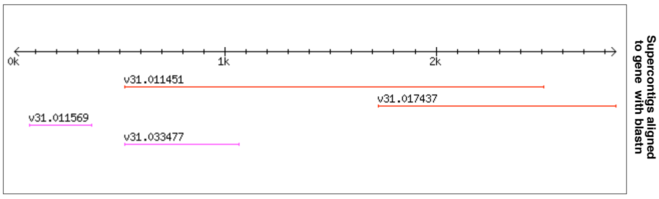

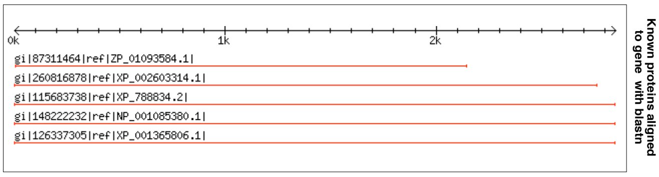

Gene_08409

Supercontigs mapped to this gene

v31.001548-, v31.010232+, v31.017531-, v31.035723-

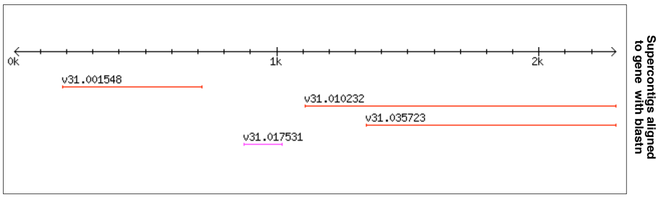

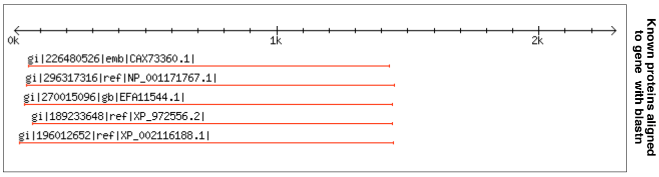

Gene_08406

Supercontigs mapped to this gene

v31.007739-, v31.009950+, v31.015355-, v31.021634-, v31.026958+

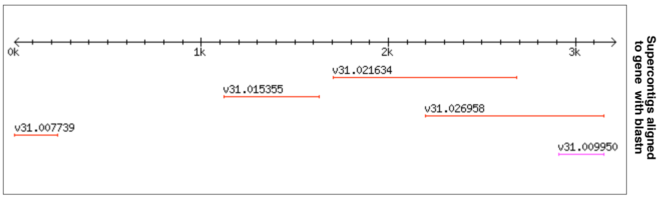

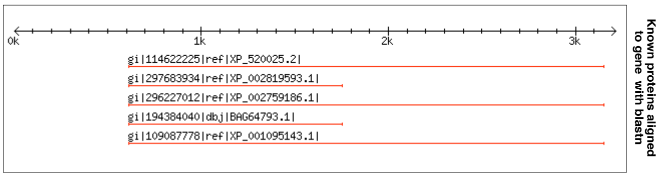

Gene_08428

Supercontigs mapped to this gene

v31.007698+, v31.010481-

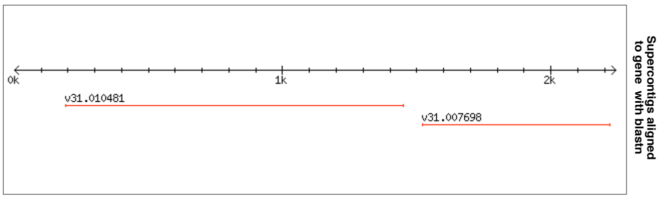

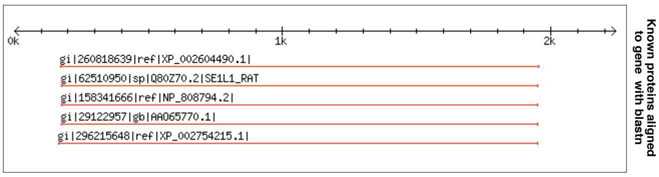

Gene_08466

Supercontigs mapped to this gene

v31.002981+, v31.016479+

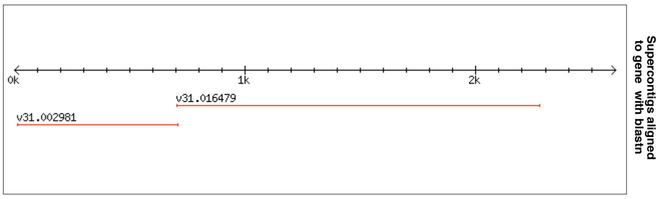

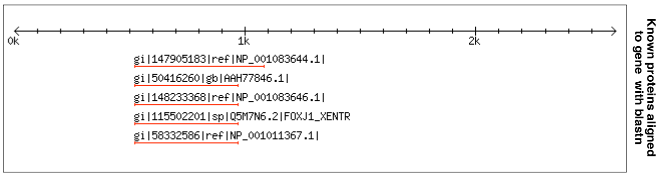

Gene_00857

Supercontigs mapped to this gene

v31.000906-, v31.002952-

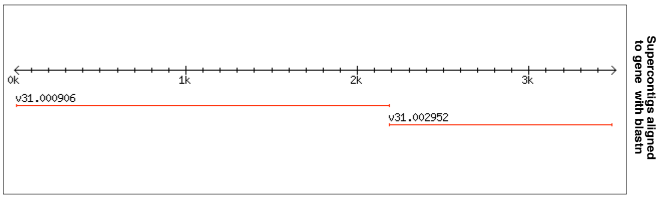

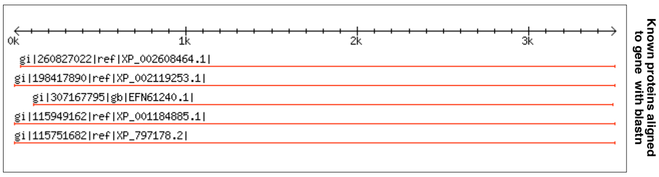

Gene_08560

Supercontigs mapped to this gene

v31.006093-, v31.012719+, v31.015404+

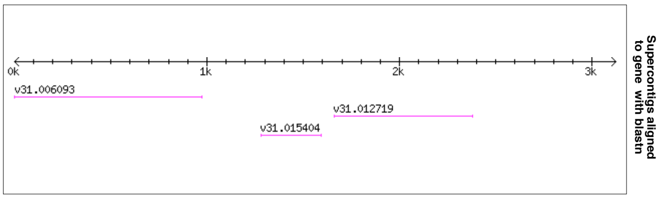

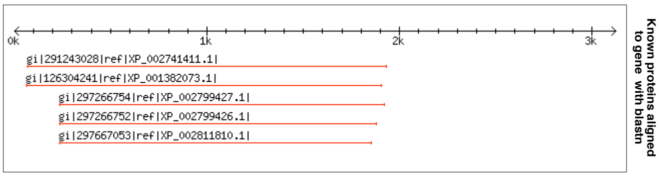

Gene_00878

Supercontigs mapped to this gene

v31.006866-, v31.010207-, v31.013327+, v31.024471-

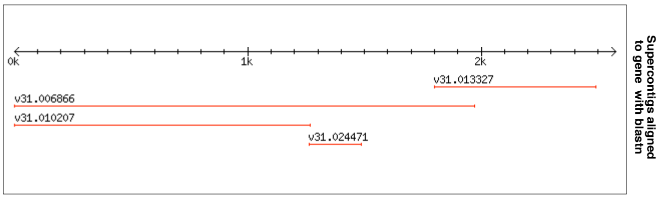

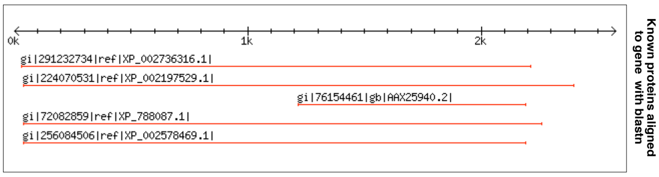

Gene_00088

Supercontigs mapped to this gene

v31.002296+, v31.003200+, v31.021986-, v31.023427+, v31.026915+

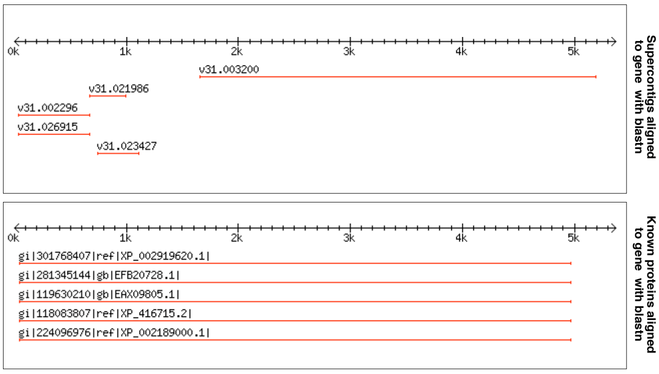

Gene_00890

Supercontigs mapped to this gene

v31.001266+, v31.006490-, v31.007080-, v31.027138+

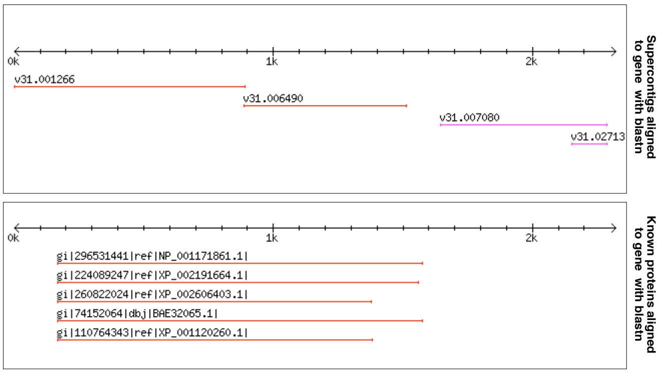

Gene_00887

Supercontigs mapped to this gene

v31.002613-, v31.005609+, v31.014270-, v31.015747+, v31.016118+, v31.023219-, v31.025995-, v31.040642+

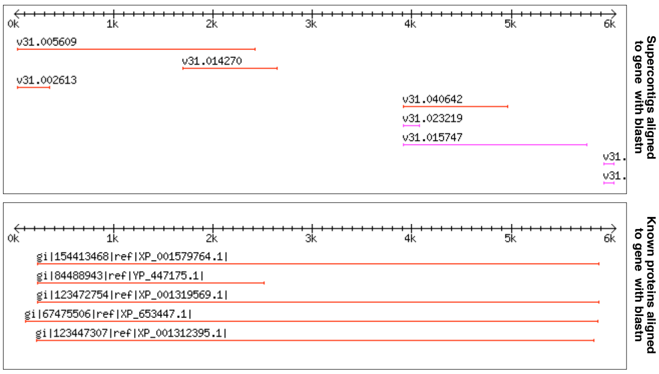

Gene_00891

Supercontigs mapped to this gene

v31.001298+, v31.007154-, v31.026652-

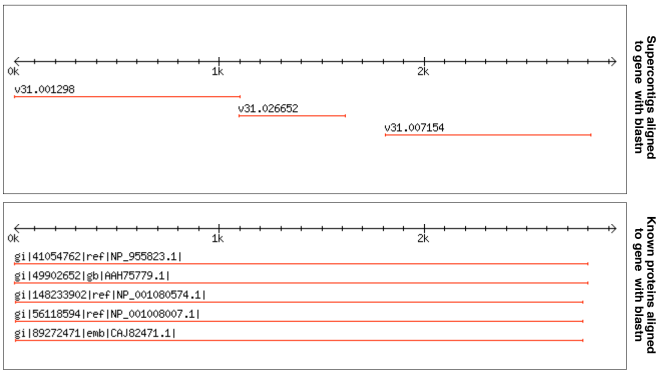

Gene_08934

Supercontigs mapped to this gene

v31.001060+, v31.015283-

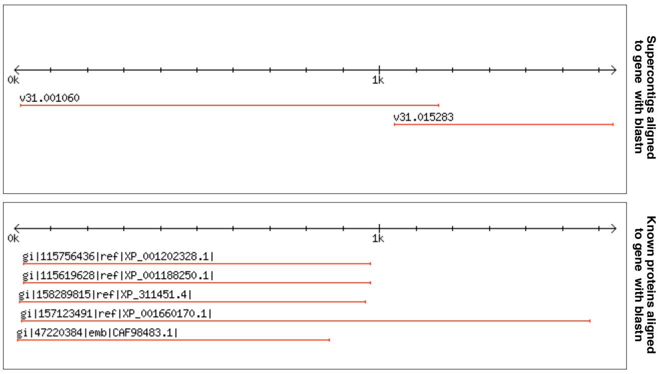

Gene_08950

Supercontigs mapped to this gene

v31.000504-, v31.003980-, v31.010962-

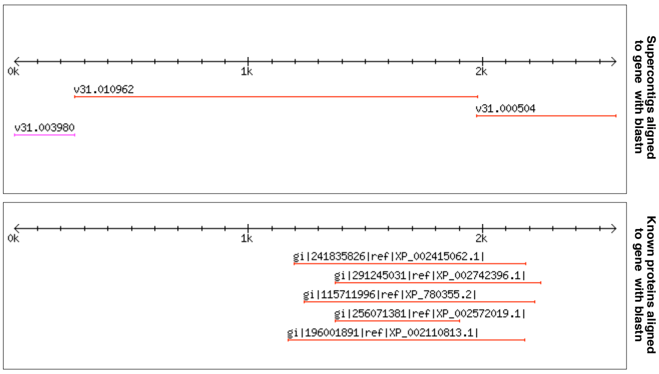

Gene_00894

Supercontigs mapped to this gene

v31.000204-, v31.005135+

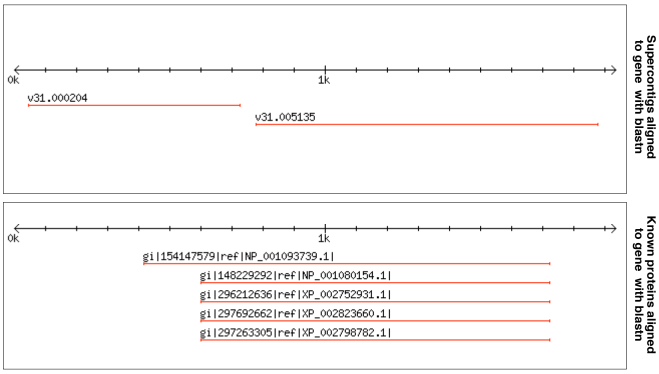

Gene_09104

Supercontigs mapped to this gene

v31.000566-, v31.012762-, v31.015667-, v31.021199+

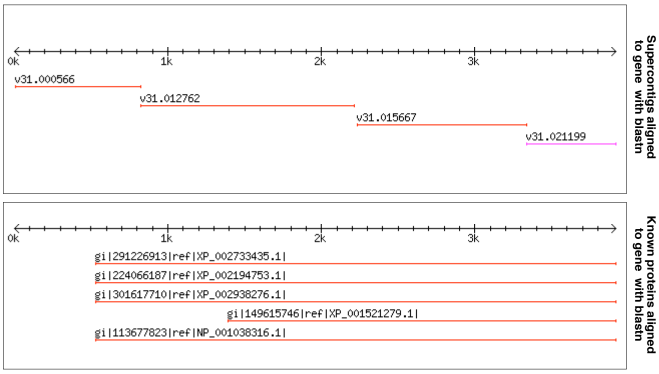

Gene_09107

Supercontigs mapped to this gene
v31.000799+, v31.000804+

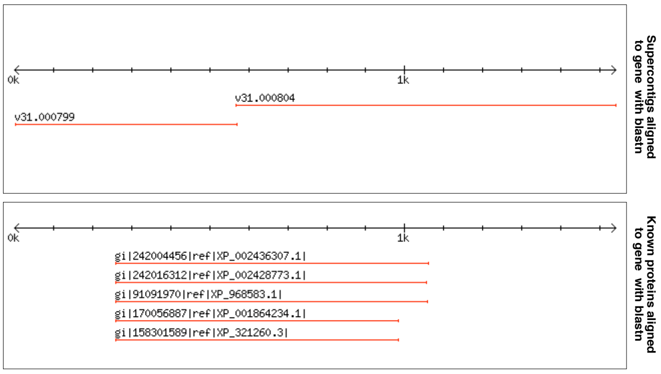

Gene_09187

Supercontigs mapped to this gene
v31.004511+, v31.007935-

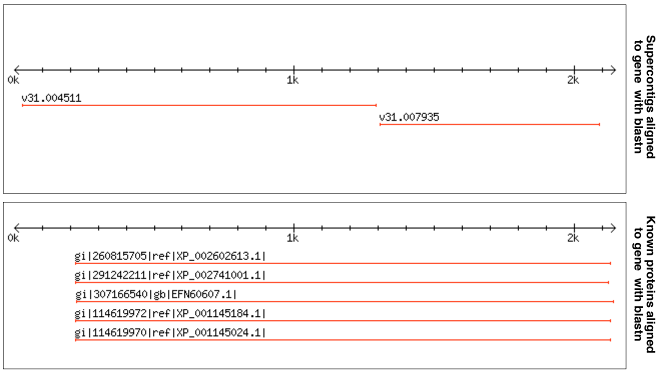

Gene_09114

Supercontigs mapped to this gene
v31.000839-, v31.007418-, v31.018384-, v31.023884+, v31.029251+, v31.036511-

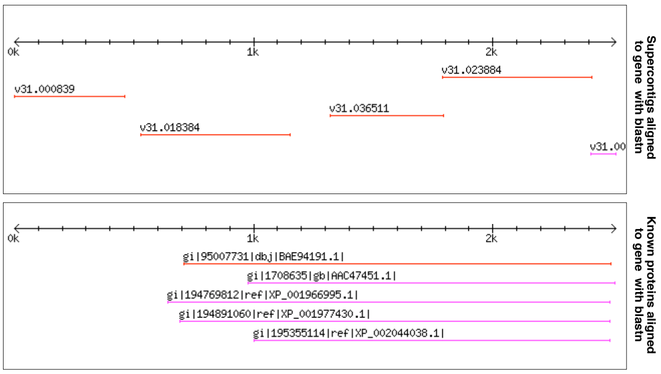

Gene_09210

Supercontigs mapped to this gene
v31.002214+, v31.020319+, v31.027881-, v31.032611+, v31.041909+

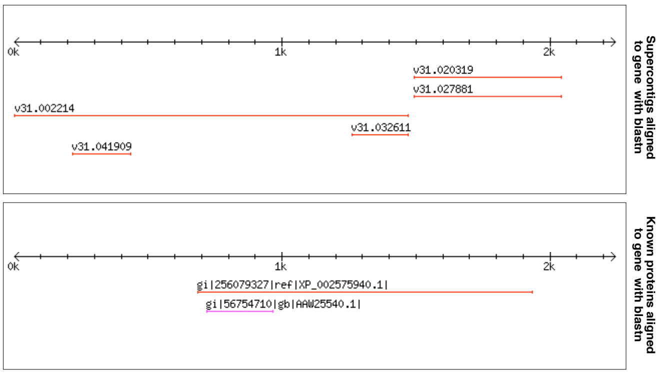

Gene_09225

Supercontigs mapped to this gene
v31.005395+, v31.008808-

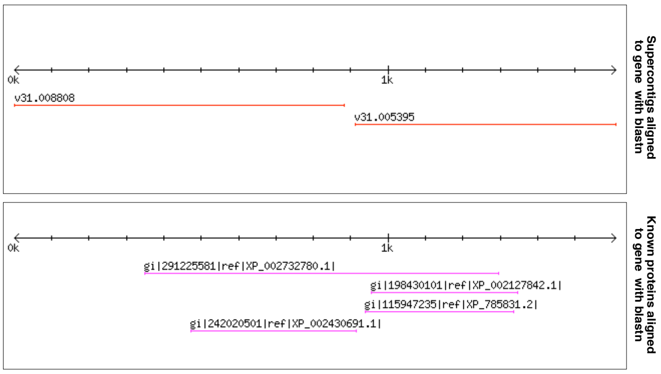

Gene_09302

Supercontigs mapped to this gene
v31.001435+, v31.010606-

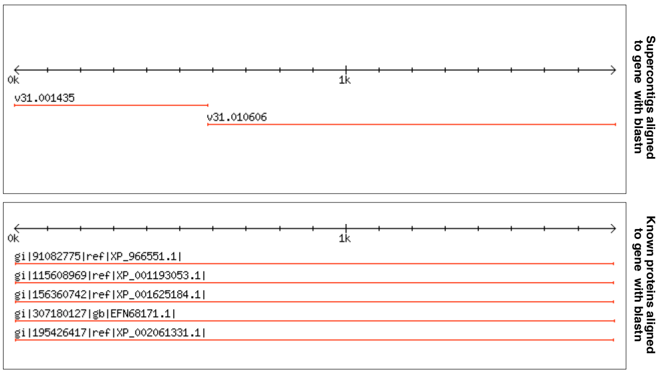

Gene_09301

Supercontigs mapped to this gene
v31.015473-, v31.019537+, v31.022343+, v31.023885-, v31.024841+, v31.028601+, v31.077666+

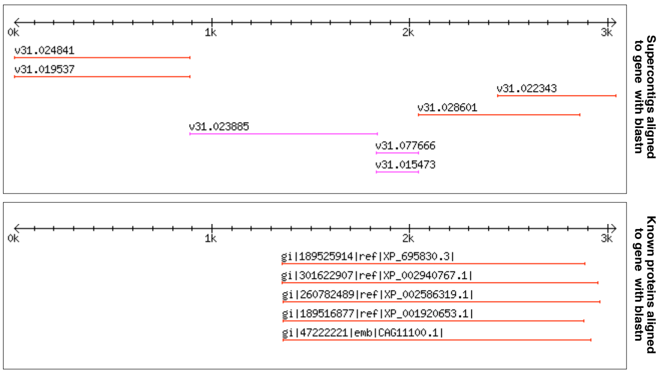

Gene_00936

Supercontigs mapped to this gene
v31.000652-, v31.008407-, v31.009584+, v31.034488+

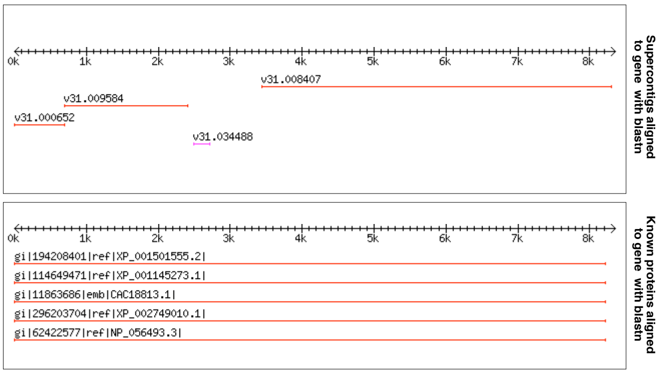

Gene_09387

Supercontigs mapped to this gene
v31.000281-, v31.009785-

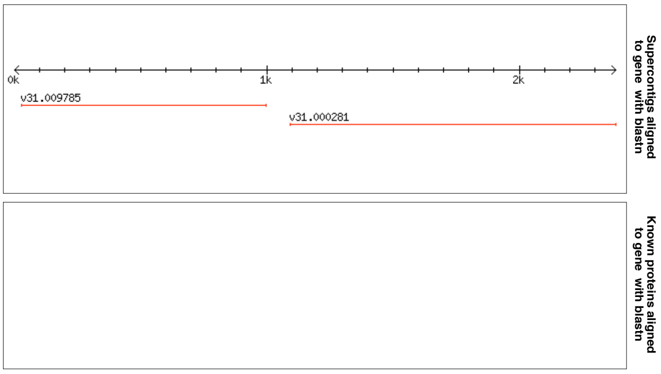

Gene_09487

Supercontigs mapped to this gene
v31.008133-, v31.014984-, v31.019776+

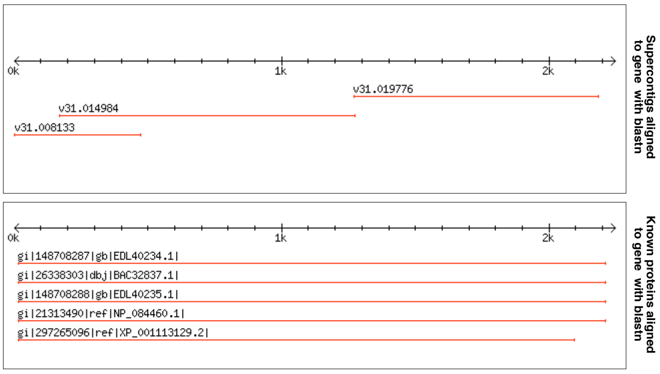

Gene_00939

Supercontigs mapped to this gene
v31.000021+, v31.003899-

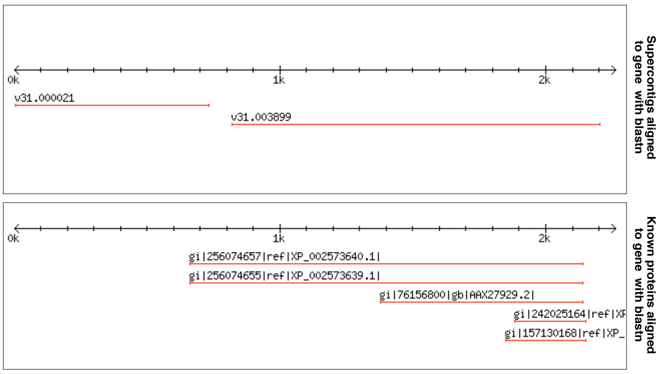

Gene_09567

Supercontigs mapped to this gene
v31.004577+, v31.011359+

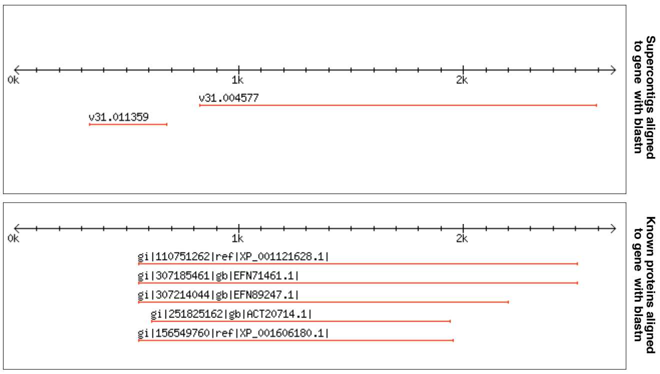

Gene_09593

Supercontigs mapped to this gene
v31.003180+, v31.005630-

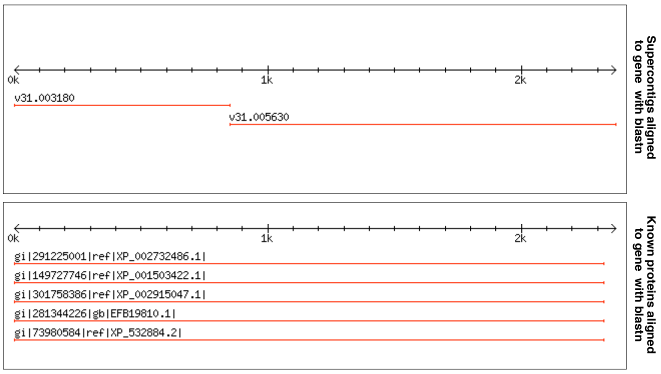

Gene_00970

Supercontigs mapped to this gene
v31.007103+, v31.007400-

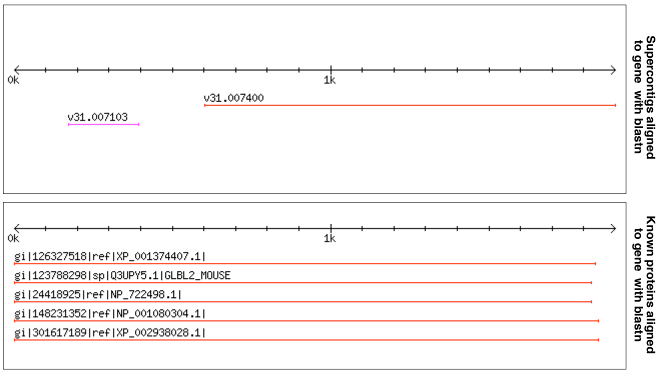

Gene_00967

Supercontigs mapped to this gene
v31.005094-, v31.010728+, v31.013658-, v31.016871+,
v31.020887+, v31.024134+, v31.029072+

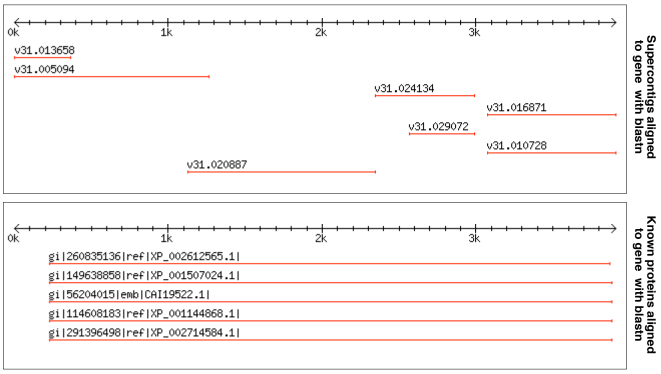

Gene_00973

Supercontigs mapped to this gene
v31.002454+, v31.017543+

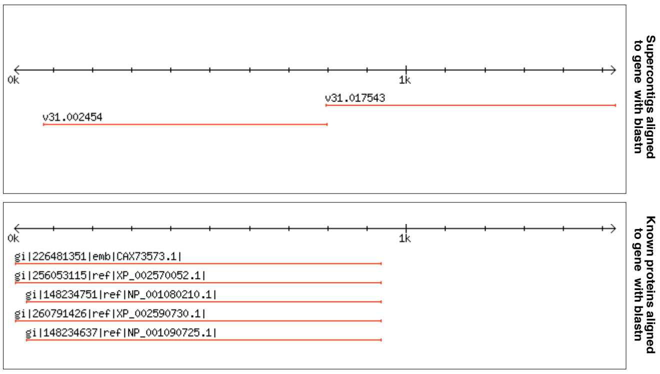

Gene_09753

Supercontigs mapped to this gene

v31.006225-, v31.012945-, v31.015062+, v31.021568+

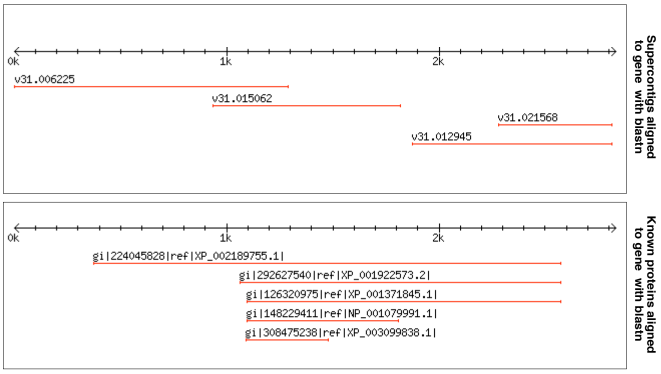

Gene_09941

Supercontigs mapped to this gene

v31.000703-, v31.008332+

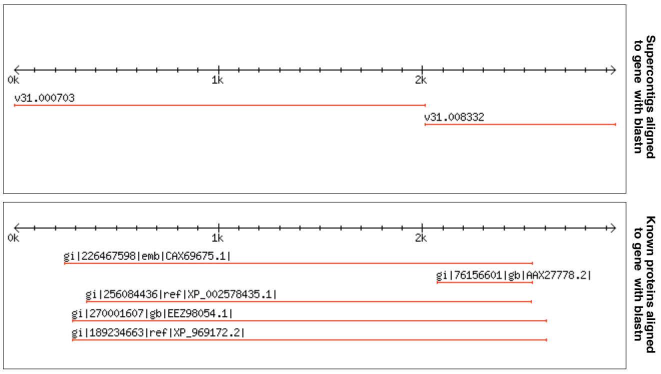

Gene_09906

Supercontigs mapped to this gene

v31.000538-, v31.000810+, v31.000873-

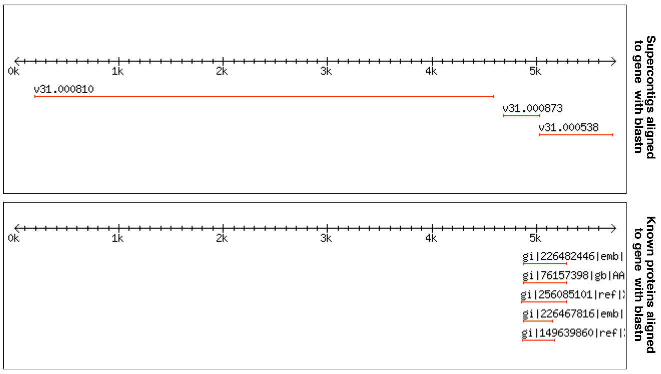

Gene_00999

Supercontigs mapped to this gene

v31.001617+, v31.003814+

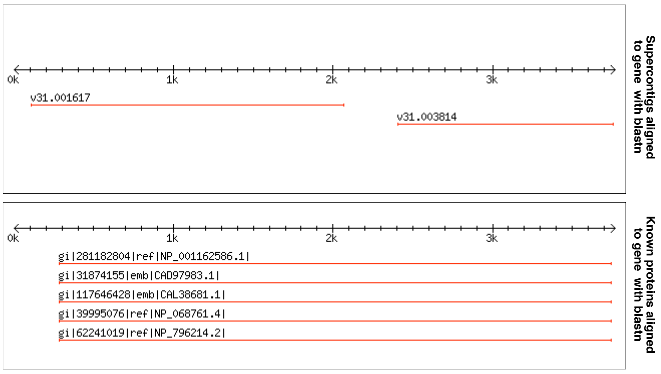

Gene_09996

Supercontigs mapped to this gene

v31.009653+, v31.010644+

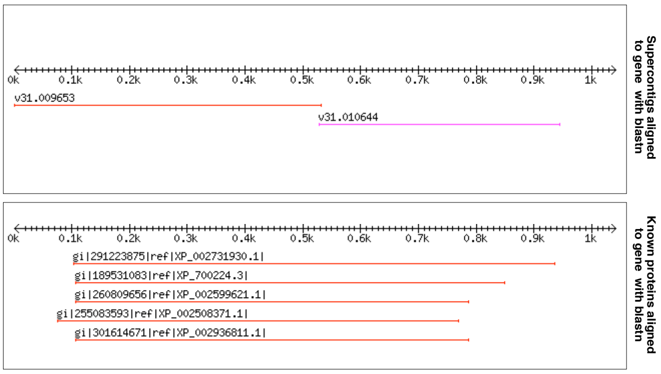

Supplement: Additional file 3 — Identification of supercontig-spanning transcripts. Illustration of 413 high-confidence supercontig-joining transcripts identified, with a description of the procedure. [file gb-2011-12-8-r76-S3.PDF]
