# Supplementary material for: The head-regeneration transcriptome of the planarian Schmidtea mediterranea
Source: Genome Biol. 2011 Aug 16;12(8):R76. doi: 10.1186/gb-2011-12-8-r76 (PMC3245616; doi:10.1186/gb-2011-12-8-r76)

Amplification of a continuous sequence connecting supercontigs v31.005068 and v31.000152 (reverse complement)

A PCR amplification with primers annealing to different supercontigs

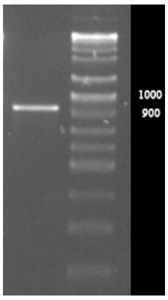

forward primer: GCCTGGTTTTCCTAGTTTGAAC
reverse primer: CAATGAGCGGTTCTCATTAGAC

B Amplicon, full sequence

TGAACTGTGACGGATCCAAAGTTTATATCAGCTTTTGGCGTAATTTGGTACTTGCTATAAAA
TGATCTGGCGGAGATTCTGATCGGAATTATTGCTATGACCTCTCCTTGGTCTGCTATATTCG
GTTCTATGACATGGCACTTCAATATCTGCTCGTCTTTGATCACTACTTCCTCCTTGGCACAA
TATGTTATTTGGACTTGGATAGCTTTTTCTCCAGGCGACATTTTCCCCTGACTCGGAGaCAA
TGTAAACAGGTTAGATAAGCTATTCTTTCGATTCTCTGAGCCGTCCAACTCGAACTTGTATT
CAATTTCATAGGGGCCTCGGTTTTTTAGGGTAATCATTTGCTTGGTCTCGTCGCCAACCTTC
AATGTTCCCATGTCGAGCCCGTTATTTGCTCCTTTAGGAAAACTCATATCCAGTGCCACATC
ATATGCTTCTGCAAGTATTTGCACAGTTTCTGTCTGCAAGATTCCCGCCAACTCCTCTACGT
CCGATATTTCGAGCCGAATATTCTTTTTTGTATTCGCCTTATACGGTTTCATTGCACGGAAA
TATGCATTTAATTTAAACTCGGAAAGAGCGTCAATCGTTCCCCCGTCCTGAGCAAATGAGAA
TTCATCACCAAGACTCTCCAGTCCGGTAATTCTCCAGTGGACTGGAAGAGCAGTACTATTTC
GTAAGTATACAGTTTTCATATCTTTCCTGTGAAGGAGAACTCTCTCGAAATGGATGACCTTA
GTGTCCAGTTCAACAAAAGGTGACACTCCGAGACAGGAAATCTTGTAGATTACGGGTTCAGG
GTTTTCTTTCACGCAGCAGATTATTGCATCTTCAAACTTCTGGGCAAATCTTGGGAAAGCCC
ACAGATTCAATTTTTGAGATTCTTCTGGTTGCAAAGTCATACTTGGGGGGTCTAA

C Blastn alignment to supercontigs, overview

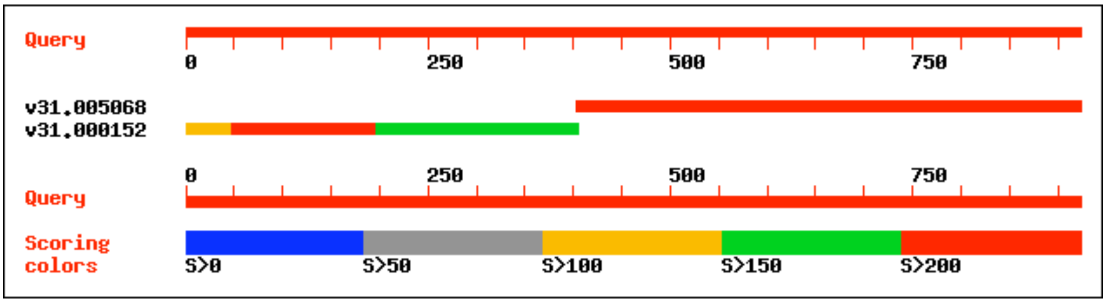

Supplement: Additional file 4 — Experimental validation of the continuity of genomic supercontigs v31.005068 and v31.000152. (a) Agarose gel electrophoresis showing the PCR amplicon produced with forward and reverse primers annealing to supercontigs v31.005068 and v31.000152, respectively. (b) The amplicon sequence was verified by Sanger sequencing. (c) Blastn results showing the 5' end of the sequence aligning to supercontig v31.000152 and the 3' end matching supercontig v31.005068. [file gb-2011-12-8-r76-S4.PDF]
