# Supplementary material for: The head-regeneration transcriptome of the planarian Schmidtea mediterranea
Source: Genome Biol. 2011 Aug 16;12(8):R76. doi: 10.1186/gb-2011-12-8-r76 (PMC3245616; doi:10.1186/gb-2011-12-8-r76)
Supplement: Additional file 5 — Experimental validation of detected transcripts by RT-PCR. (a) Agarose gel electrophoresis of amplicons amplified using primers designed against 14 different sequences from the Illumina+ assembly and two control sequences. (b) Primer sequences used in the PCRs. [file gb-2011-12-8-r76-S5.PDF]

A

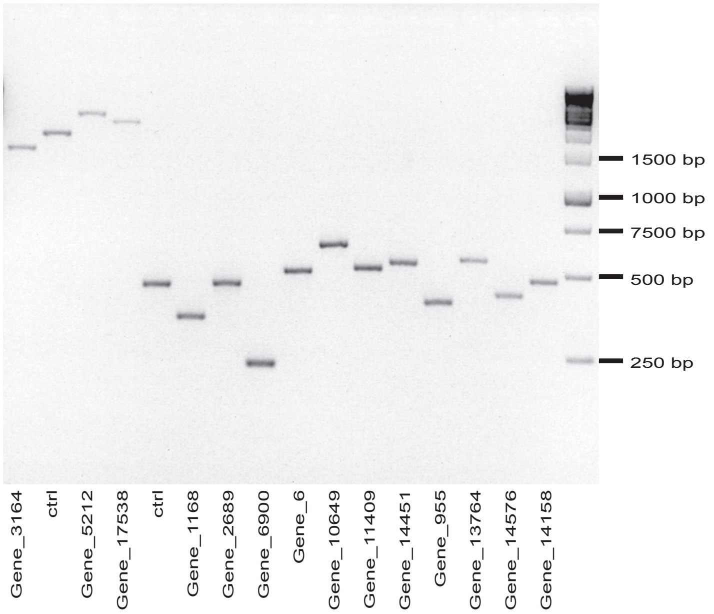

B

Transcript	Forward primer	Reverse primer
Gene_3164	ACAGATTTATTATCCATGCCTGA	ACTGGTCAATAGTTCTGATAGTT
Gene_5212	TACTCAAGTCTCAAATGCTAGA	CTTATGCGTAGGTTGCTTGT
Gene_17538	CAAGGTTACAGTCGACGGTCC	AGGTCTCCAAATCTTGGTCTTATCA
Gene_1168	GGTACGCGATGGAGCTCCAG	AACGCAACTAACTCCGGCTGAT
Gene_2689	ATGGGGAGGTCAGACAGAA	CATTCGATCGAACCGACTA
Gene_6900	ATCGAGACATCCGAAGAGAT	GGAATTAACTGACTGAGCCAA
Gene_6	CGCGATCTGCCAGACATTGAA	GGATCTCGGATCTGTGGAA
Gene_10649	GATTGATAGCCGGTTCCCGAT	CTAATTCCGCTTGGACTTGTT
Gene_11409	CGCCTTAGTCGTAATGGCAT	GTTGAACCTGTCATATCATCTA
Gene_14451	CACCAAGAATAGAAAAGTGCAA	CAGGAGAACATTCAGAACAGTT
Gene_955	CAAACTGTAGAGCTCATGGATA	TTCTTCAGCTGGTCCCGTCTGCT
Gene_13764	CCGATATTGACGAGACCTT	TTCAGAATGTTTCCCATCAT
Gene_14576	CAGAAACTGAAATACAAATCCCA	GACCTTGTTAACAATCTCATCCT
Gene_14158	CGCGAAAGATACATTTGGCAA	TTCAACTAAATGGACATAATTCTT
