# Supplementary material for: The head-regeneration transcriptome of the planarian Schmidtea mediterranea
Source: Genome Biol. 2011 Aug 16;12(8):R76. doi: 10.1186/gb-2011-12-8-r76 (PMC3245616; doi:10.1186/gb-2011-12-8-r76)
Supplement: Additional file 10 — Gene ontology enrichment analysis for each temporal expression class. Gene ontology term enrichment and depletion of each cluster compared to the full set of annotated sequences using GOSeq. [file gb-2011-12-8-r76-S10.DOC]

Of the total **1142** differentially expressed genes reproducibly clustered into the five temporal classes, 273 (23.9.0%) could be annotated with one or more GO slim terms with the Blast2GO suite using default parameters. Categories from the “molecular function” and “biological process” gene ontologies assigned to more than 3 different genes were tested for statistical enrichment or depletion compared to the full set of annotated sequences with the Bioconductor GOSeq package, using the Wallenius approximation and correcting for potential biases due to non-random sequence-length distributions. Categories enriched or depleted with p<0.05 (before Bonferroni correction) are reported in the tables show below.

** indicate p-values that remain <0.05 after Benjamini-Hochberg correction for multiple testing

**Table A: All 1142 clustered genes**

| GO category | Description | Raw p-value |  |
| --- | --- | --- | --- |
| GO:0004252 | serine-type endopeptidase activity | 0.00048** | Enriched |
| GO:0031667 | response to nutrient levels | 0.0044 | Enriched |
| GO:0003774 | motor activity | 0.0061 | Enriched |
| GO:0004867 | serine-type endopeptidase inhibitor activity | 0.0128 | Enriched |
| GO:0005506 | iron ion binding | 0.0249 | Enriched |
| GO:0050804 | regulation of synaptic transmission | 0.0267 | Enriched |
| GO:0005215 | transporter activity | 0.0297 | Enriched |
| GO:0043565 | sequence-specific DNA binding | 0.0342 | Enriched |
| GO:0003678 | DNA helicase activity | 0.0347 | Enriched |
| GO:0006508 | proteolysis | 0.0392 | Enriched |
| GO:0006811 | ion transport | 0.0480 | Enriched |
| GO:0005525 | GTP binding | 0.0241 | Depleted |
| GO:0017111 | nucleoside-triphosphatase activity | 0.0192 | Depleted |
| GO:0005488 | binding | 0.0010** | Depleted |
| GO:0005524 | ATP binding | 0.0005** | Depleted |
| GO:0006468 | protein phosphorylation | 0.0001** | Depleted |

**Table B: Cluster 1, 213 genes**

| GO category | Description | Raw p-value |  |
| --- | --- | --- | --- |
| GO:0004197 | cysteine-type endopeptidase activity | 0.0111 | Enriched |

**Table C: Cluster 2, 224 genes**

| GO category | Description | Raw p-value |  |
| --- | --- | --- | --- |
| GO:0007186 | G-protein coupled receptor protein signaling pathway | 0.0398 | Enriched |

**Table D: Cluster 3, 286 genes**

| GO category | Description | Raw p-value |  |
| --- | --- | --- | --- |
| GO:0048232 | male gamete generation | 0.0006** | Enriched |
| GO:0004867 | serine-type endopeptidase inhibitor activity | 0.0019** | Enriched |
| GO:0031667 | response to nutrient levels | 0.0067** | Enriched |
| GO:0048806 | genitalia development | 0.0195 | Enriched |
| GO:0005524 | ATP binding | 0.0459 | Depleted |

**Table E: Cluster 4, 208 genes**

| GO category | Description | Raw p-value |  |
| --- | --- | --- | --- |
| GO:0050804 | regulation of synaptic transmission | 0.0054** | Enriched |
| GO:0005215 | transporter activity | 0.0058** | Enriched |
| GO:0005216 | primary metabolic process | 0.0405 | Enriched |

**Table F: Cluster 5, 211 genes**

| GO category | Description | Raw p-value |  |
| --- | --- | --- | --- |
| GO:0004252 | serine-type endopeptidase activity | 0.00038** | Enriched |
| GO:0006811 | ion transport | 0.0236 | Enriched |
| GO:0006508 | proteolysis | 0.0428 | Enriched |
| GO:0007165 | signal transduction | 0.0464 | Enriched |
