# Supplementary material for: The head-regeneration transcriptome of the planarian Schmidtea mediterranea
Source: Genome Biol. 2011 Aug 16;12(8):R76. doi: 10.1186/gb-2011-12-8-r76 (PMC3245616; doi:10.1186/gb-2011-12-8-r76)

A

```
             :* *. * ..*: ** ***.**:**  *:*:.         : **  * :
Danio rerio  LVRTDSPNFLCSVLPSHWRCNKTLPVAFKVVAL------GDVPDGTLVTV
Homo sapiens LVRTDSPNFLCSVLPSHWRCNKTLPVAFKVVAL------GEVPDGTVVTV
Hydra        LVKTDSPNFVCSALPSHWRCNKTLPMAFKVIALS-----GDIPDGVTVTI
Dmel         LVRTSNPYFLCSALPAHWRSNKTLPMAFKVVAL------AEVGDGTYVTI
Smed         IVPTSHPLVFCTKLPQHWRSNKSLPALFQVMVYQSNNRVLPIGDGHRVWL

              *.*  .   .* ***  : : *  *:******:.****** * : * :
Danio rerio  MAGNDENYSAELRNASAVMKNQVARFNDLRFVGRSGRGKSFTLTITVFTG
Homo sapiens MAGNDENYSAELRNASAVMKNQVARFNDLRFVGRSGRGKSFTLTITVFTN
Hydra        FAGNDDNFSAELRNATAVMKNQVARFNDLRFVGRSGRGKTFTLTITVNSE
Dmel         RAGNDENCCAELRNFTTQMKNDVAKFNDLRFVGRSGRGKSFTLTITVATS
Smed         SASNPSMNSAVLRNFESKLVNGEARFNDLRFISRSGRGKYFDVYIHIECN

             *  :*.* .*************
Danio rerio  PPQVATYHRAIKVTVDGPREPRRH
Homo sapiens PPQVATYHRAIKVTVDGPREPRNP
Hydra        PPQVATYTRAIKVTVDGPREPRRH
Dmel         PPQVATYAKAIKVTVDGPREPRSK
Smed         PKIIAVYTNAIKVTVDGPREPRNK
```

B

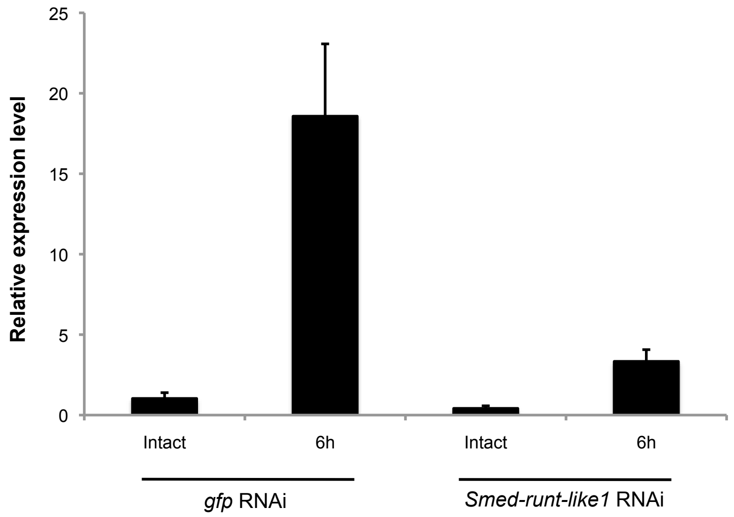

C

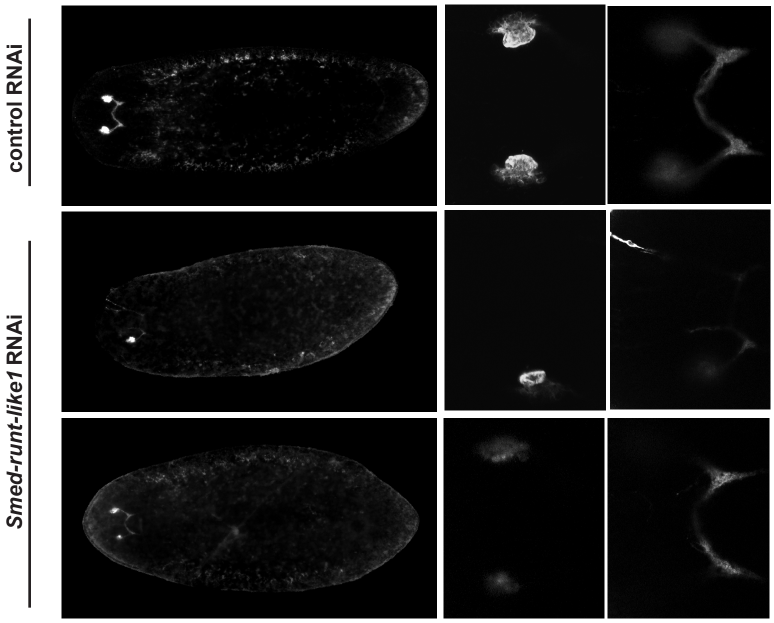

Supplement: Additional file 11 — Conservation, knockdown efficiency, and RNAi phenotype of smed-runt-like1. (a) Alignment (ClustalX) of Runt domains of proteins from vertebrate and invertebrate species: Danio rerio (Runt-related transcription factor 3, NP_571679); Homo sapiens (Runt-related transcription factor 2, EAX04279); Hydra magnipapillata (Runx, XP_002165633); Drosophila melanogaster (Lozenge, NP_001096919); Schmidtea mediterranea (Smed-Runt-like1, JF720854). The planarian Runt domain shares at least 45% sequence identity with Runt domains from other organisms. (b) qRT-PCR analysis of smed-runt-like1 expression levels in intact and regenerating planarians 6 h after head and tail dissection. Prior to dissection, animals had been injected with three pulses of 32.2 nl of a 1.5 μg/μl dsRNA solution containing either dsRNAs against smed-runt-like1 or a control gene (gfp), for three days in a row for two consecutive weeks (day 1 to 3, 8 to 10; cut on day 11). Expression levels were normalized against those of a housekeeping gene (AY068123). Error bars represent standard deviations of the mean of three independent biological replicates of five worms each. Note that the smed-runt-like1 mRNA levels are reduced but not completely abolished upon RNAi in regenerating animals. (c) Immunofluorescence analysis (anti-Arrestin) of photoreceptor neurons in regenerating control and smed-runt-like1 RNAi animals at day 14 after decapitation. Note that smed-runt-like1 RNAi animals have severe eye patterning defects. Animals were treated as described in the Materials and methods section of the main text. [file gb-2011-12-8-r76-S11.PDF]
