# Supplementary material for: The head-regeneration transcriptome of the planarian Schmidtea mediterranea
Source: Genome Biol. 2011 Aug 16;12(8):R76. doi: 10.1186/gb-2011-12-8-r76 (PMC3245616; doi:10.1186/gb-2011-12-8-r76)
Supplement: Additional file 15 — Primers used for experimental validation of expression profiles by qRT-PCR. Primer sequences used for qRT-PCR analysis. [file gb-2011-12-8-r76-S15.PDF]

Transcript	Forward primer	Reverse primer
Gene_3442	TTGTAGAATGTTGGGTATTTCTGG	CAACTCCCGATCCAAATCTAGT
Gene_5777	CAGACTGGATATACCCCATTGC	AACTCAATGTCTCTGCAATCCA
Gene_17538	GCTGATGGCAACGACACTT	TGATGGGATTGCGAGTTGTA
Gene_4339	ATGTCACCGAAAGAAATCGAG	TCTCGACAATAAACCGACGA
Gene_343	TCAATTGGTGACTGTCCGACT	GCAACATCATTGCCGTAACA
Gene_3164	AATATACCCCAATGGCGATG	TTGAACAATCATGAAATAAACTGACA
Gene_1168	TTTGTGGGCTACCTGATGAA	GGGTCGACTTTTGCAGTAGG
Gene_2782	TTGGCACAGAAACTTAACATGG	TTGCATGAGATCAACGATCC
Gene_5212	GCCAATCGACAGATCCTTTG	TCGACTATATTCCGGTTGAAACT
Gene_12046	TGATCTACACAGTGCTGGTTCAC	GAAAGCTGAGGCTTTTTAGCAA
